# Phylum-Level Conservation of Regulatory Information in Nematodes despite Extensive Non-coding Sequence Divergence

**DOI:** 10.1371/journal.pgen.1005268

**Published:** 2015-05-28

**Authors:** Kacy L. Gordon, Robert K. Arthur, Ilya Ruvinsky

**Affiliations:** 1 Department of Organismal Biology and Anatomy, The University of Chicago, Chicago, Illinois, United States of America; 2 Department of Ecology and Evolution, The University of Chicago, Chicago, Illinois, United States of America; University of California Davis, UNITED STATES

## Abstract

Gene regulatory information guides development and shapes the course of evolution. To test conservation of gene regulation within the phylum Nematoda, we compared the functions of putative cis-regulatory sequences of four sets of orthologs (*unc-47*, *unc-25*, *mec-3* and *elt-2*) from distantly-related nematode species. These species, *Caenorhabditis elegans*, its congeneric *C*. *briggsae*, and three parasitic species *Meloidogyne hapla*, *Brugia malayi*, and *Trichinella spiralis*, represent four of the five major clades in the phylum Nematoda. Despite the great phylogenetic distances sampled and the extensive sequence divergence of nematode genomes, all but one of the regulatory elements we tested are able to drive at least a subset of the expected gene expression patterns. We show that functionally conserved cis-regulatory elements have no more extended sequence similarity to their *C*. *elegans* orthologs than would be expected by chance, but they do harbor motifs that are important for proper expression of the *C*. *elegans* genes. These motifs are too short to be distinguished from the background level of sequence similarity, and while identical in sequence they are not conserved in orientation or position. Functional tests reveal that some of these motifs contribute to proper expression. Our results suggest that conserved regulatory circuitry can persist despite considerable turnover within *cis* elements.

## Author Summary

To explore the phylogenetic limits of conservation of *cis*-regulatory elements, we used transgenesis to test the functions of enhancers of four genes from several species spanning the phylum Nematoda. While we found a striking degree of functional conservation among the examined *cis* elements, their DNA sequences lacked apparent conservation with the *C*. *elegans* orthologs. In fact, sequence similarity between *C*. *elegans* and the distantly related nematodes was no greater than would be expected by chance. Short motifs, similar to known regulatory sequences in *C*. *elegans*, can be detected in most of the *cis* elements. When tested, some of these sites appear to mediate regulatory function. However, they seem to have originated through motif turnover, rather than to have been preserved from a common ancestor. Our results suggest that gene regulatory networks are broadly conserved in the phylum Nematoda, but this conservation persists despite substantial reorganization of regulatory elements and could not be detected using naïve comparisons of sequence similarity.

## Introduction

Similar expression patterns of orthologous genes imply similarity of developmental programs in different species. Numerous such examples have been uncovered, including *hox* [1], *dlx* [2], and *dpp/BMP* [3] genes, as well as genetic programs regulating photoreceptor [4] and muscle [5] development in distantly related bilaterian animals. Largely based on these and similar findings, a current view of evolution of development emerged that emphasizes the conservation of the genetic “toolkit” within animals and the relative importance of regulatory changes in driving morphological change [6].

The mechanisms responsible for expression pattern conservation are less clear, however. One possibility is that ancestral gene regulatory programs are strictly retained. An alternative is that expression similarity is mediated by divergent regulatory processes [7,8], a phenomenon known as “developmental system drift” [9]. Regulatory rewiring of the latter type is known to occur even when individual components of the diverged networks are highly conserved developmental regulators [10–12]. One way to probe the evolution of regulatory linkages is with enhancer swap experiments, in which *cis*-regulatory DNA from one species is used to drive expression of a reporter gene in another species (reviewed in [13]). The resulting pattern of gene expression can be compared to the pattern driven by the endogenous regulatory element, with the similarities and differences giving evidence of conservation and divergence in the gene regulatory network.

We wanted to assess the conservation of gene regulatory programs among distantly-related members of the phylum Nematoda, a group of morphologically similar worms with mostly small, vermiform bodies. This body plan is largely conserved, with numbers of certain neuronal subtypes nearly identical in even deeply diverged taxa [14,15], and the intestine arising from a clonal cell lineage [16] in most (but not all, see [17]) nematodes studied. However, instances of developmental divergence have been documented in this clade [18–23]. We therefore performed enhancer swap experiments with regulatory elements of genes expressed in two subsets of neurons and in the developing intestine. By examining the function of *cis* regulatory sequences from four different nematode species in transgenic *C*. *elegans*, we sought to determine the extent of *cis*-regulatory conservation within this phylum.

## Results

### Selection of species and genes

The phylum Nematoda is comprised of animals with simple vermiform body plans and diverse life-history strategies. To look for evidence of gene regulatory conservation across this phylum, we carried out a series of enhancer-swap experiments between several distantly-related nematodes and a *C*. *elegans* host. Regulatory regions from orthologous *C*. *elegans* genes driving the *mCherry* reporter were co-expressed as controls with the exogenous *cis* elements driving expression of the *GFP* gene. This approach allows us to isolate and compare *cis-*regulatory functions of the two orthologous regulatory elements in a common *trans*-regulatory background. Any observed differences can then be attributed to the divergence of the *cis-*regulatory DNA.

**Fig 1:**
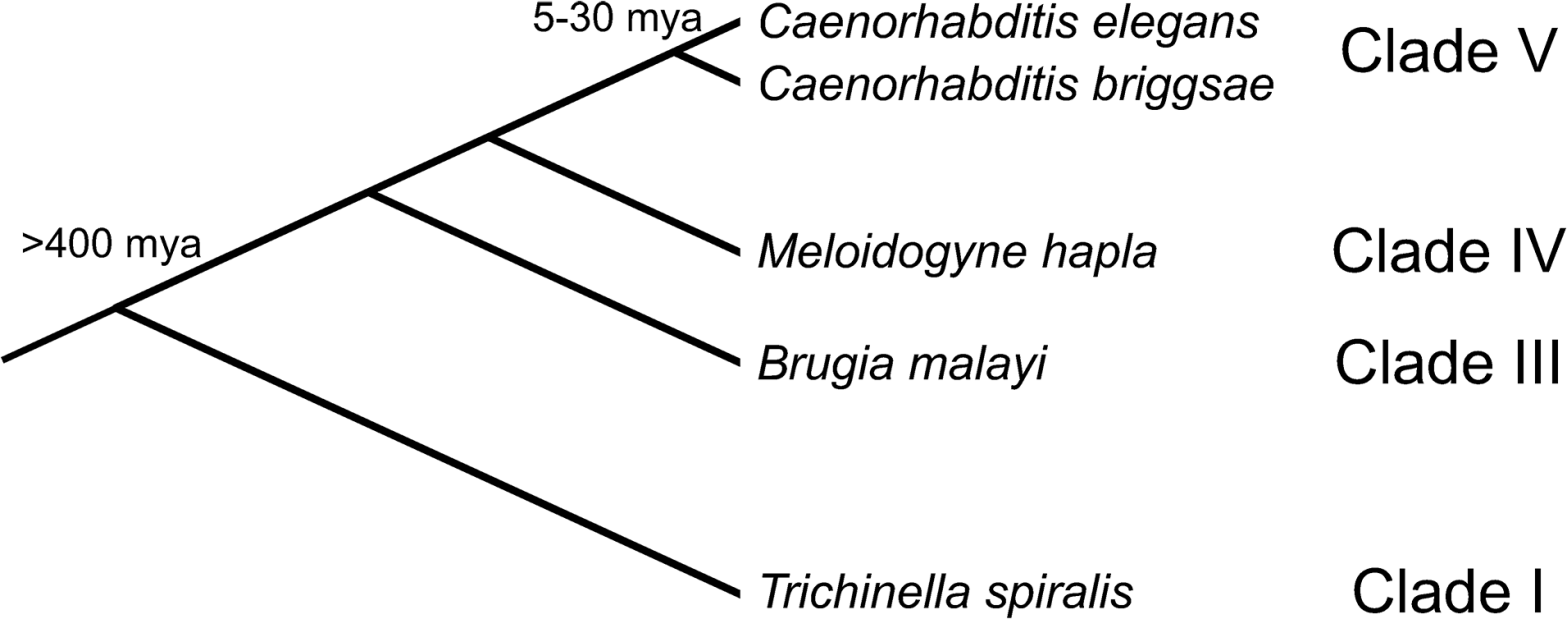
Phylogenetic relationships between the species used in this study. Topology of the tree and clade assignments are after [25].

We sought broad coverage of the phylum, which is hypothesized to have diversified in the Silurian [24]. Representatives from two basally branching nematode groups have sequenced genomes [25]. These are the Chromodorea (comprised of Clades III-V) and the Dorylaimia (Clade I). No Enoplia (Clade II) genomes have been sequenced to date. For this study we used *C*. *elegans* [26] as the transgenic host species, and its congeneric *C*. *briggsae* [27] to test divergence of regulatory elements among close relatives (both are from Clade V). The next most closely related nematode species is *Meloidogyne hapla* (Clade IV, [28,29]), followed by *Brugia malayi* (Clade III, [30,31]). Finally, as a representative of Clade I, we used *Trichinella spiralis* [32]. Divergence of Clade I was one of the earliest events in nematode evolution. The relationships among these five species are shown in Fig 1. We leveraged both this phylogeny and the amenability of *C*. *elegans* to genetic manipulation to create a series of comparisons of expression of *cis-*regulatory elements from progressively more distantly-related species in transgenic *C*. *elegans*. *C*. *elegans* have been used as transgenic hosts of regulatory DNA from a number of different species (reviewed, along with similar studies using *Drosophila melanogaster*, in [13]), however, to our knowledge, this study is the first explicit test of the relationship between evolutionary relatedness and conservation of *cis-*regulatory function among a set of genes.

While our selection of species gave us unprecedented ability to test the phylogenetic limits of regulatory conservation, it also rendered reciprocal transgenesis infeasible due to the complex modes of reproduction of the parasitic species.

We selected genes that have considerable conservation of their coding sequences and are single-copy orthologs among the species. Two genes are expressed in GABAergic neurons in *Caenorhabditis* nematodes, *unc-47* [33,34] and *unc-25* [35]. We have previously investigated the evolution of their regulation within this clade [36–39]. The third gene, *mec-3*, is expressed in another neuronal cell type, the touch-receptor neurons, in *C*. *elegans* [40,41]. The regulatory region of the *C*. *briggsae* ortholog of *mec-*3 has previously been shown to drive gene expression in *C*. *elegans* [42]. Finally, we chose the gene *elt-2*, which is expressed in the endoderm [43,44], and shows evidence of regulatory conservation outside the genus Caenorhabditis [45]. These *cis-*regulatory elements are expressed in different cell types, and drive expression of terminal differentiation genes (*unc-47* and *unc-25*) as well as transcription factors (*mec-3* and *elt-2*). Where possible (see Materials and Methods), the putative regulatory regions we investigated ranged from the start of recognizable protein-coding sequence conservation with *C*. *elegans* on the 3’ end to the next upstream coding element on the 5’ end. This choice of putative regulatory sequences in no way depended on non-coding conservation between species.

### Regulatory elements from distantly-related nematodes retain some, but not all, functions when swapped into *C*. *elegans*

#### *unc-47*

**Fig 2:**
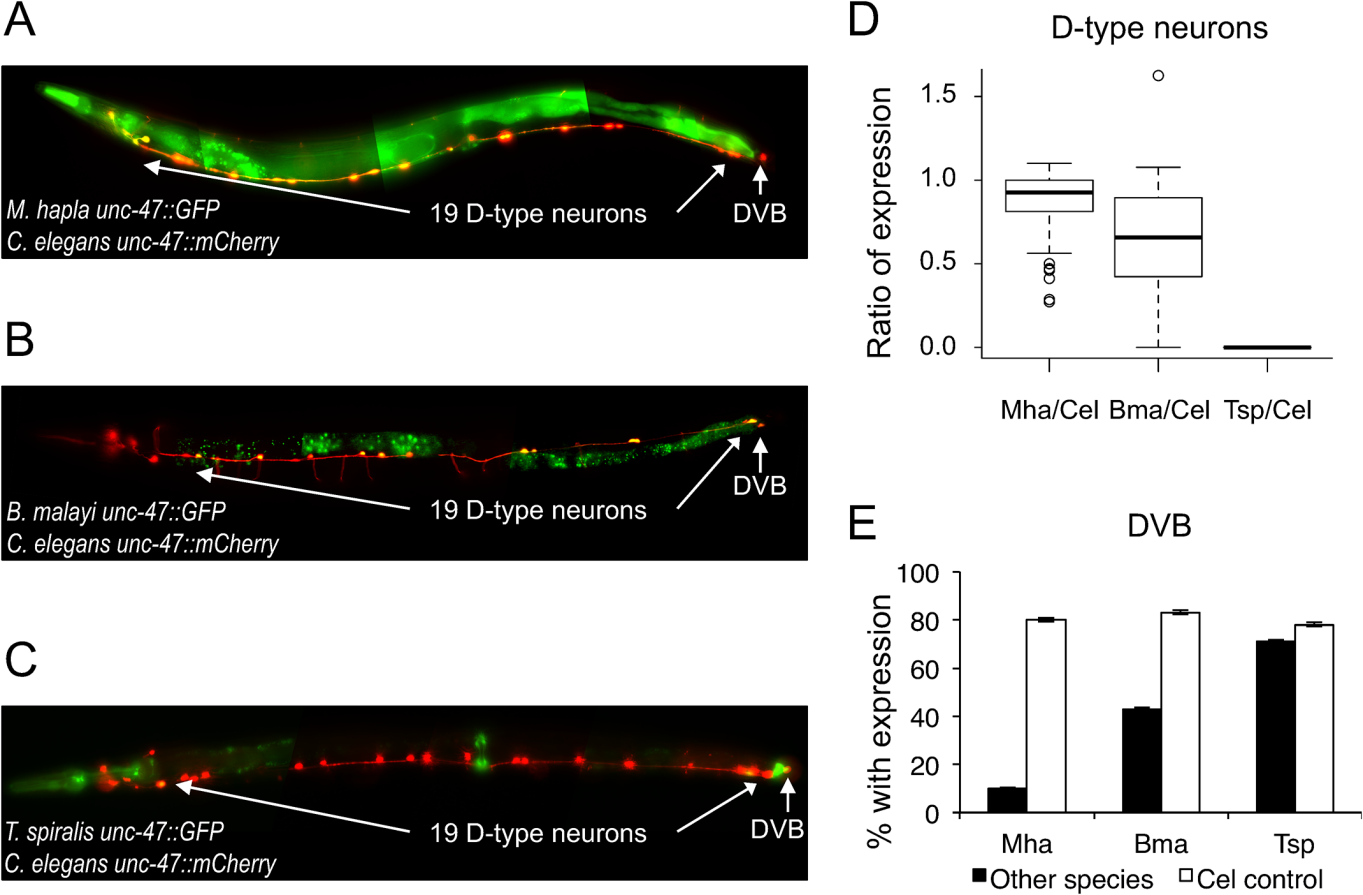
*unc-47* regulatory sequences from distantly-related nematodes drive expression in *C*. *elegans*. (A-C) *C*. *elegans unc-47* regulatory sequence drives expression of *mCherry* in all transgenic strains; (A) *M*. *hapla*, (B) *B*. *malayi*, (C) *T*. *spiralis unc-47* regulatory sequences drive expression of *GFP*. Animals were photographed at 400x magnification. Images are false-colored composites of single animals. Separate GFP and mCherry images are shown in S1 Fig. (D) Ratios of the number of D-type neurons expressing GFP/mCherry in individuals carrying indicated transgene pairs (see Materials and Methods and S1 Table for total counts). *T*. *spiralis cis-*element drives no expression in the D-type neurons. *M*. *hapla* and *B*. *malayi* differ in their fidelity to the *C*. *elegans* expression pattern (Kruskal-Wallis test, p = 2.095×10^−9^). (E) Percentage of individuals with expression in the cell DVB from the heterologous regulatory element (black) and the *C*. *elegans* regulatory element (white) for each transgene pair (see Materials and Methods and S1 Table for total counts). Error bars show 95% confidence intervals for the proportion expressing.

The *cis-*regulatory elements of the *unc-47* genes from all three distant relatives drove gene expression in *C*. *elegans* in portions of the endogenous GABAergic neuronal expression pattern (Fig 2). The cells that we examined with particular attention were the D-type neurons in the ventral nerve cord, and the post-anal neuron DVB. The *unc-47 cis-*regulatory elements from *C*. *briggsae*, *C*. *brenneri*, and *C*. *remanei*, and *C*. *japonica* all drove strong and consistent expression in these cells [36–38]. The *M*. *hapla* and *B*. *malayi unc-47 cis*-regulatory elements drove substantially weaker (S1A and S1B Fig) expression that was less consistent than that of the *C*. *elegans unc-47 cis* element (Fig 2A, 2B and 2D). The upstream region of the *T*. *spiralis* gene failed to direct expression in the D-type neurons (Figs 2C, 2D and S1C). However, expression in DVB showed the opposite pattern. Both the *M*. *hapla* and *B*. *malayi unc-47* regulatory DNA drove expression far less consistently than the *C*. *elegans* element (Fig 2E). In contrast, the *T*. *spiralis* ortholog directed bright and consistent expression in DVB that was not significantly different from *C*. *elegans* expression (Fisher’s Exact test, p = 0.3304), as well as the head neuron RIS (Figs 2C, 2E and S1D). Both of these cells are GABAergic neurons that endogenously express *unc-47* in *C*. *elegans*. *T*. *spiralis* regulatory DNA drove expression in the vulval and rectal epithelia, as well as the neuron PVT, common sites of ectopic expression), and several unidentified head neurons (Fig 2C). Expression patterns were consistent across independently generated transgenic strains (S2 Table).

#### *unc-25*

**Fig 3:**
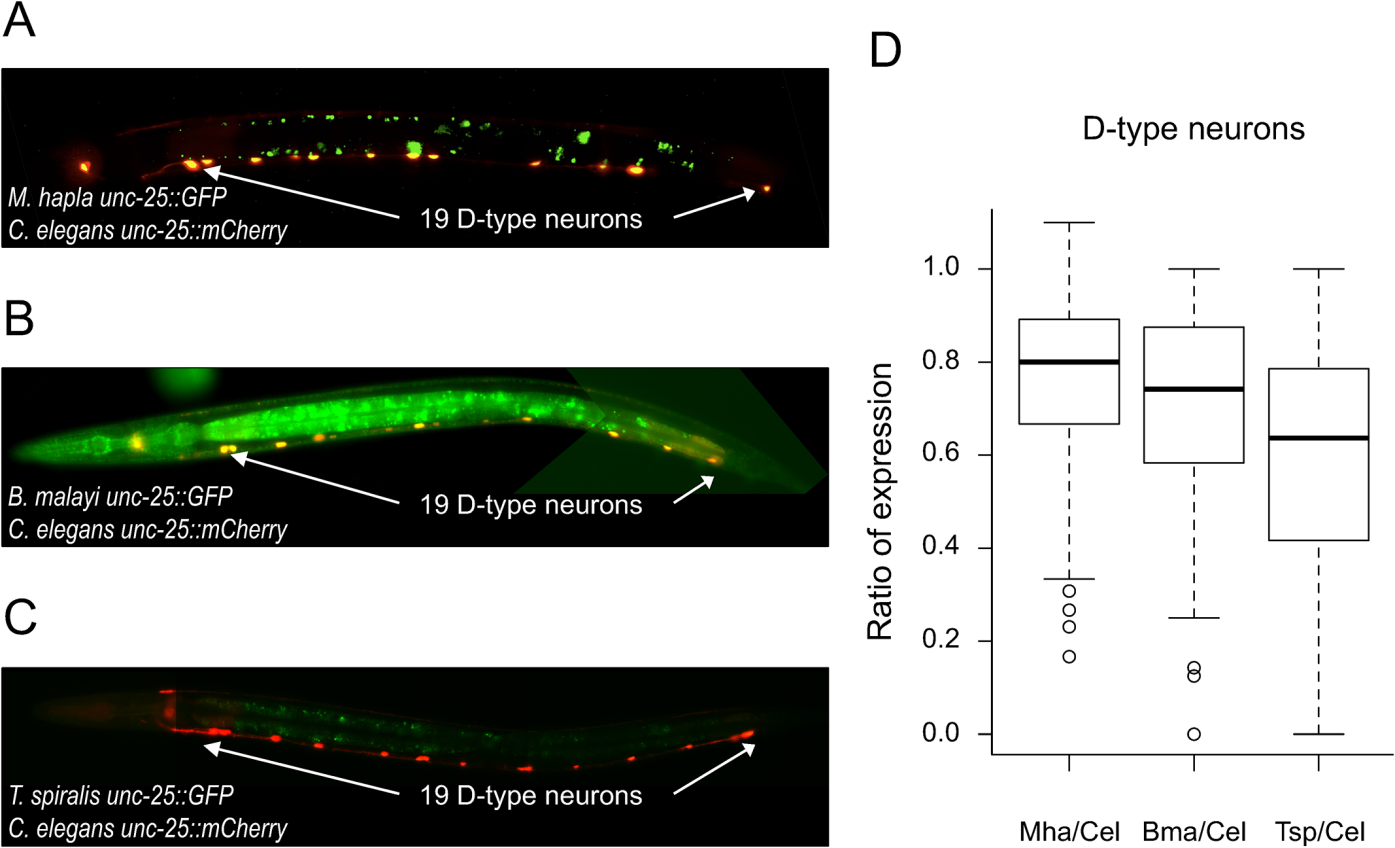
*unc-25* regulatory sequences from distantly-related nematodes drive expression in *C*. *elegans*. (A-C) *C*. *elegans unc-25* regulatory sequence drives expression of *mCherry* in all transgenic strains; (A) *M*. *hapla*, (B) *B*. *malayi*, (C) *T*. *spiralis unc-25* regulatory sequences drive expression of *GFP*. Animals were photographed at 400x magnification. Images are false-colored composites of single animals. Separate GFP and mCherry images are shown in S2 Fig. (D) Ratios of the number of D-type neurons expressing GFP/mCherry in individuals carrying each transgene pair (see Materials and Methods and S1 Table for total counts). While each strain drives expression in D-type neurons, the three strains do show differences in their distributions of the ratios of cells expressing GFP relative to mCherry (Kruskal-Wallis test, p = 1.53×10^−5^).

The regulatory elements of *unc-25* from *C*. *elegans*, *C*. *briggsae*, *C*. *brenneri*, *C*. *remanei*, and *C*. *japonica* directed consistent expression in the D-type GABAergic neurons, which constitute the majority of the endogenous expression pattern of this gene [38,39]. The same was the case for their *M*. *hapla*, *B*. *malayi*, and *T*. *spiralis* orthologs, despite the great phylogenetic distances separating these species (Figs 3A–3D and S2). Note that while the *T*. *spiralis unc-47 cis-*regulatory DNA did not drive expression in D-type neurons (Fig 2C and 2D), the *unc-25* upstream region from this same species did so (Fig 3C and 3D). Expression patterns were consistent across independently generated transgenic strains (S2 Table).

#### *mec-3*

**Fig 4:**
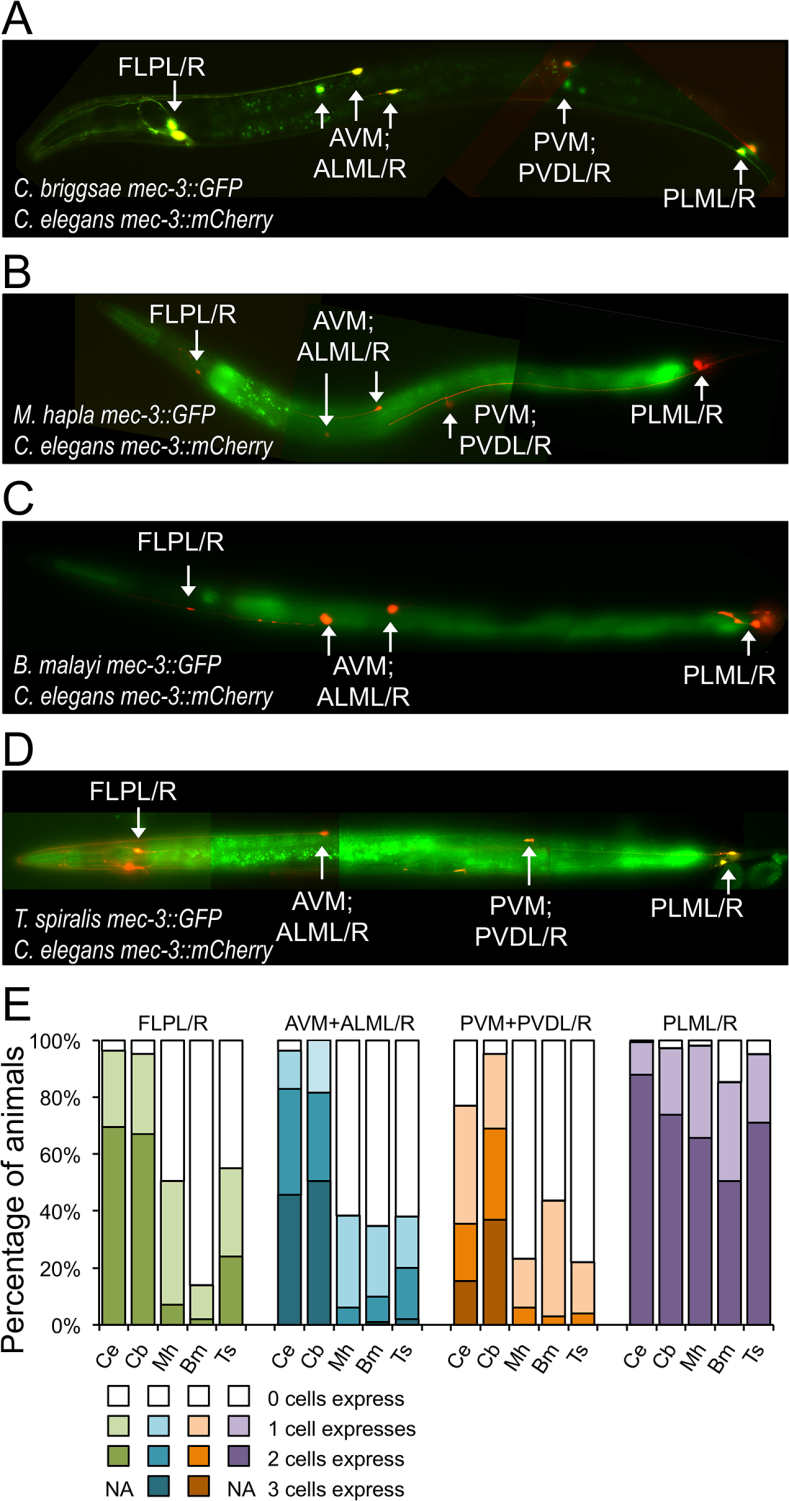
*mec-3* regulatory sequences from distantly-related nematodes drive expression in *C*. *elegans*. (A-D) *C*. *elegans mec-3* regulatory sequence drives expression of *mCherry* in all transgenic strains; (A) *C*. *briggsae*, (B) *M*. *hapla*, (C) *B*. *malayi*, (D) *T*. *spiralis mec-3* regulatory sequences drive expression of *GFP*. Animals were photographed at 400x magnification. Images are false-colored composites of single animals. Separate GFP and mCherry images are shown in S3 Fig. (E) Percentage of animals with 0 (white), 1 (light), 2 (mid-tone), or in some cases 3 (darkest shade) cells with reporter gene expression of *GFP* directed by the heterologous elements and *mCherry* directed by the *C*. *elegans* element. The data are based on counting over 100 individuals carrying each transgene pair (see Materials and Methods and S1 Table for total counts). The *C*. *elegans* data are derived from averaging the number of animals expressing mCherry across all *mec-3* carrying strains shown here (see Materials and Methods and S1 Table). A maximum of two cells for the FLPs and PLMs and three cells for AVM+ALMs and PVM+PVDs could have expression.

The DNA upstream of orthologs of *mec-3* drove expression in the six touch-receptor neurons (ALML/R, AVM, PVM, and PLML/R) and two additional, extensively branched, pairs of mechanosensory neurons, FLPL/R in the head, and PVDL/R in the posterior midbody (Fig 4); these cells constitute the endogenous gene expression pattern in *C*. *elegans* [40]. Expression was strong and consistent in all cells from both *C*. *elegans* and *C*. *briggsae cis-*regulatory elements (Fig 4A, 4E and S3). While none of the regulatory elements completely failed to drive expression in any of these cells, those from the three distantly-related nematodes directed dramatically less consistent expression in all cells but PLML/R in the tail (Fig 4E). In addition to driving expression in the mechanosensory neurons as expected, the *B*. *malayi cis* element also drove ectopic expression in several ventral cord neurons (S4 Fig). Expression patterns were consistent across independently generated transgenic strains (S2 Table).

#### *elt-2*

**Fig 5:**
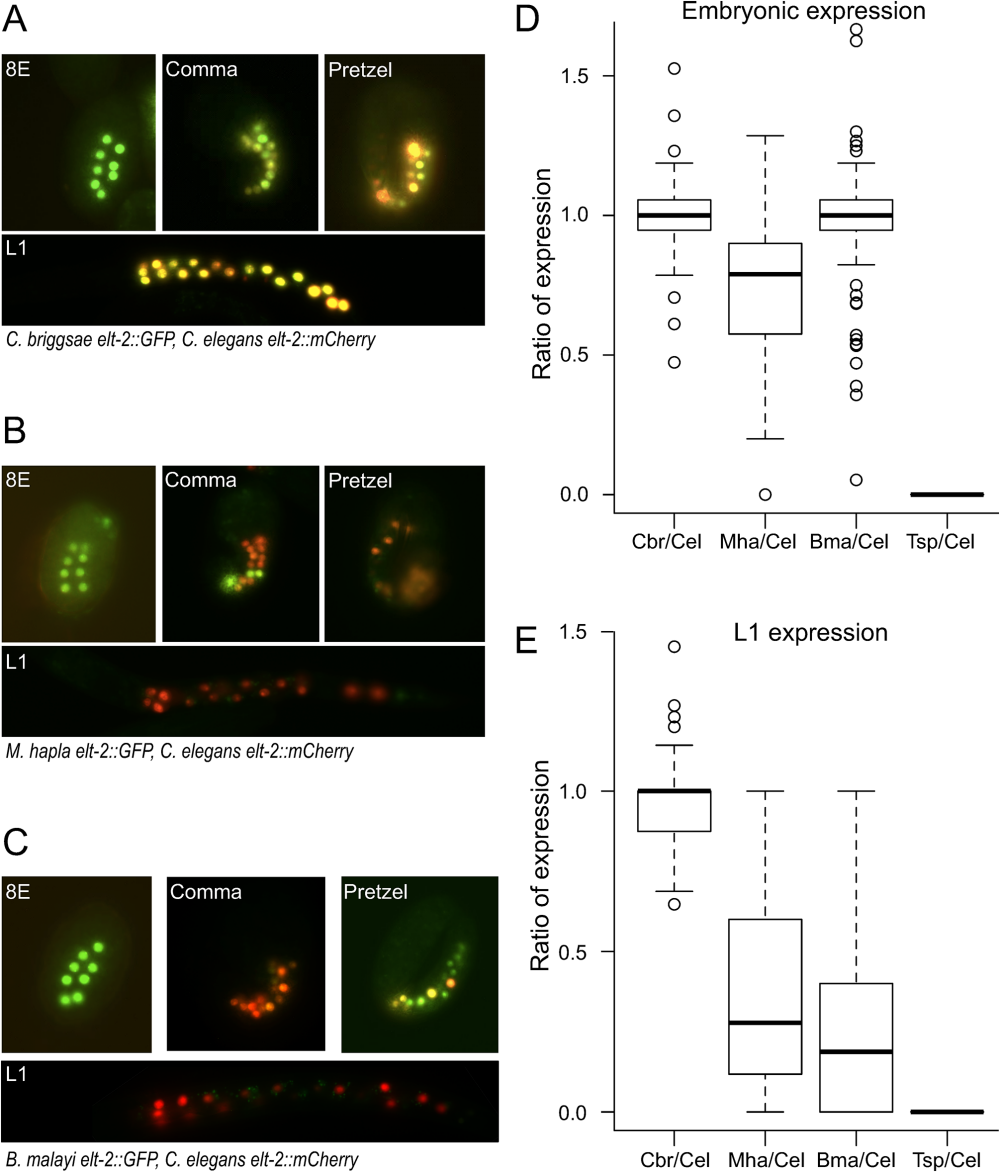
*elt-2* regulatory sequences from distantly-related nematodes drive expression in *C*. *elegans*. (A-C) *C*. *elegans elt-2* regulatory sequence drives expression of *mCherry* in all transgenic strains; (A) *C*. *briggsae*, (B) *M*. *hapla*, (C) *B*. *malayi elt-2* regulatory sequences drive expression of *GFP*. 8E, comma, pretzel and L1 refer to three characteristic embryonic stages and the first larval stage, respectively. Animals were photographed at 400x magnification. Images are false-colored composites of single animals. Separate GFP and mCherry images are shown in S5 Fig. (D) Ratios of the number of E-cell descendants in pretzel stage embryos expressing GFP/mCherry. While the Mha/Cel ratios are significantly different from the other two (Wilcoxon rank sum test, p<10^−14^), Bma/Cel and Cbr/Cel do not differ (Wilcoxon rank sum test, p = 0.99). (E) Ratios of the number of E-cell descendants in L1 larvae expressing GFP/mCherry. While Cbr/Cel was not significantly different from Bma/Cel at the pretzel stage, at the L1 stage Cbr/Cel is significantly different from both Bma/Cel and Mha/Cel (Wilcoxon rank sum test, p<2.2×10^−16^). See Materials and Methods and S1 Table for total counts.

The *C*. *elegans elt-2* gene is endogenously expressed in the endodermal cells throughout development [46], and appears to have the same expression in a Clade V nematode, *Haemonchus contortus* [45]. The upstream regions of orthologs of *elt-2* drove the expected endodermal expression that was first detectable at the 4E stage, and consistently visible at the embryonic 8E stage (Figs 5 and S5). The *elt-2* regulatory DNA of *C*. *briggsae*, *M*. *hapla*, and *B*. *malayi* all drove consistent expression at the embryonic stages (Fig 5A–5D). However, only the *C*. *elegans* and *C*. *briggsae elt-2* upstream sequences directed consistent expression in the first larval stage (Fig 5E), and beyond. The sequence upstream of *T*. *spiralis elt-2* did not drive any detectable expression in any tissue at any stage (Fig 5E). The *M*. *hapla elt-2 cis-*regulatory DNA occasionally drove expression in cells anterior to the developing gut, but this expression was not consistent and diminished in later stages earlier than gut expression (Figs 5B and S5B). Expression patterns were consistent across independently generated transgenic strains (S2 Table).

### Orthologous regulatory sequences from distantly-related nematodes have little similarity

All but one of the 12 regulatory sequences from distantly related species that we tested in *C*. *elegans* directed expression in at least a subset of the expected cells, so some degree of functional conservation is preserved even at these great phylogenetic distances. Since the putative regulatory regions from the distant relatives were selected without regard for non-coding conservation, we next examined them for sequence similarity with the *C*. *elegans* orthologs. We did not know, *a priori*, what types of sequence similarity to expect, and did not find any extended sequence conservation. For this reason, we conducted three types of sequence comparison to ascertain the extent of sequence similarity between *C*. *elegans* and each of the distantly-related nematodes.

**Fig 6:**
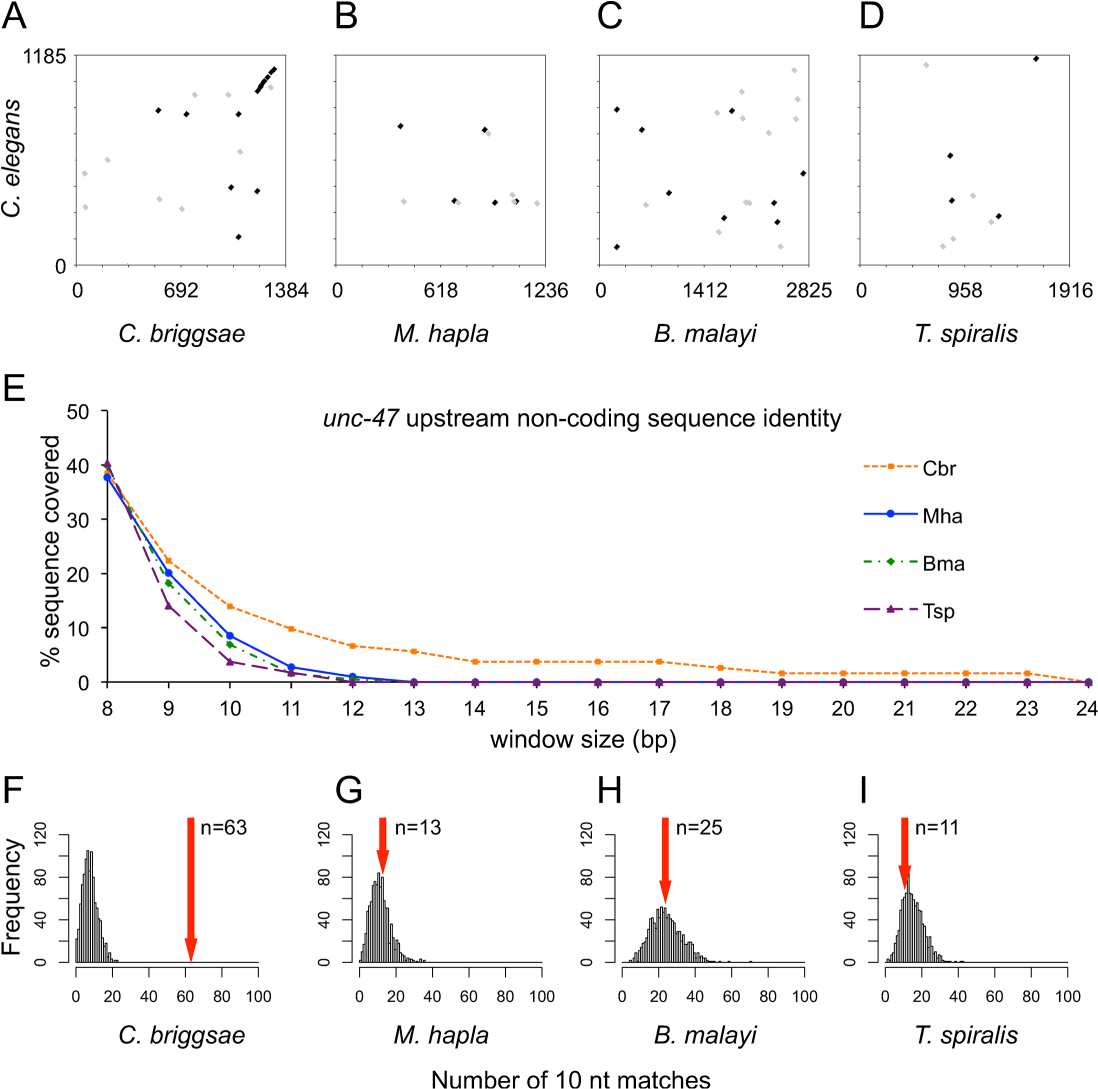
Sequence similarity of orthologous *unc-47 cis* elements. (A-D) Dotplots showing perfect sequence identity (in 10 nucleotide windows) of the *unc-47* upstream sequences in direct (black) and reverse (gray) orientations between *C*. *elegans* and (A) *C*. *briggsae*, (B) *M*. *hapla*, (C) *B*. *malayi*, (D) *T*. *spiralis*. (E) Percentage (y-axis) of *unc-47* upstream sequences composed of short blocks of sequence identity with *C*. *elegans* across various window sizes (x-axis). (F-I) Distributions of the number of perfect 10 nucleotide matches between 1000 replicates of reshuffled *C*. *elegans unc-47* upstream sequence and the upstream *unc-47* sequences of (F) *C*. *briggsae*, (G) *M*. *hapla*, (H) *B*. *malayi*, (I) *T*. *spiralis*. Trinucleotide frequencies of the original *C*. *elegans unc-47* upstream sequence were retained. Red arrows indicate the actual number of 10 nucleotide matches between *C*. *elegans* and each ortholog. Only *C*. *briggsae* has more than would be expected by chance (see Results). Note that *B*. *malayi* sequence is approximately twice as long as that of *C*. *elegans*, *C*. *briggsae*, and *M*. *hapla*.

First, we created dotplots, which depict the positions of nucleotide strings of a certain length that are shared by the *C*. *elegans unc-47* sequence and a sequence from another nematode (10 bp examples shown in Fig 6A–6D). Only the *C*. *briggsae cis* element displayed substantial evidence of sequence *conservation*, represented by collinear blocks of sequence with conserved spacing upstream of the translation start site (upper right diagonal, Fig 6A). Not only do the distantly-related nematodes lack any such collinear blocks of sequence (evidence of conservation), they lack much in with way of sequence *similarity* as well, with only a few scattered motifs found in both the *C*. *elegans unc-47* upstream region and those upstream regions from *M*. *hapla*, *B*. *malayi*, and *T*. *spiralis* (Fig 6B–6D).

We looked more closely at the few 10 bp motifs in each of the divergent sequences that are shared with the *C*. *elegans cis* element (Fig 6B–6D). Since the functional units of *cis*-regulatory elements are thought to be short binding sites, we next hypothesized that the divergent *cis* elements might be enriched for such short, shared motifs. We tested this in two ways. First, we broke the sequences down into their component k-mers, and asked what percentage of the total sequence length was made up of k-mers shared with the *C*. *elegans* sequence. For example, by definition, 100% of the *M*. *hapla unc-47 cis* element is made up of 1-mers (A, T, G, or C) that are also found in the *C*. *elegans unc-47 cis* element. Approximately 40% of the examined *cis* elements of *C*. *briggsae* and the other 3 nematodes are made up of 8-mers that are also found in the *C*. *elegans* sequence (Fig 6E), suggesting that window sizes shorter than 9 nucleotides are not likely to be informative for this comparison. For 9-mers, slight differences in the proportion of shared sequence can be detected among species; at window sizes of 10–12 nucleotides, the difference between *C*. *briggsae* and the distantly-related nematodes becomes apparent (Fig 6E). Note that the *B*. *malayi unc-47 cis* element, while it functions remarkably better than the *T*. *spiralis* ortholog (Fig 2), is not substantially more similar in sequence to the *C*. *elegans* regulatory element. None of the three distantly-related nematodes had any identical sequence blocks longer than 12 nucleotides, and blocks longer than 10 nucleotides were primarily low-complexity polynucleotide sequences (S6 Fig), while *C*. *briggsae* had identical sequences of up to 23 nucleotides in length (Fig 6E). Alignments showing all of the identical sequence matches in the *unc-47* upstream regions that are 9 nucleotides or longer can be found in S6 Fig. These identical blocks are not enriched proximal to the start of the coding sequence. Similar levels of conservation were found for *unc-25*, *mec-3*, and *elt-2* as well (S7–S9 Figs).

The next method that we used to test whether the orthologous *cis* elements were enriched for short motifs shared with the *C*. *elegans unc-47* upstream region compared the number of shared motifs detected with the number that might be expected by chance. Here, “chance” refers to a random reordering of the *C*. *elegans* sequence that preserves nucleotide, dinucleotide, or trinucleotide frequencies. For each of the four genes, we reshuffled the *C*. *elegans* sequence 1000 times. The *cis* elements from *C*. *briggsae*, *M*. *hapla*, *B*. *malayi*, and *T*. *spiralis* were compared to each of the 1000 reshuffled *C*. *elegans* sequences, and we calculated the numbers of nucleotide blocks (length 8–12) that were identical between each reshuffled *C*. *elegans cis* element and each of the orthologs. This provided empirically derived distributions of sequence identity that could be expected solely as a result of basic nucleotide composition properties. The results for tests of 10 nucleotide blocks are shown in Fig 6F–6I. For the 1000 comparisons between the reshuffled *C*. *elegans* sequences and the other nematode’s upstream *unc-47* sequence, the number of identical motifs was plotted (Fig 6F–6I). The number of motifs form distributions centered between about 10–20 motifs per reshuffled sequence, depending on the length of the ortholog. Comparing the actual number of conserved blocks of various lengths between *C*. *elegans cis* elements and their orthologs revealed that only *C*. *briggsae* had more sequence identity than our “chance” rearrangements, with 63 identical 10-mers (Fig 6F). The other three distantly-related nematodes’ sequences had no more similarity than expected by chance, with numbers of shared 10-mers that fell close to the means of the distributions (Fig 6G–6I). The same was true for the upstream noncoding sequences of *unc-25*, *mec-3*, and *elt-2* (S10 Fig).

### Orthologous *cis* elements share short putative regulatory motifs

Comparisons of noncoding sequence identity did not reveal any substantially conserved regions likely to be responsible for the functional conservation of orthologous *cis* elements. And yet, 11 of the 12 *cis*-regulatory elements from deeply diverged nematodes drove gene expression in *C*. *elegans* that recapitulated at least some of the expected endogenous expression pattern. We therefore searched the orthologous sequences for motifs known to be functionally important in the *C*. *elegans* sequences. Expression of *unc-47* is regulated by direct binding of UNC-30 [47] to TAATCC sites. Mutations to this motif abolish expression in the D-type neurons [47]. Perhaps functional conservation of the *unc-47 cis* elements from distantly-related nematodes is due to the presence of this and other short sequences below the level of detection in our naive sequence comparison.

**Fig 7:**
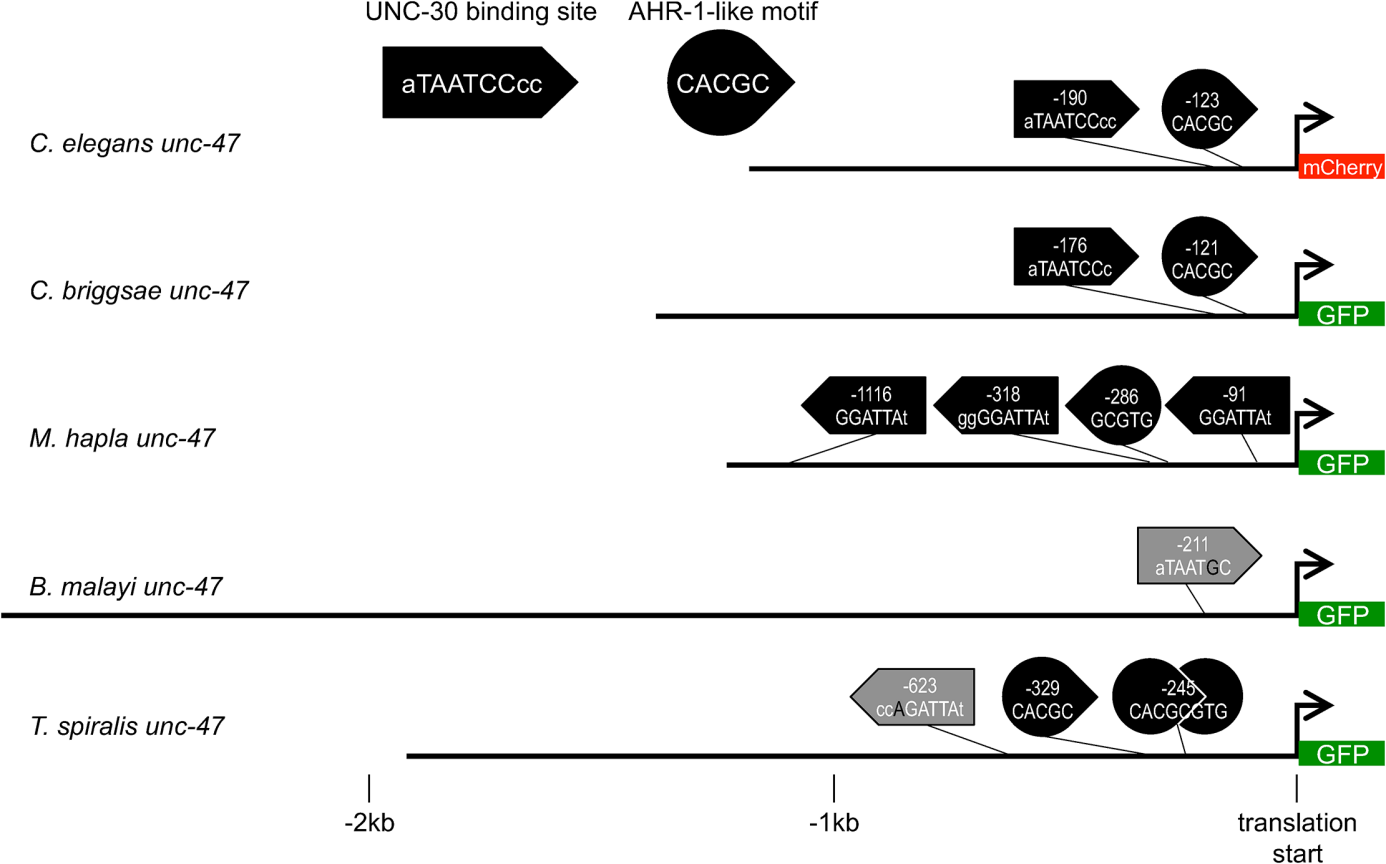
Matches to motifs responsible for the *C*. *elegans unc-47* gene expression pattern can be found in orthologous sequences. Cartoons depicting sequences upstream of the translation start sites (bent arrows) of *unc-47* orthologs. These were fused to *mCherry* (*C*. *elegans*) or *GFP* (all others). UNC-30 (box arrow) and AHR-1-like (rounded arrow) motifs are shown, in uppercase letters; conserved flanking nucleotides shown in lowercase. Locations of motifs relative to the endogenous translation start sites are indicated. Where the best match has a single mismatch with the consensus sequence, it is shown by a gray shaded arrow with the mismatched nucleotide shown in black.

Searching for the TAATCC site revealed a perfect match, including one flanking base pair on either side in *C*. *briggsae*, with similar spacing from the translational start site (Fig 7). The noncoding sequence upstream of *M*. *hapla unc-47* has three instances of this motif, all on the reverse strand, with additional identical nucleotides flanking the core site (Fig 7). The upstream sequences from *B*. *malayi* and *T*. *spiralis* lack perfect matches to this consensus, but do have 5/6 bp core matches with some additional flanking identity (Fig 7). Either these close matches are divergent *cis*-regulatory sites, hinting at evolved differences in TF-TFBS recognition, or else there is more to the control of expression in D-type neurons than we have recognized in *C*. *elegans* thus far.

The *C*. *elegans* UNC-30 binding site controls expression in D-type neurons, but not in DVB in the tail or AVL, RIS, or the RMEs in the head [47]. One site that contributes to expression in DVB, RIS, and AVL is the AHR-1-like motif [37]. This motif has the sequence CACGC and is conserved in sequence and position between *C*. *elegans*, *C*. *briggsae*, *C*. *brenneri*, and *C*. *remanei* [37]. A match to this motif is found on the reverse strand of the *M*. *hapla unc-47 cis* element (Fig 7). A palindromic sequence CACGCGTG, that is, two overlapping AHR-1-like motifs on opposite strands, along with an additional single instance of this motif, are present upstream of the *T*. *spiralis unc-47* gene (Fig 7).

Similar motif-matching analyses were carried out for the other three sets of orthologous *cis*-regulatory elements. Matches to motifs known to be necessary for function in *C*. *elegans* were identified in almost all tested orthologs from distantly-related nematodes (S1 Text; S11–S13 Figs). However, the occurrence of even multiple instances of motifs corresponding to transcription factor binding sites should not be construed as evidence of conservation. First, these motifs are not found any more frequently than in randomly reshuffled *C*. *elegans* sequences. We explicitly estimated the probability of finding these motifs in the randomly reshuffled *C*. *elegans* sequences. The probability of finding TAATCC (the UNC-30 binding site) in sequences preserving the single-nucleotide composition of the *C*. *elegans cis* element was 0.558, conserving dinucleotides it was 0.378, and trinucleotides it was 0.566. The probabilities of finding CACGC (the AHR-1-like motif) in these same sequences were 0.632, 0.606, 0.674, respectively. Second, these motifs were routinely found in the *cis* elements of the other genes we examined (S3 Table). Third, these motifs are often found on the opposite strand, suggesting that, while individual motifs are born and die, this sequence turnover maintains at least one instance of the motif in each of the orthologous regulatory elements. Therefore, identical motifs are not, strictly speaking, conserved.

### Motif similarity identifies functional sites in diverged orthologs

It is suggestive that the *unc-47* regulatory sequence from a distantly-related nematode that retains the best function in D-type neurons, that of *M*. *hapla*, has the best match to the UNC-30 binding site. Similarly, the regulatory sequence with the best function in DVB—*T*. *spiralis unc-47—*has the best matches to the AHR-1-like motif. We therefore tested the contribution of these motifs to functional conservation.

**Fig 8:**
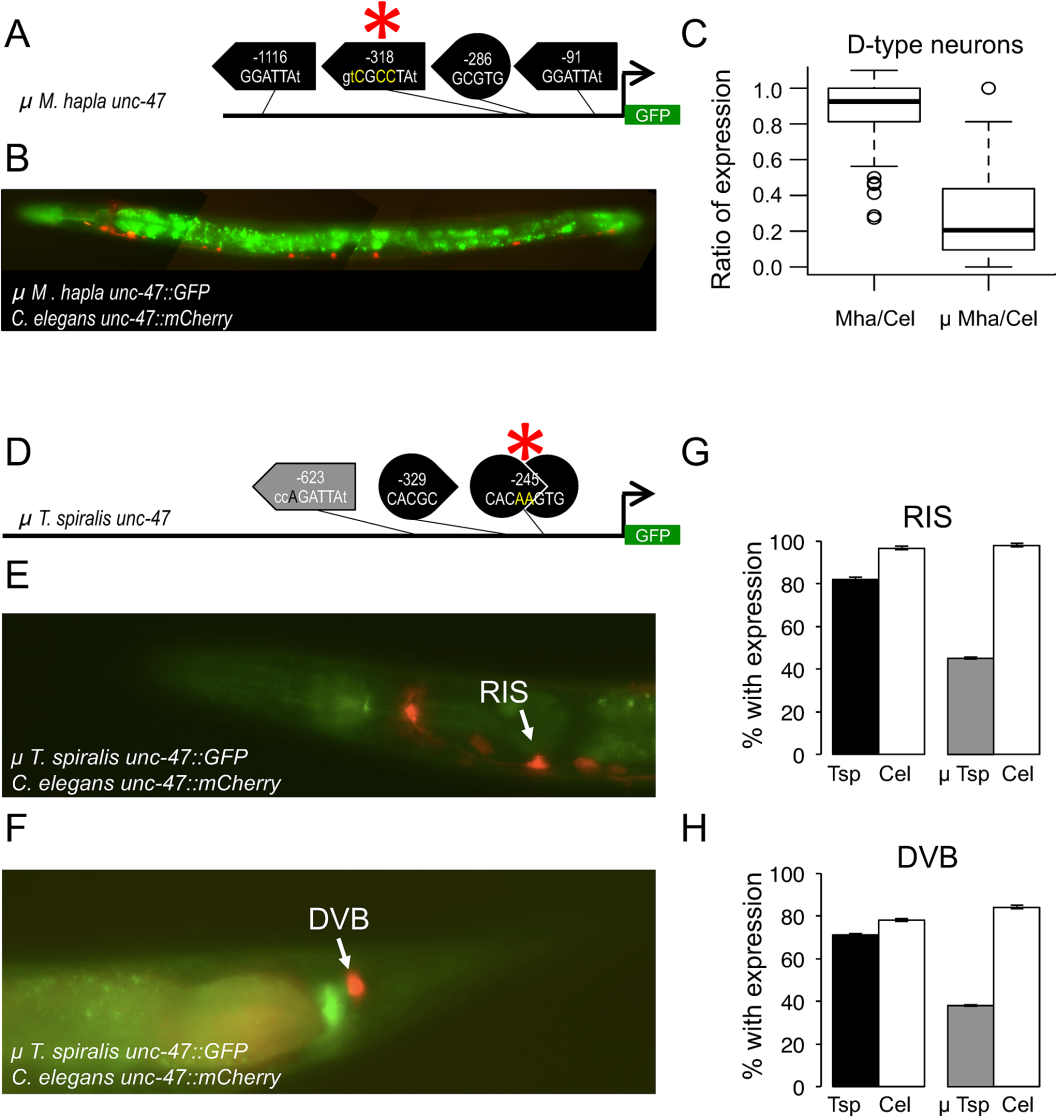
Mutations in putative transcription factor binding sites disrupt *cis*-regulatory functions. (A) Cartoon depicting the mutations made to the *M*. *hapla unc-47 cis* sequence, with the affected motif designated by the asterisk and the sequence changes shown in yellow. (B) Mutant *M*. *hapla unc-47 cis* element drives expression of *GFP*. *C*. *elegans unc-47* regulatory sequence drives expression of *mCherry*. Animals were photographed at 400x magnification. Images are false-colored composites of single animals. Separate GFP and mCherry images are shown in S14 Fig. (C) Ratios of the number of D-type neurons expressing GFP/mCherry for wild-type (Mha/Cel) and mutant (μ Mha/Cel) *cis-*regulatory elements. Each ratio is based on counting over 100 individuals carrying each transgene pair (see Materials and Methods and S1 Table for total counts). The two are significantly different (Kruskal-Wallis test, p < 2.2 ×10^−16^). (D) Cartoon depicting the mutations made to the *T*. *spiralis unc-47 cis* sequence, with the affected motif designated by the asterisk and the sequence changes shown in yellow. Mutant *T*. *spiralis unc-47 cis* element drives expression of *GFP* in RIS (E) and DVB (F). Wild-type *C*. *elegans unc-47* regulatory sequence drives expression of *mCherry*. Animals were photographed at 400x magnification. Images are false-colored composites of single animals. Separate GFP and mCherry images are shown in S14 Fig. Percentage of individuals with expression in RIS (G) and DVB (H) from the *T*. *spiralis* wild-type (black) and mutant (μ Tsp, gray) *cis*-regulatory elements (see Materials and Methods and S1 Table for total counts). The mutated *T*. *spiralis unc-47 cis* element drives substantially less consistent expression than the wild-type, in RIS (Fisher’s Exact Test, p = 4.564×10^−6^) and DVB (Fisher’s Exact Test, p = 2.536× 10^−6^). Expression of *C*. *elegans unc-47* regulatory sequence is shown in white; this control is not significantly different for either RIS (p = 0.7056) or DVB (p = 0.3775). Error bars represent 95% confidence interval for the proportion expressing.

We introduced mutations into an UNC-30 binding motif in the *M*. *hapla unc-47 cis* element. This motif was selected because it shares the longest similarity in the flanking sequences with the UNC-30 binding site of the *C*. *elegans unc-47 cis* element (Fig 8A). The mutant *M*. *hapla unc-47* sequence directed less consistent expression in the D-type neurons than its wild-type counterpart (Fig 8B, 8C and S14A), suggesting that this UNC-30 motif contributes to control of gene expression in the D-type neurons.

Next, we introduced mutations into the palindromic double AHR-1-like motif of the *T*. *spiralis unc-47* element, eliminating the consensus sequence on both strands (Fig 8D). This resulted in a substantial decrease in the fraction of animals expressing the transgene in RIS and DVB (Figs 8E–8H and S14B). This suggests that the palindromic AHR-1-like motif upstream of the *T*. *spiralis unc-47* gene is partially responsible for expression in DVB and RIS, just as the AHR-l-like motif is in *C*. *elegans* [37].

Neither mutation eliminated expression in the affected cells entirely, implying that these sites contribute to but are not strictly essential for expression. This could be due to the redundancy of these binding sites in both *cis* elements (Fig 7). As another possible explanation, consider the case of the *B*. *malayi unc-47* element that lacks good matches for either the UNC-30 or the AHR-1-like motifs, and yet is reasonably well expressed in both the D-type neurons and in DVB. It is possible that some orthologous *cis* elements retain functional conservation via sequences that can be recognized by *C*. *elegans* transcription factors, but that we currently cannot recognize as functional.

## Discussion

We investigated *cis*-regulatory function in an explicitly evolutionary framework. The extent of divergence between the species involved in this study ranged from that of congenerics (*C*. *elegans* and *C*. *briggsae*) to the deepest in the phylum Nematoda (*C*. *elegans* and *T*. *spiralis*). This allowed us to test how regulatory information breaks down over time. Transgenic experiments were conducted in the “common garden” of *C*. *elegans* to control for the effects of *trans*-regulatory divergence and to focus comparisons on the *cis* elements (see Potential Caveats in Materials and Methods). We selected the putative regulatory regions without regard for non-coding sequence similarity, which then permitted us to comment on the relationship between functional and sequence conservation. We used a standard methodology to look at the *cis* elements of four genes, allowing us to make four generalizations.

First, despite the vast spans of evolutionary time that we sampled—the most distantly-related species diverged perhaps as long as 400 million years ago [24]—the majority of the *cis-*regulatory elements exhibited appreciably conserved gene regulatory function in *C*. *elegans* (Table 1). Two reasons compelled us to focus explicitly on the *conserved*, rather than divergent, aspects of expression. First, at such great phylogenetic distances, any conservation might be less expected than divergence. Second, for technical reasons, we can only know the endogenous function of the *C*. *elegans* regulatory elements, not the patterns driven by the divergent *cis* elements in their native species (see Potential Caveats in Materials and Methods). Our findings are consistent with previous reports of functional conservation of *cis*-regulatory elements between distantly-related members of the same phylum, most extensively tested in arthropods and chordates [48,49]. Although there have been a number of reports of functional conservation of *cis* elements between different phyla [50–56], this is not true for *cis* elements of all genes tested [39,57]. It is possible that the evolutionary dynamics of regulatory elements may be sufficiently idiosyncratic to preclude general conclusions about the “outer limits” of *cis*-regulatory conservation.

**Table 1:** Summary of gene expression driven by parasitic nematode *cis* elements relative to *C*. *elegans* expression.

	*M*. *hapla*	*B*. *malayi*	*T*. *spiralis*
*unc-47*	Weak, missing DVB cell consistently	Weak, patchy	Absent in 24/26 cells consistently, expressed in 2/26 consistently, ectopic expression in non-neuronal cell types
*unc-25*	Correct but weak	Correct but weak	Correct but weak
*mec-3*	Inconsistent in anterior cells, but very consistent in PLMs	Inconsistent in anterior cells, but very consistent in PLMs; ectopic in VC neurons	Inconsistent in anterior cells, but very consistent in PLMs
*elt-2*	Indistinguishable until hatching, reduced in larvae, absent in adults	Indistinguishable until hatching, reduced in larvae, absent in adults	No expression

Second, in most cases *cis*-regulatory elements from more distant relatives have retained less function than elements from closer relatives. However, there are notable exceptions and, importantly, the pattern of functional divergence that we observed reflects modular organization of *cis-*regulatory elements—separable elements control different aspects of expression [58–60]. Due to modularity of *cis* elements, evolution can “tinker” with some functions while avoiding pleiotropic effects on others [61]. In *C*. *elegans*, expression of *unc-47* is controlled by different mechanisms in D-type neurons and DVB, RIS, and AVL [37,47]. Accordingly, we see that whereas the *T*. *spiralis unc-47* element is not expressed in D-type neurons, it functions relatively well in DVB and RIS (Figs 2 and S1). In contrast, the *M*. *hapla unc-47* element is expressed well in the D-type neurons, but not in DVB (Fig 2). Similarly, the *elt-2* elements from *M*. *hapla* and *B*. *malayi* are expressed reasonably well during embryogenesis, but not in later stages (Fig 5). We consider this good evidence for separate regulation of pattern, timing, and levels of expression, as well as substantiating evidence that the weak expression of some of these regulatory elements is due to genuine divergence of regulatory information rather than experimental artifacts of weak transgene expression. We conclude that modular organization of *cis* elements manifests in different rates of divergence for different aspects of expression patterns [62] and may be quite common [13]. Mechanisms controlling spatial, temporal, and levels of expression may be particularly prone to different rates of divergence (e.g. [45,63]).

Third, despite their substantially conserved functions, the regulatory elements of all species but *C*. *briggsae* have not retained more sequence similarity than would be expected by chance. This finding is consistent with previous reports that suggested that conservation of *cis*-regulatory function does not, strictly speaking, require extended sequence conservation [64–70]. Since different types of regulatory elements evolve under different constraints [36], relying on sequence conservation to find *cis*-regulatory elements might bias discovery to only particular types of elements with highly constrained sequences [71]. Additionally, because sequences of different elements evolve at different rates [38], it is not *a priori* clear how distant the species to be compared should be to discover *cis* elements of different types. Even when some short stretches of identical nucleotides are discovered between distantly-related orthologous *cis* elements, this should not be taken as evidence of conservation. This is because many short matches will always be found by chance, particularly in regions with biased nucleotide composition. For instance, the co-occurrence of the UNC-30 and AHR-1-like motifs upstream of *unc-47* orthologs (Fig 7) is more plausibly explained by a birth-and-death process rather than strict conservation, considering that these motifs are found on opposite strands of DNA in different species.

Fourth, despite the lack of extended sequence conservation, for all four genes we could readily identify motifs corresponding to transcription factor binding sites previously identified as functionally important for regulation of *C*. *elegans* orthologs. The motifs that we tested contributed to gene regulation of the orthologous *cis* elements, implying that gene regulatory output can be conserved, even among distantly-related organisms, as long as key gene regulatory connections—“kernels” [10,72] or “input-output devices” [73]—are maintained. This further reinforces the view that when developmental programs evolve, the regulatory “toolkit” controlling major patterning and cell-type specification programs remains relatively static [6]. Of course, the mere presence of these short motifs is not likely to be sufficient to explain regulatory output. For instance, we can find chance matches to GATA motifs important for *elt-2* expression in many of the other sequences we tested, which do not drive expression in the intestinal precursor cells. Similarly, we can find matches to the AHR-1-like motif (that regulates *unc-47* expression) in the *elt-2 cis* elements of *C*. *elegans*, *C*. *briggsae*, and *T*. *spiralis*, none of which drive expression in DVB, RIS, or AVL.

In this study we aimed to understand how the patterns of divergence of gene regulatory mechanisms between closely related species scale up over long evolutionary times. Models have predicted [74] that regulatory control can be shifted from one site to another within a *cis*-regulatory sequence; if these sites arise somewhat stochastically, longer wait times increase the likelihood of new sites originating and being optimized. These new sites could diminish the strength of purifying selection acting on ancestral motifs [75]. On shorter evolutionary time scales, new motifs do not have the time to arise, so function relies on conservation of existing sites [76]. As the same process plays out over different timespans, *cis*-regulatory conservation remains common among close relatives, but is mostly absent among more distantly-related species.

Naturally, the rates of divergence and motif turnover are different for different genes. An important factor determining the rate of evolution could be the organization of a *cis* element, whether it is flexible [77,78] or constrained [79], a billboard or an enhanceosome [80,81]. Modeling suggests that some enhancer sequences are inherently more prone to higher rates of turnover than others [74]. Better understanding of the structure of *cis*-regulatory elements may provide clues to their evolution [70,82,83].

Practically, our results advocate the use of *C*. *elegans* as a convenient and reliable experimental system for testing the functions of putative regulatory elements from nematode species, many of them parasites of major economic and medical significance, that are not amenable to transgenic studies [45]. Furthermore, the fact that *C*. *elegans* has been a genetic model system for decades means that the wealth of information about gene regulation in this species could be leveraged into hypothesis-driven investigation of non-model organisms.

As discussed above, functionally conserved sequences can retain no more sequence conservation than would be expected by chance. Indeed, motifs that mediate functional conservation, namely transcription factor binding sites, are short enough that they would be likely to be found by chance in sequences of the lengths of these *cis* elements. By the measures of sequence conservation we applied, including alignment-free methods, *M*. *hapla* does not have appreciably greater sequence similarity to *C*. *elegans* than does *T*. *spiralis*. Nevertheless, *M*. *hapla cis* elements of all four tested genes drive more consistent and correct expression in transgenic *C*. *elegans* than elements from *T*. *spiralis* do. This means that some sequence properties were retained to a greater extent by the more closely related species. Identification of these properties would lead to a better understanding of function and evolution of gene regulatory elements.

## Materials and Methods

### Cloning of *cis*-regulatory elements

Orthologous genes from *C*. *briggsae*, *M*. *hapla*, *B*. *malayi*, and *T*. *spiralis* were identified as best tblastn/blastx matches with the *C*. *elegans* protein sequence. For *C*. *briggsae*, *B*. *malayi*, and *T*. *spiralis*, the genome browser on Wormbase was used. For *M*. *hapla*, the genome browser at www.hapla.org was used. Forward primers were designed proximal to the next upstream gene, or failing that the 5’-most part of the contig on which the orthologous coding sequence was found. Reverse primers were selected to make in-frame translational fusions with *GFP* in the 5’-most part of the gene with protein coding sequence similarity with *C*. *elegans*. The only cases in which this was not possible were *B*. *malayi* and *T*. *spiralis elt-2*, in which protein-coding conservation started deep in the protein-coding sequence, and the fusions were generated in the first exon. A previous study of *elt-2* from a parasitic nematode, the less divergent *Haemonchus contortus* [84], found that despite protein sequence divergence from *C*. *elegans*, the *H*. *contortus* protein retained function when expressed transgenically in *C*. *elegans* by a *C*. *elegans* heat shock promoter, so this increases our confidence that these can be *elt-2* orthologs despite coding sequence divergence. In all cases, the start codon of the ortholog was included in the fusion. To generate reporter transgenes, upstream non-coding sequences were PCR amplified from genomic DNA and cloned upstream of *GFP* into the Fire vector pPD95.75, or upstream of mCherry (for *C*. *elegans* genes), which was inserted in place of *GFP* in a modified vector pPD95.75 [85]. *elt-2* transgenes carried a nuclear localization signal upstream of *GFP* or *mCherry*. Prior to injection, all transgenes were sequenced to ensure accuracy.

### Transgenes and strains

We injected a mixture (5 ng/μL (for *C*. *briggsae*; 10 ng/μL for the other species) promoter::*GFP* plasmid, 5 or 10 ng/μL promoter::*mCherry* plasmid, 5 ng/μL *pha-1* rescue transgene, 100 ng/μL salmon sperm DNA) into temperature-sensitive *C*. *elegans pha-1*(*e2123*) strain [86]. Transformants were selected at 25°C. Multiple strains were examined for each transgenic construct. Statistical analyses of consistency of expression patterns between strains and individuals are presented in S2 Table, since extrachromosomal transgenes are known to have more variable expression than integrated transgenes. Our previous reports [36,37] thoroughly addressed the similarity of expression driven by transgenes of different types—extrachromosomal, multicopy integrated, and single-copy integrated. We found that while the strength of the signal increases with multiple copies, and variability increases with extrachromosomal transgenes, the patterns generated by these different methods are consistent.

The structures of extrachromosomal transgene arrays are generally not known. Although there is a possibility of cross-talk between promoters from different species if they land close enough when the DNA is concatenated, we mitigate against this by including an excess of salmon sperm DNA and vector sequence to create distance between the promoters and reduce the repetitiveness of the arrays. We measure expression in multiple independent strains. We also tested several of the highly divergent promoters alone, without a coexpressed *C*. *elegans*-DNA-driven reporter (S15 Fig). Without the coexpressed mCherry marker, cells were more difficult to identify, so counts were not attempted for these strains, but expression was observed in the same subsets of cells that it was observed in coexpressing lines.

The coexpressing strains also allowed us to control for the mosaicism inherent in extrachromosomal transgenes. Since the transgenes are concatenated, *mCherry* and *GFP* are inherited together by cells, and if array loss or silencing causes the loss of expression of one marker, the other will also disappear. This is why, for most of our quantification, we describe expression as the ratio of mCherry (control) positive cells that also express GFP (see Figs 2–5 and 7).

### Mutagenesis of *unc-47 cis* elements

We tested the functions of motifs corresponding to consensus sequences of binding sites of UNC-30 [47] (TAATCC) and AHR-1-like [37] (CACGC). Motifs were identified using the ConsensusSequence feature on the GeneGrokker web server (https://genegrokker.biology.uiowa.edu). Of the several UNC-30 motifs in the *M*. *hapla unc-47* element, we selected for mutagenesis the longest extended match to the *C*. *elegans* sequence: aTAATCCcc (reverse complement, since the motif is found on the (-) strand). This motif was mutagenized to aTA**GG**C**Ga**c (changes highlighted). Of the several matches to the AHR-1-like motif in the *T*. *spiralis unc-47* element, the motif selected for mutagenesis was a palindromic sequence (CACGCGTG), which matches two overlapping instances of the AHR-1-like motif (one on each strand). This sequence was mutagenized to CACAAGTG, changing the CACGC sequence on the (+) strand to CAC**AA** and on the (-) strand to CAC**TT**.

All mutations were introduced by PCR with overlapping, opposite-facing primers carrying the mutant sequence. Primers were used to amplify plasmid DNA carrying the wild-type sequence. Following PCR, the reaction was digested with the methylation sensitive restriction enzyme *Dpn*I to selectively digest the wild-type plasmid template. A second PCR reaction was performed, amplifying the mutagenized *cis* element and some flanking vector sequence. This PCR product was purified and digested for directional cloning back into the expression vector. Mutations were verified by sequencing before microinjection.

### Microscopy

Mixed-stage populations of *C*. *elegans* carrying transgenes were grown with abundant food. Worms of appropriate stages were selected. These were immobilized on agar slides with 10 mM sodium azide in M9 buffer. The slides were examined on a Leica DM5000B compound microscope under 400-fold magnification, except in S4 and S15 Figs, which include micrographs taken at 1000-fold magnification (as labeled). Exposure times varied as necessary for each transgene. Each photograph showing worms in figures is composed of several images of the same individual capturing anterior, middle, and posterior sections, as well as shallow and deep focus. False-colored composite images were generated with QCapturePro. Brightness, contrast, and scaling of images were adjusted where necessary in final display items.

The stronger background visible in the GFP images relative to their mCherry counterparts may have several explanations. First, GFP has higher background relative to mCherry, and the autofluorescence of the gut is detectable with GFP filters. Second, longer exposure times were necessary to capture expression of the more weakly expressing exogenous *cis-*regulatory elements. Finally, GFP fluorescence in the gut is a known site of off-target expression [38]. Worms were also injected with a subset of the *GFP* transgenes carrying the other nematode’s *cis* elements alone (without a *C*. *elegans mCherry* control), and results were consistent (S1 and S2 Tables, S15 Fig).

### Cell counting

Young adult individuals were examined for gene expression, except for *elt-2*, in which case pretzel stage embryos and L1 larvae were counted. Worms without any visible fluorescence were assumed to have lost the transgene and were ignored. Presence of mCherry was a precondition for the worm to be counted, but without regard for the strength or completeness of the mCherry expression pattern.

### Sequence analysis

Motifs matching between *C*. *elegans* and each orthologous *cis* element (identified by the Mirror tool on the GeneGrokker web server https://genegrokker.biology.uiowa.edu) were mapped back to the orthologous sequence, and the total amount of the sequence covered by blocks of conservation of different sizes is plotted in Fig 6A.

Empirical p-values for the sequence similarity of the *C*. *elegans* elements to their orthologs were calculated by generating 1000 reshuffled replicates of the *C*. *elegans* sequence. Replicates were generated using single, di-, and tri-nucleotide sampling from the *C*. *elegans* sequence. Each replicate was compared to each ortholog and scored for similarity in windows of different sizes. The distributions of these similarity scores were plotted (Fig 6F–6I). The actual number of observed motif matches between the *C*. *elegans* sequence and its relevant orthologs were indicated on those distributions. The reported p-value is equal to the number of shuffled replicates that had more motif matches than the actual number, divided by 1000. Only *C*. *briggsae* had more similar motifs than would be expected by chance.

### Potential caveats

We used multicopy extrachromosomal transgenes, which could have made the detected levels of expression higher and less consistent than what would have been produced by single-copy transgenes. In previous work [36,37] we did determine that, at least in the case of *unc-47* from *C*. *elegans* and *C*. *briggsae*, the nature of the transgene (multi- vs. single-copy, extrachromosomal vs. integrated) did not change the pattern, but rather the amount and consistency of expression. If the same principle holds for the genes examined here, the conserved patterns we detected represent the cell types where the foreign *cis* elements are truly active in *C*. *elegans*, but the expression levels could be overestimated. The fact that in most instances only subsets of the overall pattern were conserved suggests that artificially higher expression levels were not solely responsible for the conserved expression patterns we detected.

Any apparent divergence—i.e. incongruence between the pattern driven by the *C*. *elegans cis* element and its orthologs—could be due to *cis*-regulatory changes (in the function of the donor element), *trans*-regulatory changes (in the function of transcription factor(s) in *C*. *elegans*), or due to the experimental combination of the two. In addition, endogenous expression patterns may have diverged between *C*. *elegans* and other species. For technical reasons, it is difficult to determine endogenous patterns of gene expression in divergent parasitic nematodes used in this study. It is even more difficult to generate transgenic animals in these species. These technical limitations make it essentially impossible to assess divergence in endogenous expression patterns or to disentangle their causes (that is, *cis* vs. *trans* changes). For these reasons, we focused on enumerating *similarities*, rather than *differences* in expression. Our tests actually underestimate the extent of regulatory conservation, because a failure of a *cis* element from a distant nematode when tested in *C*. *elegans* may reflect a genuine divergence in *cis*-regulation in that species that was compensated in *trans*, therefore maintaining the same overall expression pattern.

## Supporting Information

S1 Fig Expression patterns directed by diverse *unc-47* regulatory sequences in *C*. *elegans*. (A-C) *C*. *elegans unc-47* regulatory sequence drives expression of *mCherry* in all transgenic strains; (A) *M*. *hapla*, (B) *B*. *malayi*, (C) *T*. *spiralis unc-47* regulatory sequences drive expression of *GFP*. (D) *T*. *spiralis unc-47* element drives expression of *GFP* in RIS and DVB. Animals photographed at 400x magnification. Images are mosaics of single animals.
(PDF)

S2 Fig Expression patterns directed by diverse unc-25 regulatory sequences in C. elegans. (A-C) *C*. *elegans unc-25* regulatory sequence drives expression of *mCherry* in all transgenic strains; (A) *M*. *hapla*, (B) *B*. *malayi*, (C) *T*. *spiralis unc-25* regulatory sequences drive expression of *GFP*. Animals photographed at 400x magnification. Images are mosaics of single animals.
(PDF)

S3 Fig Expression patterns directed by diverse *mec-3* regulatory sequences in *C*. *elegans*. (A-D) *C*. *elegans mec3* regulatory sequence drives expression of *mCherry* in all transgenic strains; (A) *C*. *briggsae*, (B) *M*. *hapla*, (C) *B*. *malayi*, (D) *T*. *spiralis mec-3* regulatory sequences drive expression of *GFP*. Animals photographed at 400x magnification. Images are mosaics of single animals.
(PDF)

S4 Fig *B*. *malayi mec-3* regulatory sequence drives expression in ventral cord neurons. (A) *B*. *malayi mec-3* regulatory sequence drives expression of *GFP*. Animal photographed at 400x magnification, ventral cord at bottom. Image is a mosaic of single animals. (B) 1000x magnification of animal with ventral side up. (C) 1000x magnification of animal with ventral side up, vulva at center.</**SI_Caption>**
(PDF)

S5 Fig Expression patterns directed by diverse *elt-2* regulatory sequences in *C*. *elegans*. (A-C) *C*. *elegans unc-25* regulatory sequence drives expression of *mCherry* in all transgenic strains; (A) *C*. *briggsae*, (B) *M*. *hapla*, (C) *B*. *malayi elt-2* regulatory sequences drive expression of *GFP*. Stages shown are 8E, comma, and pretzel embryonic stages, with L1 larval stage below. Animals photographed at 400x magnification.</***SI_Caption>***
(PDF)

S6 Fig Motifs with identity between *C*. *elegans* and orthologous *unc-47* upstream sequences. All blocks of sequence identity in window sizes shown for each comparison with positions within the upstream non-coding sequence.
(DOCX)

S7 Fig Motifs with identity between *C*. *elegans* and orthologous *unc-25* upstream sequences. Motifs with identity between *C*. *elegans* and orthologous *unc-25* upstream sequences. All blocks of sequence identity in window sizes shown for each comparison with positions within the upstream non-coding sequence.
(DOCX)

S8 Fig Motifs with identity between *C*. *elegans* and orthologous *mec-3* upstream sequences. Motifs with identity between *C*. *elegans* and orthologous *mec-3* upstream sequences. All blocks of sequence identity in window sizes shown for each comparison with positions within the upstream non-coding sequence.
(DOCX)

S9 Fig Motifs with identity between *C*. *elegans* and orthologous *elt-2* upstream sequences. Motifs with identity between *C*. *elegans* and orthologous *elt-2* upstream sequences. All blocks of sequence identity in window sizes shown for each comparison with positions within the upstream non-coding sequence.
(DOCX)

S10 Fig Sequence identity in orthologous upstream sequences is not greater than expected by chance. Graphs showing the proportion of sequence similarity over different window sizes for *unc-25*, *mec-3*, and *elt-2*. The number of identical 10 nt blocks between each of the nematode relatives and the *C*. *elegans* upstream sequence of the same genes shown in red, on histograms showing the number of predicted 10 nt matches between the relatives’ sequences and 1000 reshuffled *C*. *elegans* sequences that preserve tri-nucleotide frequencies.
(PDF)

S11 Fig Matches to motifs responsible for the *C*. *elegans unc-25* gene expression pattern can be found in orthlogous sequences. Cartoons depicting all orthologous upstream *unc-25* sequences fused to *mCherry* (*C*. *elegans*) or *GFP* (all others) near the translation start site (bent arrow), or further downstream. Exons are thick black boxes, introns are gray lines. UNC-30 (box arrow) consensus motifs are shown above, in uppercase letters; conserved flanking nucleotides shown in lowercase. Locations of motifs relative to the endogenous translation start site are indicated. Daggers denote binding sites found to be bound by UNC-30. See S1 Text.
(PDF)

S12 Fig Matches to motifs responsible for the *C*. *elegans mec-3* gene expression pattern can be found in orthlogous sequences. Cartoons depicting the all orthologous upstream *mec-3* sequences fused to *GFP* near the translation start site (bent arrow) or further downstream. Exons are thick black boxes, introns are gray lines. UNC-86 (triangle) and MEC-3 (fletched arrow) consensus motifs are shown above. Locations of motifs relative to the endogenous translation start site are indicated. Several distal motifs are omitted from *C*. *elegans*, *C*. *briggsae*, and *B*. *malayi*. See S1 Text.
(PDF)

S13 Fig Matches to motifs responsible for the *C*. *elegans elt-2* gene expression pattern can be found in orthlogous sequences. Cartoons depicting the all orthologous upstream *elt-2* sequences fused to *GFP* near the translation start site (bent arrow). Gut-enriched extended GATA motif (sideways heart) and generic GATA motif (vertical lines) are shown above. Locations of motifs relative to the endogenous translation start site are indicated. See S1 Text.</**SI_Caption>**
(PDF)

S14 Fig Mutations to putative transcription factor binding sites in orthologous *cis*-regulatory sequences disrupt their functions. (A) Mutant *M*. *hapla unc-47* element drives expression of *GFP*. *C*. *elegans unc-47* regulatory sequence drives expression of *mCherry*. Images are mosaics of single animals. Mutant *T*. *spiralis unc-47* regulatory element drives expression of *GFP*, but not consistently in RIS (B) or DVB (C). μ denotes mutated *unc-47 cis* elements. *C*. *elegans unc-47* regulatory sequence drives expression of *mCherry* in these cells. All animals photographed at 400x magnification.
(PDF)

S15 Fig *GFP* expression driven by *cis* elements from distant relatives in the appropriate cells is detected when not coexpressed with mCherry. (A) *M*. *hapla unc-47*::*GFP* is expressed in several D-type neurons of the ventral cord. (B) *T*. *spiralis unc-47*::*GFP* is expressed in RIS and DVB. (C) *M*. *hapla mec-3*::*GFP* is expressed in the head neuron FLP.
(PDF)

S1 Table Cell counts for strains reported in Figs **2**–5 and 8. (XLSX)

S2 Table Statistical analyses of gene expression differences. (XLSX)

S3 Table Instances of *unc-47* regulatory motifs in other *cis* elements (XLSX)

S1 Text Short motifs with identity to *C*. *elegans* binding sites are present in promoters of all genes examined. (DOCX)
