# Supplementary material for: Phylum-Level Conservation of Regulatory Information in Nematodes despite Extensive Non-coding Sequence Divergence
Source: PLoS Genet. 2015 May 28;11(5):e1005268. doi: 10.1371/journal.pgen.1005268 (PMC4447282; doi:10.1371/journal.pgen.1005268)
Supplement: S2 Fig — (A-C) C. elegans unc-25 regulatory sequence drives expression of mCherry in all transgenic strains; (A) M. hapla, (B) B. malayi, (C) T. spiralis unc-25 regulatory sequences drive expression of GFP. Animals photographed at 400x magnification. Images are mosaics of single animals. (PDF) [file pgen.1005268.s002.pdf]

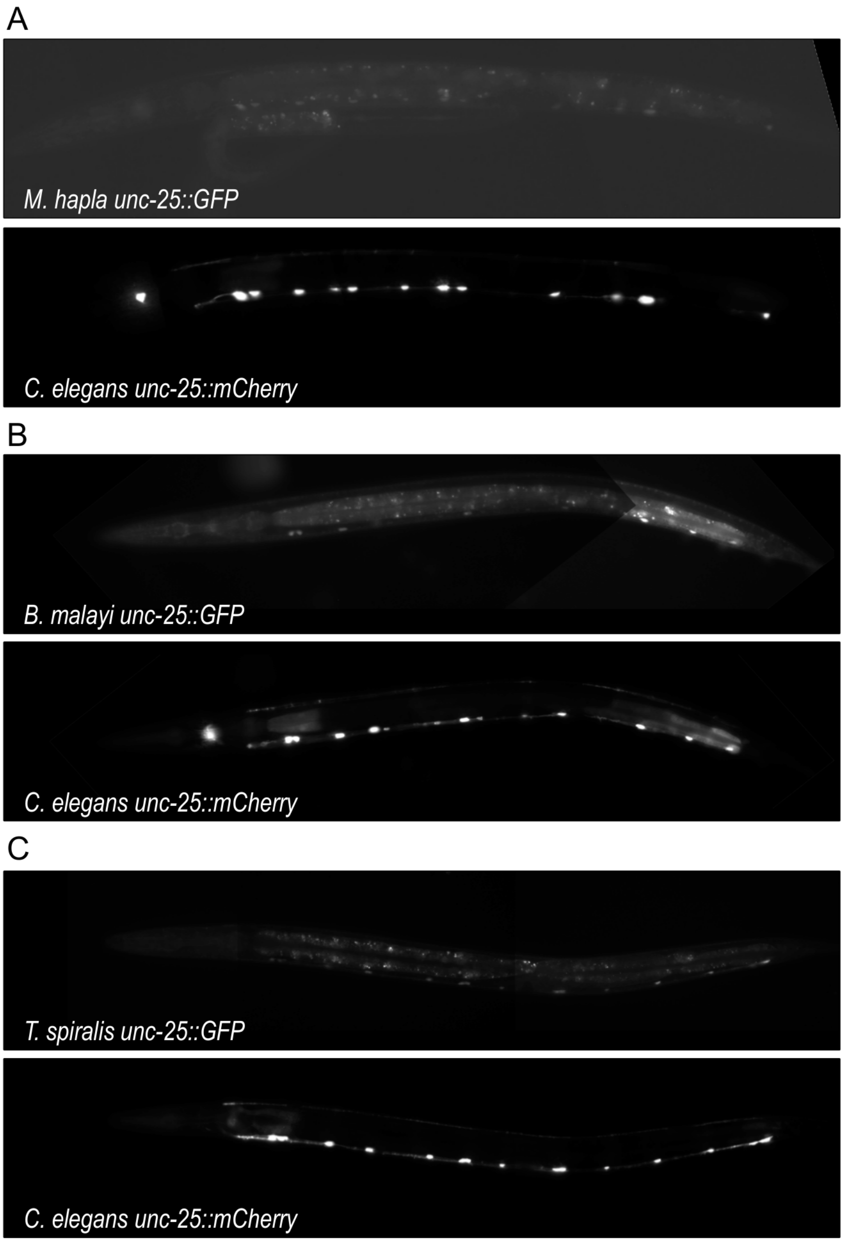


**S2 Figure. Expression patterns directed by diverse *unc-25* regulatory sequences in *C. elegans.*** (A-C) *C. elegans unc-25* regulatory sequence drives expression of *mCherry* in all transgenic strains; (A) *M. hapla,* (B) *B. malayi,* (C) *T. spiralis unc-25* regulatory sequences drive expression of *GFP.* Animals photographed at 400x magnification. Images are mosaics of single animals.
