# Supplementary material for: Phylum-Level Conservation of Regulatory Information in Nematodes despite Extensive Non-coding Sequence Divergence
Source: PLoS Genet. 2015 May 28;11(5):e1005268. doi: 10.1371/journal.pgen.1005268 (PMC4447282; doi:10.1371/journal.pgen.1005268)
Supplement: S3 Fig — (A-D) C. elegans mec3 regulatory sequence drives expression of mCherry in all transgenic strains; (A) C. briggsae, (B) M. hapla, (C) B. malayi, (D) T. spiralis mec-3 regulatory sequences drive expression of GFP. Animals photographed at 400x magnification. Images are mosaics of single animals. (PDF) [file pgen.1005268.s003.pdf]

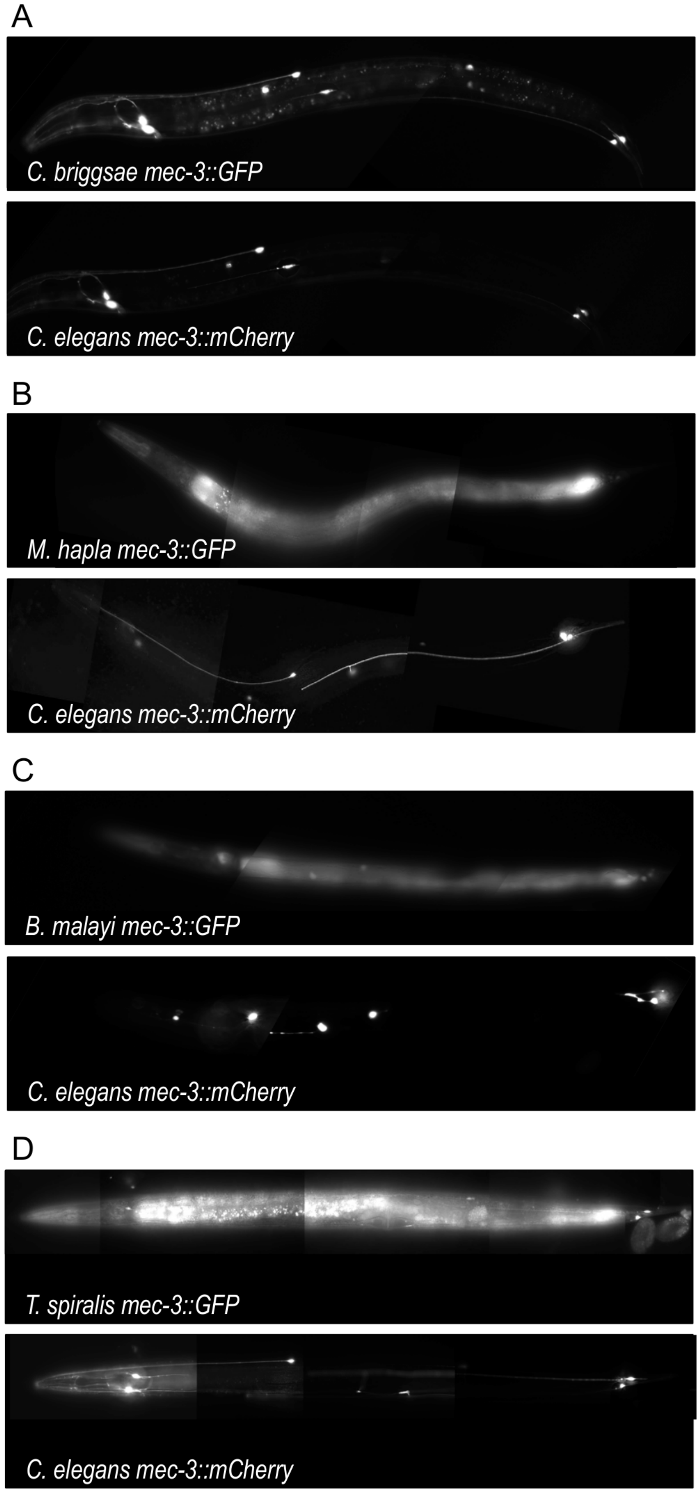

S3 Figure. Expression patterns directed by diverse *mec-3* regulatory sequences in *C. elegans.* (A-D) *C. elegans mec3* regulatory sequence drives expression of *mCherry* in all transgenic strains; (A) *C. briggsae,* (B) *M. hapla,* (C) *B. malayi,* (D) *T. spiralis mec-3* regulatory sequences drive expression of *GFP*. Animals photographed at 400x magnification. Images are mosaics of single animals.
