# Supplementary material for: Phylum-Level Conservation of Regulatory Information in Nematodes despite Extensive Non-coding Sequence Divergence
Source: PLoS Genet. 2015 May 28;11(5):e1005268. doi: 10.1371/journal.pgen.1005268 (PMC4447282; doi:10.1371/journal.pgen.1005268)
Supplement: S4 Fig — (A) B. malayi mec-3 regulatory sequence drives expression of GFP. Animal photographed at 400x magnification, ventral cord at bottom. Image is a mosaic of single animals. (B) 1000x magnification of animal with ventral side up. (C) 1000x magnification of animal with ventral side up, vulva at center. (PDF) [file pgen.1005268.s004.pdf]

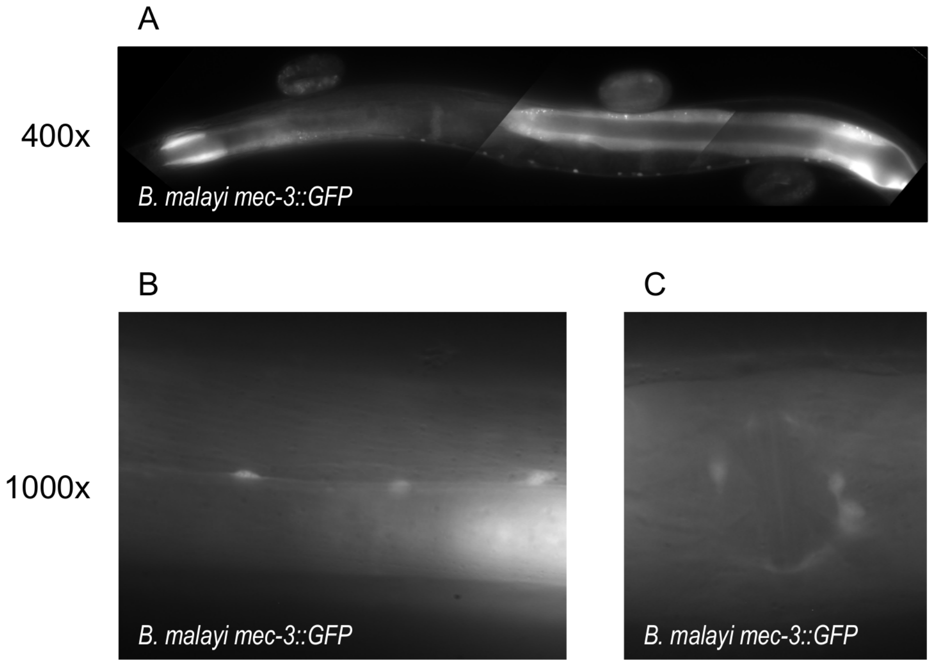

S4 Figure. ***B. malayi mec-3*** **regulatory sequence drives expression in ventral cord neurons.**

(A) *B. malayi mec-3* regulatory sequence drives expression of *GFP*. Animal photographed at 400x magnification, ventral cord at bottom. Image is a mosaic of single animals. (B) 1000x magnification of animal with ventral side up. (C) 1000x magnification of animal with ventral side up, vulva at center.
