# Supplementary material for: Phylum-Level Conservation of Regulatory Information in Nematodes despite Extensive Non-coding Sequence Divergence
Source: PLoS Genet. 2015 May 28;11(5):e1005268. doi: 10.1371/journal.pgen.1005268 (PMC4447282; doi:10.1371/journal.pgen.1005268)
Supplement: S5 Fig — (A-C) C. elegans unc-25 regulatory sequence drives expression of mCherry in all transgenic strains; (A) C. briggsae, (B) M. hapla, (C) B. malayi elt-2 regulatory sequences drive expression of GFP. Stages shown are 8E, comma, and pretzel embryonic stages, with L1 larval stage below. Animals photographed at 400x magnification. (PDF) [file pgen.1005268.s005.pdf]

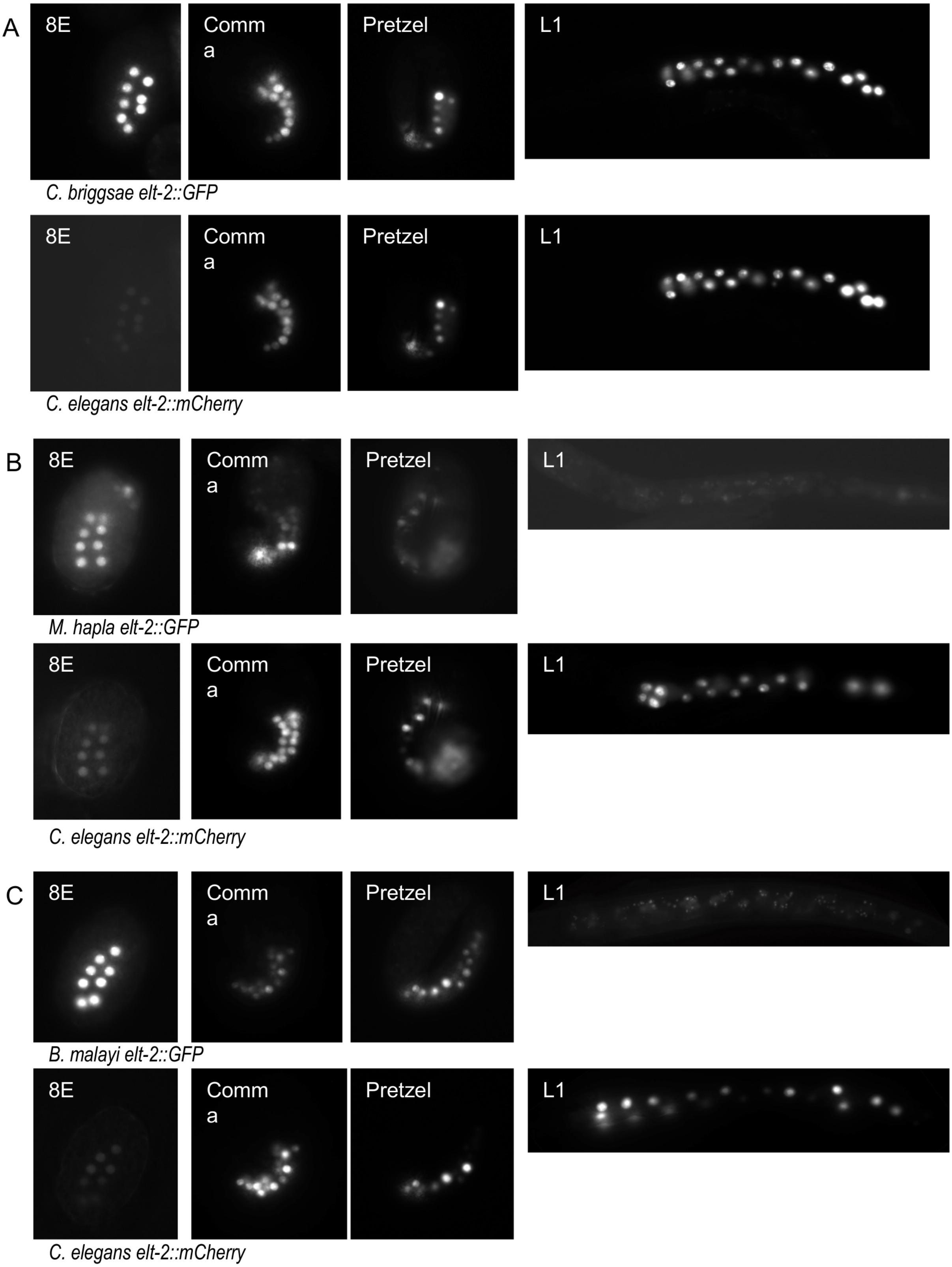

S5 Figure. Expression patterns directed by diverse *elt-2* regulatory sequences in *C. elegans.* See legend next page.

S5 Figure. Expression patterns directed by diverse *elt-2* regulatory sequences in *C. elegans.*

(A-C) *C. elegans unc-25* regulatory sequence drives expression of *mCherry* in all transgenic strains; (A) *C. briggsae,* (B) *M. hapla,* (C) *B. malayi elt-2* regulatory sequences drive expression of *GFP.* Stages shown are 8E, comma, and pretzel embryonic stages, with L1 larval stage below. Animals photographed at 400x magnification.
