# Supplementary material for: Phylum-Level Conservation of Regulatory Information in Nematodes despite Extensive Non-coding Sequence Divergence
Source: PLoS Genet. 2015 May 28;11(5):e1005268. doi: 10.1371/journal.pgen.1005268 (PMC4447282; doi:10.1371/journal.pgen.1005268)
Supplement: S6 Fig — All blocks of sequence identity in window sizes shown for each comparison with positions within the upstream non-coding sequence. (DOCX) [file pgen.1005268.s006.docx]

**S6 Figure. Motifs with identity between *C. elegans* and orthologous *unc-47* upstream sequences.** All blocks of sequence identity in window sizes shown for each comparison with positions within the upstream non-coding sequence.

**C. briggsae/C. elegans unc-47, 9bp window**

Alignment Length: 11; Identity: 11

| **seq 1:** | **cbrunc47** | Seq 1 | 1081 | CAAATTTCCGG | 1091 |
| --- | --- | --- | --- | --- | --- |
| **seq 2:** | **celunc47** |  |  | \|\|\|\|\|\|\|\|\|\|\| |  |
|  |  | Seq 2 | 862 | CAAATTTCCGG | 872 |

Alignment Length: 9; Identity: 9

Seq 1 225 ATTGAAAAT 233

|  | \|\|\|\|\|\|\|\|\| |  | Seq 1 | 1082 | AAATTTCCGG | 1091 |
| --- | --- | --- | --- | --- | --- | --- |
| Seq 2 156 | ATTGAAAAT | 164 |  |  | \|\|\|\|\|\|\|\|\|\| |  |
|  |  |  | Seq 2 | 161 | AAATTTCCGG | 170 |

Alignment Length: 10; Identity: 10

Alignment Length: 11; Identity: 11

Seq 1 344 ACAGTCGAAAG 354

|  | \|\|\|\|\|\|\|\|\|\|\| |  | Seq 1 | 1130 | CTCTTTTCC | 1138 |
| --- | --- | --- | --- | --- | --- | --- |
| Seq 2 8 | ACAGTCGAAAG | 18 |  |  | \|\|\|\|\|\|\|\|\| |  |
|  |  |  | Seq 2 | 1111 | CTCTTTTCC | 1119 |

Alignment Length: 9; Identity: 9

Alignment Length: 9; Identity: 9

Seq 1 386 GACCAAATT 394

|||||||||

Seq 2 829 GACCAAATT 837

Alignment Length: 9; Identity: 9

Seq 1 388 CCAAATTTC 396

Alignment Length: 9; Identity: 9

Seq 1 1154 ATCCATCAG 1162

|||||||||

Seq 2 946 ATCCATCAG 954

Alignment Length: 9; Identity: 9

|  | \|\|\|\|\|\|\|\|\| |  | Seq 1 | 1169 | AAAAAGAAA | 1177 |
| --- | --- | --- | --- | --- | --- | --- |
| Seq 2 861 | CCAAATTTC | 869 |  |  | \|\|\|\|\|\|\|\|\| |  |
|  |  |  | Seq 2 | 843 | AAAAAGAAA | 851 |

Alignment Length: 10; Identity: 10

Seq 1 541 CGTTTTTTTT 550

|  | \|\|\|\|\|\|\|\|\|\| |  | Seq 1 | 1209 | CGTCTAATAA | 1218 |
| --- | --- | --- | --- | --- | --- | --- |
| Seq 2 882 | CGTTTTTTTT | 891 |  |  | \|\|\|\|\|\|\|\|\|\| |  |
|  |  |  | Seq 2 | 406 | CGTCTAATAA | 415 |

Alignment Length: 10; Identity: 10

Alignment Length: 9; Identity: 9

Seq 1 545 TTTTTTTTG 553

|  | \|\|\|\|\|\|\|\|\| |  | Seq 1 | 1210 | GTCTAATAATCCC 1222 |
| --- | --- | --- | --- | --- | --- |
| Seq 2 884 | TTTTTTTTG | 892 |  |  | \|\|\|\|\|\|\|\|\|\|\|\|\| |
|  |  |  | Seq 2 | 997 | GTCTAATAATCCC 1009 |

Alignment Length: 13; Identity: 13

Alignment Length: 9; Identity: 9

Seq 1 549 TTTTGAAAA 557

|||||||||

Seq 2 324 TTTTGAAAA 332

Alignment Length: 9; Identity: 9

Seq 1 669 GTTTTTTTT 677

|||||||||

Seq 2 883 GTTTTTTTT 891

Alignment Length: 9; Identity: 9

Seq 1 680 TTTTCAAAA 688

|||||||||

Seq 2 376 TTTTCAAAA 384

Alignment Length: 10; Identity: 10

Seq 1 724 TCCCAAATTT 733

Alignment Length: 23; Identity: 23

Seq 1 1225 CTTCAAATCATTGTGCCAACACA 1247

|||||||||||||||||||||||

Seq 2 1017 CTTCAAATCATTGTGCCAACACA 1039

Alignment Length: 18; Identity: 18

Seq 1 1243 ACACAGACACACTTTATG 1260

||||||||||||||||||

Seq 2 1037 ACACAGACACACTTTATG 1054

Alignment Length: 17; Identity: 17

Seq 1 1270 CACGCTATTTGAAGAGC 1286

|||||||||||||||||

Seq 2 1069 CACGCTATTTGAAGAGC 1085

Alignment Length: 10; Identity: 10

|  | \|\|\|\|\|\|\|\|\|\| |  | Seq 1 | 1288 | ACGACGACGA | 1297 |
| --- | --- | --- | --- | --- | --- | --- |
| Seq 2 859 | TCCCAAATTT | 868 |  |  | \|\|\|\|\|\|\|\|\|\| |  |
|  |  |  | Seq 2 | 1090 | ACGACGACGA | 1099 |

Alignment Length: 9; Identity: 9

Seq 1 959 TTTCAAAAA 967

Alignment Length: 9; Identity: 9

| Seq 2 377 | \|\|\|\|\|\|\|\|\|  TTTCAAAAA | 385 | Seq 1  Seq 2 | 1288  1093 | ACGACGACG  \|\|\|\|\|\|\|\|\| ACGACGACG | 1296  1101 |
| --- | --- | --- | --- | --- | --- | --- |
| Alignment Length: 10; Identity: 10 | | | | | | |
| Seq 1 1040  Seq 2 443 | ATTGATGTTC  \|\|\|\|\|\|\|\|\|\| ATTGATGTTC | 1049  452 | | | | |

**S6 Figure, continued.** Motifs with identity between *C. elegans* and orthologous *unc-47* upstream sequences

Alignment Length: 13; Identity: 13

Seq 1 1320 CAGAGCTCTTTTC 1332

Alignment Length: 9; Identity: 9

|  | \|\|\|\|\|\|\|\|\|\|\|\|\| |  | Seq 1 | 681 | AAAGAAAAA | 673 |
| --- | --- | --- | --- | --- | --- | --- |
| Seq 2 1106 | CAGAGCTCTTTTC | 1118 |  |  | \|\|\|\|\|\|\|\|\| |  |
|  |  |  | Seq 2 | 358 | AAAGAAAAA | 366 |

**OPPOSITE STRAND**

Alignment Length: 9; Identity: 9

Alignment Length: 9; Identity: 9

| Seq 1 1348 | AAAGAAGAG | 1340 | Seq 1 | 679 | AGAAAAAAA | 671 |
| --- | --- | --- | --- | --- | --- | --- |
|  | \|\|\|\|\|\|\|\|\| |  |  |  | \|\|\|\|\|\|\|\|\| |  |
| Seq 2 967 | AAAGAAGAG | 975 | Seq 2 | 839 | AGAAAAAAA | 847 |

Alignment Length: 12; Identity: 12

Alignment Length: 10; Identity: 10

| Seq 1 1287 | TGCTCTTCAAAT | 1276 | Seq 1 | 557 | TTTTCAAAAA | 548 |
| --- | --- | --- | --- | --- | --- | --- |
|  | \|\|\|\|\|\|\|\|\|\|\|\| |  |  |  | \|\|\|\|\|\|\|\|\|\| |  |
| Seq 2 1013 | TGCTCTTCAAAT | 1024 | Seq 2 | 376 | TTTTCAAAAA | 385 |

Alignment Length: 10; Identity: 10

Alignment Length: 9; Identity: 9

| Seq 1 1091 | CCGGAAATTT | 1082 | Seq 1 491 | TCTAATAAT | 483 |
| --- | --- | --- | --- | --- | --- |
|  | \|\|\|\|\|\|\|\|\|\| |  |  | \|\|\|\|\|\|\|\|\| |  |
| Seq 2 648 | CCGGAAATTT | 657 | Seq 2 998 | TCTAATAAT | 1006 |

Alignment Length: 9; Identity: 9

Seq 1 1029 ATTCAGAAA 1021

|  | \|\|\|\|\|\|\|\|\| |  | Seq 1 | 233 | ATTTTCAAT | 225 |
| --- | --- | --- | --- | --- | --- | --- |
| Seq 2 835 | ATTCAGAAA | 843 |  |  | \|\|\|\|\|\|\|\|\| |  |
|  |  |  | Seq 2 | 619 | ATTTTCAAT | 627 |

Alignment Length: 9; Identity: 9

Alignment Length: 9; Identity: 9

Seq 1 1018 CATTACAGA 1010

|  | \|\|\|\|\|\|\|\|\| |  | Seq 1 | 210 | CGGTTTTCCAA | 200 |
| --- | --- | --- | --- | --- | --- | --- |
| Seq 2 1184 | CATTACAGA | 1192 |  |  | \|\|\|\|\|\|\|\|\|\|\| |  |
|  |  |  | Seq 2 | 599 | CGGTTTTCCAA | 609 |

Alignment Length: 11; Identity: 11

Alignment Length: 9; Identity: 9

Seq 1 1013 CAGAAAAAA 1005

|  | \|\|\|\|\|\|\|\|\| |  | Seq 1 | 55 | TTTGAAAAGT | 46 |
| --- | --- | --- | --- | --- | --- | --- |
| Seq 2 838 | CAGAAAAAA | 846 |  |  | \|\|\|\|\|\|\|\|\|\| |  |
|  |  |  | Seq 2 | 325 | TTTGAAAAGT | 334 |

Alignment Length: 10; Identity: 10

Alignment Length: 12; Identity: 12

Seq 1 1013 CAGAAAAAAGAA 1002

||||||||||||

Seq 2 961 CAGAAAAAAGAA 972

Alignment Length: 9; Identity: 9

Seq 1 1010 AAAAAAGAA 1002

|||||||||

Seq 2 842 AAAAAAGAA 850

Alignment Length: 9; Identity: 9

| Seq 1 874 | TCAGAAAAA | 866 |
| --- | --- | --- |
|  | \|\|\|\|\|\|\|\|\| |  |
| Seq 2 837 | TCAGAAAAA | 845 |

Alignment Length: 10; Identity: 10

Seq 1 54 TTGAAAAGTT 45

||||||||||

Seq 2 526 TTGAAAAGTT 535

Alignment Length: 11; Identity: 11

| Seq 1 783 | AAGAAGAGCCT | 773 |
| --- | --- | --- |
|  | \|\|\|\|\|\|\|\|\|\|\| |  |
| Seq 2 968 | AAGAAGAGCCT | 978 |

Alignment Length: 10; Identity: 10

| Seq 1 689 | ATTTTGAAAA | 680 |
| --- | --- | --- |
|  | \|\|\|\|\|\|\|\|\|\| |  |
| Seq 2 323 | ATTTTGAAAA | 332 |

Alignment Length: 9; Identity: 9

| Seq 1 683 | AAAAAGAAA | 675 |
| --- | --- | --- |
|  | \|\|\|\|\|\|\|\|\| |  |
| Seq 2 843 | AAAAAGAAA | 851 |

**S6 Figure. continued.** Motifs with identity between *C. elegans* and orthologous *unc-47* upstream sequences

**M. hapla/C. elegans 9bp window**

Alignment Length: 10; Identity: 10

| **seq 1:** | **mhaunc47** | Seq 1 | 886 | AAATAAAATG | 895 |
| --- | --- | --- | --- | --- | --- |
| **seq 2:** | **celunc47** |  |  | \|\|\|\|\|\|\|\|\|\| |  |
|  |  | Seq 2 | 772 | AAATAAAATG | 781 |

Alignment Length: 9; Identity: 9

Alignment Length: 10; Identity: 10

| Seq 1 63 | CCATCAGTG | 71 |
| --- | --- | --- |
|  | \|\|\|\|\|\|\|\|\| |  |
| Seq 2 948 | CCATCAGTG | 956 |

Alignment Length: 9; Identity: 9

| Seq 1 959 | AAAAATGGAA | 968 |
| --- | --- | --- |
|  | \|\|\|\|\|\|\|\|\|\| |  |
| Seq 2 362 | AAAAATGGAA | 371 |

Alignment Length: 10; Identity: 10

| Seq 1 81 | ACATTTTTA | 89 |
| --- | --- | --- |
|  | \|\|\|\|\|\|\|\|\| |  |
| Seq 2 752 | ACATTTTTA | 760 |

Alignment Length: 9; Identity: 9

| Seq 1 1084 | AAAAATGGAA | 1093 |
| --- | --- | --- |
|  | \|\|\|\|\|\|\|\|\|\| |  |
| Seq 2 362 | AAAAATGGAA | 371 |

Alignment Length: 9; Identity: 9

| Seq 1 85 | TTTTAAGTG | 93 |
| --- | --- | --- |
|  | \|\|\|\|\|\|\|\|\| |  |
| Seq 2 393 | TTTTAAGTG | 401 |

Alignment Length: 9; Identity: 9

| Seq 1 1098 | AAAAGAAGA | 1106 |
| --- | --- | --- |
|  | \|\|\|\|\|\|\|\|\| |  |
| Seq 2 966 | AAAAGAAGA | 974 |

Alignment Length: 9; Identity: 9

| Seq 1 265 | AAATTGCTG | 273 |
| --- | --- | --- |
|  | \|\|\|\|\|\|\|\|\| |  |
| Seq 2 187 | AAATTGCTG | 195 |

Alignment Length: 9; Identity: 9

| Seq 1 1104 | AGAAAAAAA | 1112 |
| --- | --- | --- |
|  | \|\|\|\|\|\|\|\|\| |  |
| Seq 2 839 | AGAAAAAAA | 847 |

Alignment Length: 9; Identity: 9

| Seq 1 383 | AAAAAATTT | 391 |
| --- | --- | --- |
|  | \|\|\|\|\|\|\|\|\| |  |
| Seq 2 370 | AAAAAATTT | 378 |

| Seq 1 1221 | TTTTTTTTG | 1229 |
| --- | --- | --- |
|  | \|\|\|\|\|\|\|\|\| |  |
| Seq 2 884 | TTTTTTTTG | 892 |

Alignment Length: 11; Identity: 11

Seq 1 385 AAAATTTAATT 395

|||||||||||

Seq 2 790 AAAATTTAATT 800

Alignment Length: 9; Identity: 9

**OPPOSITE STRAND**

Alignment Length: 12; Identity: 12

| Seq 1 | 1202 | AAAAATGGAAAA | 1191 |
| --- | --- | --- | --- |
|  |  | \|\|\|\|\|\|\|\|\|\|\|\| |  |
| Seq 2 | 362 | AAAAATGGAAAA | 373 |

Alignment Length: 9; Identity: 9

| Seq 1 403 | AAAAATTTT | 411 |
| --- | --- | --- |
|  | \|\|\|\|\|\|\|\|\| |  |
| Seq 2 371 | AAAAATTTT | 379 |

Alignment Length: 9; Identity: 9

| Seq 1 1169 | TTTTGCCAA | 1161 |
| --- | --- | --- |
|  | \|\|\|\|\|\|\|\|\| |  |
| Seq 2 888 | TTTTGCCAA | 896 |

Alignment Length: 9; Identity: 9

| Seq 1 408 | TTTTTTTTG | 416 |
| --- | --- | --- |
|  | \|\|\|\|\|\|\|\|\| |  |
| Seq 2 884 | TTTTTTTTG | 892 |

Alignment Length: 9; Identity: 9

| Seq 1 1114 | GTTTTTTTT | 1106 |
| --- | --- | --- |
|  | \|\|\|\|\|\|\|\|\| |  |
| Seq 2 883 | GTTTTTTTT | 891 |

Alignment Length: 9; Identity: 9

| Seq 1 411 | TTTTTGGAA | 419 |
| --- | --- | --- |
|  | \|\|\|\|\|\|\|\|\| |  |
| Seq 2 260 | TTTTTGGAA | 268 |

Alignment Length: 9; Identity: 9

| Seq 1 | 1075 | AAATTGATG | 1067 |
| --- | --- | --- | --- |
|  |  | \|\|\|\|\|\|\|\|\| |  |
| Seq 2 | 441 | AAATTGATG | 449 |

Alignment Length: 10; Identity: 10

| Seq 1 708 | AATTGAAAA | 716 |
| --- | --- | --- |
|  | \|\|\|\|\|\|\|\|\| |  |
| Seq 2 155 | AATTGAAAA | 163 |

Alignment Length: 9; Identity: 9

| Seq 1 | 1060 | ATGGAAAAAA | 1051 |
| --- | --- | --- | --- |
|  |  | \|\|\|\|\|\|\|\|\|\| |  |
| Seq 2 | 366 | ATGGAAAAAA | 375 |

Alignment Length: 9; Identity: 9

| Seq 1 708 | AATTGAAAA | 716 |
| --- | --- | --- |
|  | \|\|\|\|\|\|\|\|\| |  |
| Seq 2 524 | AATTGAAAA | 532 |

Alignment Length: 10; Identity: 10

| Seq 1 | 1057 | GAAAAAAAG | 1049 |
| --- | --- | --- | --- |
|  |  | \|\|\|\|\|\|\|\|\| |  |
| Seq 2 | 840 | GAAAAAAAG | 848 |

Seq 1 714 AAAAAATTTT 723

||||||||||

Seq 2 370 AAAAAATTTT 379

**S6 Figure, continued.** Motifs with identity between *C. elegans* and orthologous *unc-47* upstream sequences

Alignment Length: 10; Identity: 10

| Seq 1 1047 | AATAAAAAAA | 1038 |
| --- | --- | --- |
|  | \|\|\|\|\|\|\|\|\|\| |  |
| Seq 2 411 | AATAAAAAAA | 420 |

Alignment Length: 9; Identity: 9

| Seq 1 965 | CATTTTTAG | 957 |
| --- | --- | --- |
|  | \|\|\|\|\|\|\|\|\| |  |
| Seq 2 753 | CATTTTTAG | 761 |

Alignment Length: 9; Identity: 9

| Seq 1 927 | ATAATCCCC | 919 |
| --- | --- | --- |
|  | \|\|\|\|\|\|\|\|\| |  |
| Seq 2 1002 | ATAATCCCC | 1010 |

Alignment Length: 10; Identity: 10

| Seq 1 908 | AACATTTTTA | 899 |
| --- | --- | --- |
|  | \|\|\|\|\|\|\|\|\|\| |  |
| Seq 2 751 | AACATTTTTA | 760 |

Alignment Length: 9; Identity: 9

| Seq 1 773 | CATCTATTT | 765 |
| --- | --- | --- |
|  | \|\|\|\|\|\|\|\|\| |  |
| Seq 2 318 | CATCTATTT | 326 |

Alignment Length: 9; Identity: 9

| Seq 1 751 | CACTTTTTG | 743 |
| --- | --- | --- |
|  | \|\|\|\|\|\|\|\|\| |  |
| Seq 2 257 | CACTTTTTG | 265 |

Alignment Length: 11; Identity: 11

| Seq 1 726 | GAAAAAATTTT | 716 |
| --- | --- | --- |
|  | \|\|\|\|\|\|\|\|\|\|\| |  |
| Seq 2 369 | GAAAAAATTTT | 379 |

Alignment Length: 9; Identity: 9

| Seq 1 716 | TTTTCAATT | 708 |
| --- | --- | --- |
|  | \|\|\|\|\|\|\|\|\| |  |
| Seq 2 620 | TTTTCAATT | 628 |

Alignment Length: 9; Identity: 9

| Seq 1 654 | GGTGGCAAG | 646 |
| --- | --- | --- |
|  | \|\|\|\|\|\|\|\|\| |  |
| Seq 2 21 | GGTGGCAAG | 29 |

Alignment Length: 9; Identity: 9

| Seq 1 580 | AGAAAAATG | 572 |
| --- | --- | --- |
|  | \|\|\|\|\|\|\|\|\| |  |
| Seq 2 360 | AGAAAAATG | 368 |

Alignment Length: 9; Identity: 9

| Seq 1 488 | AGCAAATTC | 480 |
| --- | --- | --- |
|  | \|\|\|\|\|\|\|\|\| |  |
| Seq 2 336 | AGCAAATTC | 344 |

Alignment Length: 10; Identity: 10

| Seq 1 413 | AAAAAATTTT | 404 |
| --- | --- | --- |
|  | \|\|\|\|\|\|\|\|\|\| |  |
| Seq 2 370 | AAAAAATTTT | 379 |

Alignment Length: 9; Identity: 9

| Seq 1 105 | GCACTTTTT | 97 |
| --- | --- | --- |
|  | \|\|\|\|\|\|\|\|\| |  |
| Seq 2 256 | GCACTTTTT | 264 |

**S6 Figure, continued.** Motifs with identity between *C. elegans* and orthologous *unc-47* upstream sequences

**B. malayi/C. elegans 9bp window**

**Seq 1: bmaunc47**

**Seq 2: celunc47**

Alignment Length: 9; Identity: 9

| Seq 1 114 | TTGCTGATT | 122 |
| --- | --- | --- |
|  | \|\|\|\|\|\|\|\|\| |  |
| Seq 2 190 | TTGCTGATT | 198 |

Alignment Length: 9; Identity: 9

Seq 1 1064 AAATAAAAT 1072

|||||||||

Seq 2 772 AAATAAAAT 780

Alignment Length: 9; Identity: 9

Alignment Length: 10; Identity: 10

| Seq 1 1141 | TTTCAAAAA | 1149 |
| --- | --- | --- |
|  | \|\|\|\|\|\|\|\|\| |  |
| Seq 2 377 | TTTCAAAAA | 385 |

Alignment Length: 9; Identity: 9

| Seq 1 227 | ATTGCCAATA | 236 |
| --- | --- | --- |
|  | \|\|\|\|\|\|\|\|\|\| |  |
| Seq 2 113 | ATTGCCAATA | 122 |

Alignment Length: 10; Identity: 10

| Seq 1 1470 | GTTTTTTTT | 1478 |
| --- | --- | --- |
|  | \|\|\|\|\|\|\|\|\| |  |
| Seq 2 883 | GTTTTTTTT | 891 |

Alignment Length: 9; Identity: 9

| Seq 1 228 | TTGCCAATAA | 237 |
| --- | --- | --- |
|  | \|\|\|\|\|\|\|\|\|\| |  |
| Seq 2 890 | TTGCCAATAA | 899 |

Alignment Length: 9; Identity: 9

| Seq 1 1472 | TTTTTTTTG | 1480 |
| --- | --- | --- |
|  | \|\|\|\|\|\|\|\|\| |  |
| Seq 2 884 | TTTTTTTTG | 892 |

Alignment Length: 9; Identity: 9

| Seq 1 432 | AAAAAAAGA | 440 |
| --- | --- | --- |
|  | \|\|\|\|\|\|\|\|\| |  |
| Seq 2 841 | AAAAAAAGA | 849 |

Alignment Length: 9; Identity: 9

| Seq 1 1601 | ATATTGAAA | 1609 |
| --- | --- | --- |
|  | \|\|\|\|\|\|\|\|\| |  |
| Seq 2 120 | ATATTGAAA | 128 |

Alignment Length: 9; Identity: 9

| Seq 1 466 | CATCTATTT | 474 |
| --- | --- | --- |
|  | \|\|\|\|\|\|\|\|\| |  |
| Seq 2 318 | CATCTATTT | 326 |

Alignment Length: 9; Identity: 9

| Seq 1 1627 | GTTTTTTTT | 1635 |
| --- | --- | --- |
|  | \|\|\|\|\|\|\|\|\| |  |
| Seq 2 883 | GTTTTTTTT | 891 |

Alignment Length: 10; Identity: 10

| Seq 1 522 | TAATTCAGG | 530 |
| --- | --- | --- |
|  | \|\|\|\|\|\|\|\|\| |  |
| Seq 2 658 | TAATTCAGG | 666 |

Alignment Length: 9; Identity: 9

| Seq 1 1704 | TTGGAAATTT | 1713 |
| --- | --- | --- |
|  | \|\|\|\|\|\|\|\|\|\| |  |
| Seq 2 263 | TTGGAAATTT | 272 |

Alignment Length: 9; Identity: 9

| Seq 1 573 | CATTTTTAG | 581 |
| --- | --- | --- |
|  | \|\|\|\|\|\|\|\|\| |  |
| Seq 2 753 | CATTTTTAG | 761 |

Alignment Length: 10; Identity: 10

| Seq 1 1713 | TAAAATTTA | 1721 |
| --- | --- | --- |
| Seq 2 789 | \|\|\|\|\|\|\|\|\| TAAAATTTA | 797 |

Alignment Length: 9; Identity: 9

| Seq 1 580 | AGAAATAAAA | 589 |
| --- | --- | --- |
|  | \|\|\|\|\|\|\|\|\|\| |  |
| Seq 2 770 | AGAAATAAAA | 779 |

Alignment Length: 9; Identity: 9

| Seq 1 1794 | GTTTTTTTT | 1802 |
| --- | --- | --- |
|  | \|\|\|\|\|\|\|\|\| |  |
| Seq 2 883 | GTTTTTTTT | 891 |

Alignment Length: 10; Identity: 10

| Seq 1 590 | ATTGAAATA | 598 |
| --- | --- | --- |
|  | \|\|\|\|\|\|\|\|\| |  |
| Seq 2 122 | ATTGAAATA | 130 |

Alignment Length: 10; Identity: 10

| Seq 1 1797 | TTTTTTTTGC | 1806 |
| --- | --- | --- |
|  | \|\|\|\|\|\|\|\|\|\| |  |
| Seq 2 884 | TTTTTTTTGC | 893 |

Alignment Length: 9; Identity: 9

| Seq 1 943 | AATAAAAAAA | 952 |
| --- | --- | --- |
|  | \|\|\|\|\|\|\|\|\|\| |  |
| Seq 2 411 | AATAAAAAAA | 420 |

Alignment Length: 9; Identity: 9

| Seq 1 1933 | ATTTTGAAA | 1941 |
| --- | --- | --- |
|  | \|\|\|\|\|\|\|\|\| |  |
| Seq 2 323 | ATTTTGAAA | 331 |

Alignment Length: 9; Identity: 9

| Seq 1 1032 | CAGAAAAAA | 1040 |
| --- | --- | --- |
|  | \|\|\|\|\|\|\|\|\| |  |
| Seq 2 838 | CAGAAAAAA | 846 |

Alignment Length: 9; Identity: 9

| Seq 1 2088 | AAAAATTTT | 2096 |
| --- | --- | --- |
|  | \|\|\|\|\|\|\|\|\| |  |
| Seq 2 371 | AAAAATTTT | 379 |

| Seq 1 1032 | CAGAAAAAA | 1040 |
| --- | --- | --- |
|  | \|\|\|\|\|\|\|\|\| |  |
| Seq 2 961 | CAGAAAAAA | 969 |

**S6 Figure, continued.** Motifs with identity between *C. elegans* and orthologous *unc-47* upstream sequences

Alignment Length: 9; Identity: 9

Alignment Length: 10; Identity: 10

| Seq 1 2221 | TTGAAAATT | 2229 | Seq 1 | 2741 | AAAAAAGAAA | 2732 |
| --- | --- | --- | --- | --- | --- | --- |
|  | \|\|\|\|\|\|\|\|\| |  |  |  | \|\|\|\|\|\|\|\|\|\| |  |
| Seq 2 157 | TTGAAAATT | 165 | Seq 2 | 842 | AAAAAAGAAA | 851 |

Alignment Length: 9; Identity: 9

Alignment Length: 9; Identity: 9

| Seq 1 2293 | CAGTTTTAA | 2301 | Seq 1 | 2738 | AAAGAAAAA | 2730 |
| --- | --- | --- | --- | --- | --- | --- |
|  | \|\|\|\|\|\|\|\|\| |  |  |  | \|\|\|\|\|\|\|\|\| |  |
| Seq 2 390 | CAGTTTTAA | 398 | Seq 2 | 358 | AAAGAAAAA | 366 |

Alignment Length: 9; Identity: 9

Alignment Length: 9; Identity: 9

| Seq 1 2307 | TTTATTTCA | 2315 | Seq 1 | 2736 | AGAAAAAAA | 2728 |
| --- | --- | --- | --- | --- | --- | --- |
|  | \|\|\|\|\|\|\|\|\| |  |  |  | \|\|\|\|\|\|\|\|\| |  |
| Seq 2 1177 | TTTATTTCA | 1185 | Seq 2 | 839 | AGAAAAAAA | 847 |

Alignment Length: 10; Identity: 10

Alignment Length: 9; Identity: 9

Seq 1 2733 AAAAAAAGA 2725

| Seq 1 2370 | TAAAGAAAAA | 2379 |  |  | \|\|\|\|\|\|\|\|\| |  |
| --- | --- | --- | --- | --- | --- | --- |
|  | \|\|\|\|\|\|\|\|\|\| |  | Seq 2 | 841 | AAAAAAAGA | 849 |
| Seq 2 357 | TAAAGAAAAA | 366 |  |  |  |  |

Alignment Length: 9; Identity: 9

Alignment Length: 10; Identity: 10

Seq 1 2687 TGCTCCATCT 2678

| Seq 1 2391 | GAGAGTAGG | 2399 |  |  | \|\|\|\|\|\|\|\|\|\| |  |
| --- | --- | --- | --- | --- | --- | --- |
|  | \|\|\|\|\|\|\|\|\| |  | Seq 2 | 1128 | TGCTCCATCT | 1137 |
| Seq 2 988 | GAGAGTAGG | 996 |  |  |  |  |

Alignment Length: 11; Identity: 11

Alignment Length: 9; Identity: 9

Seq 1 2663 AAATTTTCA 2655

| Seq 1 2443 | AATTGTGGTTT | 2453 |  |  | \|\|\|\|\|\|\|\|\| |  |
| --- | --- | --- | --- | --- | --- | --- |
|  | \|\|\|\|\|\|\|\|\|\|\| |  | Seq 2 | 373 | AAATTTTCA | 381 |
| Seq 2 244 | AATTGTGGTTT | 254 |  |  |  |  |

Alignment Length: 9; Identity: 9

Alignment Length: 9; Identity: 9

Seq 1 2663 AAATTTTCA 2655

| Seq 1 2655 | TGAAAATTT | 2663 |  |  | \|\|\|\|\|\|\|\|\| |  |
| --- | --- | --- | --- | --- | --- | --- |
|  | \|\|\|\|\|\|\|\|\| |  | Seq 2 | 617 | AAATTTTCA | 625 |
| Seq 2 158 | TGAAAATTT | 166 |  |  |  |  |

Alignment Length: 9; Identity: 9

Alignment Length: 10; Identity: 10

Seq 1 2516 TTGAAATACC 2507

| Seq 1 2657 | AAAATTTAA | 2665 |  |  | \|\|\|\|\|\|\|\|\|\| |  |
| --- | --- | --- | --- | --- | --- | --- |
|  | \|\|\|\|\|\|\|\|\| |  | Seq 2 | 123 | TTGAAATACC | 132 |
| Seq 2 790 | AAAATTTAA | 798 |  |  |  |  |

Alignment Length: 9; Identity: 9

Alignment Length: 9; Identity: 9

Seq 1 2469 AATTTTAAT 2461

| Seq 1 2798 | AATTGAAAA | 2806 |  |  | \|\|\|\|\|\|\|\|\| |  |
| --- | --- | --- | --- | --- | --- | --- |
|  | \|\|\|\|\|\|\|\|\| |  | Seq 2 | 653 | AATTTTAAT | 661 |
| Seq 2 155 | AATTGAAAA | 163 |  |  |  |  |

Alignment Length: 10; Identity: 10

Seq 1 2798 AATTGAAAAG 2807

||||||||||

Seq 2 524 AATTGAAAAG 533

**OPPOSITE STRAND**

Alignment Length: 9; Identity: 9

Seq 1 2806 TTTTCAATT 2798

|||||||||

Seq 2 620 TTTTCAATT 628

Alignment Length: 10; Identity: 10

Seq 1 2742 GAAAAAAGAA 2733

||||||||||

Seq 2 963 GAAAAAAGAA 972

Alignment Length: 9; Identity: 9

Seq 1 2403 TTTTCCTAC 2395

|||||||||

Seq 2 310 TTTTCCTAC 318

Alignment Length: 10; Identity: 10

Seq 1 2359 AGAAATAAAA 2350

||||||||||

Seq 2 770 AGAAATAAAA 779

Alignment Length: 9; Identity: 9

Seq 1 2356 AATAAAAAA 2348

|||||||||

Seq 2 411 AATAAAAAA 419

Alignment Length: 9; Identity: 9

Seq 1 2353 AAAAAATTT 2345

|||||||||

Seq 2 370 AAAAAATTT 378

**S6 Figure, continued.** Motifs with identity between *C. elegans* and orthologous *unc-47* upstream sequences

Alignment Length: 9; Identity: 9

Alignment Length: 9; Identity: 9

| Seq 1 2314 | GAAATAAAA | 2306 | Seq 1 | 1710 | TTTCCAATT | 1702 |
| --- | --- | --- | --- | --- | --- | --- |
|  | \|\|\|\|\|\|\|\|\| |  |  |  | \|\|\|\|\|\|\|\|\| |  |
| Seq 2 771 | GAAATAAAA | 779 | Seq 2 | 603 | TTTCCAATT | 611 |

Alignment Length: 9; Identity: 9

Alignment Length: 10; Identity: 10

| Seq 1 2229 | AATTTTCAA | 2221 | Seq 1 | 1685 | CCGAATTTGC | 1676 |
| --- | --- | --- | --- | --- | --- | --- |
|  | \|\|\|\|\|\|\|\|\| |  |  |  | \|\|\|\|\|\|\|\|\|\| |  |
| Seq 2 374 | AATTTTCAA | 382 | Seq 2 | 201 | CCGAATTTGC | 210 |

Alignment Length: 9; Identity: 9

Alignment Length: 11; Identity: 11

| Seq 1 2229 | AATTTTCAA | 2221 | Seq 1 | 1666 | TCGTTTTTTTT | 1656 |
| --- | --- | --- | --- | --- | --- | --- |
|  | \|\|\|\|\|\|\|\|\| |  |  |  | \|\|\|\|\|\|\|\|\|\|\| |  |
| Seq 2 618 | AATTTTCAA | 626 | Seq 2 | 881 | TCGTTTTTTTT | 891 |

Alignment Length: 9; Identity: 9

Alignment Length: 9; Identity: 9

| Seq 1 2103 | CGGAAAAAA | 2095 | Seq 1 | 1534 | CAAATTCTA | 1526 |
| --- | --- | --- | --- | --- | --- | --- |
|  | \|\|\|\|\|\|\|\|\| |  |  |  | \|\|\|\|\|\|\|\|\| |  |
| Seq 2 699 | CGGAAAAAA | 707 | Seq 2 | 338 | CAAATTCTA | 346 |

Alignment Length: 10; Identity: 10

Alignment Length: 9; Identity: 9

| Seq 1 2098 | AAAAAATTTT | 2089 | Seq 1 | 1152 | TTTTTTTTG | 1144 |
| --- | --- | --- | --- | --- | --- | --- |
|  | \|\|\|\|\|\|\|\|\|\| |  |  |  | \|\|\|\|\|\|\|\|\| |  |
| Seq 2 370 | AAAAAATTTT | 379 | Seq 2 | 884 | TTTTTTTTG | 892 |

Alignment Length: 10; Identity: 10

Alignment Length: 9; Identity: 9

| Seq 1 2072 | AAAAATTTTC | 2063 | Seq 1 | 1053 | AAAAAATTT | 1045 |
| --- | --- | --- | --- | --- | --- | --- |
|  | \|\|\|\|\|\|\|\|\|\| |  |  |  | \|\|\|\|\|\|\|\|\| |  |
| Seq 2 371 | AAAAATTTTC | 380 | Seq 2 | 370 | AAAAAATTT | 378 |

Alignment Length: 9; Identity: 9

| Seq 1 2061 | AAATAAAAT | 2053 |  |  | \|\|\|\|\|\|\|\|\| |  |
| --- | --- | --- | --- | --- | --- | --- |
|  | \|\|\|\|\|\|\|\|\| |  | Seq 2 | 883 | GTTTTTTTT | 891 |
| Seq 2 772 | AAATAAAAT | 780 |  |  |  |  |

Alignment Length: 9; Identity: 9

Seq 1 965 GTTTTTTTT 957

Alignment Length: 9; Identity: 9

| Seq 1 2040 | ATTGATGTT | 2032 |  |  | \|\|\|\|\|\|\|\|\| |  |
| --- | --- | --- | --- | --- | --- | --- |
|  | \|\|\|\|\|\|\|\|\| |  | Seq 2 | 790 | AAAATTTAA | 798 |
| Seq 2 443 | ATTGATGTT | 451 |  |  |  |  |

Alignment Length: 9; Identity: 9

Seq 1 910 AAAATTTAA 902

Alignment Length: 11; Identity: 11

| Seq 1 2013 | AAAAAAAGAAA | 2003 |  |  | \|\|\|\|\|\|\|\|\| |  |
| --- | --- | --- | --- | --- | --- | --- |
|  | \|\|\|\|\|\|\|\|\|\|\| |  | Seq 2 | 1119 | CACGAAATT | 1127 |
| Seq 2 841 | AAAAAAAGAAA | 851 |  |  |  |  |

Alignment Length: 9; Identity: 9

Seq 1 894 CACGAAATT 886

Alignment Length: 9; Identity: 9

| Seq 1 2012 | AAAAAAGAA | 2004 |  |  | \|\|\|\|\|\|\|\|\| |  |
| --- | --- | --- | --- | --- | --- | --- |
|  | \|\|\|\|\|\|\|\|\| |  | Seq 2 | 481 | AAATTACAG | 489 |
| Seq 2 964 | AAAAAAGAA | 972 |  |  |  |  |

Alignment Length: 9; Identity: 9

Seq 1 890 AAATTACAG 882

Alignment Length: 10; Identity: 10

| Seq 1 1972 | AGGGTCTAAT | 1963 |  |  | \|\|\|\|\|\|\|\|\| |  |
| --- | --- | --- | --- | --- | --- | --- |
|  | \|\|\|\|\|\|\|\|\|\| |  | Seq 2 | 735 | ACCTTGATT | 743 |
| Seq 2 994 | AGGGTCTAAT | 1003 |  |  |  |  |

Alignment Length: 9; Identity: 9

Seq 1 816 ACCTTGATT 808

Alignment Length: 9; Identity: 9

| Seq 1 1843 | GAAAAAAAG | 1835 |  |  | \|\|\|\|\|\|\|\|\|\|\|\| |  |
| --- | --- | --- | --- | --- | --- | --- |
|  | \|\|\|\|\|\|\|\|\| |  | Seq 2 | 364 | AAATGGAAAAAA | 375 |
| Seq 2 840 | GAAAAAAAG | 848 |  |  |  |  |

Alignment Length: 12; Identity: 12

Seq 1 701 AAATGGAAAAAA 690

Alignment Length: 9; Identity: 9

Alignment Length: 9; Identity: 9

Seq 1 668 TGAATTTTC 660

| Seq 1 1720 | AAATTTTAA | 1712 |  |  | \|\|\|\|\|\|\|\|\| |  |
| --- | --- | --- | --- | --- | --- | --- |
|  | \|\|\|\|\|\|\|\|\| |  | Seq 2 | 306 | TGAATTTTC | 314 |
| Seq 2 652 | AAATTTTAA | 660 |  |  |  |  |

**S6 Figure, continued.** Motifs with identity between *C. elegans* and orthologous *unc-47* upstream sequences

Alignment Length: 9; Identity: 9

| Seq 1 286 | AAATGAATC | 278 |
| --- | --- | --- |
|  | \|\|\|\|\|\|\|\|\| |  |
| Seq 2 777 | AAATGAATC | 785 |

Alignment Length: 9; Identity: 9

| Seq 1 200 | CCGAAAATT | 192 |
| --- | --- | --- |
|  | \|\|\|\|\|\|\|\|\| |  |
| Seq 2 477 | CCGAAAATT | 485 |

Alignment Length: 9; Identity: 9

| Seq 1 71 | ATTTTAATT | 63 |
| --- | --- | --- |
|  | \|\|\|\|\|\|\|\|\| |  |
| Seq 2 654 | ATTTTAATT | 662 |

Alignment Length: 9; Identity: 9

| Seq 1 32 | TTGAAAAGT | 24 |
| --- | --- | --- |
|  | \|\|\|\|\|\|\|\|\| |  |
| Seq 2 326 | TTGAAAAGT | 334 |

Alignment Length: 9; Identity: 9

| Seq 1 32 | TTGAAAAGT | 24 |
| --- | --- | --- |
|  | \|\|\|\|\|\|\|\|\| |  |
| Seq 2 526 | TTGAAAAGT | 534 |

**S6 Figure, continued.** Motifs with identity between *C. elegans* and orthologous *unc-47* upstream sequences

**T. spiralis/C. elegans 9bp window**

Alignment Length: 10; Identity: 10

|  | | Seq 1 | 1279 | AATTTCAATC | 1288 |
| --- | --- | --- | --- | --- | --- |
| **seq 1:** | **tspunc47** |  |  | \|\|\|\|\|\|\|\|\|\| |  |
| **seq 2:** | **celunc47** | Seq 2 | 276 | AATTTCAATC | 285 |

Alignment Length: 9; Identity: 9

Seq 1 63 AAAAGAAGA 71

|  | \|\|\|\|\|\|\|\|\| |  | Seq 1 | 1315 | CAAATTTCC | 1323 |
| --- | --- | --- | --- | --- | --- | --- |
| Seq 2 966 | AAAAGAAGA | 974 |  |  | \|\|\|\|\|\|\|\|\| |  |
|  |  |  | Seq 2 | 862 | CAAATTTCC | 870 |

Alignment Length: 9; Identity: 9

Alignment Length: 9; Identity: 9

Seq 1 190 TACCAATCC 198

|  | \|\|\|\|\|\|\|\|\| |  | Seq 1 | 1323 | CACATTTAT | 1331 |
| --- | --- | --- | --- | --- | --- | --- |
| Seq 2 941 | TACCAATCC | 949 |  |  | \|\|\|\|\|\|\|\|\| |  |
|  |  |  | Seq 2 | 930 | CACATTTAT | 938 |

Alignment Length: 9; Identity: 9

Alignment Length: 9; Identity: 9

Seq 1 316 AAAAAGAAA 324

|  | \|\|\|\|\|\|\|\|\| |  | Seq 1 | 1323 | CACATTTAT | 1331 |
| --- | --- | --- | --- | --- | --- | --- |
| Seq 2 843 | AAAAAGAAA | 851 |  |  | \|\|\|\|\|\|\|\|\| |  |
|  |  |  | Seq 2 | 1173 | CACATTTAT | 1181 |

Alignment Length: 9; Identity: 9

Alignment Length: 9; Identity: 9

Seq 1 377 AAAATGAAT 385

|  | \|\|\|\|\|\|\|\|\| |  | Seq 1 | 1366 | AATTGAAAA | 1374 |
| --- | --- | --- | --- | --- | --- | --- |
| Seq 2 776 | AAAATGAAT | 784 |  |  | \|\|\|\|\|\|\|\|\| |  |
|  |  |  | Seq 2 | 155 | AATTGAAAA | 163 |

Alignment Length: 9; Identity: 9

Alignment Length: 9; Identity: 9

Seq 1 405 AAAAAAATC 413

|  | \|\|\|\|\|\|\|\|\| |  | Seq 1 | 1366 | AATTGAAAA | 1374 |
| --- | --- | --- | --- | --- | --- | --- |
| Seq 2 414 | AAAAAAATC | 422 |  |  | \|\|\|\|\|\|\|\|\| |  |
|  |  |  | Seq 2 | 524 | AATTGAAAA | 532 |

Alignment Length: 9; Identity: 9

Alignment Length: 9; Identity: 9

Seq 1 814 AATTTTAAT 822

|  | \|\|\|\|\|\|\|\|\| |  | Seq 1 | 1501 | AAATTTTCA | 1509 |
| --- | --- | --- | --- | --- | --- | --- |
| Seq 2 653 | AATTTTAAT | 661 |  |  | \|\|\|\|\|\|\|\|\| |  |
|  |  |  | Seq 2 | 373 | AAATTTTCA | 381 |

Alignment Length: 9; Identity: 9

Alignment Length: 11; Identity: 11

Seq 1 848 AAATTTTCAAA 858

|  | \|\|\|\|\|\|\|\|\|\|\| |  | Seq 1 | 1501 | AAATTTTCA | 1509 |
| --- | --- | --- | --- | --- | --- | --- |
| Seq 2 373 | AAATTTTCAAA | 383 |  |  | \|\|\|\|\|\|\|\|\| |  |
|  |  |  | Seq 2 | 617 | AAATTTTCA | 625 |

Alignment Length: 9; Identity: 9

Alignment Length: 10; Identity: 10

Seq 1 848 AAATTTTCAA 857

|  | \|\|\|\|\|\|\|\|\|\| |  | Seq 1 | 1570 | AAAAATGGA | 1578 |
| --- | --- | --- | --- | --- | --- | --- |
| Seq 2 617 | AAATTTTCAA | 626 |  |  | \|\|\|\|\|\|\|\|\| |  |
|  |  |  | Seq 2 | 362 | AAAAATGGA | 370 |

Alignment Length: 9; Identity: 9

Alignment Length: 9; Identity: 9

Seq 1 1241 TTTAATTGA 1249

|  | \|\|\|\|\|\|\|\|\| |  | Seq 1 | 1619 | TTATTTCATT | 1628 |
| --- | --- | --- | --- | --- | --- | --- |
| Seq 2 794 | TTTAATTGA | 802 |  |  | \|\|\|\|\|\|\|\|\|\| |  |
|  |  |  | Seq 2 | 1178 | TTATTTCATT | 1187 |

Alignment Length: 10; Identity: 10

Alignment Length: 9; Identity: 9

Seq 1 1273 AAATAAAAT 1281

Alignment Length: 9; Identity: 9

|  | \|\|\|\|\|\|\|\|\| |  | Seq 1 | 1865 | ATTGAAAAT | 1873 |
| --- | --- | --- | --- | --- | --- | --- |
| Seq 2 772 | AAATAAAAT | 780 |  |  | \|\|\|\|\|\|\|\|\| |  |
|  |  |  | Seq 2 | 156 | ATTGAAAAT | 164 |

**S6 Figure, continued.** Motifs with identity between *C. elegans* and orthologous *unc-47* upstream sequences

**Opposite Strand**

Alignment Length: 9; Identity: 9

Seq 1 1873 ATTTTCAAT 1865

|||||||||

Seq 2 619 ATTTTCAAT 627

Alignment Length: 9; Identity: 9

Seq 1 1866 ATCTTTCCA 1858

|||||||||

Seq 2 1134 ATCTTTCCA 1142

Alignment Length: 9; Identity: 9

Seq 1 1509 TGAAAATTT 1501

|||||||||

Seq 2 158 TGAAAATTT 166

Alignment Length: 9; Identity: 9

Seq 1 1465 AAAAGAAAG 1457

|||||||||

Seq 2 844 AAAAGAAAG 852

Alignment Length: 9; Identity: 9

Seq 1 1440 AAATTTCAG 1432

|||||||||

Seq 2 267 AAATTTCAG 275

Alignment Length: 9; Identity: 9

Seq 1 1374 TTTTCAATT 1366

|||||||||

Seq 2 620 TTTTCAATT 628

Alignment Length: 9; Identity: 9

Seq 1 1324 TGGAAATTT 1316

|||||||||

Seq 2 264 TGGAAATTT 272

Alignment Length: 9; Identity: 9

Seq 1 1284 GAAATTTTA 1276

|||||||||

Seq 2 651 GAAATTTTA 659

Alignment Length: 10; Identity: 10

Seq 1 1225 CTTTTTGGAA 1216

||||||||||

Seq 2 259 CTTTTTGGAA 268

Alignment Length: 9; Identity: 9

Seq 1 1142 ACAAATCAG 1134

|||||||||

Seq 2 59 ACAAATCAG 67

Alignment Length: 11; Identity: 11

Seq 1 1042 TAATAAAAAAA 1032

[|||||||||||](#_TOC_250009)

Seq 2 410 TAATAAAAAAA 420

Alignment Length: 10; Identity: 10

Seq 1 857 TTGAAAATTT 848

[||||||||||](#_TOC_250008)

Seq 2 157 TTGAAAATTT 166

Alignment Length: 9; Identity: 9

Seq 1 854 AAAATTTAA 846

[|||||||||](#_TOC_250007)

Seq 2 790 AAAATTTAA 798

Alignment Length: 11; Identity: 11

Seq 1 775 ATATTGAAATA 765

[|||||||||||](#_TOC_250006)

Seq 2 120 ATATTGAAATA 130

Alignment Length: 9; Identity: 9

Seq 1 695 AAATTGCCA 687

[|||||||||](#_TOC_250005)

Seq 2 111 AAATTGCCA 119

Alignment Length: 10; Identity: 10

Seq 1 611 TTTCCTGTGA 602

[||||||||||](#_TOC_250004)

Seq 2 1152 TTTCCTGTGA 1161

Alignment Length: 9; Identity: 9

Seq 1 423 TTCACATTT 415

[|||||||||](#_TOC_250003)

Seq 2 928 TTCACATTT 936

Alignment Length: 9; Identity: 9

Seq 1 344 ACATTTATT 336

[|||||||||](#_TOC_250002)

Seq 2 931 ACATTTATT 939

Alignment Length: 9; Identity: 9

Seq 1 344 ACATTTATT 336

[|||||||||](#_TOC_250001)

Seq 2 1174 ACATTTATT 1182

Alignment Length: 9; Identity: 9

Seq 1 185 TGTGGTTTT 177

[|||||||||](#_TOC_250000)

Seq 2 247 TGTGGTTTT 255

Alignment Length: 9; Identity: 9

| Seq 1 181 | GTTTTTTTT | 173 |
| --- | --- | --- |
|  | \|\|\|\|\|\|\|\|\| |  |
| Seq 2 883 | GTTTTTTTT | 891 |

**S6 Figure, continued.** Motifs with identity between *C. elegans* and orthologous *unc-47* upstream sequences

Alignment Length: 9; Identity: 9

| Seq 1 159 | GTTTTTTTT | 151 |
| --- | --- | --- |
|  | \|\|\|\|\|\|\|\|\| |  |
| Seq 2 883 | GTTTTTTTT | 891 |
