# Supplementary material for: Phylum-Level Conservation of Regulatory Information in Nematodes despite Extensive Non-coding Sequence Divergence
Source: PLoS Genet. 2015 May 28;11(5):e1005268. doi: 10.1371/journal.pgen.1005268 (PMC4447282; doi:10.1371/journal.pgen.1005268)
Supplement: S7 Fig — Motifs with identity between C. elegans and orthologous unc-25 upstream sequences. All blocks of sequence identity in window sizes shown for each comparison with positions within the upstream non-coding sequence. (DOCX) [file pgen.1005268.s007.docx]

**S7 Figure. Motifs with identity between *C. elegans* and orthologous *unc-25* upstream sequences.** Motifs with identity between *C. elegans* and orthologous *unc-25* upstream sequences. All blocks of sequence identity in window sizes shown for each comparison with positions within the upstream non-coding sequence.

**C. briggsae/C. elegans unc-25, 10bp window**

**seq 1: cbrunc25**

**seq 2: celunc25**

Alignment Length: 10; Identity: 10

Alignment Length: 10; Identity: 10

| Seq 1 | 44 | ACAAAAACTA | 53 |
| --- | --- | --- | --- |
|  |  | \|\|\|\|\|\|\|\|\|\| |  |
| Seq 2 | 2153 | ACAAAAACTA | 2162 |

Alignment Length: 10; Identity: 10

| Seq 1 15 | CGAAATTCTT | 24 |
| --- | --- | --- |
|  | \|\|\|\|\|\|\|\|\|\| |  |
| Seq 2 1317 | CGAAATTCTT | 1326 |

Alignment Length: 10; Identity: 10

| Seq 1 | 73 | TCGATTTTCC | 82 |
| --- | --- | --- | --- |
|  |  | \|\|\|\|\|\|\|\|\|\| |  |
| Seq 2 | 502 | TCGATTTTCC | 511 |

Alignment Length: 12; Identity: 12

| Seq 1 24 | TAGTTTTTTT | 33 |
| --- | --- | --- |
|  | \|\|\|\|\|\|\|\|\|\| |  |
| Seq 2 462 | TAGTTTTTTT | 471 |

Alignment Length: 12; Identity: 12

| Seq 1 | 73 | TCGATTTTCCGG | 84 |
| --- | --- | --- | --- |
|  |  | \|\|\|\|\|\|\|\|\|\|\|\| |  |
| Seq 2 | 878 | TCGATTTTCCGG | 889 |

Alignment Length: 11; Identity: 11

| Seq 1 25 | AGTTTTTTTTTT | 36 |
| --- | --- | --- |
|  | \|\|\|\|\|\|\|\|\|\|\|\| |  |
| Seq 2 671 | AGTTTTTTTTTT | 682 |

Alignment Length: 10; Identity: 10

| Seq 1 | 76 | ATTTTCCGGCA | 86 |
| --- | --- | --- | --- |
|  |  | \|\|\|\|\|\|\|\|\|\|\| |  |
| Seq 2 | 2547 | ATTTTCCGGCA | 2557 |

Alignment Length: 10; Identity: 10

| Seq 1 27 | TTTTTTTTTT | 36 |
| --- | --- | --- |
|  | \|\|\|\|\|\|\|\|\|\| |  |
| Seq 2 483 | TTTTTTTTTT | 492 |

Alignment Length: 10; Identity: 10

| Seq 1 | 203 | CTTTTTGATC | 212 |
| --- | --- | --- | --- |
|  |  | \|\|\|\|\|\|\|\|\|\| |  |
| Seq 2 | 160 | CTTTTTGATC | 169 |

Alignment Length: 10; Identity: 10

| Seq 1 27 | TTTTTTTTTT | 36 |
| --- | --- | --- |
|  | \|\|\|\|\|\|\|\|\|\| |  |
| Seq 2 914 | TTTTTTTTTT | 923 |

Alignment Length: 12; Identity: 12

| Seq 1 | 271 | AAATTTTTTT | 280 |
| --- | --- | --- | --- |
|  |  | \|\|\|\|\|\|\|\|\|\| |  |
| Seq 2 | 547 | AAATTTTTTT | 556 |

Alignment Length: 10; Identity: 10

| Seq 1 28 | TTTTTTTTTTGA | 39 |
| --- | --- | --- |
|  | \|\|\|\|\|\|\|\|\|\|\|\| |  |
| Seq 2 483 | TTTTTTTTTTGA | 494 |

Alignment Length: 11; Identity: 11

| Seq 1 272 | AATTTTTTTC | 281 |
| --- | --- | --- |
|  | \|\|\|\|\|\|\|\|\|\| |  |
| Seq 2 809 | AATTTTTTTC | 818 |

Alignment Length: 10; Identity: 10

| Seq 1 28 | TTTTTTTTTTG | 38 |
| --- | --- | --- |
|  | \|\|\|\|\|\|\|\|\|\|\| |  |
| Seq 2 673 | TTTTTTTTTTG | 683 |

Alignment Length: 10; Identity: 10

| Seq 1 | 272 | AATTTTTTTC | 281 |
| --- | --- | --- | --- |
|  |  | \|\|\|\|\|\|\|\|\|\| |  |
| Seq 2 | 2055 | AATTTTTTTC | 2064 |

Alignment Length: 11; Identity: 11

| Seq 1 28 | TTTTTTTTTT | 37 |
| --- | --- | --- |
|  | \|\|\|\|\|\|\|\|\|\| |  |
| Seq 2 914 | TTTTTTTTTT | 923 |

Alignment Length: 10; Identity: 10

| Seq 1 | 303 | GAAATTTTTTT | 313 |
| --- | --- | --- | --- |
|  |  | \|\|\|\|\|\|\|\|\|\|\| |  |
| Seq 2 | 546 | GAAATTTTTTT | 556 |

Alignment Length: 11; Identity: 11

| Seq 1 29 | TTTTTTTTTG | 38 |
| --- | --- | --- |
|  | \|\|\|\|\|\|\|\|\|\| |  |
| Seq 2 1595 | TTTTTTTTTG | 1604 |

Alignment Length: 10; Identity: 10

| Seq 1 | 355 | AAAATCAAAAA | 365 |
| --- | --- | --- | --- |
|  |  | \|\|\|\|\|\|\|\|\|\|\| |  |
| Seq 2 | 417 | AAAATCAAAAA | 427 |

Alignment Length: 10; Identity: 10

| Seq 1 30 | TTTTTTTTGA | 39 |
| --- | --- | --- |
|  | \|\|\|\|\|\|\|\|\|\| |  |
| Seq 2 550 | TTTTTTTTGA | 559 |

Alignment Length: 11; Identity: 11

| Seq 1 360 | CAAAAATCCC | 369 |
| --- | --- | --- |
|  | \|\|\|\|\|\|\|\|\|\| |  |
| Seq 2 1453 | CAAAAATCCC | 1462 |

Alignment Length: 10; Identity: 10

| Seq 1 33 | TTTTTGAAATT | 43 |
| --- | --- | --- |
|  | \|\|\|\|\|\|\|\|\|\|\| |  |
| Seq 2 1084 | TTTTTGAAATT | 1094 |

Alignment Length: 10; Identity: 10

| Seq 1 | 490 | AATAGTTTTT | 499 |
| --- | --- | --- | --- |
|  |  | \|\|\|\|\|\|\|\|\|\| |  |
| Seq 2 | 2125 | AATAGTTTTT | 2134 |

Seq 1 34 TTTTGAAATT 43

||||||||||

Seq 2 2323 TTTTGAAATT 2332

**S7 Figure, continued.** Motifs with identity between *C. elegans* and orthologous *unc-25* upstream sequences.

Alignment Length: 10; Identity: 10

Alignment Length: 10; Identity: 10

| Seq 1 517 | TTTTTTGACT | 526 | Seq 1 | 600 | TTTTTTTTTC | 609 |
| --- | --- | --- | --- | --- | --- | --- |
|  | \|\|\|\|\|\|\|\|\|\| |  |  |  | \|\|\|\|\|\|\|\|\|\| |  |
| Seq 2 1520 | TTTTTTGACT | 1529 | Seq 2 | 95 | TTTTTTTTTC | 104 |

Alignment Length: 11; Identity: 11

Alignment Length: 11; Identity: 11

| Seq 1 583 | GCCAAATTTTT | 593 |
| --- | --- | --- |
|  | \|\|\|\|\|\|\|\|\|\|\| |  |
| Seq 2 2657 | GCCAAATTTTT | 2667 |

Alignment Length: 11; Identity: 11

| Seq 1 601 | TTTTTTTTCAA | 611 |
| --- | --- | --- |
|  | \|\|\|\|\|\|\|\|\|\|\| |  |
| Seq 2 1608 | TTTTTTTTCAA | 1618 |

Alignment Length: 10; Identity: 10

| Seq 1 586 | AAATTTTTTTT | 596 |
| --- | --- | --- |
|  | \|\|\|\|\|\|\|\|\|\|\| |  |
| Seq 2 547 | AAATTTTTTTT | 557 |

Alignment Length: 11; Identity: 11

| Seq 1 686 | ACGTCTTCTT | 695 |
| --- | --- | --- |
|  | \|\|\|\|\|\|\|\|\|\| |  |
| Seq 2 302 | ACGTCTTCTT | 311 |

Alignment Length: 10; Identity: 10

| Seq 1 587 | AATTTTTTTTT | 597 |
| --- | --- | --- |
|  | \|\|\|\|\|\|\|\|\|\|\| |  |
| Seq 2 93 | AATTTTTTTTT | 103 |

Alignment Length: 10; Identity: 10

| Seq 1 750 | ATTTTTCAGA | 759 |
| --- | --- | --- |
|  | \|\|\|\|\|\|\|\|\|\| |  |
| Seq 2 384 | ATTTTTCAGA | 393 |

Alignment Length: 10; Identity: 10

| Seq 1 587 | AATTTTTTTT | 596 |
| --- | --- | --- |
|  | \|\|\|\|\|\|\|\|\|\| |  |
| Seq 2 1396 | AATTTTTTTT | 1405 |

Alignment Length: 10; Identity: 10

| Seq 1 751 | TTTTTCAGAA | 760 |
| --- | --- | --- |
|  | \|\|\|\|\|\|\|\|\|\| |  |
| Seq 2 919 | TTTTTCAGAA | 928 |

Alignment Length: 11; Identity: 11

| Seq 1 588 | ATTTTTTTTT | 597 |
| --- | --- | --- |
|  | \|\|\|\|\|\|\|\|\|\| |  |
| Seq 2 482 | ATTTTTTTTT | 491 |

Alignment Length: 10; Identity: 10

| Seq 1 806 | TTCCTTTTTTT | 816 |
| --- | --- | --- |
|  | \|\|\|\|\|\|\|\|\|\|\| |  |
| Seq 2 1376 | TTCCTTTTTTT | 1386 |

Alignment Length: 16; Identity: 16

| Seq 1 588 | ATTTTTTTTT | 597 |
| --- | --- | --- |
|  | \|\|\|\|\|\|\|\|\|\| |  |
| Seq 2 913 | ATTTTTTTTT | 922 |

Alignment Length: 10; Identity: 10

| Seq 1 939 | GAGCCACAGTAATCCT | 954 |
| --- | --- | --- |
| Seq 2 1885 | \|\|\|\|\|\|\|\|\|\|\|\|\|\|\|\| GAGCCACAGTAATCCT | 1900 |

Alignment Length: 10; Identity: 10

| Seq 1 589 | TTTTTTTTTG | 598 |
| --- | --- | --- |
|  | \|\|\|\|\|\|\|\|\|\| |  |
| Seq 2 484 | TTTTTTTTTG | 493 |

| Seq 1 1007 | CAGTTTTCCC | 1016 |
| --- | --- | --- |
|  | \|\|\|\|\|\|\|\|\|\| |  |
| Seq 2 1463 | CAGTTTTCCC | 1472 |

Alignment Length: 10; Identity: 10

Seq 1 589 TTTTTTTTTG 598

||||||||||

Seq 2 674 TTTTTTTTTG 683

Alignment Length: 10; Identity: 10

**OPPOSITE STRAND**

Alignment Length: 10; Identity: 10

| Seq 1 | 823 | AAAACCCAAA | 814 |
| --- | --- | --- | --- |
|  |  | \|\|\|\|\|\|\|\|\|\| |  |
| Seq 2 | 764 | AAAACCCAAA | 773 |

Alignment Length: 10; Identity: 10

| Seq 1 589 | TTTTTTTTTG | 598 |
| --- | --- | --- |
|  | \|\|\|\|\|\|\|\|\|\| |  |
| Seq 2 1595 | TTTTTTTTTG | 1604 |

Alignment Length: 11; Identity: 11

| Seq 1 | 620 | CCAAAAATTT | 611 |
| --- | --- | --- | --- |
|  |  | \|\|\|\|\|\|\|\|\|\| |  |
| Seq 2 | 104 | CCAAAAATTT | 113 |

Alignment Length: 11; Identity: 11

| Seq 1 598 | GTTTTTTTTTT | 608 |
| --- | --- | --- |
|  | \|\|\|\|\|\|\|\|\|\|\| |  |
| Seq 2 672 | GTTTTTTTTTT | 682 |

Alignment Length: 10; Identity: 10

| Seq 1 617 | AAAATTTTGAA | 607 |
| --- | --- | --- |
|  | \|\|\|\|\|\|\|\|\|\|\| |  |
| Seq 2 605 | AAAATTTTGAA | 615 |

Alignment Length: 10; Identity: 10

| Seq 1 599 | TTTTTTTTTT | 608 |
| --- | --- | --- |
|  | \|\|\|\|\|\|\|\|\|\| |  |
| Seq 2 483 | TTTTTTTTTT | 492 |

Alignment Length: 12; Identity: 12

| Seq 1 | 614 | ATTTTGAAAA | 605 |
| --- | --- | --- | --- |
|  |  | \|\|\|\|\|\|\|\|\|\| |  |
| Seq 2 | 1293 | ATTTTGAAAA | 1302 |

Seq 1 599 TTTTTTTTTTCA 610

||||||||||||

Seq 2 914 TTTTTTTTTTCA 925

**S7 Figure, continued.** Motifs with identity between *C. elegans* and orthologous *unc-25* upstream sequences.

Alignment Length: 11; Identity: 11

Alignment Length: 10; Identity: 10

| Seq 1 609 | GAAAAAAAAAA | 599 | Seq 1 | 282 | AGAAAAAAAT | 273 |
| --- | --- | --- | --- | --- | --- | --- |
|  | \|\|\|\|\|\|\|\|\|\|\| |  |  |  | \|\|\|\|\|\|\|\|\|\| |  |
| Seq 2 410 | GAAAAAAAAAA | 420 | Seq 2 | 1027 | AGAAAAAAAT | 1036 |

Alignment Length: 10; Identity: 10

Alignment Length: 10; Identity: 10

| Seq 1 609 | GAAAAAAAAA | 600 | Seq 1 | 231 | TTTCTGAGAA | 222 |
| --- | --- | --- | --- | --- | --- | --- |
|  | \|\|\|\|\|\|\|\|\|\| |  |  |  | \|\|\|\|\|\|\|\|\|\| |  |
| Seq 2 758 | GAAAAAAAAA | 767 | Seq 2 | 733 | TTTCTGAGAA | 742 |

Alignment Length: 10; Identity: 10

Alignment Length: 10; Identity: 10

| Seq 1 608 | AAAAAAAAAA | 599 | Seq 1 | 112 | GCATTTTTCA | 103 |
| --- | --- | --- | --- | --- | --- | --- |
|  | \|\|\|\|\|\|\|\|\|\| |  |  |  | \|\|\|\|\|\|\|\|\|\| |  |
| Seq 2 512 | AAAAAAAAAA | 521 | Seq 2 | 269 | GCATTTTTCA | 278 |

Alignment Length: 10; Identity: 10

Alignment Length: 10; Identity: 10

| Seq 1 607 | AAAAAAAAAC | 598 | Seq 1 | 110 | ATTTTTCAGA | 101 |
| --- | --- | --- | --- | --- | --- | --- |
|  | \|\|\|\|\|\|\|\|\|\| |  |  |  | \|\|\|\|\|\|\|\|\|\| |  |
| Seq 2 759 | AAAAAAAAAC | 768 | Seq 2 | 384 | ATTTTTCAGA | 393 |

Alignment Length: 10; Identity: 10

Alignment Length: 12; Identity: 12

| Seq 1 598 | CAAAAAAAAA | 589 | Seq 1 | 109 | TTTTTCAGAAAA | 98 |
| --- | --- | --- | --- | --- | --- | --- |
|  | \|\|\|\|\|\|\|\|\|\| |  |  |  | \|\|\|\|\|\|\|\|\|\|\|\| |  |
| Seq 2 511 | CAAAAAAAAA | 520 | Seq 2 | 919 | TTTTTCAGAAAA | 930 |

Alignment Length: 10; Identity: 10

Alignment Length: 10; Identity: 10

| Seq 1 597 | AAAAAAAAAT | 588 | Seq 1 | 107 | TTTCAGAAAA | 98 |
| --- | --- | --- | --- | --- | --- | --- |
|  | \|\|\|\|\|\|\|\|\|\| |  |  |  | \|\|\|\|\|\|\|\|\|\| |  |
| Seq 2 412 | AAAAAAAAAT | 421 | Seq 2 | 1093 | TTTCAGAAAA | 1102 |

Alignment Length: 10; Identity: 10

Alignment Length: 10; Identity: 10

| Seq 1 597 | AAAAAAAAAT | 588 | Seq 1 | 39 | TCAAAAAAAA | 30 |
| --- | --- | --- | --- | --- | --- | --- |
|  | \|\|\|\|\|\|\|\|\|\| |  |  |  | \|\|\|\|\|\|\|\|\|\| |  |
| Seq 2 513 | AAAAAAAAAT | 522 | Seq 2 | 858 | TCAAAAAAAA | 867 |

Alignment Length: 10; Identity: 10

Alignment Length: 11; Identity: 11

| Seq 1 500 | CAAAAACTAT | 491 | Seq 1 | 38 | CAAAAAAAAAA | 28 |
| --- | --- | --- | --- | --- | --- | --- |
|  | \|\|\|\|\|\|\|\|\|\| |  |  |  | \|\|\|\|\|\|\|\|\|\|\| |  |
| Seq 2 2154 | CAAAAACTAT | 2163 | Seq 2 | 511 | CAAAAAAAAAA | 521 |

Alignment Length: 10; Identity: 10

Alignment Length: 10; Identity: 10

| Seq 1 390 | TGCCGGAAAT | 381 | Seq 1 | 37 | AAAAAAAAAA | 28 |
| --- | --- | --- | --- | --- | --- | --- |
|  | \|\|\|\|\|\|\|\|\|\| |  |  |  | \|\|\|\|\|\|\|\|\|\| |  |
| Seq 2 2578 | TGCCGGAAAT | 2587 | Seq 2 | 411 | AAAAAAAAAA | 420 |

Alignment Length: 10; Identity: 10

Alignment Length: 10; Identity: 10

| Seq 1 390 | TGCCGGAAAT | 381 | Seq 1 | 36 | AAAAAAAAAA | 27 |
| --- | --- | --- | --- | --- | --- | --- |
|  | \|\|\|\|\|\|\|\|\|\| |  |  |  | \|\|\|\|\|\|\|\|\|\| |  |
| Seq 2 2768 | TGCCGGAAAT | 2777 | Seq 2 | 411 | AAAAAAAAAA | 420 |

Alignment Length: 10; Identity: 10

Alignment Length: 10; Identity: 10

| Seq 1 390 | TGCCGGAAAT | 381 | Seq 1 | 36 | AAAAAAAAAA | 27 |
| --- | --- | --- | --- | --- | --- | --- |
|  | \|\|\|\|\|\|\|\|\|\| |  |  |  | \|\|\|\|\|\|\|\|\|\| |  |
| Seq 2 2854 | TGCCGGAAAT | 2863 | Seq 2 | 512 | AAAAAAAAAA | 521 |

Alignment Length: 10; Identity: 10

Alignment Length: 10; Identity: 10

| Seq 1 366 | ATTTTTGATT | 357 | Seq 1 35 | AAAAAAAAAC | 26 |
| --- | --- | --- | --- | --- | --- |
|  | \|\|\|\|\|\|\|\|\|\| |  |  | \|\|\|\|\|\|\|\|\|\| |  |
| Seq 2 2461 | ATTTTTGATT | 2470 | Seq 2 759 | AAAAAAAAAC | 768 |

Alignment Length: 12; Identity: 12

| Seq 1 309 | AAATTTCAGAAA | 298 |
| --- | --- | --- |
|  | \|\|\|\|\|\|\|\|\|\|\|\| |  |
| Seq 2 1090 | AAATTTCAGAAA | 1101 |

**S7 Figure, continued.** Motifs with identity between *C. elegans* and orthologous *unc-25* upstream sequences.

**M. hapla/C. elegans unc-25, 10bp window**

**seq 1: mhaunc25**

**seq 2: celunc25**

Alignment Length: 10; Identity: 10

Alignment Length: 11; Identity: 11

| Seq 1 1030 | AATATTTTTTT | 1040 |
| --- | --- | --- |
|  | \|\|\|\|\|\|\|\|\|\|\| |  |
| Seq 2 910 | AATATTTTTTT | 920 |

Alignment Length: 10; Identity: 10

| Seq 1 199 | AATTTTTATC | 208 |
| --- | --- | --- |
|  | \|\|\|\|\|\|\|\|\|\| |  |
| Seq 2 2104 | AATTTTTATC | 2113 |

Alignment Length: 10; Identity: 10

| Seq 1 1034 | TTTTTTTAAA | 1043 |
| --- | --- | --- |
|  | \|\|\|\|\|\|\|\|\|\| |  |
| Seq 2 1325 | TTTTTTTAAA | 1334 |

Alignment Length: 10; Identity: 10

| Seq 1 294 | TTTAAATTTC | 303 |
| --- | --- | --- |
|  | \|\|\|\|\|\|\|\|\|\| |  |
| Seq 2 111 | TTTAAATTTC | 120 |

Alignment Length: 10; Identity: 10

| Seq 1 1034 | TTTTTTTAAA | 1043 |
| --- | --- | --- |
|  | \|\|\|\|\|\|\|\|\|\| |  |
| Seq 2 1399 | TTTTTTTAAA | 1408 |

Alignment Length: 10; Identity: 10

| Seq 1 520 | AAAATTTAAA | 529 |
| --- | --- | --- |
|  | \|\|\|\|\|\|\|\|\|\| |  |
| Seq 2 107 | AAAATTTAAA | 116 |

Alignment Length: 10; Identity: 10

| Seq 1 1034 | TTTTTTTAAA | 1043 |
| --- | --- | --- |
|  | \|\|\|\|\|\|\|\|\|\| |  |
| Seq 2 1530 | TTTTTTTAAA | 1539 |

| Seq 1 524 | TTTAAAATTC | 533 |
| --- | --- | --- |
|  | \|\|\|\|\|\|\|\|\|\| |  |
| Seq 2 1403 | TTTAAAATTC | 1412 |

Alignment Length: 10; Identity: 10

| Seq 1 531 | TTCAATTTTT | 540 |
| --- | --- | --- |
|  | \|\|\|\|\|\|\|\|\|\| |  |
| Seq 2 1393 | TTCAATTTTT | 1402 |

Alignment Length: 13; Identity: 13

Seq 1 1071 GTTTGGAATTTTT 1083

|||||||||||||

Seq 2 2098 GTTTGGAATTTTT 2110

Alignment Length: 10; Identity: 10

Alignment Length: 10; Identity: 10

| Seq 1 1074 | TGGAATTTTT | 1083 |
| --- | --- | --- |
|  | \|\|\|\|\|\|\|\|\|\| |  |
| Seq 2 2693 | TGGAATTTTT | 2702 |

Alignment Length: 11; Identity: 11

| Seq 1 690 | ATTTTTTGGT | 699 |
| --- | --- | --- |
|  | \|\|\|\|\|\|\|\|\|\| |  |
| Seq 2 432 | ATTTTTTGGT | 441 |

Alignment Length: 10; Identity: 10

| Seq 1 1075 | GGAATTTTTTA | 1085 |
| --- | --- | --- |
|  | \|\|\|\|\|\|\|\|\|\|\| |  |
| Seq 2 829 | GGAATTTTTTA | 839 |

Alignment Length: 10; Identity: 10

| Seq 1 748 | TAATTTTTTT | 757 |
| --- | --- | --- |
|  | \|\|\|\|\|\|\|\|\|\| |  |
| Seq 2 92 | TAATTTTTTT | 101 |

Alignment Length: 10; Identity: 10

| Seq 1 1078 | ATTTTTTAAA | 1087 |
| --- | --- | --- |
|  | \|\|\|\|\|\|\|\|\|\| |  |
| Seq 2 2115 | ATTTTTTAAA | 2124 |

Alignment Length: 12; Identity: 12

| Seq 1 748 | TAATTTTTTT | 757 |
| --- | --- | --- |
|  | \|\|\|\|\|\|\|\|\|\| |  |
| Seq 2 808 | TAATTTTTTT | 817 |

Alignment Length: 13; Identity: 13

| Seq 1 1095 | TTATTTTTTTTC | 1106 |
| --- | --- | --- |
|  | \|\|\|\|\|\|\|\|\|\|\|\| |  |
| Seq 2 1579 | TTATTTTTTTTC | 1590 |

Alignment Length: 10; Identity: 10

| Seq 1 790 | AAAAATATTTTTT | 802 |
| --- | --- | --- |
|  | \|\|\|\|\|\|\|\|\|\|\|\|\| |  |
| Seq 2 907 | AAAAATATTTTTT | 919 |

Alignment Length: 10; Identity: 10

| Seq 1 1096 | TATTTTTTTT | 1105 |
| --- | --- | --- |
|  | \|\|\|\|\|\|\|\|\|\| |  |
| Seq 2 912 | TATTTTTTTT | 921 |

Alignment Length: 12; Identity: 12

| Seq 1 799 | TTTTAATTTT | 808 |
| --- | --- | --- |
|  | \|\|\|\|\|\|\|\|\|\| |  |
| Seq 2 2933 | TTTTAATTTT | 2942 |

Alignment Length: 12; Identity: 12

| Seq 1 1098 | TTTTTTTTCCAA | 1109 |
| --- | --- | --- |
|  | \|\|\|\|\|\|\|\|\|\|\|\| |  |
| Seq 2 96 | TTTTTTTTCCAA | 107 |

Alignment Length: 10; Identity: 10

| Seq 1 802 | TAATTTTTAGAT | 813 |
| --- | --- | --- |
|  | \|\|\|\|\|\|\|\|\|\|\|\| |  |
| Seq 2 995 | TAATTTTTAGAT | 1006 |

Alignment Length: 10; Identity: 10

| Seq 1 1177 | TAAAGTTTTT | 1186 |
| --- | --- | --- |
|  | \|\|\|\|\|\|\|\|\|\| |  |
| Seq 2 668 | TAAAGTTTTT | 677 |

Alignment Length: 11; Identity: 11

| Seq 1 1028 | ATAATATTTT | 1037 |
| --- | --- | --- |
|  | \|\|\|\|\|\|\|\|\|\| |  |
| Seq 2 1288 | ATAATATTTT | 1297 |

Seq 1 1208 TGATTTTTCCC 1218

|||||||||||

Seq 2 1494 TGATTTTTCCC 1504

**S7 Figure, continued.** Motifs with identity between *C. elegans* and orthologous *unc-25* upstream sequences.

Alignment Length: 11; Identity: 11

Alignment Length: 10; Identity: 10

| Seq 1 1278 | TTTCTTTTTTT | 1288 | Seq 1 | 1704 | AATCAAAAAA | 1713 |
| --- | --- | --- | --- | --- | --- | --- |
|  | \|\|\|\|\|\|\|\|\|\|\| |  |  |  | \|\|\|\|\|\|\|\|\|\| |  |
| Seq 2 1591 | TTTCTTTTTTT | 1601 | Seq 2 | 419 | AATCAAAAAA | 428 |

Alignment Length: 10; Identity: 10

Alignment Length: 10; Identity: 10

| Seq 1 1279 | TTCTTTTTTT | 1288 | Seq 1 | 1706 | TCAAAAAAAA | 1715 |
| --- | --- | --- | --- | --- | --- | --- |
|  | \|\|\|\|\|\|\|\|\|\| |  |  |  | \|\|\|\|\|\|\|\|\|\| |  |
| Seq 2 1322 | TTCTTTTTTT | 1331 | Seq 2 | 858 | TCAAAAAAAA | 867 |

Alignment Length: 10; Identity: 10

Alignment Length: 11; Identity: 11

Seq 1 1711 AAAAATTTAAA 1721

| Seq 1 1282 | TTTTTTTGAG | 1291 |  |  | \|\|\|\|\|\|\|\|\|\|\| |  |
| --- | --- | --- | --- | --- | --- | --- |
|  | \|\|\|\|\|\|\|\|\|\| |  | Seq 2 | 106 | AAAAATTTAAA | 116 |
| Seq 2 486 | TTTTTTTGAG | 495 |  |  |  |  |

Alignment Length: 10; Identity: 10

Alignment Length: 10; Identity: 10

Seq 1 1799 TTTTTTCTTT 1808

| Seq 1 1282 | TTTTTTTGAG | 1291 |  |  | \|\|\|\|\|\|\|\|\|\| |  |
| --- | --- | --- | --- | --- | --- | --- |
|  | \|\|\|\|\|\|\|\|\|\| |  | Seq 2 | 1584 | TTTTTTCTTT | 1593 |
| Seq 2 551 | TTTTTTTGAG | 560 |  |  |  |  |

Alignment Length: 10; Identity: 10

Alignment Length: 10; Identity: 10

Seq 1 1883 TAAAAAATTA 1892

| Seq 1 1320 | ATTTTTCCCC | 1329 |  |  | \|\|\|\|\|\|\|\|\|\| |  |
| --- | --- | --- | --- | --- | --- | --- |
|  | \|\|\|\|\|\|\|\|\|\| |  | Seq 2 | 636 | TAAAAAATTA | 645 |
| Seq 2 1496 | ATTTTTCCCC | 1505 |  |  |  |  |

Alignment Length: 13; Identity: 13

Alignment Length: 10; Identity: 10

Seq 1 1901 AAAAATTTAA 1910

| Seq 1 1460 | TAAAAAAATTAAA | 1472 |  |  | \|\|\|\|\|\|\|\|\|\| |  |
| --- | --- | --- | --- | --- | --- | --- |
|  | \|\|\|\|\|\|\|\|\|\|\|\|\| |  | Seq 2 | 106 | AAAAATTTAA | 115 |
| Seq 2 2477 | TAAAAAAATTAAA | 2489 |  |  |  |  |

Alignment Length: 10; Identity: 10

Alignment Length: 10; Identity: 10

Seq 1 1932 ATTTTTTGGT 1941

| Seq 1 1507 | GAAAAAAAAA | 1516 |  |  | \|\|\|\|\|\|\|\|\|\| |  |
| --- | --- | --- | --- | --- | --- | --- |
|  | \|\|\|\|\|\|\|\|\|\| |  | Seq 2 | 432 | ATTTTTTGGT | 441 |
| Seq 2 410 | GAAAAAAAAA | 419 |  |  |  |  |

Alignment Length: 10; Identity: 10

Alignment Length: 12; Identity: 12

Seq 1 1933 TTTTTTGGTTTT 1944

| Seq 1 1507 | GAAAAAAAAA | 1516 |  |  | \|\|\|\|\|\|\|\|\|\|\|\| |  |
| --- | --- | --- | --- | --- | --- | --- |
|  | \|\|\|\|\|\|\|\|\|\| |  | Seq 2 | 1382 | TTTTTTGGTTTT | 1393 |
| Seq 2 758 | GAAAAAAAAA | 767 |  |  |  |  |

Alignment Length: 11; Identity: 11

Alignment Length: 14; Identity: 14

Seq 1 1971 AAAAATATTTTTTT 1984

| Seq 1 1531 | TTTGACTTTTT | 1541 |  |  | \|\|\|\|\|\|\|\|\|\|\|\|\|\| |
| --- | --- | --- | --- | --- | --- |
|  | \|\|\|\|\|\|\|\|\|\|\| |  | Seq 2 | 907 | AAAAATATTTTTTT 920 |
| Seq 2 1523 | TTTGACTTTTT | 1533 |  |  |  |

Alignment Length: 12; Identity: 12

Alignment Length: 10; Identity: 10

Seq 1 1977 ATTTTTTTGA 1986

| Seq 1 1566 | ATTGCATTGATT | 1577 |  |  | \|\|\|\|\|\|\|\|\|\| |  |
| --- | --- | --- | --- | --- | --- | --- |
|  | \|\|\|\|\|\|\|\|\|\|\|\| |  | Seq 2 | 1518 | ATTTTTTTGA | 1527 |
| Seq 2 2137 | ATTGCATTGATT | 2148 |  |  |  |  |

Alignment Length: 10; Identity: 10

Alignment Length: 10; Identity: 10

Seq 1 2028 ATTTTTTAAA 2037

| Seq 1 1585 | AATACTAAAA | 1594 |  |  | \|\|\|\|\|\|\|\|\|\| |  |
| --- | --- | --- | --- | --- | --- | --- |
|  | \|\|\|\|\|\|\|\|\|\| |  | Seq 2 | 2115 | ATTTTTTAAA | 2124 |
| Seq 2 599 | AATACTAAAA | 608 |  |  |  |  |

Alignment Length: 10; Identity: 10

Alignment Length: 10; Identity: 10

Seq 1 2251 ATTTTTGAAA 2260

| Seq 1 1693 | AAATTTATAA | 1702 |  |  | \|\|\|\|\|\|\|\|\|\| |  |
| --- | --- | --- | --- | --- | --- | --- |
|  | \|\|\|\|\|\|\|\|\|\| |  | Seq 2 | 1083 | ATTTTTGAAA | 1092 |
| Seq 2 943 | AAATTTATAA | 952 |  |  |  |  |

Alignment Length: 10; Identity: 10

Seq 1 1698 TATAATAATC 1707

||||||||||

Seq 2 2987 TATAATAATC 2996

Alignment Length: 11; Identity: 11

Seq 1 2264 AAAATCTTTTT 2274

|||||||||||

Seq 2 894 AAAATCTTTTT 904

**S7 Figure, continued.** Motifs with identity between *C. elegans* and orthologous *unc-25* upstream sequences.

Alignment Length: 12; Identity: 12

| Seq 1 2307 | TTTTCAGAAAAT | 2318 | Seq 1 | 2568 | TTTTTAAAAT | 2577 |
| --- | --- | --- | --- | --- | --- | --- |
|  | \|\|\|\|\|\|\|\|\|\|\|\| |  |  |  | \|\|\|\|\|\|\|\|\|\| |  |
| Seq 2 920 | TTTTCAGAAAAT | 931 | Seq 2 | 1532 | TTTTTAAAAT | 1541 |

Alignment Length: 10; Identity: 10

Alignment Length: 11; Identity: 11

| Seq 1 2308 | TTTCAGAAAAT | 2318 | Seq 1 | 2648 | GGAATTTTTTA | 2658 |
| --- | --- | --- | --- | --- | --- | --- |
|  | \|\|\|\|\|\|\|\|\|\|\| |  |  |  | \|\|\|\|\|\|\|\|\|\|\| |  |
| Seq 2 1093 | TTTCAGAAAAT | 1103 | Seq 2 | 829 | GGAATTTTTTA | 839 |

Alignment Length: 11; Identity: 11

Alignment Length: 10; Identity: 10

| Seq 1 2322 | TTTAAAAAAT | 2331 | Seq 1 | 2757 | CAGAAAATCG | 2766 |
| --- | --- | --- | --- | --- | --- | --- |
|  | \|\|\|\|\|\|\|\|\|\| |  |  |  | \|\|\|\|\|\|\|\|\|\| |  |
| Seq 2 1329 | TTTAAAAAAT | 1338 | Seq 2 | 1096 | CAGAAAATCG | 1105 |

Alignment Length: 10; Identity: 10

Alignment Length: 13; Identity: 13

| Seq 1 2324 | TAAAAAATCTTCT | 2336 | Seq 1 | 2798 | TCGTCACGAA | 2807 |
| --- | --- | --- | --- | --- | --- | --- |
|  | \|\|\|\|\|\|\|\|\|\|\|\|\| |  |  |  | \|\|\|\|\|\|\|\|\|\| |  |
| Seq 2 2379 | TAAAAAATCTTCT | 2391 | Seq 2 | 1706 | TCGTCACGAA | 1715 |

Alignment Length: 10; Identity: 10

Alignment Length: 11; Identity: 11

| Seq 1 2325 | AAAAAATCTTC | 2335 | Seq 1 | 2969 | TATTTTTATTA | 2979 |
| --- | --- | --- | --- | --- | --- | --- |
|  | \|\|\|\|\|\|\|\|\|\|\| |  |  |  | \|\|\|\|\|\|\|\|\|\|\| |  |
| Seq 2 227 | AAAAAATCTTC | 237 | Seq 2 | 2353 | TATTTTTATTA | 2363 |

Alignment Length: 11; Identity: 11

Alignment Length: 10; Identity: 10

| Seq 1 2325 | AAAAAATCTT | 2334 | Seq 1 | 2978 | TAATTTTTGA | 2987 |
| --- | --- | --- | --- | --- | --- | --- |
|  | \|\|\|\|\|\|\|\|\|\| |  |  |  | \|\|\|\|\|\|\|\|\|\| |  |
| Seq 2 892 | AAAAAATCTT | 901 | Seq 2 | 2459 | TAATTTTTGA | 2468 |

Alignment Length: 10; Identity: 10

Alignment Length: 11; Identity: 11

Seq 1 2367 AAAATTCCAAA 2377

|  | \|\|\|\|\|\|\|\|\|\|\| |  | Seq 1 2766 | CGATTTTCTG | 2757 |
| --- | --- | --- | --- | --- | --- |
| Seq 2 1406 | AAAATTCCAAA | 1416 |  | \|\|\|\|\|\|\|\|\|\| |  |
|  |  |  | Seq 2 729 | CGATTTTCTG | 738 |

**OPPOSITE STRAND**

Alignment Length: 10; Identity: 10

Alignment Length: 10; Identity: 10

Seq 1 2371 TTCCAAAAAT 2380

|  | \|\|\|\|\|\|\|\|\|\| |  | Seq 1 | 2660 | GTTAAAAAATT | 2650 |
| --- | --- | --- | --- | --- | --- | --- |
| Seq 2 102 | TTCCAAAAAT | 111 |  |  | \|\|\|\|\|\|\|\|\|\|\| |  |
|  |  |  | Seq 2 | 634 | GTTAAAAAATT | 644 |

Alignment Length: 11; Identity: 11

Alignment Length: 10; Identity: 10

Seq 1 2513 AAAATTTAAA 2522

|  | \|\|\|\|\|\|\|\|\|\| |  | Seq 1 | 2482 | TTTTAAAGAC | 2473 |
| --- | --- | --- | --- | --- | --- | --- |
| Seq 2 107 | AAAATTTAAA | 116 |  |  | \|\|\|\|\|\|\|\|\|\| |  |
|  |  |  | Seq 2 | 651 | TTTTAAAGAC | 660 |

Alignment Length: 10; Identity: 10

Alignment Length: 10; Identity: 10

Seq 1 2514 AAATTTAAAG 2523

|  | \|\|\|\|\|\|\|\|\|\| |  | Seq 1 | 2447 | TTTCCATTTT | 2438 |
| --- | --- | --- | --- | --- | --- | --- |
| Seq 2 451 | AAATTTAAAG | 460 |  |  | \|\|\|\|\|\|\|\|\|\| |  |
|  |  |  | Seq 2 | 2742 | TTTCCATTTT | 2751 |

Alignment Length: 10; Identity: 10

Alignment Length: 10; Identity: 10

Seq 1 2514 AAATTTAAAG 2523

|  | \|\|\|\|\|\|\|\|\|\| |  | Seq 1 | 2377 | TTTGGAATTTT | 2367 |
| --- | --- | --- | --- | --- | --- | --- |
| Seq 2 663 | AAATTTAAAG | 672 |  |  | \|\|\|\|\|\|\|\|\|\|\| |  |
|  |  |  | Seq 2 | 2099 | TTTGGAATTTT | 2109 |

Alignment Length: 11; Identity: 11

Alignment Length: 10; Identity: 10

Seq 1 2567 CTTTTTAAAA 2576

|  | \|\|\|\|\|\|\|\|\|\| |  | Seq 1 | 2332 | GATTTTTTAAA | 2322 |
| --- | --- | --- | --- | --- | --- | --- |
| Seq 2 899 | CTTTTTAAAA | 908 |  |  | \|\|\|\|\|\|\|\|\|\|\| |  |
|  |  |  | Seq 2 | 2114 | GATTTTTTAAA | 2124 |

Alignment Length: 11; Identity: 11

Alignment Length: 10; Identity: 10

Seq 1 2568 TTTTTAAAAT 2577

|  | \|\|\|\|\|\|\|\|\|\| |  | Seq 1 | 2324 | AAAGAAATTTTC | 2313 |
| --- | --- | --- | --- | --- | --- | --- |
| Seq 2 1401 | TTTTTAAAAT | 1410 |  |  | \|\|\|\|\|\|\|\|\|\|\|\| |  |
|  |  |  | Seq 2 | 718 | AAAGAAATTTTC | 729 |

Alignment Length: 12; Identity: 12

**S7 Figure, continued.** Motifs with identity between *C. elegans* and orthologous *unc-25* upstream sequences.

Alignment Length: 10; Identity: 10

Alignment Length: 13; Identity: 13

| Seq 1 2273 | AAAAGATTTT | 2264 | Seq 1 | 1718 | AAATTTTTTTTGA 1706 |
| --- | --- | --- | --- | --- | --- |
|  | \|\|\|\|\|\|\|\|\|\| |  |  |  | \|\|\|\|\|\|\|\|\|\|\|\|\| |
| Seq 2 1078 | AAAAGATTTT | 1087 | Seq 2 | 547 | AAATTTTTTTTGA 559 |

Alignment Length: 10; Identity: 10

Alignment Length: 10; Identity: 10

| Seq 1 2217 | AAAGTTTTTT | 2208 | Seq 1 | 1717 | AATTTTTTTT | 1708 |
| --- | --- | --- | --- | --- | --- | --- |
|  | \|\|\|\|\|\|\|\|\|\| |  |  |  | \|\|\|\|\|\|\|\|\|\| |  |
| Seq 2 669 | AAAGTTTTTT | 678 | Seq 2 | 93 | AATTTTTTTT | 102 |

Alignment Length: 10; Identity: 10

Alignment Length: 10; Identity: 10

| Seq 1 2045 | AACTAAAATT | 2036 | Seq 1 | 1717 | AATTTTTTTT | 1708 |
| --- | --- | --- | --- | --- | --- | --- |
|  | \|\|\|\|\|\|\|\|\|\| |  |  |  | \|\|\|\|\|\|\|\|\|\| |  |
| Seq 2 123 | AACTAAAATT | 132 | Seq 2 | 1396 | AATTTTTTTT | 1405 |

Alignment Length: 10; Identity: 10

Alignment Length: 10; Identity: 10

| Seq 1 2044 | ACTAAAATTT | 2035 | Seq 1 | 1715 | TTTTTTTTGA | 1706 |
| --- | --- | --- | --- | --- | --- | --- |
|  | \|\|\|\|\|\|\|\|\|\| |  |  |  | \|\|\|\|\|\|\|\|\|\| |  |
| Seq 2 602 | ACTAAAATTT | 611 | Seq 2 | 485 | TTTTTTTTGA | 494 |

Alignment Length: 10; Identity: 10

Alignment Length: 10; Identity: 10

| Seq 1 2041 | AAAATTTAAA | 2032 | Seq 1 | 1712 | TTTTTGATTA | 1703 |
| --- | --- | --- | --- | --- | --- | --- |
|  | \|\|\|\|\|\|\|\|\|\| |  |  |  | \|\|\|\|\|\|\|\|\|\| |  |
| Seq 2 107 | AAAATTTAAA | 116 | Seq 2 | 2462 | TTTTTGATTA | 2471 |

Alignment Length: 12; Identity: 12

Alignment Length: 10; Identity: 10

| Seq 1 2037 | TTTAAAAAATAA | 2026 | Seq 1 | 1708 | TGATTATTAT | 1699 |
| --- | --- | --- | --- | --- | --- | --- |
|  | \|\|\|\|\|\|\|\|\|\|\|\| |  |  |  | \|\|\|\|\|\|\|\|\|\| |  |
| Seq 2 1329 | TTTAAAAAATAA | 1340 | Seq 2 | 1541 | TGATTATTAT | 1550 |

Alignment Length: 10; Identity: 10

Alignment Length: 11; Identity: 11

| Seq 1 1988 | AATCAAAAAA | 1979 | Seq 1 | 1600 | ACATATTTTTA | 1590 |
| --- | --- | --- | --- | --- | --- | --- |
|  | \|\|\|\|\|\|\|\|\|\| |  |  |  | \|\|\|\|\|\|\|\|\|\|\| |  |
| Seq 2 419 | AATCAAAAAA | 428 | Seq 2 | 2350 | ACATATTTTTA | 2360 |

Alignment Length: 14; Identity: 14

Alignment Length: 11; Identity: 11

| Seq 1 1984 | AAAAAAATATTTTT 1971 | Seq 1 | 1518 | TCTTTTTTTTT | 1508 |
| --- | --- | --- | --- | --- | --- |
|  | \|\|\|\|\|\|\|\|\|\|\|\|\|\| |  |  | \|\|\|\|\|\|\|\|\|\|\| |  |
| Seq 2 905 | AAAAAAATATTTTT 918 | Seq 2 | 1593 | TCTTTTTTTTT | 1603 |

Alignment Length: 11; Identity: 11

Alignment Length: 10; Identity: 10

| Seq 1 1908 | AAATTTTTCAA | 1898 | Seq 1 | 1518 | TCTTTTTTTT | 1509 |
| --- | --- | --- | --- | --- | --- | --- |
|  | \|\|\|\|\|\|\|\|\|\|\| |  |  |  | \|\|\|\|\|\|\|\|\|\| |  |
| Seq 2 2660 | AAATTTTTCAA | 2670 | Seq 2 | 1606 | TCTTTTTTTT | 1615 |

Alignment Length: 10; Identity: 10

Alignment Length: 10; Identity: 10

| Seq 1 1907 | AATTTTTCAA | 1898 | Seq 1 | 1516 | TTTTTTTTTC | 1507 |
| --- | --- | --- | --- | --- | --- | --- |
|  | \|\|\|\|\|\|\|\|\|\| |  |  |  | \|\|\|\|\|\|\|\|\|\| |  |
| Seq 2 2696 | AATTTTTCAA | 2705 | Seq 2 | 95 | TTTTTTTTTC | 104 |

Alignment Length: 10; Identity: 10

Alignment Length: 11; Identity: 11

| Seq 1 1894 | AATAATTTTT | 1885 | Seq 1 | 1516 | TTTTTTTTTCA | 1506 |
| --- | --- | --- | --- | --- | --- | --- |
|  | \|\|\|\|\|\|\|\|\|\| |  |  |  | \|\|\|\|\|\|\|\|\|\|\| |  |
| Seq 2 993 | AATAATTTTT | 1002 | Seq 2 | 915 | TTTTTTTTTCA | 925 |

Alignment Length: 10; Identity: 10

Alignment Length: 11; Identity: 11

| Seq 1 1893 | ATAATTTTTT | 1884 | Seq 1 | 1515 | TTTTTTTTCAA | 1505 |
| --- | --- | --- | --- | --- | --- | --- |
|  | \|\|\|\|\|\|\|\|\|\| |  |  |  | \|\|\|\|\|\|\|\|\|\|\| |  |
| Seq 2 91 | ATAATTTTTT | 100 | Seq 2 | 1608 | TTTTTTTTCAA | 1618 |

Alignment Length: 10; Identity: 10

Alignment Length: 10; Identity: 10

| Seq 1 1891 | AATTTTTTAC | 1882 | Seq 1 1495 | TGTAATTTTT | 1486 |
| --- | --- | --- | --- | --- | --- |
|  | \|\|\|\|\|\|\|\|\|\| |  |  | \|\|\|\|\|\|\|\|\|\| |  |
| Seq 2 831 | AATTTTTTAC | 840 | Seq 2 806 | TGTAATTTTT | 815 |

**S7 Figure, continued.** Motifs with identity between *C. elegans* and orthologous *unc-25* upstream sequences.

Alignment Length: 10; Identity: 10

Alignment Length: 11; Identity: 11

| Seq 1 1479 | TTTTCTATTT | 1470 | Seq 1 | 1043 | TTTAAAAAAAT | 1033 |
| --- | --- | --- | --- | --- | --- | --- |
|  | \|\|\|\|\|\|\|\|\|\| |  |  |  | \|\|\|\|\|\|\|\|\|\|\| |  |
| Seq 2 1556 | TTTTCTATTT | 1565 | Seq 2 | 2475 | TTTAAAAAAAT | 2485 |

Alignment Length: 10; Identity: 10

Alignment Length: 12; Identity: 12

| Seq 1 1470 | TAATTTTTTT | 1461 | Seq 1 | 1025 | AAAATCAAAAAA | 1014 |
| --- | --- | --- | --- | --- | --- | --- |
|  | \|\|\|\|\|\|\|\|\|\| |  |  |  | \|\|\|\|\|\|\|\|\|\|\|\| |  |
| Seq 2 92 | TAATTTTTTT | 101 | Seq 2 | 417 | AAAATCAAAAAA | 428 |

Alignment Length: 10; Identity: 10

Alignment Length: 10; Identity: 10

| Seq 1 1470 | TAATTTTTTT | 1461 | Seq 1 | 919 | TTCTCTCAGT | 910 |
| --- | --- | --- | --- | --- | --- | --- |
|  | \|\|\|\|\|\|\|\|\|\| |  |  |  | \|\|\|\|\|\|\|\|\|\| |  |
| Seq 2 808 | TAATTTTTTT | 817 | Seq 2 | 1349 | TTCTCTCAGT | 1358 |

Alignment Length: 10; Identity: 10

Alignment Length: 10; Identity: 10

| Seq 1 1369 | ATCTAAAATT | 1360 | Seq 1 | 809 | AAAAATTAAA | 800 |
| --- | --- | --- | --- | --- | --- | --- |
|  | \|\|\|\|\|\|\|\|\|\| |  |  |  | \|\|\|\|\|\|\|\|\|\| |  |
| Seq 2 707 | ATCTAAAATT | 716 | Seq 2 | 2480 | AAAAATTAAA | 2489 |

Alignment Length: 10; Identity: 10

Alignment Length: 10; Identity: 10

| Seq 1 1364 | AAATTTATAA | 1355 | Seq 1 | 804 | TTAAAAAATA | 795 |
| --- | --- | --- | --- | --- | --- | --- |
|  | \|\|\|\|\|\|\|\|\|\| |  |  |  | \|\|\|\|\|\|\|\|\|\| |  |
| Seq 2 943 | AAATTTATAA | 952 | Seq 2 | 1330 | TTAAAAAATA | 1339 |

Alignment Length: 10; Identity: 10

Alignment Length: 13; Identity: 13

| Seq 1 1359 | TATAATTTTT | 1350 | Seq 1 | 802 | AAAAAATATTTTT 790 |
| --- | --- | --- | --- | --- | --- |
|  | \|\|\|\|\|\|\|\|\|\| |  |  |  | \|\|\|\|\|\|\|\|\|\|\|\|\| |
| Seq 2 90 | TATAATTTTT | 99 | Seq 2 | 906 | AAAAAATATTTTT 918 |

Alignment Length: 11; Identity: 11

Alignment Length: 11; Identity: 11

| Seq 1 1292 | ACTCAAAAAAA | 1282 | Seq 1 | 757 | AAAAAAATTAA | 747 |
| --- | --- | --- | --- | --- | --- | --- |
|  | \|\|\|\|\|\|\|\|\|\|\| |  |  |  | \|\|\|\|\|\|\|\|\|\|\| |  |
| Seq 2 856 | ACTCAAAAAAA | 866 | Seq 2 | 2478 | AAAAAAATTAA | 2488 |

Alignment Length: 10; Identity: 10

Alignment Length: 11; Identity: 11

| Seq 1 1250 | CTTCTTTTCC | 1241 | Seq 1 | 697 | CAAAAAATGTC | 687 |
| --- | --- | --- | --- | --- | --- | --- |
|  | \|\|\|\|\|\|\|\|\|\| |  |  |  | \|\|\|\|\|\|\|\|\|\|\| |  |
| Seq 2 327 | CTTCTTTTCC | 336 | Seq 2 | 2230 | CAAAAAATGTC | 2240 |

Alignment Length: 11; Identity: 11

Alignment Length: 10; Identity: 10

| Seq 1 1107 | GGAAAAAAAAT | 1097 | Seq 1 | 695 | AAAAATGTCC | 686 |
| --- | --- | --- | --- | --- | --- | --- |
|  | \|\|\|\|\|\|\|\|\|\|\| |  |  |  | \|\|\|\|\|\|\|\|\|\| |  |
| Seq 2 622 | GGAAAAAAAAT | 632 | Seq 2 | 1957 | AAAAATGTCC | 1966 |

Alignment Length: 10; Identity: 10

Alignment Length: 10; Identity: 10

| Seq 1 1087 | TTTAAAAAAT | 1078 | Seq 1 | 461 | GAAAGAAATT | 452 |
| --- | --- | --- | --- | --- | --- | --- |
|  | \|\|\|\|\|\|\|\|\|\| |  |  |  | \|\|\|\|\|\|\|\|\|\| |  |
| Seq 2 1329 | TTTAAAAAAT | 1338 | Seq 2 | 717 | GAAAGAAATT | 726 |

Alignment Length: 10; Identity: 10

Alignment Length: 10; Identity: 10

| Seq 1 1086 | TTAAAAAATT | 1077 | Seq 1 | 457 | GAAATTCTTT | 448 |
| --- | --- | --- | --- | --- | --- | --- |
|  | \|\|\|\|\|\|\|\|\|\| |  |  |  | \|\|\|\|\|\|\|\|\|\| |  |
| Seq 2 635 | TTAAAAAATT | 644 | Seq 2 | 1318 | GAAATTCTTT | 1327 |

Alignment Length: 11; Identity: 11

Alignment Length: 10; Identity: 10

| Seq 1 1082 | AAAATTCCAAA | 1072 | Seq 1 314 | TTAGTTTTTA | 305 |
| --- | --- | --- | --- | --- | --- |
|  | \|\|\|\|\|\|\|\|\|\|\| |  |  | \|\|\|\|\|\|\|\|\|\| |  |
| Seq 2 1406 | AAAATTCCAAA | 1416 | Seq 2 2469 | TTAGTTTTTA | 2478 |

Alignment Length: 14; Identity: 14

Alignment Length: 10; Identity: 10

| Seq 1 1043 | TTTAAAAAAATATT 1030 | Seq 1 | 303 | GAAATTTAAA | 294 |
| --- | --- | --- | --- | --- | --- |
|  | \|\|\|\|\|\|\|\|\|\|\|\|\|\| |  |  | \|\|\|\|\|\|\|\|\|\| |  |
| Seq 2 902 | TTTAAAAAAATATT 915 | Seq 2 | 450 | GAAATTTAAA | 459 |

**S7 Figure, continued.** Motifs with identity between *C. elegans* and orthologous *unc-25* upstream sequences.

Alignment Length: 10; Identity: 10

| Seq 1 303 | GAAATTTAAA | 294 |
| --- | --- | --- |
|  | \|\|\|\|\|\|\|\|\|\| |  |
| Seq 2 662 | GAAATTTAAA | 671 |

Alignment Length: 10; Identity: 10

| Seq 1 | 62 | ATAAAATAAA | 53 |
| --- | --- | --- | --- |
|  |  | \|\|\|\|\|\|\|\|\|\| |  |
| Seq 2 | 2273 | ATAAAATAAA | 2282 |

**S7 Figure, continued.** Motifs with identity between *C. elegans* and orthologous *unc-25* upstream sequences.

**B. malayi/C. elegans unc-25, 11bp window**

Alignment Length: 11; Identity: 11

|  | | Seq 1 | 1371 | TTTTTTTTTTG | 1381 |
| --- | --- | --- | --- | --- | --- |
| **seq 1:** | **bmaunc25** |  |  | \|\|\|\|\|\|\|\|\|\|\| |  |
| **seq 2:** | **celunc25** | Seq 2 | 673 | TTTTTTTTTTG | 683 |

Alignment Length: 13; Identity: 13

Alignment Length: 13; Identity: 13

| Seq 1 538 | TTTTTTTAATATA | 550 | Seq 1 | 1383 | TTTCTTTTTTTTT 1395 |
| --- | --- | --- | --- | --- | --- |
|  | \|\|\|\|\|\|\|\|\|\|\|\|\| |  |  |  | \|\|\|\|\|\|\|\|\|\|\|\|\| |
| Seq 2 2368 | TTTTTTTAATATA | 2380 | Seq 2 | 1591 | TTTCTTTTTTTTT 1603 |

Alignment Length: 12; Identity: 12

Alignment Length: 11; Identity: 11

| Seq 1 858 | AGTTTTTTTTTT | 869 | Seq 1 | 1389 | TTTTTTTTTTG | 1399 |
| --- | --- | --- | --- | --- | --- | --- |
|  | \|\|\|\|\|\|\|\|\|\|\|\| |  |  |  | \|\|\|\|\|\|\|\|\|\|\| |  |
| Seq 2 671 | AGTTTTTTTTTT | 682 | Seq 2 | 483 | TTTTTTTTTTG | 493 |

Alignment Length: 11; Identity: 11

Alignment Length: 11; Identity: 11

| Seq 1 861 | TTTTTTTTTTC | 871 | Seq 1 | 1389 | TTTTTTTTTTG | 1399 |
| --- | --- | --- | --- | --- | --- | --- |
|  | \|\|\|\|\|\|\|\|\|\|\| |  |  |  | \|\|\|\|\|\|\|\|\|\|\| |  |
| Seq 2 914 | TTTTTTTTTTC | 924 | Seq 2 | 673 | TTTTTTTTTTG | 683 |

Alignment Length: 12; Identity: 12

Alignment Length: 12; Identity: 12

| Seq 1 976 | ACTCTTTTTAAG | 987 | Seq 1 | 1390 | TTTTTTTTTGCT | 1401 |
| --- | --- | --- | --- | --- | --- | --- |
|  | \|\|\|\|\|\|\|\|\|\|\|\| |  |  |  | \|\|\|\|\|\|\|\|\|\|\|\| |  |
| Seq 2 2194 | ACTCTTTTTAAG | 2205 | Seq 2 | 1595 | TTTTTTTTTGCT | 1606 |

Alignment Length: 12; Identity: 12

Alignment Length: 12; Identity: 12

| Seq 1 1042 | TCTTTTTTTTTG | 1053 | Seq 1 | 1494 | GAAAGCATTTTT | 1505 |
| --- | --- | --- | --- | --- | --- | --- |
|  | \|\|\|\|\|\|\|\|\|\|\|\| |  |  |  | \|\|\|\|\|\|\|\|\|\|\|\| |  |
| Seq 2 1593 | TCTTTTTTTTTG | 1604 | Seq 2 | 1266 | GAAAGCATTTTT | 1277 |

Alignment Length: 11; Identity: 11

Alignment Length: 11; Identity: 11

| Seq 1 1083 | AAAATATTTTT | 1093 | Seq 1 | 1857 | TTAAAAAATAA | 1867 |
| --- | --- | --- | --- | --- | --- | --- |
|  | \|\|\|\|\|\|\|\|\|\|\| |  |  |  | \|\|\|\|\|\|\|\|\|\|\| |  |
| Seq 2 908 | AAAATATTTTT | 918 | Seq 2 | 1330 | TTAAAAAATAA | 1340 |

Alignment Length: 11; Identity: 11

Alignment Length: 11; Identity: 11

| Seq 1 1345 | TTCTTCTTTTC | 1355 | Seq 1 | 1880 | GGATTTTTTTT | 1890 |
| --- | --- | --- | --- | --- | --- | --- |
|  | \|\|\|\|\|\|\|\|\|\|\| |  |  |  | \|\|\|\|\|\|\|\|\|\|\| |  |
| Seq 2 325 | TTCTTCTTTTC | 335 | Seq 2 | 2364 | GGATTTTTTTT | 2374 |

Alignment Length: 13; Identity: 13

Alignment Length: 12; Identity: 12

| Seq 1 1352 | TTTCTTTTTTTTT | 1364 | Seq 1 | 1881 | GATTTTTTTTTT | 1892 |
| --- | --- | --- | --- | --- | --- | --- |
|  | \|\|\|\|\|\|\|\|\|\|\|\|\| |  |  |  | \|\|\|\|\|\|\|\|\|\|\|\| |  |
| Seq 2 1591 | TTTCTTTTTTTTT | 1603 | Seq 2 | 481 | GATTTTTTTTTT | 492 |

Alignment Length: 11; Identity: 11

Alignment Length: 11; Identity: 11

| Seq 1 1356 | TTTTTTTTTTC | 1366 | Seq 1 | 1882 | ATTTTTTTTTT | 1892 |
| --- | --- | --- | --- | --- | --- | --- |
|  | \|\|\|\|\|\|\|\|\|\|\| |  |  |  | \|\|\|\|\|\|\|\|\|\|\| |  |
| Seq 2 914 | TTTTTTTTTTC | 924 | Seq 2 | 913 | ATTTTTTTTTT | 923 |

Alignment Length: 11; Identity: 11

Alignment Length: 11; Identity: 11

| Seq 1 1357 | TTTTTTTTTCC | 1367 | Seq 1 | 1885 | TTTTTTTTAAA | 1895 |
| --- | --- | --- | --- | --- | --- | --- |
|  | \|\|\|\|\|\|\|\|\|\|\| |  |  |  | \|\|\|\|\|\|\|\|\|\|\| |  |
| Seq 2 95 | TTTTTTTTTCC | 105 | Seq 2 | 1398 | TTTTTTTTAAA | 1408 |

Alignment Length: 12; Identity: 12

Alignment Length: 11; Identity: 11

| Seq 1 1364 | TTCCTTTTTTTT | 1375 | Seq 1 1885 | TTTTTTTTAAA | 1895 |
| --- | --- | --- | --- | --- | --- |
|  | \|\|\|\|\|\|\|\|\|\|\|\| |  |  | \|\|\|\|\|\|\|\|\|\|\| |  |
| Seq 2 1376 | TTCCTTTTTTTT | 1387 | Seq 2 1529 | TTTTTTTTAAA | 1539 |

Alignment Length: 11; Identity: 11

Alignment Length: 11; Identity: 11

| Seq 1 1371 | TTTTTTTTTTG | 1381 | Seq 1 | 1984 | TCCAAAAATTT | 1994 |
| --- | --- | --- | --- | --- | --- | --- |
|  | \|\|\|\|\|\|\|\|\|\|\| |  |  |  | \|\|\|\|\|\|\|\|\|\|\| |  |
| Seq 2 483 | TTTTTTTTTTG | 493 | Seq 2 | 103 | TCCAAAAATTT | 113 |

**S7 Figure, continued.** Motifs with identity between *C. elegans* and orthologous *unc-25* upstream sequences.

Alignment Length: 11; Identity: 11

Alignment Length: 11; Identity: 11

| Seq 1 2033 | CTCTTTTTTTT | 2043 | Seq 1 | 2724 | TTTTTTTTAAT | 2734 |
| --- | --- | --- | --- | --- | --- | --- |
|  | \|\|\|\|\|\|\|\|\|\|\| |  |  |  | \|\|\|\|\|\|\|\|\|\|\| |  |
| Seq 2 1605 | CTCTTTTTTTT | 1615 | Seq 2 | 2367 | TTTTTTTTAAT | 2377 |

Alignment Length: 11; Identity: 11

Seq 1 2034 TCTTTTTTTTT 2044

|||||||||||

Seq 2 1593 TCTTTTTTTTT 1603

Alignment Length: 11; Identity: 11

Seq 1 2038 TTTTTTTTAAT 2048

|||||||||||

Seq 2 2367 TTTTTTTTAAT 2377

Alignment Length: 12; Identity: 12

Seq 1 2109 GGAAATTGTCAA 2120

||||||||||||

Seq 2 2772 GGAAATTGTCAA 2783

Alignment Length: 11; Identity: 11

Seq 1 2543 TTAAAAAATAA 2553

|||||||||||

Seq 2 1330 TTAAAAAATAA 1340

Alignment Length: 11; Identity: 11

Seq 1 2566 GGATTTTTTTT 2576

|||||||||||

Seq 2 2364 GGATTTTTTTT 2374

Alignment Length: 12; Identity: 12

Seq 1 2567 GATTTTTTTTTT 2578

||||||||||||

Seq 2 481 GATTTTTTTTTT 492

Alignment Length: 11; Identity: 11

Seq 1 2568 ATTTTTTTTTT 2578

|||||||||||

Seq 2 913 ATTTTTTTTTT 923

Alignment Length: 11; Identity: 11

Seq 1 2571 TTTTTTTTAAA 2581

|||||||||||

Seq 2 1398 TTTTTTTTAAA 1408

Alignment Length: 11; Identity: 11

Seq 1 2571 TTTTTTTTAAA 2581

|||||||||||

Seq 2 1529 TTTTTTTTAAA 1539

Alignment Length: 11; Identity: 11

Seq 1 2670 TCCAAAAATTT 2680

|||||||||||

Seq 2 103 TCCAAAAATTT 113

Alignment Length: 11; Identity: 11

Seq 1 2719 CTCTTTTTTTT 2729

|||||||||||

Seq 2 1605 CTCTTTTTTTT 1615

Alignment Length: 11; Identity: 11

Seq 1 2720 TCTTTTTTTTT 2730

|||||||||||

Seq 2 1593 TCTTTTTTTTT 1603

**OPPOSITE STRAND**

Alignment Length: 11; Identity: 11

Seq 1 2667 TTATTTTTTTT 2657

|||||||||||

Seq 2 1579 TTATTTTTTTT 1589

Alignment Length: 11; Identity: 11

Seq 1 2666 TATTTTTTTTT 2656

|||||||||||

Seq 2 912 TATTTTTTTTT 922

Alignment Length: 11; Identity: 11

Seq 1 2599 TTTTCAGAAAA 2589

|||||||||||

Seq 2 920 TTTTCAGAAAA 930

Alignment Length: 12; Identity: 12

Seq 1 2578 AAAAAAAAAATC 2567

||||||||||||

Seq 2 411 AAAAAAAAAATC 422

Alignment Length: 12; Identity: 12

Seq 1 2578 AAAAAAAAAATC 2567

||||||||||||

Seq 2 512 AAAAAAAAAATC 523

Alignment Length: 13; Identity: 13

Seq 1 2571 AAATCCCAAAAAT 2559

|||||||||||||

Seq 2 1447 AAATCCCAAAAAT 1459

Alignment Length: 11; Identity: 11

Seq 1 2562 AAATTATTTTT 2552

|||||||||||

Seq 2 1576 AAATTATTTTT 1586

Alignment Length: 11; Identity: 11

Seq 1 2379 TATATGAATTT 2369

|||||||||||

Seq 2 2508 TATATGAATTT 2518

Alignment Length: 11; Identity: 11

Seq 1 2204 AATTTTTGCAT 2194

|||||||||||

Seq 2 135 AATTTTTGCAT 145

Alignment Length: 11; Identity: 11

Seq 1 2108 TCCAAAAATTT 2098

|||||||||||

Seq 2 103 TCCAAAAATTT 113

Alignment Length: 11; Identity: 11

Seq 1 1981 TTATTTTTTTT 1971

|||||||||||

Seq 2 1579 TTATTTTTTTT 1589

Alignment Length: 11; Identity: 11

Seq 1 1980 TATTTTTTTTT 1970

|||||||||||

Seq 2 912 TATTTTTTTTT 922

**S7 Figure, continued.** Motifs with identity between *C. elegans* and orthologous *unc-25* upstream sequences.

Alignment Length: 11; Identity: 11

Alignment Length: 11; Identity: 11

| Seq 1 1913 | TTTTCAGAAAA | 1903 | Seq 1 | 739 | AATTTGCCGCT | 729 |
| --- | --- | --- | --- | --- | --- | --- |
|  | \|\|\|\|\|\|\|\|\|\|\| |  |  |  | \|\|\|\|\|\|\|\|\|\|\| |  |
| Seq 2 920 | TTTTCAGAAAA | 930 | Seq 2 | 2876 | AATTTGCCGCT | 2886 |

Alignment Length: 12; Identity: 12

Alignment Length: 12; Identity: 12

| Seq 1 1892 | AAAAAAAAAATC | 1881 | Seq 1 | 546 | TTAAAAAAATAT | 535 |
| --- | --- | --- | --- | --- | --- | --- |
|  | \|\|\|\|\|\|\|\|\|\|\|\| |  |  |  | \|\|\|\|\|\|\|\|\|\|\|\| |  |
| Seq 2 411 | AAAAAAAAAATC | 422 | Seq 2 | 903 | TTAAAAAAATAT | 914 |

Alignment Length: 12; Identity: 12

Alignment Length: 11; Identity: 11

| Seq 1 1892 | AAAAAAAAAATC | 1881 | Seq 1 | 430 | ATAAAAAATCT | 420 |
| --- | --- | --- | --- | --- | --- | --- |
|  | \|\|\|\|\|\|\|\|\|\|\|\| |  |  |  | \|\|\|\|\|\|\|\|\|\|\| |  |
| Seq 2 512 | AAAAAAAAAATC | 523 | Seq 2 | 2378 | ATAAAAAATCT | 2388 |

Alignment Length: 13; Identity: 13

Alignment Length: 11; Identity: 11

| Seq 1 1885 | AAATCCCAAAAAT | 1873 | Seq 1 | 394 | AATTTTTCAAT | 384 |
| --- | --- | --- | --- | --- | --- | --- |
|  | \|\|\|\|\|\|\|\|\|\|\|\|\| |  |  |  | \|\|\|\|\|\|\|\|\|\|\| |  |
| Seq 2 1447 | AAATCCCAAAAAT | 1459 | Seq 2 | 2661 | AATTTTTCAAT | 2671 |

Alignment Length: 11; Identity: 11

Alignment Length: 11; Identity: 11

| Seq 1 1876 | AAATTATTTTT | 1866 | Seq 1 | 394 | AATTTTTCAAT | 384 |
| --- | --- | --- | --- | --- | --- | --- |
|  | \|\|\|\|\|\|\|\|\|\|\| |  |  |  | \|\|\|\|\|\|\|\|\|\|\| |  |
| Seq 2 1576 | AAATTATTTTT | 1586 | Seq 2 | 2696 | AATTTTTCAAT | 2706 |

Alignment Length: 11; Identity: 11

| Seq 1 1691 | TATATGAATTT | 1681 |
| --- | --- | --- |
|  | \|\|\|\|\|\|\|\|\|\|\| |  |
| Seq 2 2508 | TATATGAATTT | 2518 |

Alignment Length: 11; Identity: 11

| Seq 1 1399 | CAAAAAAAAAA | 1389 |
| --- | --- | --- |
|  | \|\|\|\|\|\|\|\|\|\|\| |  |
| Seq 2 511 | CAAAAAAAAAA | 521 |

Alignment Length: 11; Identity: 11

| Seq 1 1381 | CAAAAAAAAAA | 1371 |
| --- | --- | --- |
|  | \|\|\|\|\|\|\|\|\|\|\| |  |
| Seq 2 511 | CAAAAAAAAAA | 521 |

Alignment Length: 11; Identity: 11

| Seq 1 1366 | GAAAAAAAAAA | 1356 |
| --- | --- | --- |
|  | \|\|\|\|\|\|\|\|\|\|\| |  |
| Seq 2 410 | GAAAAAAAAAA | 420 |

Alignment Length: 11; Identity: 11

| Seq 1 1191 | AGTTTTTAAAA | 1181 |
| --- | --- | --- |
|  | \|\|\|\|\|\|\|\|\|\|\| |  |
| Seq 2 2471 | AGTTTTTAAAA | 2481 |

Alignment Length: 11; Identity: 11

| Seq 1 1189 | TTTTTAAAATT | 1179 |
| --- | --- | --- |
|  | \|\|\|\|\|\|\|\|\|\|\| |  |
| Seq 2 1401 | TTTTTAAAATT | 1411 |

Alignment Length: 11; Identity: 11

| Seq 1 1093 | AAAAATATTTT | 1083 |
| --- | --- | --- |
|  | \|\|\|\|\|\|\|\|\|\|\| |  |
| Seq 2 907 | AAAAATATTTT | 917 |

Alignment Length: 12; Identity: 12

| Seq 1 872 | AGAAAAAAAAAA | 861 |
| --- | --- | --- |
|  | \|\|\|\|\|\|\|\|\|\|\|\| |  |
| Seq 2 409 | AGAAAAAAAAAA | 420 |

**S7 Figure, continued.** Motifs with identity between *C. elegans* and orthologous *unc-25* upstream sequences.

**T. spiralis/C. elegans unc-25, 10bp window**

Alignment Length: 10; Identity: 10

|  | | Seq 1 | 800 | TGTAGAAAAT | 809 |
| --- | --- | --- | --- | --- | --- |
| **seq 1:** | **tspunc25** |  |  | \|\|\|\|\|\|\|\|\|\| |  |
| **seq 2:** | **celunc25** | Seq 2 | 2309 | TGTAGAAAAT | 2318 |

Alignment Length: 10; Identity: 10

Seq 1 146 AAGAAATTTT 155

|  | \|\|\|\|\|\|\|\|\|\| |  | Seq 1 | 854 | ATATAAAAAA | 863 |
| --- | --- | --- | --- | --- | --- | --- |
| Seq 2 722 | AAGAAATTTT | 731 |  |  | \|\|\|\|\|\|\|\|\|\| |  |
|  |  |  | Seq 2 | 2379 | ATATAAAAAA | 2388 |

Alignment Length: 10; Identity: 10

Alignment Length: 10; Identity: 10

Seq 1 148 GAAATTTTTT 157

|  | \|\|\|\|\|\|\|\|\|\| |  | Seq 1 | 857 | TAAAAAAAAT | 866 |
| --- | --- | --- | --- | --- | --- | --- |
| Seq 2 549 | GAAATTTTTT | 558 |  |  | \|\|\|\|\|\|\|\|\|\| |  |
|  |  |  | Seq 2 | 227 | TAAAAAAAAT | 236 |

Alignment Length: 10; Identity: 10

Alignment Length: 10; Identity: 10

Seq 1 173 TTTACAAAAT 182

| \|\|\|\|\|\|\|\|\|\| | | | Seq 1 | 938 | TCGAAAAATC | 947 |
| --- | --- | --- | --- | --- | --- | --- |
| Seq 2 839 | TTTACAAAAT | 848 |  |  | \|\|\|\|\|\|\|\|\|\| |  |
|  |  |  | Seq 2 | 567 | TCGAAAAATC | 576 |

Alignment Length: 10; Identity: 10

Alignment Length: 12; Identity: 12

Seq 1 260 ATTTCCATCTTC 271

|  | \|\|\|\|\|\|\|\|\|\|\|\| |  | Seq 1 | 985 | CTCAAATTTG | 994 |
| --- | --- | --- | --- | --- | --- | --- |
| Seq 2 2589 | ATTTCCATCTTC | 2600 |  |  | \|\|\|\|\|\|\|\|\|\| |  |
|  |  |  | Seq 2 | 172 | CTCAAATTTG | 181 |

Alignment Length: 10; Identity: 10

Alignment Length: 10; Identity: 10

Seq 1 288 AGCTGAAAAT 297

|  | \|\|\|\|\|\|\|\|\|\| |  | Seq 1 | 1061 | CAAAAAAAAA | 1070 |
| --- | --- | --- | --- | --- | --- | --- |
| Seq 2 870 | AGCTGAAAAT | 879 |  |  | \|\|\|\|\|\|\|\|\|\| |  |
|  |  |  | Seq 2 | 514 | CAAAAAAAAA | 523 |

Alignment Length: 10; Identity: 10

Alignment Length: 11; Identity: 11

Seq 1 379 AAACTGAAAAC 389

|  | \|\|\|\|\|\|\|\|\|\|\| |  | Seq 1 | 1062 | AAAAAAAAAC | 1071 |
| --- | --- | --- | --- | --- | --- | --- |
| Seq 2 3005 | AAACTGAAAAC | 3015 |  |  | \|\|\|\|\|\|\|\|\|\| |  |
|  |  |  | Seq 2 | 762 | AAAAAAAAAC | 771 |

Alignment Length: 10; Identity: 10

Alignment Length: 10; Identity: 10

Seq 1 485 ATTATTTTTT 494

|  | \|\|\|\|\|\|\|\|\|\| |  | Seq 1 | 1123 | GAAAAATCGAT | 1133 |
| --- | --- | --- | --- | --- | --- | --- |
| Seq 2 1581 | ATTATTTTTT | 1590 |  |  | \|\|\|\|\|\|\|\|\|\|\| |  |
|  |  |  | Seq 2 | 2167 | GAAAAATCGAT | 2177 |

Alignment Length: 11; Identity: 11

Alignment Length: 10; Identity: 10

Seq 1 499 ATAAAATAAA 508

|  | \|\|\|\|\|\|\|\|\|\| |  | Seq 1 | 1280 | AATAAAATAAAA | 1291 |
| --- | --- | --- | --- | --- | --- | --- |
| Seq 2 2276 | ATAAAATAAA | 2285 |  |  | \|\|\|\|\|\|\|\|\|\|\|\| |  |
|  |  |  | Seq 2 | 2275 | AATAAAATAAAA | 2286 |

Alignment Length: 12; Identity: 12

Alignment Length: 13; Identity: 13

Seq 1 723 AAAAACCAATAAT 735

|  | \|\|\|\|\|\|\|\|\|\|\|\|\| |  | Seq 1 | 1401 | TATAAAAAAT | 1410 |
| --- | --- | --- | --- | --- | --- | --- |
| Seq 2 197 | AAAAACCAATAAT | 209 |  |  | \|\|\|\|\|\|\|\|\|\| |  |
|  |  |  | Seq 2 | 2380 | TATAAAAAAT | 2389 |

Alignment Length: 10; Identity: 10

Alignment Length: 10; Identity: 10

Seq 1 739 CTGAAAATTT 748

|  | \|\|\|\|\|\|\|\|\|\| |  | Seq 1 | 1415 | TTTTTTTTCA | 1424 |
| --- | --- | --- | --- | --- | --- | --- |
| Seq 2 872 | CTGAAAATTT | 881 |  |  | \|\|\|\|\|\|\|\|\|\| |  |
|  |  |  | Seq 2 | 919 | TTTTTTTTCA | 928 |

Alignment Length: 10; Identity: 10

Alignment Length: 10; Identity: 10

Seq 1 743 AAATTTTCAA 752

|  | \|\|\|\|\|\|\|\|\|\| |  | Seq 1 1415 | TTTTTTTTCA | 1424 |
| --- | --- | --- | --- | --- | --- |
| Seq 2 2897 | AAATTTTCAA | 2906 |  | \|\|\|\|\|\|\|\|\|\| |  |
|  |  |  | Seq 2 1611 | TTTTTTTTCA | 1620 |

Alignment Length: 10; Identity: 10

Alignment Length: 10; Identity: 10

Seq 1 791 ATGTAATTTT 800

|  | \|\|\|\|\|\|\|\|\|\| |  | Seq 1 | 1423 | CATTTTTTTGA | 1433 |
| --- | --- | --- | --- | --- | --- | --- |
| Seq 2 808 | ATGTAATTTT | 817 |  |  | \|\|\|\|\|\|\|\|\|\|\| |  |
|  |  |  | Seq 2 | 1520 | CATTTTTTTGA | 1530 |

Alignment Length: 11; Identity: 11

**S7 Figure, continued.** Motifs with identity between *C. elegans* and orthologous *unc-25* upstream sequences.

Alignment Length: 11; Identity: 11

Alignment Length: 10; Identity: 10

| Seq 1 1836 | CAAAAACTATG | 1846 | Seq 1 | 2779 | TTTTTTTCAA | 2788 |
| --- | --- | --- | --- | --- | --- | --- |
|  | \|\|\|\|\|\|\|\|\|\|\| |  |  |  | \|\|\|\|\|\|\|\|\|\| |  |
| Seq 2 2157 | CAAAAACTATG | 2167 | Seq 2 | 1612 | TTTTTTTCAA | 1621 |

Alignment Length: 10; Identity: 10

Alignment Length: 10; Identity: 10

| Seq 1 2060 | ACATACATTT | 2069 | Seq 1 | 2818 | ATAATATTTT | 2827 |
| --- | --- | --- | --- | --- | --- | --- |
|  | \|\|\|\|\|\|\|\|\|\| |  |  |  | \|\|\|\|\|\|\|\|\|\| |  |
| Seq 2 2211 | ACATACATTT | 2220 | Seq 2 | 1291 | ATAATATTTT | 1300 |

Alignment Length: 10; Identity: 10

Alignment Length: 11; Identity: 11

| Seq 1 2089 | TTTTTTAAAA | 2098 | Seq 1 | 3134 | AAATTTTGAAT | 3144 |
| --- | --- | --- | --- | --- | --- | --- |
|  | \|\|\|\|\|\|\|\|\|\| |  |  |  | \|\|\|\|\|\|\|\|\|\|\| |  |
| Seq 2 1329 | TTTTTTAAAA | 1338 | Seq 2 | 609 | AAATTTTGAAT | 619 |

Alignment Length: 11; Identity: 11

Alignment Length: 10; Identity: 10

| Seq 1 2089 | TTTTTTAAAAT | 2099 | Seq 1 | 3182 | TTCTTTTAAA | 3191 |
| --- | --- | --- | --- | --- | --- | --- |
|  | \|\|\|\|\|\|\|\|\|\|\| |  |  |  | \|\|\|\|\|\|\|\|\|\| |  |
| Seq 2 1403 | TTTTTTAAAAT | 1413 | Seq 2 | 651 | TTCTTTTAAA | 660 |

Alignment Length: 11; Identity: 11

Alignment Length: 11; Identity: 11

| Seq 1 2089 | TTTTTTAAAAT | 2099 | Seq 1 | 3397 | AACAACAACAA | 3407 |
| --- | --- | --- | --- | --- | --- | --- |
|  | \|\|\|\|\|\|\|\|\|\|\| |  |  |  | \|\|\|\|\|\|\|\|\|\|\| |  |
| Seq 2 1534 | TTTTTTAAAAT | 1544 | Seq 2 | 260 | AACAACAACAA | 270 |

Alignment Length: 10; Identity: 10

Alignment Length: 11; Identity: 11

| Seq 1 2089 | TTTTTTAAAA | 2098 | Seq 1 | 3515 | AAAATCAAAAA | 3525 |
| --- | --- | --- | --- | --- | --- | --- |
|  | \|\|\|\|\|\|\|\|\|\| |  |  |  | \|\|\|\|\|\|\|\|\|\|\| |  |
| Seq 2 2119 | TTTTTTAAAA | 2128 | Seq 2 | 420 | AAAATCAAAAA | 430 |

Alignment Length: 11; Identity: 11

Alignment Length: 11; Identity: 11

| Seq 1 2383 | AAATATTTTTT | 2393 | Seq 1 | 3599 | TAAAATGATTA | 3609 |
| --- | --- | --- | --- | --- | --- | --- |
|  | \|\|\|\|\|\|\|\|\|\|\| |  |  |  | \|\|\|\|\|\|\|\|\|\|\| |  |
| Seq 2 912 | AAATATTTTTT | 922 | Seq 2 | 1539 | TAAAATGATTA | 1549 |

Alignment Length: 11; Identity: 11

Alignment Length: 10; Identity: 10

| Seq 1 2653 | AAAAATATTTT | 2663 | Seq 1 | 3661 | ATTTTTTAAA | 3670 |
| --- | --- | --- | --- | --- | --- | --- |
|  | \|\|\|\|\|\|\|\|\|\|\| |  |  |  | \|\|\|\|\|\|\|\|\|\| |  |
| Seq 2 910 | AAAAATATTTT | 920 | Seq 2 | 2118 | ATTTTTTAAA | 2127 |

Alignment Length: 10; Identity: 10

Alignment Length: 11; Identity: 11

| Seq 1 2672 | GAAATGACAA | 2681 | Seq 1 | 3708 | AGATTTGACAT | 3718 |
| --- | --- | --- | --- | --- | --- | --- |
|  | \|\|\|\|\|\|\|\|\|\| |  |  |  | \|\|\|\|\|\|\|\|\|\|\| |  |
| Seq 2 1131 | GAAATGACAA | 1140 | Seq 2 | 375 | AGATTTGACAT | 385 |

Alignment Length: 11; Identity: 11

Alignment Length: 11; Identity: 11

| Seq 1 2737 | ATTTTTGAAAT | 2747 | Seq 1 | 3792 | GGAAAAAAAAT | 3802 |
| --- | --- | --- | --- | --- | --- | --- |
|  | \|\|\|\|\|\|\|\|\|\|\| |  |  |  | \|\|\|\|\|\|\|\|\|\|\| |  |
| Seq 2 1086 | ATTTTTGAAAT | 1096 | Seq 2 | 625 | GGAAAAAAAAT | 635 |

Alignment Length: 11; Identity: 11

Alignment Length: 10; Identity: 10

| Seq 1 2775 | GAAATTTTTTT | 2785 | Seq 1 | 3921 | TTTCTTTTTT | 3930 |
| --- | --- | --- | --- | --- | --- | --- |
|  | \|\|\|\|\|\|\|\|\|\|\| |  |  |  | \|\|\|\|\|\|\|\|\|\| |  |
| Seq 2 549 | GAAATTTTTTT | 559 | Seq 2 | 1594 | TTTCTTTTTT | 1603 |

Alignment Length: 10; Identity: 10

**OPPOSITE STRAND**

| Seq 1 2777 | AATTTTTTTC | 2786 Alignment Length: 12; Identity: 12 | | | | |
| --- | --- | --- | --- | --- | --- | --- |
| Seq 2 812 | \|\|\|\|\|\|\|\|\|\|  AATTTTTTTC | 821 | Seq 1  Seq 2 | 3873  1512 | TTTTAAAACATT  \|\|\|\|\|\|\|\|\|\|\|\| TTTTAAAACATT | 3862  1523 |

Alignment Length: 10; Identity: 10

Seq 1 2777 AATTTTTTTC 2786

Alignment Length: 10; Identity: 10

|  | \|\|\|\|\|\|\|\|\|\| |  | Seq 1 3848 | AAATTTATAA | 3839 |
| --- | --- | --- | --- | --- | --- |
| Seq 2 2058 | AATTTTTTTC | 2067 |  | \|\|\|\|\|\|\|\|\|\| |  |
|  |  |  | Seq 2 946 | AAATTTATAA | 955 |

**S7 Figure, continued.** Motifs with identity between *C. elegans* and orthologous *unc-25* upstream sequences.

Alignment Length: 11; Identity: 11

Alignment Length: 10; Identity: 10

| Seq 1 3804 | AAATTTTTTTT | 3794 | Seq 1 | 3282 | AATCTTATTG | 3273 |
| --- | --- | --- | --- | --- | --- | --- |
|  | \|\|\|\|\|\|\|\|\|\|\| |  |  |  | \|\|\|\|\|\|\|\|\|\| |  |
| Seq 2 550 | AAATTTTTTTT | 560 | Seq 2 | 2006 | AATCTTATTG | 2015 |

Alignment Length: 10; Identity: 10

Alignment Length: 10; Identity: 10

| Seq 1 3803 | AATTTTTTTT | 3794 | Seq 1 | 3210 | TCACTTTTAA | 3201 |
| --- | --- | --- | --- | --- | --- | --- |
|  | \|\|\|\|\|\|\|\|\|\| |  |  |  | \|\|\|\|\|\|\|\|\|\| |  |
| Seq 2 96 | AATTTTTTTT | 105 | Seq 2 | 2945 | TCACTTTTAA | 2954 |

Alignment Length: 10; Identity: 10

Alignment Length: 10; Identity: 10

| Seq 1 3803 | AATTTTTTTT | 3794 | Seq 1 | 2890 | TTCCAAAAAT | 2881 |
| --- | --- | --- | --- | --- | --- | --- |
|  | \|\|\|\|\|\|\|\|\|\| |  |  |  | \|\|\|\|\|\|\|\|\|\| |  |
| Seq 2 1399 | AATTTTTTTT | 1408 | Seq 2 | 105 | TTCCAAAAAT | 114 |

Alignment Length: 10; Identity: 10

Alignment Length: 10; Identity: 10

| Seq 1 3802 | ATTTTTTTTC | 3793 | Seq 1 | 2853 | TCGAATTTTT | 2844 |
| --- | --- | --- | --- | --- | --- | --- |
|  | \|\|\|\|\|\|\|\|\|\| |  |  |  | \|\|\|\|\|\|\|\|\|\| |  |
| Seq 2 1584 | ATTTTTTTTC | 1593 | Seq 2 | 135 | TCGAATTTTT | 144 |

Alignment Length: 12; Identity: 12

Alignment Length: 10; Identity: 10

| Seq 1 3801 | TTTTTTTTCCAA | 3790 | Seq 1 | 2851 | GAATTTTTCA | 2842 |
| --- | --- | --- | --- | --- | --- | --- |
|  | \|\|\|\|\|\|\|\|\|\|\|\| |  |  |  | \|\|\|\|\|\|\|\|\|\| |  |
| Seq 2 99 | TTTTTTTTCCAA | 110 | Seq 2 | 2698 | GAATTTTTCA | 2707 |

Alignment Length: 12; Identity: 12

Alignment Length: 10; Identity: 10

| Seq 1 3670 | TTTAAAAAATAA | 3659 | Seq 1 | 2682 | TTTGTCATTT | 2673 |
| --- | --- | --- | --- | --- | --- | --- |
|  | \|\|\|\|\|\|\|\|\|\|\|\| |  |  |  | \|\|\|\|\|\|\|\|\|\| |  |
| Seq 2 1332 | TTTAAAAAATAA | 1343 | Seq 2 | 1660 | TTTGTCATTT | 1669 |

Alignment Length: 13; Identity: 13

Alignment Length: 11; Identity: 11

| Seq 1 3653 | TGTTTTCTATTTC | 3641 | Seq 1 | 2663 | AAAATATTTTT | 2653 |
| --- | --- | --- | --- | --- | --- | --- |
|  | \|\|\|\|\|\|\|\|\|\|\|\|\| |  |  |  | \|\|\|\|\|\|\|\|\|\|\| |  |
| Seq 2 1557 | TGTTTTCTATTTC | 1569 | Seq 2 | 911 | AAAATATTTTT | 921 |

Alignment Length: 11; Identity: 11

Alignment Length: 11; Identity: 11

| Seq 1 3618 | GATTATTATTA | 3608 | Seq 1 | 2651 | TTTAATTTTCA | 2641 |
| --- | --- | --- | --- | --- | --- | --- |
|  | \|\|\|\|\|\|\|\|\|\|\| |  |  |  | \|\|\|\|\|\|\|\|\|\|\| |  |
| Seq 2 1545 | GATTATTATTA | 1555 | Seq 2 | 2937 | TTTAATTTTCA | 2947 |

Alignment Length: 10; Identity: 10

Alignment Length: 12; Identity: 12

| Seq 1 3617 | ATTATTATTA | 3608 | Seq 1 | 2473 | AATAAAATAAAA | 2462 |
| --- | --- | --- | --- | --- | --- | --- |
|  | \|\|\|\|\|\|\|\|\|\| |  |  |  | \|\|\|\|\|\|\|\|\|\|\|\| |  |
| Seq 2 1742 | ATTATTATTA | 1751 | Seq 2 | 2275 | AATAAAATAAAA | 2286 |

Alignment Length: 10; Identity: 10

Alignment Length: 11; Identity: 11

| Seq 1 3617 | ATTATTATTA | 3608 | Seq 1 | 2468 | AATAAAATAAA | 2458 |
| --- | --- | --- | --- | --- | --- | --- |
|  | \|\|\|\|\|\|\|\|\|\| |  |  |  | \|\|\|\|\|\|\|\|\|\|\| |  |
| Seq 2 1745 | ATTATTATTA | 1754 | Seq 2 | 2275 | AATAAAATAAA | 2285 |

Alignment Length: 10; Identity: 10

Alignment Length: 10; Identity: 10

| Seq 1 3617 | ATTATTATTA | 3608 | Seq 1 | 2395 | TTAAAAAATA | 2386 |
| --- | --- | --- | --- | --- | --- | --- |
|  | \|\|\|\|\|\|\|\|\|\| |  |  |  | \|\|\|\|\|\|\|\|\|\| |  |
| Seq 2 1748 | ATTATTATTA | 1757 | Seq 2 | 1333 | TTAAAAAATA | 1342 |

Alignment Length: 12; Identity: 12

Alignment Length: 11; Identity: 11

| Seq 1 3528 | TAATTTTTGATT | 3517 | Seq 1 2393 | AAAAAATATTT | 2383 |
| --- | --- | --- | --- | --- | --- |
|  | \|\|\|\|\|\|\|\|\|\|\|\| |  |  | \|\|\|\|\|\|\|\|\|\|\| |  |
| Seq 2 2462 | TAATTTTTGATT | 2473 | Seq 2 909 | AAAAAATATTT | 919 |

Alignment Length: 10; Identity: 10

Alignment Length: 10; Identity: 10

| Seq 1 3301 | AAATTTCTAA | 3292 | Seq 1 | 2370 | TTTTTCAATT | 2361 |
| --- | --- | --- | --- | --- | --- | --- |
|  | \|\|\|\|\|\|\|\|\|\| |  |  |  | \|\|\|\|\|\|\|\|\|\| |  |
| Seq 2 117 | AAATTTCTAA | 126 | Seq 2 | 1393 | TTTTTCAATT | 1402 |

**S7 Figure, continued.** Motifs with identity between *C. elegans* and orthologous *unc-25* upstream sequences.

Alignment Length: 10; Identity: 10

Alignment Length: 11; Identity: 11

| Seq 1 2370 | TTTTTCAATT | 2361 | Seq 1 | 1160 | ACAATTTTTTT | 1150 |
| --- | --- | --- | --- | --- | --- | --- |
|  | \|\|\|\|\|\|\|\|\|\| |  |  |  | \|\|\|\|\|\|\|\|\|\|\| |  |
| Seq 2 2666 | TTTTTCAATT | 2675 | Seq 2 | 2056 | ACAATTTTTTT | 2066 |

Alignment Length: 10; Identity: 10

Alignment Length: 11; Identity: 11

| Seq 1 2370 | TTTTTCAATT | 2361 | Seq 1 | 1159 | CAATTTTTTTT | 1149 |
| --- | --- | --- | --- | --- | --- | --- |
|  | \|\|\|\|\|\|\|\|\|\| |  |  |  | \|\|\|\|\|\|\|\|\|\|\| |  |
| Seq 2 2701 | TTTTTCAATT | 2710 | Seq 2 | 1398 | CAATTTTTTTT | 1408 |

Alignment Length: 10; Identity: 10

Alignment Length: 10; Identity: 10

| Seq 1 2269 | ATAGCATAAA | 2260 | Seq 1 | 1158 | AATTTTTTTT | 1149 |
| --- | --- | --- | --- | --- | --- | --- |
|  | \|\|\|\|\|\|\|\|\|\| |  |  |  | \|\|\|\|\|\|\|\|\|\| |  |
| Seq 2 2970 | ATAGCATAAA | 2979 | Seq 2 | 96 | AATTTTTTTT | 105 |

Alignment Length: 12; Identity: 12

Alignment Length: 11; Identity: 11

| Seq 1 2176 | TAAATTATTTTT | 2165 | Seq 1 | 1158 | AATTTTTTTTG | 1148 |
| --- | --- | --- | --- | --- | --- | --- |
|  | \|\|\|\|\|\|\|\|\|\|\|\| |  |  |  | \|\|\|\|\|\|\|\|\|\|\| |  |
| Seq 2 1578 | TAAATTATTTTT | 1589 | Seq 2 | 551 | AATTTTTTTTG | 561 |

Alignment Length: 10; Identity: 10

Alignment Length: 10; Identity: 10

| Seq 1 2171 | TATTTTTATT | 2162 | Seq 1 | 1133 | ATCGATTTTT | 1124 |
| --- | --- | --- | --- | --- | --- | --- |
|  | \|\|\|\|\|\|\|\|\|\| |  |  |  | \|\|\|\|\|\|\|\|\|\| |  |
| Seq 2 2356 | TATTTTTATT | 2365 | Seq 2 | 481 | ATCGATTTTT | 490 |

Alignment Length: 10; Identity: 10

Alignment Length: 10; Identity: 10

| Seq 1 2098 | TTTTAAAAAA | 2089 | Seq 1 | 1133 | ATCGATTTTT | 1124 |
| --- | --- | --- | --- | --- | --- | --- |
|  | \|\|\|\|\|\|\|\|\|\| |  |  |  | \|\|\|\|\|\|\|\|\|\| |  |
| Seq 2 904 | TTTTAAAAAA | 913 | Seq 2 | 692 | ATCGATTTTT | 701 |

Alignment Length: 10; Identity: 10

Alignment Length: 10; Identity: 10

| Seq 1 2098 | TTTTAAAAAA | 2089 | Seq 1 | 1133 | ATCGATTTTT | 1124 |
| --- | --- | --- | --- | --- | --- | --- |
|  | \|\|\|\|\|\|\|\|\|\| |  |  |  | \|\|\|\|\|\|\|\|\|\| |  |
| Seq 2 1331 | TTTTAAAAAA | 1340 | Seq 2 | 2114 | ATCGATTTTT | 2123 |

Alignment Length: 10; Identity: 10

| Seq 1 2098 | TTTTAAAAAA | 2089 |  |  | \|\|\|\|\|\|\|\|\|\| |  |
| --- | --- | --- | --- | --- | --- | --- |
|  | \|\|\|\|\|\|\|\|\|\| |  | Seq 2 | 675 | GTTTTTTTTT | 684 |
| Seq 2 2477 | TTTTAAAAAA | 2486 |  |  |  |  |

Alignment Length: 10; Identity: 10

Seq 1 1071 GTTTTTTTTT 1062

Alignment Length: 11; Identity: 11

| Seq 1 2027 | AAAACCCAAAT | 2017 |  |  | \|\|\|\|\|\|\|\|\|\| |  |
| --- | --- | --- | --- | --- | --- | --- |
|  | \|\|\|\|\|\|\|\|\|\|\| |  | Seq 2 | 487 | TTTTTTTTTG | 496 |
| Seq 2 767 | AAAACCCAAAT | 777 |  |  |  |  |

Alignment Length: 10; Identity: 10

Seq 1 1070 TTTTTTTTTG 1061

Alignment Length: 10; Identity: 10

| Seq 1 1812 | CTTTTAATTT | 1803 |  |  | \|\|\|\|\|\|\|\|\|\| |  |
| --- | --- | --- | --- | --- | --- | --- |
|  | \|\|\|\|\|\|\|\|\|\| |  | Seq 2 | 677 | TTTTTTTTTG | 686 |
| Seq 2 2935 | CTTTTAATTT | 2944 |  |  |  |  |

Alignment Length: 10; Identity: 10

Seq 1 1070 TTTTTTTTTG 1061

Alignment Length: 10; Identity: 10

| Seq 1 1633 | TTAGTTTTTA | 1624 |  |  | \|\|\|\|\|\|\|\|\|\| |  |
| --- | --- | --- | --- | --- | --- | --- |
|  | \|\|\|\|\|\|\|\|\|\| |  | Seq 2 | 1598 | TTTTTTTTTG | 1607 |
| Seq 2 2472 | TTAGTTTTTA | 2481 |  |  |  |  |

Alignment Length: 10; Identity: 10

Seq 1 1070 TTTTTTTTTG 1061

Alignment Length: 10; Identity: 10

Seq 1 1480 AATATAAAAA 1471

||||||||||

Seq 2 2378 AATATAAAAA 2387

Alignment Length: 10; Identity: 10

| Seq 1 1466 | ATTATTGTTT | 1457 |  |  | \|\|\|\|\|\|\|\|\|\| |  |
| --- | --- | --- | --- | --- | --- | --- |
|  | \|\|\|\|\|\|\|\|\|\| |  | Seq 2 | 1295 | TATTTTGAAA | 1304 |
| Seq 2 1552 | ATTATTGTTT | 1561 |  |  |  |  |

Alignment Length: 10; Identity: 10

Seq 1 1001 TTTTTTTCAA 992

||||||||||

Seq 2 1612 TTTTTTTCAA 1621

Alignment Length: 10; Identity: 10

Seq 1 928 TATTTTGAAA 919

**S7 Figure, continued.** Motifs with identity between *C. elegans* and orthologous *unc-25* upstream sequences.

Alignment Length: 11; Identity: 11

| Seq 1 877 | CTTTTAATTTT | 867 |
| --- | --- | --- |
|  | \|\|\|\|\|\|\|\|\|\|\| |  |
| Seq 2 2935 | CTTTTAATTTT | 2945 |

Alignment Length: 11; Identity: 11

| Seq 1 868 | TTATTTTTTTT | 858 |
| --- | --- | --- |
|  | \|\|\|\|\|\|\|\|\|\|\| |  |
| Seq 2 1582 | TTATTTTTTTT | 1592 |

Alignment Length: 10; Identity: 10

| Seq 1 867 | TATTTTTTTT | 858 |
| --- | --- | --- |
|  | \|\|\|\|\|\|\|\|\|\| |  |
| Seq 2 915 | TATTTTTTTT | 924 |

Alignment Length: 10; Identity: 10

| Seq 1 866 | ATTTTTTTTA | 857 |
| --- | --- | --- |
|  | \|\|\|\|\|\|\|\|\|\| |  |
| Seq 2 1400 | ATTTTTTTTA | 1409 |

Alignment Length: 10; Identity: 10

| Seq 1 866 | ATTTTTTTTA | 857 |
| --- | --- | --- |
|  | \|\|\|\|\|\|\|\|\|\| |  |
| Seq 2 2369 | ATTTTTTTTA | 2378 |

Alignment Length: 10; Identity: 10

| Seq 1 838 | TTGTTCTGAT | 829 |
| --- | --- | --- |
|  | \|\|\|\|\|\|\|\|\|\| |  |
| Seq 2 1669 | TTGTTCTGAT | 1678 |

Alignment Length: 10; Identity: 10

| Seq 1 755 | ATTTTGAAAA | 746 |
| --- | --- | --- |
|  | \|\|\|\|\|\|\|\|\|\| |  |
| Seq 2 1296 | ATTTTGAAAA | 1305 |

Alignment Length: 10; Identity: 10

| Seq 1 494 | AAAAAATAAT | 485 |
| --- | --- | --- |
|  | \|\|\|\|\|\|\|\|\|\| |  |
| Seq 2 1335 | AAAAAATAAT | 1344 |

Alignment Length: 10; Identity: 10

| Seq 1 320 | AATATAAAAA | 311 |
| --- | --- | --- |
|  | \|\|\|\|\|\|\|\|\|\| |  |
| Seq 2 2378 | AATATAAAAA | 2387 |

Alignment Length: 10; Identity: 10

| Seq 1 315 | AAAAATTTAA | 306 |
| --- | --- | --- |
|  | \|\|\|\|\|\|\|\|\|\| |  |
| Seq 2 109 | AAAAATTTAA | 118 |

Alignment Length: 12; Identity: 12

| Seq 1 133 | AAAATAAGAAAA | 122 |
| --- | --- | --- |
|  | \|\|\|\|\|\|\|\|\|\|\|\| |  |
| Seq 2 930 | AAAATAAGAAAA | 941 |

Alignment Length: 10; Identity: 10

| Seq 1 132 | AAATAAGAAA | 123 |
| --- | --- | --- |
|  | \|\|\|\|\|\|\|\|\|\| |  |
| Seq 2 77 | AAATAAGAAA | 86 |
