# Supplementary material for: Phylum-Level Conservation of Regulatory Information in Nematodes despite Extensive Non-coding Sequence Divergence
Source: PLoS Genet. 2015 May 28;11(5):e1005268. doi: 10.1371/journal.pgen.1005268 (PMC4447282; doi:10.1371/journal.pgen.1005268)
Supplement: S8 Fig — Motifs with identity between C. elegans and orthologous mec-3 upstream sequences. All blocks of sequence identity in window sizes shown for each comparison with positions within the upstream non-coding sequence. (DOCX) [file pgen.1005268.s008.docx]

**S8 Figure. Motifs with identity between *C. elegans* and orthologous *mec-3* upstream sequences.** Motifs with identity between *C. elegans* and orthologous *mec-3* upstream sequences. All blocks of sequence identity in window sizes shown for each comparison with positions within the upstream non-coding sequence.

**C. briggsae/C. elegans mec-3, 11bp window**

Alignment Length: 11; Identity: 11

|  | | Seq 1 | 826 | TTAGAATTTTA | 836 |
| --- | --- | --- | --- | --- | --- |
| **seq 1:** | **cbrmec3** |  |  | \|\|\|\|\|\|\|\|\|\|\| |  |
| **seq 2:** | **celmec3** | Seq 2 | 822 | TTAGAATTTTA | 832 |

Alignment Length: 11; Identity: 11

| Seq 1 110 | AAGGAGAAAAT | 120 Alignment Length: 11; Identity: 11 | | | | |
| --- | --- | --- | --- | --- | --- | --- |
|  | \|\|\|\|\|\|\|\|\|\|\| |  | Seq 1 | 834 | TTAGAATTTTA | 844 |
| Seq 2 1969 | AAGGAGAAAAT | 1979 |  |  | \|\|\|\|\|\|\|\|\|\|\| |  |
|  |  |  | Seq 2 | 822 | TTAGAATTTTA | 832 |
| Alignment Length: 11; Identity: 11 | | | | | | |
| Seq 1 209 | CTTCATTTTTT | 219 Alignment Length: 11; Identity: 11 | | | | |
|  | \|\|\|\|\|\|\|\|\|\|\| |  | Seq 1 | 846 | AATTTTGAAAT | 856 |
| Seq 2 443 | CTTCATTTTTT | 453 |  |  | \|\|\|\|\|\|\|\|\|\|\| |  |
|  |  |  | Seq 2 | 834 | AATTTTGAAAT | 844 |

Alignment Length: 14; Identity: 14

Seq 1 409 GTCAATCTGAATTT 422

|||||||||||||| Seq 2 354 GTCAATCTGAATTT 367

Alignment Length: 11; Identity: 11

| Seq 1 418 | AATTTTTAATG | 428 |
| --- | --- | --- |
|  | \|\|\|\|\|\|\|\|\|\|\| |  |
| Seq 2 2383 | AATTTTTAATG | 2393 |

Alignment Length: 11; Identity: 11

Alignment Length: 14; Identity: 14

Seq 1 847 ATTTTGAAATTTTA 860

||||||||||||||

Seq 2 1153 ATTTTGAAATTTTA 1166

Alignment Length: 11; Identity: 11

Seq 1 858 TTAGAATTTTA 868

|||||||||||

Seq 2 822 TTAGAATTTTA 832

| Seq 1 639 | AAAATCTGAAT | 649 Alignment Length: 11; Identity: 11 | | | | |
| --- | --- | --- | --- | --- | --- | --- |
|  | \|\|\|\|\|\|\|\|\|\|\| |  | Seq 1 | 866 | TTAGAATTTTA | 876 |
| Seq 2 2402 | AAAATCTGAAT | 2412 |  |  | \|\|\|\|\|\|\|\|\|\|\| |  |
|  |  |  | Seq 2 | 822 | TTAGAATTTTA | 832 |
| Alignment Length: 11; Identity: 11 | | | | | | |
| Seq 1 654 | GAAAATCTGAA | 664 Alignment Length: 11; Identity: 11 | | | | |
|  | \|\|\|\|\|\|\|\|\|\|\| |  | Seq 1 | 874 | TTAGAATTTTA | 884 |
| Seq 2 2401 | GAAAATCTGAA | 2411 |  |  | \|\|\|\|\|\|\|\|\|\|\| |  |
|  |  |  | Seq 2 | 822 | TTAGAATTTTA | 832 |
| Alignment Length: 12; Identity: 12 | | | | | | |
| Seq 1 734 | AATTTTGAAATC | 745 Alignment Length: 11; Identity: 11 | | | | |
|  | \|\|\|\|\|\|\|\|\|\|\|\| |  | Seq 1 | 882 | TTAGAATTTTA | 892 |
| Seq 2 834 | AATTTTGAAATC | 845 |  |  | \|\|\|\|\|\|\|\|\|\|\| |  |
|  |  |  | Seq 2 | 822 | TTAGAATTTTA | 832 |
| Alignment Length: 11; Identity: 11 | | | | | | |
| Seq 1 786 | TTAGAATTTTA | 796 Alignment Length: 11; Identity: 11 | | | | |
|  | \|\|\|\|\|\|\|\|\|\|\| |  | Seq 1 | 890 | TTAGAATTTTA | 900 |
| Seq 2 822 | TTAGAATTTTA | 832 |  |  | \|\|\|\|\|\|\|\|\|\|\| |  |
|  |  |  | Seq 2 | 822 | TTAGAATTTTA | 832 |
| Alignment Length: 11; Identity: 11 | | | | | | |
| Seq 1 794 | TTAGAATTTTA | 804 Alignment Length: 11; Identity: 11 | | | | |
|  | \|\|\|\|\|\|\|\|\|\|\| |  | Seq 1 | 898 | TTAGAATTTTA | 908 |
| Seq 2 822 | TTAGAATTTTA | 832 |  |  | \|\|\|\|\|\|\|\|\|\|\| |  |
|  |  |  | Seq 2 | 822 | TTAGAATTTTA | 832 |
| Alignment Length: 11; Identity: 11 | | | | | | |
| Seq 1 802 | TTAGAATTTTA | 812 Alignment Length: 11; Identity: 11 | | | | |
|  | \|\|\|\|\|\|\|\|\|\|\| |  | Seq 1 | 906 | TTAGAATTTTA | 916 |
| Seq 2 822 | TTAGAATTTTA | 832 |  |  | \|\|\|\|\|\|\|\|\|\|\| |  |
|  |  |  | Seq 2 | 822 | TTAGAATTTTA | 832 |
| Alignment Length: 11; Identity: 11 | | | | | | |
| Seq 1 810 | TTAGAATTTTA | 820 Alignment Length: 11; Identity: 11 | | | | |
|  | \|\|\|\|\|\|\|\|\|\|\| |  | Seq 1 | 914 | TTAGAATTTTA | 924 |
| Seq 2 822 | TTAGAATTTTA | 832 |  |  | \|\|\|\|\|\|\|\|\|\|\| |  |
|  |  |  | Seq 2 | 822 | TTAGAATTTTA | 832 |
| Alignment Length: 11; Identity: 11 | | | | | | |
| Seq 1 818 | TTAGAATTTTA | 828 Alignment Length: 11; Identity: 11 | | | | |
|  | \|\|\|\|\|\|\|\|\|\|\| |  | Seq 1 | 922 | TTAGAATTTTA | 932 |
| Seq 2 822 | TTAGAATTTTA | 832 |  |  | \|\|\|\|\|\|\|\|\|\|\| |  |
|  |  |  | Seq 2 | 822 | TTAGAATTTTA | 832 |

**S8 Figure, continued.** Motifs with identity between *C. elegans* and orthologous *mec-3* upstream sequences.

Alignment Length: 11; Identity: 11

Alignment Length: 11; Identity: 11

| Seq 1 930 | TTAGAATTTTA | 940 | Seq 1 | 1034 | TTAGAATTTTA | 1044 |
| --- | --- | --- | --- | --- | --- | --- |
|  | \|\|\|\|\|\|\|\|\|\|\| |  |  |  | \|\|\|\|\|\|\|\|\|\|\| |  |
| Seq 2 822 | TTAGAATTTTA | 832 | Seq 2 | 822 | TTAGAATTTTA | 832 |

Alignment Length: 11; Identity: 11

Alignment Length: 11; Identity: 11

| Seq 1 938 | TTAGAATTTTA | 948 | Seq 1 | 1042 | TTAGAATTTTA | 1052 |
| --- | --- | --- | --- | --- | --- | --- |
|  | \|\|\|\|\|\|\|\|\|\|\| |  |  |  | \|\|\|\|\|\|\|\|\|\|\| |  |
| Seq 2 822 | TTAGAATTTTA | 832 | Seq 2 | 822 | TTAGAATTTTA | 832 |

Alignment Length: 11; Identity: 11

Alignment Length: 11; Identity: 11

| Seq 1 946 | TTAGAATTTTA | 956 | Seq 1 | 1050 | TTAGAATTTTA | 1060 |
| --- | --- | --- | --- | --- | --- | --- |
|  | \|\|\|\|\|\|\|\|\|\|\| |  |  |  | \|\|\|\|\|\|\|\|\|\|\| |  |
| Seq 2 822 | TTAGAATTTTA | 832 | Seq 2 | 822 | TTAGAATTTTA | 832 |

Alignment Length: 11; Identity: 11

Alignment Length: 11; Identity: 11

| Seq 1 954 | TTAGAATTTTA | 964 | Seq 1 | 1058 | TTAGAATTTTA | 1068 |
| --- | --- | --- | --- | --- | --- | --- |
|  | \|\|\|\|\|\|\|\|\|\|\| |  |  |  | \|\|\|\|\|\|\|\|\|\|\| |  |
| Seq 2 822 | TTAGAATTTTA | 832 | Seq 2 | 822 | TTAGAATTTTA | 832 |

Alignment Length: 11; Identity: 11

Alignment Length: 11; Identity: 11

| Seq 1 962 | TTAGAATTTTA | 972 | Seq 1 | 1070 | AATTTTGAAAT | 1080 |
| --- | --- | --- | --- | --- | --- | --- |
|  | \|\|\|\|\|\|\|\|\|\|\| |  |  |  | \|\|\|\|\|\|\|\|\|\|\| |  |
| Seq 2 822 | TTAGAATTTTA | 832 | Seq 2 | 834 | AATTTTGAAAT | 844 |

Alignment Length: 11; Identity: 11

Alignment Length: 14; Identity: 14

| Seq 1 970 | TTAGAATTTTA | 980 | Seq 1 | 1071 | ATTTTGAAATTTTA 1084 |
| --- | --- | --- | --- | --- | --- |
|  | \|\|\|\|\|\|\|\|\|\|\| |  |  |  | \|\|\|\|\|\|\|\|\|\|\|\|\|\| |
| Seq 2 822 | TTAGAATTTTA | 832 | Seq 2 | 1153 | ATTTTGAAATTTTA 1166 |

Alignment Length: 11; Identity: 11

Alignment Length: 11; Identity: 11

| Seq 1 978 | TTAGAATTTTA | 988 | Seq 1 | 1082 | TTAGAATTTTA | 1092 |
| --- | --- | --- | --- | --- | --- | --- |
|  | \|\|\|\|\|\|\|\|\|\|\| |  |  |  | \|\|\|\|\|\|\|\|\|\|\| |  |
| Seq 2 822 | TTAGAATTTTA | 832 | Seq 2 | 822 | TTAGAATTTTA | 832 |

Alignment Length: 11; Identity: 11

Alignment Length: 11; Identity: 11

| Seq 1 986 | TTAGAATTTTA | 996 | Seq 1 | 1090 | TTAGAATTTTA | 1100 |
| --- | --- | --- | --- | --- | --- | --- |
|  | \|\|\|\|\|\|\|\|\|\|\| |  |  |  | \|\|\|\|\|\|\|\|\|\|\| |  |
| Seq 2 822 | TTAGAATTTTA | 832 | Seq 2 | 822 | TTAGAATTTTA | 832 |

Alignment Length: 11; Identity: 11

Alignment Length: 12; Identity: 12

| Seq 1 994 | TTAGAATTTTA | 1004 | Seq 1 | 1271 | AAAATTTTGAAA | 1282 |
| --- | --- | --- | --- | --- | --- | --- |
|  | \|\|\|\|\|\|\|\|\|\|\| |  |  |  | \|\|\|\|\|\|\|\|\|\|\|\| |  |
| Seq 2 822 | TTAGAATTTTA | 832 | Seq 2 | 832 | AAAATTTTGAAA | 843 |

Alignment Length: 11; Identity: 11

Alignment Length: 11; Identity: 11

| Seq 1 1002 | TTAGAATTTTA | 1012 | Seq 1 | 1444 | GTTTTCAAATT | 1454 |
| --- | --- | --- | --- | --- | --- | --- |
|  | \|\|\|\|\|\|\|\|\|\|\| |  |  |  | \|\|\|\|\|\|\|\|\|\|\| |  |
| Seq 2 822 | TTAGAATTTTA | 832 | Seq 2 | 591 | GTTTTCAAATT | 601 |

Alignment Length: 11; Identity: 11

Alignment Length: 12; Identity: 12

| Seq 1 1010 | TTAGAATTTTA | 1020 | Seq 1 | 1447 | TTCAAATTTTTA | 1458 |
| --- | --- | --- | --- | --- | --- | --- |
|  | \|\|\|\|\|\|\|\|\|\|\| |  |  |  | \|\|\|\|\|\|\|\|\|\|\|\| |  |
| Seq 2 822 | TTAGAATTTTA | 832 | Seq 2 | 2518 | TTCAAATTTTTA | 2529 |

Alignment Length: 11; Identity: 11

Alignment Length: 11; Identity: 11

| Seq 1 1018 | TTAGAATTTTA | 1028 | Seq 1 1685 | ATTTATCACCG | 1695 |
| --- | --- | --- | --- | --- | --- |
|  | \|\|\|\|\|\|\|\|\|\|\| |  |  | \|\|\|\|\|\|\|\|\|\|\| |  |
| Seq 2 822 | TTAGAATTTTA | 832 | Seq 2 1344 | ATTTATCACCG | 1354 |

Alignment Length: 11; Identity: 11

Alignment Length: 11; Identity: 11

| Seq 1 1026 | TTAGAATTTTA | 1036 | Seq 1 | 1942 | TAGAAATTGAC | 1952 |
| --- | --- | --- | --- | --- | --- | --- |
|  | \|\|\|\|\|\|\|\|\|\|\| |  |  |  | \|\|\|\|\|\|\|\|\|\|\| |  |
| Seq 2 822 | TTAGAATTTTA | 832 | Seq 2 | 1520 | TAGAAATTGAC | 1530 |

**S8 Figure, continued.** Motifs with identity between *C. elegans* and orthologous *mec-3* upstream sequences.

Alignment Length: 12; Identity: 12

Alignment Length: 11; Identity: 11

| Seq 1 2013 | AGAATTTTAAAA | 2024 | Seq 1 | 5081 | ACACACTTTCT | 5091 |
| --- | --- | --- | --- | --- | --- | --- |
|  | \|\|\|\|\|\|\|\|\|\|\|\| |  |  |  | \|\|\|\|\|\|\|\|\|\|\| |  |
| Seq 2 824 | AGAATTTTAAAA | 835 | Seq 2 | 2702 | ACACACTTTCT | 2712 |

Alignment Length: 12; Identity: 12

Alignment Length: 17; Identity: 17

| Seq 1 2204 | TTTTAAAATTTT | 2215 | Seq 1 | 5093 | TCATTCGAAATGCATTG | 5109 |
| --- | --- | --- | --- | --- | --- | --- |
|  | \|\|\|\|\|\|\|\|\|\|\|\| |  |  |  | \|\|\|\|\|\|\|\|\|\|\|\|\|\|\|\|\| |  |
| Seq 2 828 | TTTTAAAATTTT | 839 | Seq 2 | 2590 | TCATTCGAAATGCATTG | 2606 |

Alignment Length: 12; Identity: 12

Alignment Length: 15; Identity: 15

| Seq 1 2226 | CTGTCAATCAAA | 2237 | Seq 1 | 5121 | ATCGACCGAAAAACA | 5135 |
| --- | --- | --- | --- | --- | --- | --- |
|  | \|\|\|\|\|\|\|\|\|\|\|\| |  |  |  | \|\|\|\|\|\|\|\|\|\|\|\|\|\|\| |  |
| Seq 2 1898 | CTGTCAATCAAA | 1909 | Seq 2 | 2617 | ATCGACCGAAAAACA | 2631 |

Alignment Length: 11; Identity: 11

Alignment Length: 22; Identity: 22

| Seq 1 2445 | ATTTATCAAAA | 2455 | Seq 1 | 5224 | GAAATGCATCTATTATCGTCAC 5245 |
| --- | --- | --- | --- | --- | --- |
|  | \|\|\|\|\|\|\|\|\|\|\| |  |  |  | \|\|\|\|\|\|\|\|\|\|\|\|\|\|\|\|\|\|\|\|\|\| |
| Seq 2 1669 | ATTTATCAAAA | 1679 | Seq 2 | 2723 | GAAATGCATCTATTATCGTCAC 2744 |

Alignment Length: 11; Identity: 11

Alignment Length: 19; Identity: 19

| Seq 1 2460 | AAAAACATTTT | 2470 | Seq 1 | 5277 | GCGCACATTAATAATCGAT | 5295 |
| --- | --- | --- | --- | --- | --- | --- |
|  | \|\|\|\|\|\|\|\|\|\|\| |  |  |  | \|\|\|\|\|\|\|\|\|\|\|\|\|\|\|\|\|\|\| |  |
| Seq 2 665 | AAAAACATTTT | 675 | Seq 2 | 2766 | GCGCACATTAATAATCGAT | 2784 |

Alignment Length: 13; Identity: 13

Alignment Length: 14; Identity: 14

| Seq 1 2554 | AGGTAGTTTTAAA | 2566 | Seq 1 | 5344 | GGGTGATTTGTTAC 5357 |
| --- | --- | --- | --- | --- | --- |
|  | \|\|\|\|\|\|\|\|\|\|\|\|\| |  |  |  | \|\|\|\|\|\|\|\|\|\|\|\|\|\| |
| Seq 2 715 | AGGTAGTTTTAAA | 727 | Seq 2 | 2846 | GGGTGATTTGTTAC 2859 |

Alignment Length: 11; Identity: 11

Alignment Length: 13; Identity: 13

| Seq 1 2612 | AGTTTTCAAAT | 2622 | Seq 1 | 5378 | ATTTCGCGCATTT 5390 |
| --- | --- | --- | --- | --- | --- |
|  | \|\|\|\|\|\|\|\|\|\|\| |  |  |  | \|\|\|\|\|\|\|\|\|\|\|\|\| |
| Seq 2 590 | AGTTTTCAAAT | 600 | Seq 2 | 2874 | ATTTCGCGCATTT 2886 |

Alignment Length: 11; Identity: 11

Seq 1 2631 TTTGAAATTTT 2641

**OPPOSITE STRAND**

Alignment Length: 11; Identity: 11

|  | \|\|\|\|\|\|\|\|\|\|\| |  | Seq 1 | 4748 | TGATAATGGGA | 4738 |
| --- | --- | --- | --- | --- | --- | --- |
| Seq 2 1155 | TTTGAAATTTT | 1165 |  |  | \|\|\|\|\|\|\|\|\|\|\| |  |
|  |  |  | Seq 2 | 124 | TGATAATGGGA | 134 |

Alignment Length: 12; Identity: 12

Seq 1 3152 ATCTCATTTTCT 3163

Alignment Length: 11; Identity: 11

|  | \|\|\|\|\|\|\|\|\|\|\|\| |  | Seq 1 | 4668 | AATTTTTTGGT | 4658 |
| --- | --- | --- | --- | --- | --- | --- |
| Seq 2 1329 | ATCTCATTTTCT | 1340 |  |  | \|\|\|\|\|\|\|\|\|\|\| |  |
|  |  |  | Seq 2 | 798 | AATTTTTTGGT | 808 |

Alignment Length: 11; Identity: 11

Seq 1 3153 TCTCATTTTCT 3163

Alignment Length: 11; Identity: 11

|  | \|\|\|\|\|\|\|\|\|\|\| |  | Seq 1 | 4466 | AAAACAAATAA | 4456 |
| --- | --- | --- | --- | --- | --- | --- |
| Seq 2 1773 | TCTCATTTTCT | 1783 |  |  | \|\|\|\|\|\|\|\|\|\|\| |  |
|  |  |  | Seq 2 | 1445 | AAAACAAATAA | 1455 |

Alignment Length: 11; Identity: 11

Seq 1 4278 AATTTTTATAA 4288

Alignment Length: 11; Identity: 11

|  | \|\|\|\|\|\|\|\|\|\|\| |  | Seq 1 | 3981 | ATCTGAATTTC | 3971 |
| --- | --- | --- | --- | --- | --- | --- |
| Seq 2 2522 | AATTTTTATAA | 2532 |  |  | \|\|\|\|\|\|\|\|\|\|\| |  |
|  |  |  | Seq 2 | 358 | ATCTGAATTTC | 368 |

Alignment Length: 16; Identity: 16

Seq 1 4480 TTTCACTGTTATTTGT 4495

Alignment Length: 12; Identity: 12

|  | \|\|\|\|\|\|\|\|\|\|\|\|\|\|\|\| | Seq 1 | 2814 | TTGGCCCGTTTA | 2803 |
| --- | --- | --- | --- | --- | --- |
| Seq 2 2896 | TTTCACTGTTATTTGT 2911 |  |  | \|\|\|\|\|\|\|\|\|\|\|\| |  |
|  |  | Seq 2 | 402 | TTGGCCCGTTTA | 413 |

Alignment Length: 11; Identity: 11

Seq 1 4875 AAGACATTTAT 4885

Alignment Length: 12; Identity: 12

|  | \|\|\|\|\|\|\|\|\|\|\| |  | Seq 1 2604 | TTTGCTTTTTTT | 2593 |
| --- | --- | --- | --- | --- | --- |
| Seq 2 1664 | AAGACATTTAT | 1674 |  | \|\|\|\|\|\|\|\|\|\|\|\| |  |
|  |  |  | Seq 2 161 | TTTGCTTTTTTT | 172 |

**S8 Figure, continued.** Motifs with identity between *C. elegans* and orthologous *mec-3* upstream sequences.

Alignment Length: 12; Identity: 12

| Seq 1 2463 | TTTTGAAATTTT | 2452 |
| --- | --- | --- |
|  | \|\|\|\|\|\|\|\|\|\|\|\| |  |
| Seq 2 1154 | TTTTGAAATTTT | 1165 |

Alignment Length: 11; Identity: 11

| Seq 1 1672 | CTTCATTTTTT | 1662 |
| --- | --- | --- |
|  | \|\|\|\|\|\|\|\|\|\|\| |  |
| Seq 2 443 | CTTCATTTTTT | 453 |

Alignment Length: 11; Identity: 11

| Seq 1 1483 | TCAGAAATTGA | 1473 |
| --- | --- | --- |
|  | \|\|\|\|\|\|\|\|\|\|\| |  |
| Seq 2 1213 | TCAGAAATTGA | 1223 |

Alignment Length: 12; Identity: 12

| Seq 1 1473 | AGAAAATCTGAA | 1462 |
| --- | --- | --- |
|  | \|\|\|\|\|\|\|\|\|\|\|\| |  |
| Seq 2 2400 | AGAAAATCTGAA | 2411 |

Alignment Length: 11; Identity: 11

| Seq 1 1429 | TCAGAAAGTGT | 1419 |
| --- | --- | --- |
|  | \|\|\|\|\|\|\|\|\|\|\| |  |
| Seq 2 1237 | TCAGAAAGTGT | 1247 |

Alignment Length: 11; Identity: 11

| Seq 1 1402 | GAAAATCTGAA | 1392 |
| --- | --- | --- |
|  | \|\|\|\|\|\|\|\|\|\|\| |  |
| Seq 2 2401 | GAAAATCTGAA | 2411 |

Alignment Length: 13; Identity: 13

Seq 1 1278 AAAATTTTGAAAT 1266

|||||||||||||

Seq 2 832 AAAATTTTGAAAT 844

Alignment Length: 11; Identity: 11

| Seq 1 1275 | ATTTTGAAATT | 1265 |
| --- | --- | --- |
|  | \|\|\|\|\|\|\|\|\|\|\| |  |
| Seq 2 1153 | ATTTTGAAATT | 1163 |

Alignment Length: 12; Identity: 12

| Seq 1 762 | AAATTTTGAAAT | 751 |
| --- | --- | --- |
|  | \|\|\|\|\|\|\|\|\|\|\|\| |  |
| Seq 2 833 | AAATTTTGAAAT | 844 |

Alignment Length: 11; Identity: 11

| Seq 1 760 | ATTTTGAAATT | 750 |
| --- | --- | --- |
|  | \|\|\|\|\|\|\|\|\|\|\| |  |
| Seq 2 1153 | ATTTTGAAATT | 1163 |

Alignment Length: 14; Identity: 14

Seq 1 642 TTTTTTTTAATTTT 629

||||||||||||||

Seq 2 1022 TTTTTTTTAATTTT 1035

Alignment Length: 11; Identity: 11

| Seq 1 639 | TTTTTAATTTT | 629 |
| --- | --- | --- |
|  | \|\|\|\|\|\|\|\|\|\|\| |  |
| Seq 2 427 | TTTTTAATTTT | 437 |

Alignment Length: 14; Identity: 14

Seq 1 227 AAATTGAAAAAAAA 214

||||||||||||||

Seq 2 1945 AAATTGAAAAAAAA 1958

**S8 Figure, continued.** Motifs with identity between *C. elegans* and orthologous *mec-3* upstream sequences.

**M. hapla/C. elegans mec-3, 10bp window**

Alignment Length: 10; Identity: 10

|  | | Seq 1 | 444 | AATTATTTTT | 453 |
| --- | --- | --- | --- | --- | --- |
| **seq 1:** | **mhamec3** |  |  | \|\|\|\|\|\|\|\|\|\| |  |
| **seq 2:** | **celmec3** | Seq 2 | 517 | AATTATTTTT | 526 |

Alignment Length: 10; Identity: 10

Seq 1 75 TCAAATCAAA 84

||||||||||

Seq 2 742 TCAAATCAAA 751

Alignment Length: 10; Identity: 10

Seq 1 141 TCAAAACAAA 150

||||||||||

Seq 2 1674 TCAAAACAAA 1683

Alignment Length: 14; Identity: 14

Seq 1 197 AATTTTAAAATTTT 210

|||||||||||||| Seq 2 826 AATTTTAAAATTTT 839

Alignment Length: 10; Identity: 10

**OPPOSITE STRAND**

Alignment Length: 10; Identity: 10

Seq 1 449 ATAATTTTTA 440

||||||||||

Seq 2 2381 ATAATTTTTA 2390

Alignment Length: 10; Identity: 10

Seq 1 210 AAAATTTTAA 201

||||||||||

Seq 2 1110 AAAATTTTAA 1119

Alignment Length: 12; Identity: 12

Seq 1 208 AATTTTAAAATT 197

||||||||||||

Seq 2 826 AATTTTAAAATT 837

| Seq 1 201 | TTAAAATTTT | 210 |
| --- | --- | --- |
|  | \|\|\|\|\|\|\|\|\|\| |  |
| Seq 2 1108 | TTAAAATTTT | 1117 |

Alignment Length: 10; Identity: 10

| Seq 1 222 | TATAGAAAAT | 231 |
| --- | --- | --- |
|  | \|\|\|\|\|\|\|\|\|\| |  |
| Seq 2 2397 | TATAGAAAAT | 2406 |

Alignment Length: 10; Identity: 10

| Seq 1 228 | AAATATTTAC | 237 |
| --- | --- | --- |
|  | \|\|\|\|\|\|\|\|\|\| |  |
| Seq 2 1806 | AAATATTTAC | 1815 |

Alignment Length: 10; Identity: 10

| Seq 1 279 | TCAGGAAGAA | 288 |
| --- | --- | --- |
|  | \|\|\|\|\|\|\|\|\|\| |  |
| Seq 2 1193 | TCAGGAAGAA | 1202 |

Alignment Length: 10; Identity: 10

| Seq 1 296 | AACAATTAAT | 305 |
| --- | --- | --- |
|  | \|\|\|\|\|\|\|\|\|\| |  |
| Seq 2 1580 | AACAATTAAT | 1589 |

Alignment Length: 10; Identity: 10

| Seq 1 408 | CTTTTTTTTT | 417 |
| --- | --- | --- |
|  | \|\|\|\|\|\|\|\|\|\| |  |
| Seq 2 165 | CTTTTTTTTT | 174 |

Alignment Length: 10; Identity: 10

| Seq 1 409 | TTTTTTTTTA | 418 |
| --- | --- | --- |
|  | \|\|\|\|\|\|\|\|\|\| |  |
| Seq 2 167 | TTTTTTTTTA | 176 |

Alignment Length: 11; Identity: 11

| Seq 1 409 | TTTTTTTTTAA | 419 |
| --- | --- | --- |
|  | \|\|\|\|\|\|\|\|\|\|\| |  |
| Seq 2 1021 | TTTTTTTTTAA | 1031 |

Alignment Length: 10; Identity: 10

| Seq 1 439 | ATAAAAATTA | 448 |
| --- | --- | --- |
|  | \|\|\|\|\|\|\|\|\|\| |  |
| Seq 2 965 | ATAAAAATTA | 974 |

**S8 Figure, continued.** Motifs with identity between *C. elegans* and orthologous *mec-3* upstream sequences.

**B. malayi/C. elegans mec-3, 10bp window**

Alignment Length: 10; Identity: 10

|  | | Seq 1 | 1163 | ATCAAATTTA | 1172 |
| --- | --- | --- | --- | --- | --- |
| **seq 1:** | **cbrmec3** |  |  | \|\|\|\|\|\|\|\|\|\| |  |
| **seq 2:** | **celmec3** | Seq 2 | 1846 | ATCAAATTTA | 1855 |

Alignment Length: 14; Identity: 14

Alignment Length: 11; Identity: 11

| Seq 1 460 | AATTTCGCGCATTT 473 | Seq 1 | 1216 | AGTTTCATTTT | 1226 |
| --- | --- | --- | --- | --- | --- |
|  | \|\|\|\|\|\|\|\|\|\|\|\|\|\| |  |  | \|\|\|\|\|\|\|\|\|\|\| |  |
| Seq 2 2873 | AATTTCGCGCATTT 2886 | Seq 2 | 1147 | AGTTTCATTTT | 1157 |

Alignment Length: 10; Identity: 10

Alignment Length: 10; Identity: 10

| Seq 1 669 | CAAATATTTA | 678 | Seq 1 | 1217 | GTTTCATTTT | 1226 |
| --- | --- | --- | --- | --- | --- | --- |
|  | \|\|\|\|\|\|\|\|\|\| |  |  |  | \|\|\|\|\|\|\|\|\|\| |  |
| Seq 2 1805 | CAAATATTTA | 1814 | Seq 2 | 1764 | GTTTCATTTT | 1773 |

Alignment Length: 10; Identity: 10

Alignment Length: 10; Identity: 10

| Seq 1 674 | ATTTATAAAA | 683 | Seq 1 | 1229 | TATTATCAAA | 1238 |
| --- | --- | --- | --- | --- | --- | --- |
|  | \|\|\|\|\|\|\|\|\|\| |  |  |  | \|\|\|\|\|\|\|\|\|\| |  |
| Seq 2 1137 | ATTTATAAAA | 1146 | Seq 2 | 469 | TATTATCAAA | 478 |

Alignment Length: 10; Identity: 10

Alignment Length: 10; Identity: 10

| Seq 1 692 | GAAAAAAAAA | 701 | Seq 1 | 1237 | AAATTTTTAT | 1246 |
| --- | --- | --- | --- | --- | --- | --- |
|  | \|\|\|\|\|\|\|\|\|\| |  |  |  | \|\|\|\|\|\|\|\|\|\| |  |
| Seq 2 1950 | GAAAAAAAAA | 1959 | Seq 2 | 2521 | AAATTTTTAT | 2530 |

Alignment Length: 10; Identity: 10

Alignment Length: 10; Identity: 10

| Seq 1 788 | TTTATTTTCT | 797 | Seq 1 | 1487 | TTTAAAATTT | 1496 |
| --- | --- | --- | --- | --- | --- | --- |
|  | \|\|\|\|\|\|\|\|\|\| |  |  |  | \|\|\|\|\|\|\|\|\|\| |  |
| Seq 2 435 | TTTATTTTCT | 444 | Seq 2 | 829 | TTTAAAATTT | 838 |

Alignment Length: 10; Identity: 10

Alignment Length: 10; Identity: 10

| Seq 1 840 | ATATTTTTGT | 849 | Seq 1 | 1496 | TACCATAATT | 1505 |
| --- | --- | --- | --- | --- | --- | --- |
|  | \|\|\|\|\|\|\|\|\|\| |  |  |  | \|\|\|\|\|\|\|\|\|\| |  |
| Seq 2 875 | ATATTTTTGT | 884 | Seq 2 | 2377 | TACCATAATT | 2386 |

Alignment Length: 10; Identity: 10

Alignment Length: 11; Identity: 11

| Seq 1 849 | TAATTATTTT | 858 | Seq 1 | 1523 | ACCACCAAAAA | 1533 |
| --- | --- | --- | --- | --- | --- | --- |
|  | \|\|\|\|\|\|\|\|\|\| |  |  |  | \|\|\|\|\|\|\|\|\|\|\| |  |
| Seq 2 516 | TAATTATTTT | 525 | Seq 2 | 1119 | ACCACCAAAAA | 1129 |

Alignment Length: 13; Identity: 13

Alignment Length: 10; Identity: 10

| Seq 1 854 | ATTTTCTCATTTT | 866 | Seq 1 | 1563 | AAATGATTTT | 1572 |
| --- | --- | --- | --- | --- | --- | --- |
|  | \|\|\|\|\|\|\|\|\|\|\|\|\| |  |  |  | \|\|\|\|\|\|\|\|\|\| |  |
| Seq 2 1769 | ATTTTCTCATTTT | 1781 | Seq 2 | 2024 | AAATGATTTT | 2033 |

Alignment Length: 10; Identity: 10

Alignment Length: 10; Identity: 10

| Seq 1 971 | GAAAAAAGAA | 980 | Seq 1 | 1565 | ATGATTTTAA | 1574 |
| --- | --- | --- | --- | --- | --- | --- |
|  | \|\|\|\|\|\|\|\|\|\| |  |  |  | \|\|\|\|\|\|\|\|\|\| |  |
| Seq 2 622 | GAAAAAAGAA | 631 | Seq 2 | 2224 | ATGATTTTAA | 2233 |

Alignment Length: 10; Identity: 10

Alignment Length: 10; Identity: 10

| Seq 1 978 | GAAAAATGAT | 987 | Seq 1 | 1607 | AATTTCCAAT | 1616 |
| --- | --- | --- | --- | --- | --- | --- |
|  | \|\|\|\|\|\|\|\|\|\| |  |  |  | \|\|\|\|\|\|\|\|\|\| |  |
| Seq 2 1321 | GAAAAATGAT | 1330 | Seq 2 | 363 | AATTTCCAAT | 372 |

Alignment Length: 11; Identity: 11

Alignment Length: 10; Identity: 10

| Seq 1 1040 | CAATTTTCTAA | 1050 | Seq 1 1786 | AACAAATTCA | 1795 |
| --- | --- | --- | --- | --- | --- |
|  | \|\|\|\|\|\|\|\|\|\|\| |  |  | \|\|\|\|\|\|\|\|\|\| |  |
| Seq 2 1693 | CAATTTTCTAA | 1703 | Seq 2 2583 | AACAAATTCA | 2592 |

Alignment Length: 10; Identity: 10

Alignment Length: 11; Identity: 11

| Seq 1 1069 | TTAAATTTTC | 1078 | Seq 1 | 1807 | AAATATCAATG | 1817 |
| --- | --- | --- | --- | --- | --- | --- |
|  | \|\|\|\|\|\|\|\|\|\| |  |  |  | \|\|\|\|\|\|\|\|\|\|\| |  |
| Seq 2 723 | TTAAATTTTC | 732 | Seq 2 | 1929 | AAATATCAATG | 1939 |

**S8 Figure, continued.** Motifs with identity between *C. elegans* and orthologous *mec-3* upstream sequences.

Alignment Length: 10; Identity: 10

Alignment Length: 10; Identity: 10

| Seq 1 1899 | AATTTTATTT | 1908 | Seq 1 | 2393 | TTTTCTTCTA | 2402 |
| --- | --- | --- | --- | --- | --- | --- |
|  | \|\|\|\|\|\|\|\|\|\| |  |  |  | \|\|\|\|\|\|\|\|\|\| |  |
| Seq 2 432 | AATTTTATTT | 441 | Seq 2 | 2884 | TTTTCTTCTA | 2893 |

Alignment Length: 13; Identity: 13

Alignment Length: 12; Identity: 12

| Seq 1 2328 | AGTTTTAAATTTT | 2340 | Seq 1 | 2434 | AATTCAAATTTT | 2445 |
| --- | --- | --- | --- | --- | --- | --- |
|  | \|\|\|\|\|\|\|\|\|\|\|\|\| |  |  |  | \|\|\|\|\|\|\|\|\|\|\|\| |  |
| Seq 2 719 | AGTTTTAAATTTT | 731 | Seq 2 | 2516 | AATTCAAATTTT | 2527 |

Alignment Length: 10; Identity: 10

Alignment Length: 11; Identity: 11

Seq 1 2475 CTTTTTTTTTT 2485

| Seq 1 2377 | GTTTTTTTTT | 2386 |  |  | \|\|\|\|\|\|\|\|\|\|\| |  |
| --- | --- | --- | --- | --- | --- | --- |
|  | \|\|\|\|\|\|\|\|\|\| |  | Seq 2 | 165 | CTTTTTTTTTT | 175 |
| Seq 2 1020 | GTTTTTTTTT | 1029 |  |  |  |  |

Alignment Length: 10; Identity: 10

Alignment Length: 10; Identity: 10

| Seq 1 2378 | TTTTTTTTTT | 2387 | Seq 1 | 2477 | TTTTTTTTTT | 2486 |
| --- | --- | --- | --- | --- | --- | --- |
|  | \|\|\|\|\|\|\|\|\|\| |  |  |  | \|\|\|\|\|\|\|\|\|\| |  |
| Seq 2 166 | TTTTTTTTTT | 175 | Seq 2 | 166 | TTTTTTTTTT | 175 |

Alignment Length: 10; Identity: 10

Alignment Length: 10; Identity: 10

| Seq 1 2379 | TTTTTTTTTT | 2388 | Seq 1 | 2478 | TTTTTTTTTT | 2487 |
| --- | --- | --- | --- | --- | --- | --- |
|  | \|\|\|\|\|\|\|\|\|\| |  |  |  | \|\|\|\|\|\|\|\|\|\| |  |
| Seq 2 166 | TTTTTTTTTT | 175 | Seq 2 | 166 | TTTTTTTTTT | 175 |

Alignment Length: 10; Identity: 10

Alignment Length: 10; Identity: 10

| Seq 1 2380 | TTTTTTTTTT | 2389 | Seq 1 | 2479 | TTTTTTTTTT | 2488 |
| --- | --- | --- | --- | --- | --- | --- |
|  | \|\|\|\|\|\|\|\|\|\| |  |  |  | \|\|\|\|\|\|\|\|\|\| |  |
| Seq 2 166 | TTTTTTTTTT | 175 | Seq 2 | 166 | TTTTTTTTTT | 175 |

Alignment Length: 10; Identity: 10

Alignment Length: 10; Identity: 10

| Seq 1 2381 | TTTTTTTTTT | 2390 | Seq 1 | 2480 | TTTTTTTTTT | 2489 |
| --- | --- | --- | --- | --- | --- | --- |
|  | \|\|\|\|\|\|\|\|\|\| |  |  |  | \|\|\|\|\|\|\|\|\|\| |  |
| Seq 2 166 | TTTTTTTTTT | 175 | Seq 2 | 166 | TTTTTTTTTT | 175 |

Alignment Length: 10; Identity: 10

Alignment Length: 10; Identity: 10

| Seq 1 2382 | TTTTTTTTTT | 2391 | Seq 1 | 2481 | TTTTTTTTTT | 2490 |
| --- | --- | --- | --- | --- | --- | --- |
|  | \|\|\|\|\|\|\|\|\|\| |  |  |  | \|\|\|\|\|\|\|\|\|\| |  |
| Seq 2 166 | TTTTTTTTTT | 175 | Seq 2 | 166 | TTTTTTTTTT | 175 |

Alignment Length: 10; Identity: 10

Alignment Length: 10; Identity: 10

| Seq 1 2383 | TTTTTTTTTT | 2392 | Seq 1 | 2482 | TTTTTTTTTT | 2491 |
| --- | --- | --- | --- | --- | --- | --- |
|  | \|\|\|\|\|\|\|\|\|\| |  |  |  | \|\|\|\|\|\|\|\|\|\| |  |
| Seq 2 166 | TTTTTTTTTT | 175 | Seq 2 | 166 | TTTTTTTTTT | 175 |

Alignment Length: 10; Identity: 10

Alignment Length: 10; Identity: 10

| Seq 1 2384 | TTTTTTTTTT | 2393 | Seq 1 | 2484 | TTTTTTTTGT | 2493 |
| --- | --- | --- | --- | --- | --- | --- |
|  | \|\|\|\|\|\|\|\|\|\| |  |  |  | \|\|\|\|\|\|\|\|\|\| |  |
| Seq 2 166 | TTTTTTTTTT | 175 | Seq 2 | 2476 | TTTTTTTTGT | 2485 |

Alignment Length: 10; Identity: 10

Alignment Length: 10; Identity: 10

| Seq 1 2385 | TTTTTTTTTT | 2394 | Seq 1 | 2502 | ATATAAATTC | 2511 |
| --- | --- | --- | --- | --- | --- | --- |
|  | \|\|\|\|\|\|\|\|\|\| |  |  |  | \|\|\|\|\|\|\|\|\|\| |  |
| Seq 2 166 | TTTTTTTTTT | 175 | Seq 2 | 1086 | ATATAAATTC | 1095 |

Alignment Length: 10; Identity: 10

**OPPOSITE STRAND**

| Seq 1 2386 | TTTTTTTTTT | 2395 Alignment Length: 10; Identity: 10 | | | | |
| --- | --- | --- | --- | --- | --- | --- |
|  | \|\|\|\|\|\|\|\|\|\| |  | Seq 1 | 2525 | ATTTGAAATC | 2516 |
| Seq 2 166 | TTTTTTTTTT | 175 |  |  | \|\|\|\|\|\|\|\|\|\| |  |
|  |  |  | Seq 2 | 554 | ATTTGAAATC | 563 |

Alignment Length: 10; Identity: 10

Seq 1 2387 TTTTTTTTTT 2396

Alignment Length: 10; Identity: 10

|  | \|\|\|\|\|\|\|\|\|\| |  | Seq 1 | 2512 | AGAATTTATA | 2503 |
| --- | --- | --- | --- | --- | --- | --- |
| Seq 2 166 | TTTTTTTTTT | 175 |  |  | \|\|\|\|\|\|\|\|\|\| |  |
|  |  |  | Seq 2 | 1134 | AGAATTTATA | 1143 |

**S8 Figure, continued.** Motifs with identity between *C. elegans* and orthologous *mec-3* upstream sequences.

Alignment Length: 10; Identity: 10

Alignment Length: 10; Identity: 10

| Seq 1 2504 | TATTATCAAA | 2495 | Seq 1 | 1773 | TTTTTTTTTA | 1764 |
| --- | --- | --- | --- | --- | --- | --- |
|  | \|\|\|\|\|\|\|\|\|\| |  |  |  | \|\|\|\|\|\|\|\|\|\| |  |
| Seq 2 469 | TATTATCAAA | 478 | Seq 2 | 167 | TTTTTTTTTA | 176 |

Alignment Length: 10; Identity: 10

Alignment Length: 11; Identity: 11

| Seq 1 2397 | GAAAAAAAAA | 2388 | Seq 1 | 1773 | TTTTTTTTTAA | 1763 |
| --- | --- | --- | --- | --- | --- | --- |
|  | \|\|\|\|\|\|\|\|\|\| |  |  |  | \|\|\|\|\|\|\|\|\|\|\| |  |
| Seq 2 1950 | GAAAAAAAAA | 1959 | Seq 2 | 1021 | TTTTTTTTTAA | 1031 |

Alignment Length: 11; Identity: 11

Alignment Length: 10; Identity: 10

| Seq 1 2386 | AAAAAAAAACC | 2376 | Seq 1 | 1688 | TTTTTTTTTT | 1679 |
| --- | --- | --- | --- | --- | --- | --- |
|  | \|\|\|\|\|\|\|\|\|\|\| |  |  |  | \|\|\|\|\|\|\|\|\|\| |  |
| Seq 2 1951 | AAAAAAAAACC | 1961 | Seq 2 | 166 | TTTTTTTTTT | 175 |

Alignment Length: 10; Identity: 10

Alignment Length: 10; Identity: 10

| Seq 1 2155 | TTATTTGAAA | 2146 | Seq 1 | 1687 | TTTTTTTTTT | 1678 |
| --- | --- | --- | --- | --- | --- | --- |
|  | \|\|\|\|\|\|\|\|\|\| |  |  |  | \|\|\|\|\|\|\|\|\|\| |  |
| Seq 2 552 | TTATTTGAAA | 561 | Seq 2 | 166 | TTTTTTTTTT | 175 |

Alignment Length: 10; Identity: 10

Alignment Length: 10; Identity: 10

| Seq 1 2116 | TTAATTTTAT | 2107 | Seq 1 | 1686 | TTTTTTTTTT | 1677 |
| --- | --- | --- | --- | --- | --- | --- |
|  | \|\|\|\|\|\|\|\|\|\| |  |  |  | \|\|\|\|\|\|\|\|\|\| |  |
| Seq 2 430 | TTAATTTTAT | 439 | Seq 2 | 166 | TTTTTTTTTT | 175 |

Alignment Length: 11; Identity: 11

Alignment Length: 10; Identity: 10

| Seq 1 2094 | AAATTGAAAAA | 2084 | Seq 1 | 1685 | TTTTTTTTTT | 1676 |
| --- | --- | --- | --- | --- | --- | --- |
|  | \|\|\|\|\|\|\|\|\|\|\| |  |  |  | \|\|\|\|\|\|\|\|\|\| |  |
| Seq 2 1945 | AAATTGAAAAA | 1955 | Seq 2 | 166 | TTTTTTTTTT | 175 |

Alignment Length: 10; Identity: 10

Alignment Length: 10; Identity: 10

| Seq 1 2075 | CAAATTGTTG | 2066 | Seq 1 | 1684 | TTTTTTTTTT | 1675 |
| --- | --- | --- | --- | --- | --- | --- |
|  | \|\|\|\|\|\|\|\|\|\| |  |  |  | \|\|\|\|\|\|\|\|\|\| |  |
| Seq 2 2169 | CAAATTGTTG | 2178 | Seq 2 | 166 | TTTTTTTTTT | 175 |

Alignment Length: 10; Identity: 10

Alignment Length: 10; Identity: 10

| Seq 1 2058 | TTCTATTTTT | 2049 | Seq 1 | 1683 | TTTTTTTTTT | 1674 |
| --- | --- | --- | --- | --- | --- | --- |
|  | \|\|\|\|\|\|\|\|\|\| |  |  |  | \|\|\|\|\|\|\|\|\|\| |  |
| Seq 2 1787 | TTCTATTTTT | 1796 | Seq 2 | 166 | TTTTTTTTTT | 175 |

Alignment Length: 11; Identity: 11

Alignment Length: 10; Identity: 10

| Seq 1 2045 | CTTTTTTTAAG | 2035 | Seq 1 | 1682 | TTTTTTTTTT | 1673 |
| --- | --- | --- | --- | --- | --- | --- |
|  | \|\|\|\|\|\|\|\|\|\|\| |  |  |  | \|\|\|\|\|\|\|\|\|\| |  |
| Seq 2 2337 | CTTTTTTTAAG | 2347 | Seq 2 | 166 | TTTTTTTTTT | 175 |

Alignment Length: 10; Identity: 10

Alignment Length: 10; Identity: 10

| Seq 1 1969 | TTTTATTTCA | 1960 | Seq 1 | 1681 | TTTTTTTTTT | 1672 |
| --- | --- | --- | --- | --- | --- | --- |
|  | \|\|\|\|\|\|\|\|\|\| |  |  |  | \|\|\|\|\|\|\|\|\|\| |  |
| Seq 2 136 | TTTTATTTCA | 145 | Seq 2 | 166 | TTTTTTTTTT | 175 |

Alignment Length: 10; Identity: 10

Alignment Length: 10; Identity: 10

| Seq 1 1930 | TTTTTTTAAT | 1921 | Seq 1 | 1680 | TTTTTTTTTT | 1671 |
| --- | --- | --- | --- | --- | --- | --- |
|  | \|\|\|\|\|\|\|\|\|\| |  |  |  | \|\|\|\|\|\|\|\|\|\| |  |
| Seq 2 1023 | TTTTTTTAAT | 1032 | Seq 2 | 166 | TTTTTTTTTT | 175 |

Alignment Length: 10; Identity: 10

Alignment Length: 10; Identity: 10

| Seq 1 1798 | AATTGAATTT | 1789 | Seq 1 1679 | TTTTTTTTTT | 1670 |
| --- | --- | --- | --- | --- | --- |
|  | \|\|\|\|\|\|\|\|\|\| |  |  | \|\|\|\|\|\|\|\|\|\| |  |
| Seq 2 1218 | AATTGAATTT | 1227 | Seq 2 166 | TTTTTTTTTT | 175 |

Alignment Length: 10; Identity: 10

Alignment Length: 10; Identity: 10

| Seq 1 1774 | CTTTTTTTTT | 1765 | Seq 1 | 1678 | TTTTTTTTTT | 1669 |
| --- | --- | --- | --- | --- | --- | --- |
|  | \|\|\|\|\|\|\|\|\|\| |  |  |  | \|\|\|\|\|\|\|\|\|\| |  |
| Seq 2 165 | CTTTTTTTTT | 174 | Seq 2 | 166 | TTTTTTTTTT | 175 |

**S8 Figure, continued.** Motifs with identity between *C. elegans* and orthologous *mec-3* upstream sequences.

Alignment Length: 10; Identity: 10

Alignment Length: 10; Identity: 10

| Seq 1 1677 | TTTTTTTTTT | 1668 | Seq 1 | 1560 | TTTTTTTTTT | 1551 |
| --- | --- | --- | --- | --- | --- | --- |
|  | \|\|\|\|\|\|\|\|\|\| |  |  |  | \|\|\|\|\|\|\|\|\|\| |  |
| Seq 2 166 | TTTTTTTTTT | 175 | Seq 2 | 166 | TTTTTTTTTT | 175 |

Alignment Length: 10; Identity: 10

Alignment Length: 10; Identity: 10

| Seq 1 1676 | TTTTTTTTTT | 1667 | Seq 1 | 1559 | TTTTTTTTTT | 1550 |
| --- | --- | --- | --- | --- | --- | --- |
|  | \|\|\|\|\|\|\|\|\|\| |  |  |  | \|\|\|\|\|\|\|\|\|\| |  |
| Seq 2 166 | TTTTTTTTTT | 175 | Seq 2 | 166 | TTTTTTTTTT | 175 |

Alignment Length: 10; Identity: 10

Alignment Length: 10; Identity: 10

| Seq 1 1675 | TTTTTTTTTT | 1666 | Seq 1 | 1558 | TTTTTTTTTT | 1549 |
| --- | --- | --- | --- | --- | --- | --- |
|  | \|\|\|\|\|\|\|\|\|\| |  |  |  | \|\|\|\|\|\|\|\|\|\| |  |
| Seq 2 166 | TTTTTTTTTT | 175 | Seq 2 | 166 | TTTTTTTTTT | 175 |

Alignment Length: 10; Identity: 10

Alignment Length: 10; Identity: 10

| Seq 1 1674 | TTTTTTTTTT | 1665 | Seq 1 | 1243 | AAAATTTTGA | 1234 |
| --- | --- | --- | --- | --- | --- | --- |
|  | \|\|\|\|\|\|\|\|\|\| |  |  |  | \|\|\|\|\|\|\|\|\|\| |  |
| Seq 2 166 | TTTTTTTTTT | 175 | Seq 2 | 832 | AAAATTTTGA | 841 |

Alignment Length: 10; Identity: 10

Alignment Length: 11; Identity: 11

| Seq 1 1673 | TTTTTTTTTT | 1664 | Seq 1 | 1108 | ACATTTAAATT | 1098 |
| --- | --- | --- | --- | --- | --- | --- |
|  | \|\|\|\|\|\|\|\|\|\| |  |  |  | \|\|\|\|\|\|\|\|\|\|\| |  |
| Seq 2 166 | TTTTTTTTTT | 175 | Seq 2 | 1814 | ACATTTAAATT | 1824 |

Alignment Length: 10; Identity: 10

Alignment Length: 10; Identity: 10

| Seq 1 1672 | TTTTTTTTTT | 1663 | Seq 1 | 1099 | TTTATCAGTT | 1090 |
| --- | --- | --- | --- | --- | --- | --- |
|  | \|\|\|\|\|\|\|\|\|\| |  |  |  | \|\|\|\|\|\|\|\|\|\| |  |
| Seq 2 166 | TTTTTTTTTT | 175 | Seq 2 | 1481 | TTTATCAGTT | 1490 |

Alignment Length: 11; Identity: 11

Alignment Length: 13; Identity: 13

| Seq 1 1671 | TTTTTTTTTTA | 1661 | Seq 1 | 1020 | ATTTATTTCATTT 1008 |
| --- | --- | --- | --- | --- | --- |
|  | \|\|\|\|\|\|\|\|\|\|\| |  |  |  | \|\|\|\|\|\|\|\|\|\|\|\|\| |
| Seq 2 166 | TTTTTTTTTTA | 176 | Seq 2 | 2919 | ATTTATTTCATTT 2931 |

Alignment Length: 12; Identity: 12

Alignment Length: 10; Identity: 10

| Seq 1 1670 | TTTTTTTTTAAT | 1659 | Seq 1 | 861 | GAGAAAATAA | 852 |
| --- | --- | --- | --- | --- | --- | --- |
|  | \|\|\|\|\|\|\|\|\|\|\|\| |  |  |  | \|\|\|\|\|\|\|\|\|\| |  |
| Seq 2 1021 | TTTTTTTTTAAT | 1032 | Seq 2 | 1972 | GAGAAAATAA | 1981 |

Alignment Length: 10; Identity: 10

Alignment Length: 10; Identity: 10

| Seq 1 1565 | TTTTTTTTTT | 1556 | Seq 1 | 848 | CAAAAATATA | 839 |
| --- | --- | --- | --- | --- | --- | --- |
|  | \|\|\|\|\|\|\|\|\|\| |  |  |  | \|\|\|\|\|\|\|\|\|\| |  |
| Seq 2 166 | TTTTTTTTTT | 175 | Seq 2 | 508 | CAAAAATATA | 517 |

Alignment Length: 10; Identity: 10

Alignment Length: 11; Identity: 11

| Seq 1 1564 | TTTTTTTTTT | 1555 | Seq 1 | 832 | TTGGTAATATT | 822 |
| --- | --- | --- | --- | --- | --- | --- |
|  | \|\|\|\|\|\|\|\|\|\| |  |  |  | \|\|\|\|\|\|\|\|\|\|\| |  |
| Seq 2 166 | TTTTTTTTTT | 175 | Seq 2 | 762 | TTGGTAATATT | 772 |

Alignment Length: 10; Identity: 10

Alignment Length: 13; Identity: 13

| Seq 1 1563 | TTTTTTTTTT | 1554 | Seq 1 | 789 | AATAAAATAGCAG 777 |
| --- | --- | --- | --- | --- | --- |
|  | \|\|\|\|\|\|\|\|\|\| |  |  |  | \|\|\|\|\|\|\|\|\|\|\|\|\| |
| Seq 2 166 | TTTTTTTTTT | 175 | Seq 2 | 243 | AATAAAATAGCAG 255 |

Alignment Length: 10; Identity: 10

Alignment Length: 10; Identity: 10

| Seq 1 1562 | TTTTTTTTTT | 1553 | Seq 1 702 | TTTTTTTTTT | 693 |
| --- | --- | --- | --- | --- | --- |
|  | \|\|\|\|\|\|\|\|\|\| |  |  | \|\|\|\|\|\|\|\|\|\| |  |
| Seq 2 166 | TTTTTTTTTT | 175 | Seq 2 166 | TTTTTTTTTT | 175 |

Alignment Length: 10; Identity: 10

Alignment Length: 10; Identity: 10

| Seq 1 1561 | TTTTTTTTTT | 1552 | Seq 1 | 466 | CGAAATTTCA | 457 |
| --- | --- | --- | --- | --- | --- | --- |
|  | \|\|\|\|\|\|\|\|\|\| |  |  |  | \|\|\|\|\|\|\|\|\|\| |  |
| Seq 2 166 | TTTTTTTTTT | 175 | Seq 2 | 735 | CGAAATTTCA | 744 |

**S8 Figure, continued.** Motifs with identity between *C. elegans* and orthologous *mec-3* upstream sequences.

Alignment Length: 10; Identity: 10

| Seq 1 394 | TTTTTGATTG | 385 |
| --- | --- | --- |
|  | \|\|\|\|\|\|\|\|\|\| |  |
| Seq 2 44 | TTTTTGATTG | 53 |

Alignment Length: 10; Identity: 10

| Seq 1 374 | TTCACTTTTT | 365 |
| --- | --- | --- |
|  | \|\|\|\|\|\|\|\|\|\| |  |
| Seq 2 142 | TTCACTTTTT | 151 |

Alignment Length: 11; Identity: 11

| Seq 1 329 | ATGAAATATCA | 319 |
| --- | --- | --- |
|  | \|\|\|\|\|\|\|\|\|\|\| |  |
| Seq 2 1926 | ATGAAATATCA | 1936 |

Alignment Length: 11; Identity: 11

| Seq 1 223 | TTTTCATCTTT | 213 |
| --- | --- | --- |
|  | \|\|\|\|\|\|\|\|\|\|\| |  |
| Seq 2 1226 | TTTTCATCTTT | 1236 |

Alignment Length: 10; Identity: 10

| Seq 1 160 | TATAATTATT | 151 |
| --- | --- | --- |
|  | \|\|\|\|\|\|\|\|\|\| |  |
| Seq 2 514 | TATAATTATT | 523 |

Alignment Length: 10; Identity: 10

| Seq 1 126 | AAATTGTTTT | 117 |
| --- | --- | --- |
|  | \|\|\|\|\|\|\|\|\|\| |  |
| Seq 2 597 | AAATTGTTTT | 606 |

Alignment Length: 10; Identity: 10

| Seq 1 | 91 | ATAATAATTC | 82 |
| --- | --- | --- | --- |
|  |  | \|\|\|\|\|\|\|\|\|\| |  |
| Seq 2 | 191 | ATAATAATTC | 200 |

**S8 Figure, continued.** Motifs with identity between *C. elegans* and orthologous *mec-3* upstream sequences.

**T. spiralis /C. elegans mec-3, 10bp window**

**seq 1: tspmec3**

**seq 2: celmec3**

Alignment Length: 10; Identity: 10

| Seq 1 186 | TTTCTATTAT | 195 |
| --- | --- | --- |
|  | \|\|\|\|\|\|\|\|\|\| |  |
| Seq 2 95 | TTTCTATTAT | 104 |

Alignment Length: 10; Identity: 10

| Seq 1 229 | ACTATCAAAA | 238 |
| --- | --- | --- |
|  | \|\|\|\|\|\|\|\|\|\| |  |
| Seq 2 997 | ACTATCAAAA | 1006 |

Alignment Length: 10; Identity: 10

| Seq 1 420 | GAATAGTTTT | 429 |
| --- | --- | --- |
|  | \|\|\|\|\|\|\|\|\|\| |  |
| Seq 2 586 | GAATAGTTTT | 595 |

Alignment Length: 12; Identity: 12

| Seq 1 438 | AAAACAAATAAA | 449 |
| --- | --- | --- |
|  | \|\|\|\|\|\|\|\|\|\|\|\| |  |
| Seq 2 1445 | AAAACAAATAAA | 1456 |

Alignment Length: 10; Identity: 10

| Seq 1 496 | AAAAAAACCC | 505 |
| --- | --- | --- |
|  | \|\|\|\|\|\|\|\|\|\| |  |
| Seq 2 1953 | AAAAAAACCC | 1962 |

Alignment Length: 11; Identity: 11

| Seq 1 546 | AAATAAGATTT | 556 |
| --- | --- | --- |
|  | \|\|\|\|\|\|\|\|\|\|\| |  |
| Seq 2 642 | AAATAAGATTT | 652 |

**OPPOSITE STRAND**

Alignment Length: 10; Identity: 10

| Seq 1 637 | CAAAGATCAA | 628 |
| --- | --- | --- |
|  | \|\|\|\|\|\|\|\|\|\| |  |
| Seq 2 1841 | CAAAGATCAA | 1850 |

Alignment Length: 10; Identity: 10

| Seq 1 423 | ATTCTCAAGT | 414 |
| --- | --- | --- |
|  | \|\|\|\|\|\|\|\|\|\| |  |
| Seq 2 2411 | ATTCTCAAGT | 2420 |

Alignment Length: 12; Identity: 12

| Seq 1 404 | AAATAAGATTTA | 393 |
| --- | --- | --- |
|  | \|\|\|\|\|\|\|\|\|\|\|\| |  |
| Seq 2 642 | AAATAAGATTTA | 653 |

Alignment Length: 11; Identity: 11

| Seq 1 367 | TGAAATTTTAT | 357 |
| --- | --- | --- |
|  | \|\|\|\|\|\|\|\|\|\|\| |  |
| Seq 2 1157 | TGAAATTTTAT | 1167 |

Alignment Length: 10; Identity: 10

| Seq 1 106 | ATGTTGAATT | 97 |
| --- | --- | --- |
|  | \|\|\|\|\|\|\|\|\|\| |  |
| Seq 2 694 | ATGTTGAATT | 703 |
