# Supplementary material for: Phylum-Level Conservation of Regulatory Information in Nematodes despite Extensive Non-coding Sequence Divergence
Source: PLoS Genet. 2015 May 28;11(5):e1005268. doi: 10.1371/journal.pgen.1005268 (PMC4447282; doi:10.1371/journal.pgen.1005268)
Supplement: S9 Fig — Motifs with identity between C. elegans and orthologous elt-2 upstream sequences. All blocks of sequence identity in window sizes shown for each comparison with positions within the upstream non-coding sequence. (DOCX) [file pgen.1005268.s009.docx]

**S9 Figure. Motifs with identity between *C. elegans* and orthologous *elt-2* upstream sequences.** Motifs with identity between *C. elegans* and orthologous *elt-2* upstream sequences. All blocks of sequence identity in window sizes shown for each comparison with positions within the upstream non-coding sequence.

**C. briggsae/C. elegans elt-2, 10bp window**

Alignment Length: 10; Identity: 10

|  | | Seq 1 | 626 | GTTAATTTTT | 635 |
| --- | --- | --- | --- | --- | --- |
| **seq 1:** | **cbrelt2** |  |  | \|\|\|\|\|\|\|\|\|\| |  |
| **seq 2:** | **celelt2** | Seq 2 | 3442 | GTTAATTTTT | 3451 |

Alignment Length: 23; Identity: 23

Alignment Length: 10; Identity: 10

| Seq 1 8 ACTCCAATGCACGCCATCGAACC | 30 | Seq 1 | 936 | TATCAAAACC | 945 |
| --- | --- | --- | --- | --- | --- |
| \|\|\|\|\|\|\|\|\|\|\|\|\|\|\|\|\|\|\|\|\|\|\| |  |  |  | \|\|\|\|\|\|\|\|\|\| |  |
| Seq 2 11 ACTCCAATGCACGCCATCGAACC | 33 | Seq 2 | 1574 | TATCAAAACC | 1583 |

Alignment Length: 10; Identity: 10

Alignment Length: 10; Identity: 10

| Seq 1 119 | ATCCTATTAC | 128 | Seq 1 | 944 | CCGGTATTCT | 953 |
| --- | --- | --- | --- | --- | --- | --- |
|  | \|\|\|\|\|\|\|\|\|\| |  |  |  | \|\|\|\|\|\|\|\|\|\| |  |
| Seq 2 115 | ATCCTATTAC | 124 | Seq 2 | 2363 | CCGGTATTCT | 2372 |

Alignment Length: 11; Identity: 11

Alignment Length: 43; Identity: 43

| Seq 1 173 | CAACTAATAAA | 183 | Seq 1 | 953 | TCTTTTTATACAGAATTGATAATGTTA 979 |
| --- | --- | --- | --- | --- | --- |
|  | \|\|\|\|\|\|\|\|\|\|\| |  |  |  | \|\|\|\|\|\|\|\|\|\|\|\|\|\|\|\|\|\|\|\|\|\|\|\|\|\|\| |
| Seq 2 177 | CAACTAATAAA | 187 | Seq 2 | 1591 | TCTTTTTATACAGAATTGATAATGTTA 1617 |

Alignment Length: 10; Identity: 10

Seq 1 235 TTTATTGCCT 244

||||||||||

Seq 2 241 TTTATTGCCT 250

Alignment Length: 19; Identity: 19

Seq 1 256 TTGTACCATCTAGTGTCTT 274

|||||||||||||||||||

Seq 2 256 TTGTACCATCTAGTGTCTT 274

Alignment Length: 11; Identity: 11

980 TCTTCAATTGATTTCT 995

||||||||||||||||

1618 TCTTCAATTGATTTCT 1633

Alignment Length: 18; Identity: 18

Seq 1 1007 TTCTGAGCTACGGCGATA 1024

||||||||||||||||||

Seq 2 1636 TTCTGAGCTACGGCGATA 1653

Alignment Length: 16; Identity: 16

Seq 1 1042 ACGATAATGTTGCCAT 1057

| Seq 1 309 | CTACTGATAAC | 319 |  |  | \|\|\|\|\|\|\|\|\|\|\|\|\|\|\|\| |
| --- | --- | --- | --- | --- | --- |
|  | \|\|\|\|\|\|\|\|\|\|\| |  | Seq 2 | 1670 | ACGATAATGTTGCCAT 1685 |
| Seq 2 288 | CTACTGATAAC | 298 |  |  |  |

Alignment Length: 11; Identity: 11

| Seq 1 344 | AATGCTTGGAA | 354 |  | \|\|\|\|\|\|\|\|\|\| |  |
| --- | --- | --- | --- | --- | --- |
|  | \|\|\|\|\|\|\|\|\|\|\| |  | Seq 2 554 | ATTTCTGATT | 563 |
| Seq 2 323 | AATGCTTGGAA | 333 |  |  |  |

Alignment Length: 10; Identity: 10

Seq 1 1059 ATTTCTGATT 1068

Alignment Length: 10; Identity: 10

| Seq 1 545 | ATGAGAAAGT | 554 |  |  | \|\|\|\|\|\|\|\|\|\|\|\| |  |
| --- | --- | --- | --- | --- | --- | --- |
|  | \|\|\|\|\|\|\|\|\|\| |  | Seq 2 | 1713 | GTTTCAGAACAC | 1724 |
| Seq 2 192 | ATGAGAAAGT | 201 |  |  |  |  |

Alignment Length: 12; Identity: 12

Seq 1 1083 GTTTCAGAACAC 1094

Alignment Length: 10; Identity: 10

| Seq 1 584 | ATTAATTTTT | 593 |  |  | \|\|\|\|\|\|\|\|\|\|\| |  |
| --- | --- | --- | --- | --- | --- | --- |
|  | \|\|\|\|\|\|\|\|\|\| |  | Seq 2 | 3160 | ACTGATAAGAA | 3170 |
| Seq 2 3429 | ATTAATTTTT | 3438 |  |  |  |  |

Alignment Length: 11; Identity: 11

Seq 1 1215 ACTGATAAGAA 1225

Alignment Length: 11; Identity: 11

| Seq 1 607 | ACTGAAAATTA | 617 |  |  | \|\|\|\|\|\|\|\|\|\|\|\|\|\|\|\|\| |
| --- | --- | --- | --- | --- | --- |
|  | \|\|\|\|\|\|\|\|\|\|\| |  | Seq 2 | 1845 | GTACTTCATTTCAAAAG 1861 |
| Seq 2 2129 | ACTGAAAATTA | 2139 |  |  |  |

Alignment Length: 17; Identity: 17

Seq 1 1230 GTACTTCATTTCAAAAG 1246

Alignment Length: 12; Identity: 12

Seq 1 620 AAAAATGTTAAT 631

||||||||||||

Seq 2 448 AAAAATGTTAAT 459

Alignment Length: 12; Identity: 12

Alignment Length: 21; Identity: 21

Seq 1 1259 AGCGCAAACATTGAGAAATGA 1279

|||||||||||||||||||||

Seq 2 1876 AGCGCAAACATTGAGAAATGA 1896

Alignment Length: 11; Identity: 11

Seq 1 1323 ACCTTGTACCC 1333

| Seq 1 621 | AAAATGTTAATT | 632 |  |  | \|\|\|\|\|\|\|\|\|\|\| |  |
| --- | --- | --- | --- | --- | --- | --- |
|  | \|\|\|\|\|\|\|\|\|\|\|\| |  | Seq 2 | 1937 | ACCTTGTACCC | 1947 |
| Seq 2 3464 | AAAATGTTAATT | 3475 |  |  |  |  |

**S9 Figure, continued.** Motifs with identity between *C. elegans* and orthologous *elt-2* upstream sequences.

Alignment Length: 10; Identity: 10

Alignment Length: 10; Identity: 10

| Seq 1 1367 | TTTTTTGAAA | 1376 | Seq 1 | 1742 | GTAACAAAAA | 1751 |
| --- | --- | --- | --- | --- | --- | --- |
|  | \|\|\|\|\|\|\|\|\|\| |  |  |  | \|\|\|\|\|\|\|\|\|\| |  |
| Seq 2 1182 | TTTTTTGAAA | 1191 | Seq 2 | 2339 | GTAACAAAAA | 2348 |

Alignment Length: 11; Identity: 11

Alignment Length: 10; Identity: 10

| Seq 1 1392 | ACTGAAAAAAA | 1402 | Seq 1 | 1778 | GTTTTTCTTT | 1787 |
| --- | --- | --- | --- | --- | --- | --- |
|  | \|\|\|\|\|\|\|\|\|\|\| |  |  |  | \|\|\|\|\|\|\|\|\|\| |  |
| Seq 2 569 | ACTGAAAAAAA | 579 | Seq 2 | 1175 | GTTTTTCTTT | 1184 |

Alignment Length: 11; Identity: 11

Alignment Length: 10; Identity: 10

| Seq 1 1437 | AATTATGGTGT | 1447 | Seq 1 | 2029 | ACTAATTTTT | 2038 |
| --- | --- | --- | --- | --- | --- | --- |
|  | \|\|\|\|\|\|\|\|\|\|\| |  |  |  | \|\|\|\|\|\|\|\|\|\| |  |
| Seq 2 2487 | AATTATGGTGT | 2497 | Seq 2 | 2645 | ACTAATTTTT | 2654 |

Alignment Length: 10; Identity: 10

Alignment Length: 10; Identity: 10

| Seq 1 1478 | TCTGATATTG | 1487 | Seq 1 | 2142 | TTTCATTATT | 2151 |
| --- | --- | --- | --- | --- | --- | --- |
|  | \|\|\|\|\|\|\|\|\|\| |  |  |  | \|\|\|\|\|\|\|\|\|\| |  |
| Seq 2 2070 | TCTGATATTG | 2079 | Seq 2 | 2176 | TTTCATTATT | 2185 |

Alignment Length: 10; Identity: 10

Alignment Length: 10; Identity: 10

| Seq 1 1489 | GGTGTGAAGT | 1498 | Seq 1 | 2163 | TTTTAATTTT | 2172 |
| --- | --- | --- | --- | --- | --- | --- |
|  | \|\|\|\|\|\|\|\|\|\| |  |  |  | \|\|\|\|\|\|\|\|\|\| |  |
| Seq 2 2083 | GGTGTGAAGT | 2092 | Seq 2 | 3510 | TTTTAATTTT | 3519 |

Alignment Length: 16; Identity: 16

Alignment Length: 12; Identity: 12

| Seq 1 1500 | ATATTATGTGCGTGTG | 1515 | Seq 1 | 2164 | TTTAATTTTCAA | 2175 |
| --- | --- | --- | --- | --- | --- | --- |
|  | \|\|\|\|\|\|\|\|\|\|\|\|\|\|\|\| |  |  |  | \|\|\|\|\|\|\|\|\|\|\|\| |  |
| Seq 2 2094 | ATATTATGTGCGTGTG | 2109 | Seq 2 | 2900 | TTTAATTTTCAA | 2911 |

Alignment Length: 10; Identity: 10

Alignment Length: 12; Identity: 12

| Seq 1 1542 | AATTTATCAA | 1551 | Seq 1 | 2172 | TCAAAAAAGTTT | 2183 |
| --- | --- | --- | --- | --- | --- | --- |
|  | \|\|\|\|\|\|\|\|\|\| |  |  |  | \|\|\|\|\|\|\|\|\|\|\|\| |  |
| Seq 2 2772 | AATTTATCAA | 2781 | Seq 2 | 1167 | TCAAAAAAGTTT | 1178 |

Alignment Length: 12; Identity: 12

Alignment Length: 10; Identity: 10

| Seq 1 1545 | TTATCAATTTTT | 1556 | Seq 1 | 2252 | TTTTGTTTCA | 2261 |
| --- | --- | --- | --- | --- | --- | --- |
|  | \|\|\|\|\|\|\|\|\|\|\|\| |  |  |  | \|\|\|\|\|\|\|\|\|\| |  |
| Seq 2 2137 | TTATCAATTTTT | 2148 | Seq 2 | 2746 | TTTTGTTTCA | 2755 |

Alignment Length: 11; Identity: 11

Alignment Length: 10; Identity: 10

| Seq 1 1562 | CAGGTTATCTT | 1572 | Seq 1 | 2299 | TGTTTTCATT | 2308 |
| --- | --- | --- | --- | --- | --- | --- |
|  | \|\|\|\|\|\|\|\|\|\|\| |  |  |  | \|\|\|\|\|\|\|\|\|\| |  |
| Seq 2 2152 | CAGGTTATCTT | 2162 | Seq 2 | 3603 | TGTTTTCATT | 3612 |

Alignment Length: 10; Identity: 10

Alignment Length: 11; Identity: 11

| Seq 1 1600 | GTTAAAAATG | 1609 | Seq 1 | 2306 | ATTTTCAAGTT | 2316 |
| --- | --- | --- | --- | --- | --- | --- |
|  | \|\|\|\|\|\|\|\|\|\| |  |  |  | \|\|\|\|\|\|\|\|\|\|\| |  |
| Seq 2 633 | GTTAAAAATG | 642 | Seq 2 | 2904 | ATTTTCAAGTT | 2914 |

Alignment Length: 10; Identity: 10

Alignment Length: 10; Identity: 10

| Seq 1 1600 | GTTAAAAATG | 1609 | Seq 1 | 2448 | AAAAAAAAAA | 2457 |
| --- | --- | --- | --- | --- | --- | --- |
|  | \|\|\|\|\|\|\|\|\|\| |  |  |  | \|\|\|\|\|\|\|\|\|\| |  |
| Seq 2 2541 | GTTAAAAATG | 2550 | Seq 2 | 1054 | AAAAAAAAAA | 1063 |

Alignment Length: 10; Identity: 10

Alignment Length: 10; Identity: 10

| Seq 1 1616 | TTTTTGAAAG | 1625 | Seq 1 2448 | AAAAAAAAAA | 2457 |
| --- | --- | --- | --- | --- | --- |
|  | \|\|\|\|\|\|\|\|\|\| |  |  | \|\|\|\|\|\|\|\|\|\| |  |
| Seq 2 1183 | TTTTTGAAAG | 1192 | Seq 2 1055 | AAAAAAAAAA | 1064 |

Alignment Length: 10; Identity: 10

Alignment Length: 10; Identity: 10

| Seq 1 1618 | TTTGAAAGTT | 1627 | Seq 1 | 2448 | AAAAAAAAAA | 2457 |
| --- | --- | --- | --- | --- | --- | --- |
|  | \|\|\|\|\|\|\|\|\|\| |  |  |  | \|\|\|\|\|\|\|\|\|\| |  |
| Seq 2 2867 | TTTGAAAGTT | 2876 | Seq 2 | 1056 | AAAAAAAAAA | 1065 |

**S9 Figure, continued.** Motifs with identity between *C. elegans* and orthologous *elt-2* upstream sequences.

Alignment Length: 10; Identity: 10

Alignment Length: 11; Identity: 11

| Seq 1 2448 | AAAAAAAAAA | 2457 | Seq 1 | 3091 | GTGAAAGTGAT | 3101 |
| --- | --- | --- | --- | --- | --- | --- |
|  | \|\|\|\|\|\|\|\|\|\| |  |  |  | \|\|\|\|\|\|\|\|\|\|\| |  |
| Seq 2 1057 | AAAAAAAAAA | 1066 | Seq 2 | 1139 | GTGAAAGTGAT | 1149 |

Alignment Length: 11; Identity: 11

Alignment Length: 10; Identity: 10

| Seq 1 2450 | AAAAAAAATTA | 2460 | Seq 1 | 3155 | TTTTGAATTT | 3164 |
| --- | --- | --- | --- | --- | --- | --- |
|  | \|\|\|\|\|\|\|\|\|\|\| |  |  |  | \|\|\|\|\|\|\|\|\|\| |  |
| Seq 2 2711 | AAAAAAAATTA | 2721 | Seq 2 | 3557 | TTTTGAATTT | 3566 |

Alignment Length: 11; Identity: 11

Alignment Length: 10; Identity: 10

| Seq 1 2538 | TATCAATTTTT | 2548 | Seq 1 | 3155 | TTTTGAATTT | 3164 |
| --- | --- | --- | --- | --- | --- | --- |
|  | \|\|\|\|\|\|\|\|\|\|\| |  |  |  | \|\|\|\|\|\|\|\|\|\| |  |
| Seq 2 2138 | TATCAATTTTT | 2148 | Seq 2 | 3565 | TTTTGAATTT | 3574 |

Alignment Length: 10; Identity: 10

Alignment Length: 10; Identity: 10

| Seq 1 2559 | CCACTGTTTT | 2568 | Seq 1 | 3158 | TGAATTTGAA | 3167 |
| --- | --- | --- | --- | --- | --- | --- |
|  | \|\|\|\|\|\|\|\|\|\| |  |  |  | \|\|\|\|\|\|\|\|\|\| |  |
| Seq 2 3354 | CCACTGTTTT | 3363 | Seq 2 | 859 | TGAATTTGAA | 868 |

Alignment Length: 10; Identity: 10

Alignment Length: 10; Identity: 10

| Seq 1 2559 | CCACTGTTTT | 2568 | Seq 1 | 3196 | ATTGAAAAAT | 3205 |
| --- | --- | --- | --- | --- | --- | --- |
|  | \|\|\|\|\|\|\|\|\|\| |  |  |  | \|\|\|\|\|\|\|\|\|\| |  |
| Seq 2 3599 | CCACTGTTTT | 3608 | Seq 2 | 608 | ATTGAAAAAT | 617 |

Alignment Length: 10; Identity: 10

Seq 1 2803 ACTGATAAGG 2812

||||||||||

Seq 2 1830 ACTGATAAGG 1839

Alignment Length: 10; Identity: 10

Seq 1 2854 GTCAAAAAAG 2863

||||||||||

Seq 2 1166 GTCAAAAAAG 1175

Alignment Length: 10; Identity: 10

Seq 1 2923 TTCTTCTACT 2932

||||||||||

Seq 2 4 TTCTTCTACT 13

Alignment Length: 10; Identity: 10

Seq 1 2927 TCTACTTATT 2936

||||||||||

Seq 2 2967 TCTACTTATT 2976

Alignment Length: 15; Identity: 15

Seq 1 2985 TTTTTGATAAAATCA 2999

|||||||||||||||

Seq 2 3283 TTTTTGATAAAATCA 3297

Alignment Length: 17; Identity: 17

Seq 1 3001 CCTATCTATACTTCCCA 3017

|||||||||||||||||

Seq 2 3299 CCTATCTATACTTCCCA 3315

Alignment Length: 13; Identity: 13

Seq 1 3017 AGTCTTATCGTTG 3029

|||||||||||||

Seq 2 3325 AGTCTTATCGTTG 3337

Alignment Length: 14; Identity: 14

Seq 1 3057 ACTGATATCTTCTA 3070

||||||||||||||

Seq 2 3365 ACTGATATCTTCTA 3378

**OPPOSITE STRAND**

Alignment Length: 11; Identity: 11

Seq 1 3366 AAAGTTAAAAA 3356

|||||||||||

Seq 2 2538 AAAGTTAAAAA 2548

Alignment Length: 10; Identity: 10

Seq 1 3310 AAAAAAAATT 3301

||||||||||

Seq 2 2711 AAAAAAAATT 2720

Alignment Length: 10; Identity: 10

Seq 1 3294 CAAGAAAATG 3285

||||||||||

Seq 2 3460 CAAGAAAATG 3469

Alignment Length: 10; Identity: 10

Seq 1 3164 AAATTCAAAA 3155

||||||||||

Seq 2 442 AAATTCAAAA 451

Alignment Length: 10; Identity: 10

Seq 1 2864 TCTTTTTTGA 2855

||||||||||

Seq 2 1180 TCTTTTTTGA 1189

Alignment Length: 10; Identity: 10

Seq 1 2680 AATGAGAAAG 2671

||||||||||

Seq 2 191 AATGAGAAAG 200

Alignment Length: 10; Identity: 10

Seq 1 2597 ATTGATAATG 2588

||||||||||

Seq 2 1605 ATTGATAATG 1614

Alignment Length: 11; Identity: 11

Seq 1 2590 ATGAATTTGAA 2580

|||||||||||

Seq 2 858 ATGAATTTGAA 868

**S9 Figure, continued.** Motifs with identity between *C. elegans* and orthologous *elt-2* upstream sequences.

Alignment Length: 11; Identity: 11

Alignment Length: 11; Identity: 11

| Seq 1 2461 | ATAATTTTTTT | 2451 | Seq 1 | 1065 | CAGAAATTATG | 1055 |
| --- | --- | --- | --- | --- | --- | --- |
|  | \|\|\|\|\|\|\|\|\|\|\| |  |  |  | \|\|\|\|\|\|\|\|\|\|\| |  |
| Seq 2 1695 | ATAATTTTTTT | 1705 | Seq 2 | 1129 | CAGAAATTATG | 1139 |

Alignment Length: 10; Identity: 10

Alignment Length: 10; Identity: 10

| Seq 1 2452 | TTTTTGATAA | 2443 | Seq 1 | 975 | ATTATCAATT | 966 |
| --- | --- | --- | --- | --- | --- | --- |
|  | \|\|\|\|\|\|\|\|\|\| |  |  |  | \|\|\|\|\|\|\|\|\|\| |  |
| Seq 2 3283 | TTTTTGATAA | 3292 | Seq 2 | 2136 | ATTATCAATT | 2145 |

Alignment Length: 10; Identity: 10

Alignment Length: 10; Identity: 10

| Seq 1 2218 | ACTTTTCATA | 2209 | Seq 1 | 750 | ATTTCTGATT | 741 |
| --- | --- | --- | --- | --- | --- | --- |
|  | \|\|\|\|\|\|\|\|\|\| |  |  |  | \|\|\|\|\|\|\|\|\|\| |  |
| Seq 2 2396 | ACTTTTCATA | 2405 | Seq 2 | 554 | ATTTCTGATT | 563 |

Alignment Length: 11; Identity: 11

Alignment Length: 10; Identity: 10

| Seq 1 2180 | CTTTTTTGAAA | 2170 | Seq 1 | 701 | TTTAAACTGC | 692 |
| --- | --- | --- | --- | --- | --- | --- |
|  | \|\|\|\|\|\|\|\|\|\|\| |  |  |  | \|\|\|\|\|\|\|\|\|\| |  |
| Seq 2 1181 | CTTTTTTGAAA | 1191 | Seq 2 | 3574 | TTTAAACTGC | 3583 |

Alignment Length: 10; Identity: 10

Alignment Length: 10; Identity: 10

| Seq 1 2166 | AAAAGTTTTT | 2157 | Seq 1 | 637 | TTAAAAATTA | 628 |
| --- | --- | --- | --- | --- | --- | --- |
|  | \|\|\|\|\|\|\|\|\|\| |  |  |  | \|\|\|\|\|\|\|\|\|\| |  |
| Seq 2 1171 | AAAAGTTTTT | 1180 | Seq 2 | 2509 | TTAAAAATTA | 2518 |

Alignment Length: 10; Identity: 10

Alignment Length: 10; Identity: 10

| Seq 1 2109 | TTTTCTTTGA | 2100 | Seq 1 | 540 | AAAAAAAAAC | 531 |
| --- | --- | --- | --- | --- | --- | --- |
|  | \|\|\|\|\|\|\|\|\|\| |  |  |  | \|\|\|\|\|\|\|\|\|\| |  |
| Seq 2 1290 | TTTTCTTTGA | 1299 | Seq 2 | 573 | AAAAAAAAAC | 582 |

Alignment Length: 10; Identity: 10

Alignment Length: 10; Identity: 10

| Seq 1 2098 | AGAAGCTCAC | 2089 | Seq 1 | 540 | AAAAAAAAAC | 531 |
| --- | --- | --- | --- | --- | --- | --- |
|  | \|\|\|\|\|\|\|\|\|\| |  |  |  | \|\|\|\|\|\|\|\|\|\| |  |
| Seq 2 1860 | AGAAGCTCAC | 1869 | Seq 2 | 1058 | AAAAAAAAAC | 1067 |

Alignment Length: 10; Identity: 10

Alignment Length: 11; Identity: 11

| Seq 1 1674 | AAGTGAAAAA | 1665 | Seq 1 | 539 | AAAAAAAACAT | 529 |
| --- | --- | --- | --- | --- | --- | --- |
|  | \|\|\|\|\|\|\|\|\|\| |  |  |  | \|\|\|\|\|\|\|\|\|\|\| |  |
| Seq 2 1356 | AAGTGAAAAA | 1365 | Seq 2 | 2375 | AAAAAAAACAT | 2385 |

Alignment Length: 10; Identity: 10

Alignment Length: 10; Identity: 10

| Seq 1 1648 | AAAAAAGTTT | 1639 | Seq 1 | 482 | ATTTAAGAAA | 473 |
| --- | --- | --- | --- | --- | --- | --- |
|  | \|\|\|\|\|\|\|\|\|\| |  |  |  | \|\|\|\|\|\|\|\|\|\| |  |
| Seq 2 1169 | AAAAAAGTTT | 1178 | Seq 2 | 3529 | ATTTAAGAAA | 3538 |

Alignment Length: 10; Identity: 10

Alignment Length: 10; Identity: 10

| Seq 1 1620 | AAAAACATAG | 1611 | Seq 1 | 446 | TTCAGAATTT | 437 |
| --- | --- | --- | --- | --- | --- | --- |
|  | \|\|\|\|\|\|\|\|\|\| |  |  |  | \|\|\|\|\|\|\|\|\|\| |  |
| Seq 2 2378 | AAAAACATAG | 2387 | Seq 2 | 981 | TTCAGAATTT | 990 |

Alignment Length: 10; Identity: 10

Alignment Length: 10; Identity: 10

| Seq 1 1418 | ATCAGAAAAT | 1409 | Seq 1 | 369 | CGTAAAACAC | 360 |
| --- | --- | --- | --- | --- | --- | --- |
|  | \|\|\|\|\|\|\|\|\|\| |  |  |  | \|\|\|\|\|\|\|\|\|\| |  |
| Seq 2 512 | ATCAGAAAAT | 521 | Seq 2 | 2324 | CGTAAAACAC | 2333 |

Alignment Length: 10; Identity: 10

Alignment Length: 11; Identity: 11

| Seq 1 1378 | CATTTCAAAA | 1369 | Seq 1 239 | ATAAAATGTTT | 229 |
| --- | --- | --- | --- | --- | --- |
|  | \|\|\|\|\|\|\|\|\|\| |  |  | \|\|\|\|\|\|\|\|\|\|\| |  |
| Seq 2 1851 | CATTTCAAAA | 1860 | Seq 2 838 | ATAAAATGTTT | 848 |

Alignment Length: 10; Identity: 10

| Seq 1 1122 | AAATGTTGAA | 1113 |
| --- | --- | --- |
|  | \|\|\|\|\|\|\|\|\|\| |  |
| Seq 2 2306 | AAATGTTGAA | 2315 |

**S9 Figure, continued.** Motifs with identity between *C. elegans* and orthologous *elt-2* upstream sequences.

**M. hapla/C. elegans elt-2, 10bp window**

**seq 1: mhaelt2**

**seq 2: celelt2**

Alignment Length: 10; Identity: 10

Alignment Length: 14; Identity: 14

Seq 1 455 TTAATAAATATAAA 468

||||||||||||||

Seq 2 1002 TTAATAAATATAAA 1015

Alignment Length: 10; Identity: 10

| Seq 1 32 | AATTGATAAT | 41 | Seq 1 | 512 | TTTTGATAAA | 521 |
| --- | --- | --- | --- | --- | --- | --- |
|  | \|\|\|\|\|\|\|\|\|\| |  |  |  | \|\|\|\|\|\|\|\|\|\| |  |
| Seq 2 1604 | AATTGATAAT | 1613 | Seq 2 | 3284 | TTTTGATAAA | 3293 |

Alignment Length: 11; Identity: 11

Alignment Length: 10; Identity: 10

| Seq 1 33 | ATTGATAATTA | 43 | Seq 1 | 528 | AATTCAAAAA | 537 |
| --- | --- | --- | --- | --- | --- | --- |
|  | \|\|\|\|\|\|\|\|\|\|\| |  |  |  | \|\|\|\|\|\|\|\|\|\| |  |
| Seq 2 2447 | ATTGATAATTA | 2457 | Seq 2 | 443 | AATTCAAAAA | 452 |

Alignment Length: 10; Identity: 10

Alignment Length: 10; Identity: 10

| Seq 1 108 | TTTAAAATTA | 117 | Seq 1 | 623 | AATAAATTAA | 632 |
| --- | --- | --- | --- | --- | --- | --- |
|  | \|\|\|\|\|\|\|\|\|\| |  |  |  | \|\|\|\|\|\|\|\|\|\| |  |
| Seq 2 2482 | TTTAAAATTA | 2491 | Seq 2 | 2810 | AATAAATTAA | 2819 |

Alignment Length: 11; Identity: 11

Alignment Length: 11; Identity: 11

| Seq 1 113 | AATTAATTTTT | 123 | Seq 1 | 684 | ATTAATTTTTG | 694 |
| --- | --- | --- | --- | --- | --- | --- |
|  | \|\|\|\|\|\|\|\|\|\|\| |  |  |  | \|\|\|\|\|\|\|\|\|\|\| |  |
| Seq 2 3428 | AATTAATTTTT | 3438 | Seq 2 | 3429 | ATTAATTTTTG | 3439 |

Alignment Length: 10; Identity: 10

Alignment Length: 10; Identity: 10

| Seq 1 116 | TAATTTTTTT | 125 | Seq 1 | 685 | TTAATTTTTG | 694 |
| --- | --- | --- | --- | --- | --- | --- |
|  | \|\|\|\|\|\|\|\|\|\| |  |  |  | \|\|\|\|\|\|\|\|\|\| |  |
| Seq 2 1696 | TAATTTTTTT | 1705 | Seq 2 | 3443 | TTAATTTTTG | 3452 |

Alignment Length: 10; Identity: 10

Alignment Length: 10; Identity: 10

| Seq 1 118 | ATTTTTTTGT | 127 | Seq 1 | 686 | TAATTTTTGA | 695 |
| --- | --- | --- | --- | --- | --- | --- |
|  | \|\|\|\|\|\|\|\|\|\| |  |  |  | \|\|\|\|\|\|\|\|\|\| |  |
| Seq 2 2990 | ATTTTTTTGT | 2999 | Seq 2 | 2647 | TAATTTTTGA | 2656 |

Alignment Length: 10; Identity: 10

Alignment Length: 10; Identity: 10

| Seq 1 119 | TTTTTTTGTT | 128 | Seq 1 | 754 | ATAATTTTTT | 763 |
| --- | --- | --- | --- | --- | --- | --- |
|  | \|\|\|\|\|\|\|\|\|\| |  |  |  | \|\|\|\|\|\|\|\|\|\| |  |
| Seq 2 2161 | TTTTTTTGTT | 2170 | Seq 2 | 1695 | ATAATTTTTT | 1704 |

Alignment Length: 10; Identity: 10

Alignment Length: 10; Identity: 10

| Seq 1 169 | GAAAACAATA | 178 | Seq 1 | 767 | AAAAATATAA | 776 |
| --- | --- | --- | --- | --- | --- | --- |
|  | \|\|\|\|\|\|\|\|\|\| |  |  |  | \|\|\|\|\|\|\|\|\|\| |  |
| Seq 2 498 | GAAAACAATA | 507 | Seq 2 | 1361 | AAAAATATAA | 1370 |

Alignment Length: 10; Identity: 10

Alignment Length: 11; Identity: 11

| Seq 1 244 | TAAATTTATT | 253 | Seq 1 | 804 | TACTCCTTATC | 814 |
| --- | --- | --- | --- | --- | --- | --- |
|  | \|\|\|\|\|\|\|\|\|\| |  |  |  | \|\|\|\|\|\|\|\|\|\|\| |  |
| Seq 2 1368 | TAAATTTATT | 1377 | Seq 2 | 2196 | TACTCCTTATC | 2206 |

Alignment Length: 11; Identity: 11

Alignment Length: 10; Identity: 10

| Seq 1 412 | AAATTAAAAAA | 422 | Seq 1 | 858 | TTTTTCTCTA | 867 |
| --- | --- | --- | --- | --- | --- | --- |
|  | \|\|\|\|\|\|\|\|\|\|\| |  |  |  | \|\|\|\|\|\|\|\|\|\| |  |
| Seq 2 1325 | AAATTAAAAAA | 1335 | Seq 2 | 1731 | TTTTTCTCTA | 1740 |

Alignment Length: 11; Identity: 11

Alignment Length: 10; Identity: 10

| Seq 1 412 | AAATTAAAAAA | 422 | Seq 1 1001 | AATATTTTCT | 1010 |
| --- | --- | --- | --- | --- | --- |
|  | \|\|\|\|\|\|\|\|\|\|\| |  |  | \|\|\|\|\|\|\|\|\|\| |  |
| Seq 2 2706 | AAATTAAAAAA | 2716 | Seq 2 3027 | AATATTTTCT | 3036 |

Alignment Length: 10; Identity: 10

Alignment Length: 10; Identity: 10

| Seq 1 414 | ATTAAAAAAG | 423 | Seq 1 | 1067 | ACTATAAACT | 1076 |
| --- | --- | --- | --- | --- | --- | --- |
|  | \|\|\|\|\|\|\|\|\|\| |  |  |  | \|\|\|\|\|\|\|\|\|\| |  |
| Seq 2 1099 | ATTAAAAAAG | 1108 | Seq 2 | 2612 | ACTATAAACT | 2621 |

**S9 Figure, continued.** Motifs with identity between *C. elegans* and orthologous *elt-2* upstream sequences.

Alignment Length: 11; Identity: 11

Alignment Length: 11; Identity: 11

| Seq 1 1126 | CCAAATTAAAA | 1136 | Seq 1 | 1736 | TTTTATCATTT | 1746 |
| --- | --- | --- | --- | --- | --- | --- |
|  | \|\|\|\|\|\|\|\|\|\|\| |  |  |  | \|\|\|\|\|\|\|\|\|\|\| |  |
| Seq 2 1323 | CCAAATTAAAA | 1333 | Seq 2 | 2049 | TTTTATCATTT | 2059 |

Alignment Length: 10; Identity: 10

Alignment Length: 10; Identity: 10

| Seq 1 1127 | CAAATTAAAA | 1136 | Seq 1 | 1771 | TAATTTTAAA | 1780 |
| --- | --- | --- | --- | --- | --- | --- |
|  | \|\|\|\|\|\|\|\|\|\| |  |  |  | \|\|\|\|\|\|\|\|\|\| |  |
| Seq 2 2705 | CAAATTAAAA | 2714 | Seq 2 | 1348 | TAATTTTAAA | 1357 |

Alignment Length: 12; Identity: 12

Alignment Length: 10; Identity: 10

| Seq 1 1230 | TTCTTTTCAAAC | 1241 | Seq 1 | 1771 | TAATTTTAAA | 1780 |
| --- | --- | --- | --- | --- | --- | --- |
|  | \|\|\|\|\|\|\|\|\|\|\|\| |  |  |  | \|\|\|\|\|\|\|\|\|\| |  |
| Seq 2 3239 | TTCTTTTCAAAC | 3250 | Seq 2 | 3513 | TAATTTTAAA | 3522 |

Alignment Length: 10; Identity: 10

Alignment Length: 12; Identity: 12

| Seq 1 1338 | TAAATAAAAA | 1347 | Seq 1 | 1780 | AATAAATATAAA | 1791 |
| --- | --- | --- | --- | --- | --- | --- |
|  | \|\|\|\|\|\|\|\|\|\| |  |  |  | \|\|\|\|\|\|\|\|\|\|\|\| |  |
| Seq 2 1049 | TAAATAAAAA | 1058 | Seq 2 | 1004 | AATAAATATAAA | 1015 |

Alignment Length: 10; Identity: 10

Alignment Length: 10; Identity: 10

| Seq 1 1366 | TTGTTTTATT | 1375 | Seq 1 | 1788 | TAAAAATTAT | 1797 |
| --- | --- | --- | --- | --- | --- | --- |
|  | \|\|\|\|\|\|\|\|\|\| |  |  |  | \|\|\|\|\|\|\|\|\|\| |  |
| Seq 2 2166 | TTGTTTTATT | 2175 | Seq 2 | 2510 | TAAAAATTAT | 2519 |

Alignment Length: 10; Identity: 10

Alignment Length: 10; Identity: 10

| Seq 1 1432 | ATAATAACTA | 1441 | Seq 1 | 1923 | AAAAAAATTA | 1932 |
| --- | --- | --- | --- | --- | --- | --- |
|  | \|\|\|\|\|\|\|\|\|\| |  |  |  | \|\|\|\|\|\|\|\|\|\| |  |
| Seq 2 3691 | ATAATAACTA | 3700 | Seq 2 | 2712 | AAAAAAATTA | 2721 |

Alignment Length: 10; Identity: 10

Alignment Length: 12; Identity: 12

| Seq 1 1462 | GAGCGCAAAC | 1471 | Seq 1 | 1928 | AATTAATTTTTG | 1939 |
| --- | --- | --- | --- | --- | --- | --- |
|  | \|\|\|\|\|\|\|\|\|\| |  |  |  | \|\|\|\|\|\|\|\|\|\|\|\| |  |
| Seq 2 1875 | GAGCGCAAAC | 1884 | Seq 2 | 3428 | AATTAATTTTTG | 3439 |

Alignment Length: 10; Identity: 10

Alignment Length: 10; Identity: 10

| Seq 1 1500 | AAAATATAAA | 1509 | Seq 1 | 1930 | TTAATTTTTG | 1939 |
| --- | --- | --- | --- | --- | --- | --- |
|  | \|\|\|\|\|\|\|\|\|\| |  |  |  | \|\|\|\|\|\|\|\|\|\| |  |
| Seq 2 1362 | AAAATATAAA | 1371 | Seq 2 | 3443 | TTAATTTTTG | 3452 |

Alignment Length: 10; Identity: 10

Alignment Length: 11; Identity: 11

| Seq 1 1672 | ATTAATTTTT | 1681 | Seq 1 | 1978 | ATTTTTGATTA | 1988 |
| --- | --- | --- | --- | --- | --- | --- |
|  | \|\|\|\|\|\|\|\|\|\| |  |  |  | \|\|\|\|\|\|\|\|\|\|\| |  |
| Seq 2 3429 | ATTAATTTTT | 3438 | Seq 2 | 2056 | ATTTTTGATTA | 2066 |

Alignment Length: 10; Identity: 10

Alignment Length: 10; Identity: 10

| Seq 1 1706 | ATTTAATTTT | 1715 | Seq 1 | 2052 | ATTATTATTA | 2061 |
| --- | --- | --- | --- | --- | --- | --- |
|  | \|\|\|\|\|\|\|\|\|\| |  |  |  | \|\|\|\|\|\|\|\|\|\| |  |
| Seq 2 2899 | ATTTAATTTT | 2908 | Seq 2 | 103 | ATTATTATTA | 112 |

Alignment Length: 12; Identity: 12

Alignment Length: 10; Identity: 10

| Seq 1 1707 | TTTAATTTTAAA | 1718 | Seq 1 | 2089 | TTTCTTTTTA | 2098 |
| --- | --- | --- | --- | --- | --- | --- |
|  | \|\|\|\|\|\|\|\|\|\|\|\| |  |  |  | \|\|\|\|\|\|\|\|\|\| |  |
| Seq 2 3511 | TTTAATTTTAAA | 3522 | Seq 2 | 1589 | TTTCTTTTTA | 1598 |

Alignment Length: 11; Identity: 11

Alignment Length: 10; Identity: 10

| Seq 1 1708 | TTAATTTTAAA | 1718 | Seq 1 2129 | TTTACTGATT | 2138 |
| --- | --- | --- | --- | --- | --- |
|  | \|\|\|\|\|\|\|\|\|\|\| |  |  | \|\|\|\|\|\|\|\|\|\| |  |
| Seq 2 1347 | TTAATTTTAAA | 1357 | Seq 2 1703 | TTTACTGATT | 1712 |

Alignment Length: 10; Identity: 10

Alignment Length: 10; Identity: 10

| Seq 1 1730 | TTATCTTTTT | 1739 | Seq 1 | 2162 | CTTTAAAATT | 2171 |
| --- | --- | --- | --- | --- | --- | --- |
|  | \|\|\|\|\|\|\|\|\|\| |  |  |  | \|\|\|\|\|\|\|\|\|\| |  |
| Seq 2 2156 | TTATCTTTTT | 2165 | Seq 2 | 2481 | CTTTAAAATT | 2490 |

**S9 Figure, continued.** Motifs with identity between *C. elegans* and orthologous *elt-2* upstream sequences.

Alignment Length: 10; Identity: 10

Alignment Length: 11; Identity: 11

| Seq 1 2194 | TTTAATAAAC | 2203 | Seq 1 | 2171 | AATTTTAAAGT | 2161 |
| --- | --- | --- | --- | --- | --- | --- |
|  | \|\|\|\|\|\|\|\|\|\| |  |  |  | \|\|\|\|\|\|\|\|\|\|\| |  |
| Seq 2 913 | TTTAATAAAC | 922 | Seq 2 | 1349 | AATTTTAAAGT | 1359 |

Alignment Length: 10; Identity: 10

Alignment Length: 10; Identity: 10

| Seq 1 2263 | ATTGTATTCT | 2272 | Seq 1 | 2171 | AATTTTAAAG | 2162 |
| --- | --- | --- | --- | --- | --- | --- |
|  | \|\|\|\|\|\|\|\|\|\| |  |  |  | \|\|\|\|\|\|\|\|\|\| |  |
| Seq 2 2183 | ATTGTATTCT | 2192 | Seq 2 | 3514 | AATTTTAAAG | 3523 |

Alignment Length: 10; Identity: 10

Alignment Length: 10; Identity: 10

| Seq 1 2349 | ACAAATTATT | 2358 | Seq 1 | 2105 | TTAATTGTAA | 2096 |
| --- | --- | --- | --- | --- | --- | --- |
|  | \|\|\|\|\|\|\|\|\|\| |  |  |  | \|\|\|\|\|\|\|\|\|\| |  |
| Seq 2 2671 | ACAAATTATT | 2680 | Seq 2 | 3470 | TTAATTGTAA | 3479 |

Alignment Length: 11; Identity: 11

Alignment Length: 11; Identity: 11

| Seq 1 2353 | ATTATTATTAT | 2363 | Seq 1 | 1935 | AATTAATTTTT | 1925 |
| --- | --- | --- | --- | --- | --- | --- |
|  | \|\|\|\|\|\|\|\|\|\|\| |  |  |  | \|\|\|\|\|\|\|\|\|\|\| |  |
| Seq 2 103 | ATTATTATTAT | 113 | Seq 2 | 3428 | AATTAATTTTT | 3438 |

Alignment Length: 10; Identity: 10

Alignment Length: 11; Identity: 11

| Seq 1 2359 | ATTATCAATT | 2368 | Seq 1 | 1932 | TAATTTTTTTA | 1922 |
| --- | --- | --- | --- | --- | --- | --- |
|  | \|\|\|\|\|\|\|\|\|\| |  |  |  | \|\|\|\|\|\|\|\|\|\|\| |  |
| Seq 2 2136 | ATTATCAATT | 2145 | Seq 2 | 1696 | TAATTTTTTTA | 1706 |

Alignment Length: 11; Identity: 11

Alignment Length: 10; Identity: 10

| Seq 1 2382 | TTAATTGTAAT | 2392 | Seq 1 | 1882 | GGGTGTGAAG | 1873 |
| --- | --- | --- | --- | --- | --- | --- |
|  | \|\|\|\|\|\|\|\|\|\|\| |  |  |  | \|\|\|\|\|\|\|\|\|\| |  |
| Seq 2 3470 | TTAATTGTAAT | 3480 | Seq 2 | 2082 | GGGTGTGAAG | 2091 |

**OPPOSITE STRAND**

Alignment Length: 10; Identity: 10

Alignment Length: 10; Identity: 10

Seq 1 1850 AAAGTTTTTC 1841

| Seq 1 2476 | ATAATTTTTT | 2467 |  |  | \|\|\|\|\|\|\|\|\|\| |  |
| --- | --- | --- | --- | --- | --- | --- |
|  | \|\|\|\|\|\|\|\|\|\| |  | Seq 2 | 1172 | AAAGTTTTTC | 1181 |
| Seq 2 1695 | ATAATTTTTT | 1704 |  |  |  |  |

Alignment Length: 10; Identity: 10

Alignment Length: 11; Identity: 11

Seq 1 1822 TTTTATTTTTC 1812

| Seq 1 2453 | TCTAAATAAA | 2444 |  |  | \|\|\|\|\|\|\|\|\|\|\| |  |
| --- | --- | --- | --- | --- | --- | --- |
|  | \|\|\|\|\|\|\|\|\|\| |  | Seq 2 | 2169 | TTTTATTTTTC | 2179 |
| Seq 2 1265 | TCTAAATAAA | 1274 |  |  |  |  |

Alignment Length: 12; Identity: 12

Alignment Length: 11; Identity: 11

Seq 1 1780 TTTAAAATTAT 1770

| Seq 1 2449 | AATAAATATAAA | 2438 |  |  | \|\|\|\|\|\|\|\|\|\|\| |  |
| --- | --- | --- | --- | --- | --- | --- |
|  | \|\|\|\|\|\|\|\|\|\|\|\| |  | Seq 2 | 2482 | TTTAAAATTAT | 2492 |
| Seq 2 1004 | AATAAATATAAA | 1015 |  |  |  |  |

Alignment Length: 10; Identity: 10

Alignment Length: 11; Identity: 11

Seq 1 1745 AATGATAAAAA 1735

| Seq 1 2409 | ATTAATTTTA | 2400 |  |  | \|\|\|\|\|\|\|\|\|\|\| |  |
| --- | --- | --- | --- | --- | --- | --- |
|  | \|\|\|\|\|\|\|\|\|\| |  | Seq 2 | 457 | AATGATAAAAA | 467 |
| Seq 2 1346 | ATTAATTTTA | 1355 |  |  |  |  |

Alignment Length: 10; Identity: 10

Alignment Length: 10; Identity: 10

Seq 1 1718 TTTAAAATTA 1709

| Seq 1 2368 | AATTGATAAT | 2359 |  |  | \|\|\|\|\|\|\|\|\|\| |  |
| --- | --- | --- | --- | --- | --- | --- |
|  | \|\|\|\|\|\|\|\|\|\| |  | Seq 2 | 2482 | TTTAAAATTA | 2491 |
| Seq 2 1604 | AATTGATAAT | 1613 |  |  |  |  |

Alignment Length: 10; Identity: 10

Seq 1 2279 AATAATCAGA 2270

||||||||||

Seq 2 615 AATAATCAGA 624

Alignment Length: 10; Identity: 10

Alignment Length: 10; Identity: 10

Seq 1 1707 ATCAGAAAAT 1698

||||||||||

Seq 2 512 ATCAGAAAAT 521

Alignment Length: 10; Identity: 10

Seq 1 1623 AATTTAATTT 1614

| Seq 1 2197 | TAAATTTATT | 2188 |  |  | \|\|\|\|\|\|\|\|\|\| |  |
| --- | --- | --- | --- | --- | --- | --- |
|  | \|\|\|\|\|\|\|\|\|\| |  | Seq 2 | 2898 | AATTTAATTT | 2907 |
| Seq 2 1368 | TAAATTTATT | 1377 |  |  |  |  |

**S9 Figure, continued.** Motifs with identity between *C. elegans* and orthologous *elt-2* upstream sequences.

Alignment Length: 10; Identity: 10

Alignment Length: 10; Identity: 10

| Seq 1 1616 | TTTAATAAAC | 1607 | Seq 1 | 662 | AATAAAATAG | 653 |
| --- | --- | --- | --- | --- | --- | --- |
|  | \|\|\|\|\|\|\|\|\|\| |  |  |  | \|\|\|\|\|\|\|\|\|\| |  |
| Seq 2 913 | TTTAATAAAC | 922 | Seq 2 | 3655 | AATAAAATAG | 3664 |

Alignment Length: 11; Identity: 11

Alignment Length: 10; Identity: 10

| Seq 1 1441 | TAGTTATTATA | 1431 | Seq 1 | 622 | TTTTCTTTGA | 613 |
| --- | --- | --- | --- | --- | --- | --- |
|  | \|\|\|\|\|\|\|\|\|\|\| |  |  |  | \|\|\|\|\|\|\|\|\|\| |  |
| Seq 2 2999 | TAGTTATTATA | 3009 | Seq 2 | 1290 | TTTTCTTTGA | 1299 |

Alignment Length: 10; Identity: 10

Alignment Length: 10; Identity: 10

| Seq 1 1289 | AACTATTATT | 1280 | Seq 1 | 548 | CTTTTTGATA | 539 |
| --- | --- | --- | --- | --- | --- | --- |
|  | \|\|\|\|\|\|\|\|\|\| |  |  |  | \|\|\|\|\|\|\|\|\|\| |  |
| Seq 2 99 | AACTATTATT | 108 | Seq 2 | 3282 | CTTTTTGATA | 3291 |

Alignment Length: 10; Identity: 10

Alignment Length: 10; Identity: 10

| Seq 1 1138 | TATTTTAATT | 1129 | Seq 1 | 537 | TTTTTGAATT | 528 |
| --- | --- | --- | --- | --- | --- | --- |
|  | \|\|\|\|\|\|\|\|\|\| |  |  |  | \|\|\|\|\|\|\|\|\|\| |  |
| Seq 2 3422 | TATTTTAATT | 3431 | Seq 2 | 3556 | TTTTTGAATT | 3565 |

Alignment Length: 10; Identity: 10

Alignment Length: 10; Identity: 10

| Seq 1 1100 | ATTATTATTA | 1091 | Seq 1 | 537 | TTTTTGAATT | 528 |
| --- | --- | --- | --- | --- | --- | --- |
|  | \|\|\|\|\|\|\|\|\|\| |  |  |  | \|\|\|\|\|\|\|\|\|\| |  |
| Seq 2 103 | ATTATTATTA | 112 | Seq 2 | 3564 | TTTTTGAATT | 3573 |

Alignment Length: 10; Identity: 10

Alignment Length: 10; Identity: 10

| Seq 1 1019 | ACTGATAAGA | 1010 | Seq 1 | 524 | CAATTTATCA | 515 |
| --- | --- | --- | --- | --- | --- | --- |
|  | \|\|\|\|\|\|\|\|\|\| |  |  |  | \|\|\|\|\|\|\|\|\|\| |  |
| Seq 2 3160 | ACTGATAAGA | 3169 | Seq 2 | 2004 | CAATTTATCA | 2013 |

Alignment Length: 12; Identity: 12

Alignment Length: 10; Identity: 10

| Seq 1 937 | TTTTTGTTTTAT | 926 | Seq 1 | 523 | AATTTATCAA | 514 |
| --- | --- | --- | --- | --- | --- | --- |
|  | \|\|\|\|\|\|\|\|\|\|\|\| |  |  |  | \|\|\|\|\|\|\|\|\|\| |  |
| Seq 2 2163 | TTTTTGTTTTAT | 2174 | Seq 2 | 2772 | AATTTATCAA | 2781 |

Alignment Length: 10; Identity: 10

Alignment Length: 11; Identity: 11

| Seq 1 917 | CTAATAAAGA | 908 | Seq 1 | 452 | ATAAACTTTGT | 442 |
| --- | --- | --- | --- | --- | --- | --- |
|  | \|\|\|\|\|\|\|\|\|\| |  |  |  | \|\|\|\|\|\|\|\|\|\|\| |  |
| Seq 2 180 | CTAATAAAGA | 189 | Seq 2 | 2615 | ATAAACTTTGT | 2625 |

Alignment Length: 11; Identity: 11

Alignment Length: 10; Identity: 10

| Seq 1 904 | AAAGTGATTTT | 894 | Seq 1 | 364 | TTCAGAATTT | 355 |
| --- | --- | --- | --- | --- | --- | --- |
|  | \|\|\|\|\|\|\|\|\|\|\| |  |  |  | \|\|\|\|\|\|\|\|\|\| |  |
| Seq 2 1142 | AAAGTGATTTT | 1152 | Seq 2 | 981 | TTCAGAATTT | 990 |

Alignment Length: 10; Identity: 10

Alignment Length: 10; Identity: 10

| Seq 1 780 | AAAATTATAT | 771 | Seq 1 | 303 | TGTATTTGTA | 294 |
| --- | --- | --- | --- | --- | --- | --- |
|  | \|\|\|\|\|\|\|\|\|\| |  |  |  | \|\|\|\|\|\|\|\|\|\| |  |
| Seq 2 2512 | AAAATTATAT | 2521 | Seq 2 | 2460 | TGTATTTGTA | 2469 |

Alignment Length: 10; Identity: 10

Alignment Length: 10; Identity: 10

| Seq 1 748 | AAATAAATAA | 739 | Seq 1 | 257 | TATAAATAAA | 248 |
| --- | --- | --- | --- | --- | --- | --- |
|  | \|\|\|\|\|\|\|\|\|\| |  |  |  | \|\|\|\|\|\|\|\|\|\| |  |
| Seq 2 1439 | AAATAAATAA | 1448 | Seq 2 | 1047 | TATAAATAAA | 1056 |

Alignment Length: 11; Identity: 11

Alignment Length: 11; Identity: 11

| Seq 1 746 | ATAAATAAAAA | 736 | Seq 1 252 | ATAAATTTACA | 242 |
| --- | --- | --- | --- | --- | --- |
|  | \|\|\|\|\|\|\|\|\|\|\| |  |  | \|\|\|\|\|\|\|\|\|\|\| |  |
| Seq 2 1048 | ATAAATAAAAA | 1058 | Seq 2 1011 | ATAAATTTACA | 1021 |

Alignment Length: 10; Identity: 10

Alignment Length: 10; Identity: 10

| Seq 1 741 | TAAAAATTAT | 732 | Seq 1 | 199 | TCAGAATTTT | 190 |
| --- | --- | --- | --- | --- | --- | --- |
|  | \|\|\|\|\|\|\|\|\|\| |  |  |  | \|\|\|\|\|\|\|\|\|\| |  |
| Seq 2 2510 | TAAAAATTAT | 2519 | Seq 2 | 982 | TCAGAATTTT | 991 |

**S9 Figure, continued.** Motifs with identity between *C. elegans* and orthologous *elt-2* upstream sequences.

Alignment Length: 11; Identity: 11

| Seq 1 148 | AAATTAAAAAA | 138 |
| --- | --- | --- |
|  | \|\|\|\|\|\|\|\|\|\|\| |  |
| Seq 2 1325 | AAATTAAAAAA | 1335 |

Alignment Length: 11; Identity: 11

| Seq 1 148 | AAATTAAAAAA | 138 |
| --- | --- | --- |
|  | \|\|\|\|\|\|\|\|\|\|\| |  |
| Seq 2 2706 | AAATTAAAAAA | 2716 |

Alignment Length: 10; Identity: 10

| Seq 1 127 | ACAAAAAAAT | 118 |
| --- | --- | --- |
|  | \|\|\|\|\|\|\|\|\|\| |  |
| Seq 2 765 | ACAAAAAAAT | 774 |

Alignment Length: 10; Identity: 10

| Seq 1 125 | AAAAAAATTA | 116 |
| --- | --- | --- |
|  | \|\|\|\|\|\|\|\|\|\| |  |
| Seq 2 2712 | AAAAAAATTA | 2721 |

Alignment Length: 10; Identity: 10

| Seq 1 120 | AATTAATTTT | 111 |
| --- | --- | --- |
|  | \|\|\|\|\|\|\|\|\|\| |  |
| Seq 2 3428 | AATTAATTTT | 3437 |

Alignment Length: 12; Identity: 12

| Seq 1 119 | ATTAATTTTAAA | 108 |
| --- | --- | --- |
|  | \|\|\|\|\|\|\|\|\|\|\|\| |  |
| Seq 2 1346 | ATTAATTTTAAA | 1357 |

Alignment Length: 11; Identity: 11

| Seq 1 118 | TTAATTTTAAA | 108 |
| --- | --- | --- |
|  | \|\|\|\|\|\|\|\|\|\|\| |  |
| Seq 2 3512 | TTAATTTTAAA | 3522 |

Alignment Length: 10; Identity: 10

| Seq 1 94 | TATAAATAAA | 85 |
| --- | --- | --- |
|  | \|\|\|\|\|\|\|\|\|\| |  |
| Seq 2 1047 | TATAAATAAA | 1056 |

Alignment Length: 12; Identity: 12

| Seq 1 81 | TTTATCATTTTT | 70 |
| --- | --- | --- |
|  | \|\|\|\|\|\|\|\|\|\|\|\| |  |
| Seq 2 2050 | TTTATCATTTTT | 2061 |

Alignment Length: 10; Identity: 10

| Seq 1 78 | ATCATTTTTA | 69 |
| --- | --- | --- |
|  | \|\|\|\|\|\|\|\|\|\| |  |
| Seq 2 3316 | ATCATTTTTA | 3325 |

Alignment Length: 10; Identity: 10

| Seq 1 73 | TTTTAATTAA | 64 |
| --- | --- | --- |
|  | \|\|\|\|\|\|\|\|\|\| |  |
| Seq 2 3424 | TTTTAATTAA | 3433 |

Alignment Length: 15; Identity: 15

Seq 1 42 AATTATCAATTTTTC 28

|||||||||||||||

Seq 2 2135 AATTATCAATTTTTC 2149

**S9 Figure, continued.** Motifs with identity between *C. elegans* and orthologous *elt-2* upstream sequences.

**B. malayi/C. elegans elt-2, 10bp window**

Alignment Length: 12; Identity: 12

|  | | Seq 1 | 977 | AAAAAAAAAAAC | 988 |
| --- | --- | --- | --- | --- | --- |
| **seq 1:** | **bmaelt2** |  |  | \|\|\|\|\|\|\|\|\|\|\|\| |  |
| **seq 2:** | **celelt2** | Seq 2 | 1056 | AAAAAAAAAAAC | 1067 |

Alignment Length: 10; Identity: 10

Alignment Length: 10; Identity: 10

| Seq 1 88 | TTTGCAAAAT | 97 | Seq 1 | 977 | AAAAAAAAAA | 986 |
| --- | --- | --- | --- | --- | --- | --- |
|  | \|\|\|\|\|\|\|\|\|\| |  |  |  | \|\|\|\|\|\|\|\|\|\| |  |
| Seq 2 2917 | TTTGCAAAAT | 2926 | Seq 2 | 1057 | AAAAAAAAAA | 1066 |

Alignment Length: 10; Identity: 10

Alignment Length: 10; Identity: 10

| Seq 1 216 | TCTTAGTTCT | 225 | Seq 1 | 978 | AAAAAAAAAA | 987 |
| --- | --- | --- | --- | --- | --- | --- |
|  | \|\|\|\|\|\|\|\|\|\| |  |  |  | \|\|\|\|\|\|\|\|\|\| |  |
| Seq 2 146 | TCTTAGTTCT | 155 | Seq 2 | 1054 | AAAAAAAAAA | 1063 |

Alignment Length: 15; Identity: 15

Alignment Length: 10; Identity: 10

| Seq 1 296 | AATTTTTGGAATGAG | 310 | Seq 1 | 979 | AAAAAAAAAC | 988 |
| --- | --- | --- | --- | --- | --- | --- |
|  | \|\|\|\|\|\|\|\|\|\|\|\|\|\|\| |  |  |  | \|\|\|\|\|\|\|\|\|\| |  |
| Seq 2 3445 | AATTTTTGGAATGAG | 3459 | Seq 2 | 573 | AAAAAAAAAC | 582 |

Alignment Length: 10; Identity: 10

Alignment Length: 10; Identity: 10

| Seq 1 546 | TAATTGTAAT | 555 | Seq 1 | 1234 | AAAATAGAAA | 1243 |
| --- | --- | --- | --- | --- | --- | --- |
|  | \|\|\|\|\|\|\|\|\|\| |  |  |  | \|\|\|\|\|\|\|\|\|\| |  |
| Seq 2 3471 | TAATTGTAAT | 3480 | Seq 2 | 3658 | AAAATAGAAA | 3667 |

Alignment Length: 11; Identity: 11

Alignment Length: 13; Identity: 13

| Seq 1 564 | ATGAGAGCAAA | 574 | Seq 1 | 1308 | CATGCAACTGATA 1320 |
| --- | --- | --- | --- | --- | --- |
|  | \|\|\|\|\|\|\|\|\|\|\| |  |  |  | \|\|\|\|\|\|\|\|\|\|\|\|\| |
| Seq 2 1893 | ATGAGAGCAAA | 1903 | Seq 2 | 1824 | CATGCAACTGATA 1836 |

Alignment Length: 11; Identity: 11

Alignment Length: 10; Identity: 10

| Seq 1 663 | ACTGATAAGGC | 673 | Seq 1 | 1328 | TATTATTATA | 1337 |
| --- | --- | --- | --- | --- | --- | --- |
|  | \|\|\|\|\|\|\|\|\|\|\| |  |  |  | \|\|\|\|\|\|\|\|\|\| |  |
| Seq 2 1830 | ACTGATAAGGC | 1840 | Seq 2 | 105 | TATTATTATA | 114 |

Alignment Length: 10; Identity: 10

Alignment Length: 11; Identity: 11

| Seq 1 833 | TACTTTTCAT | 842 | Seq 1 | 1336 | TATTGCCTCTC | 1346 |
| --- | --- | --- | --- | --- | --- | --- |
|  | \|\|\|\|\|\|\|\|\|\| |  |  |  | \|\|\|\|\|\|\|\|\|\|\| |  |
| Seq 2 2395 | TACTTTTCAT | 2404 | Seq 2 | 243 | TATTGCCTCTC | 253 |

Alignment Length: 10; Identity: 10

Alignment Length: 11; Identity: 11

| Seq 1 969 | AAAAAAAGAA | 978 | Seq 1 | 1538 | AGATTGAAAAC | 1548 |
| --- | --- | --- | --- | --- | --- | --- |
|  | \|\|\|\|\|\|\|\|\|\| |  |  |  | \|\|\|\|\|\|\|\|\|\|\| |  |
| Seq 2 869 | AAAAAAAGAA | 878 | Seq 2 | 622 | AGATTGAAAAC | 632 |

Alignment Length: 10; Identity: 10

Alignment Length: 10; Identity: 10

| Seq 1 976 | GAAAAAAAAA | 985 | Seq 1 | 1782 | TCGAATGTGA | 1791 |
| --- | --- | --- | --- | --- | --- | --- |
|  | \|\|\|\|\|\|\|\|\|\| |  |  |  | \|\|\|\|\|\|\|\|\|\| |  |
| Seq 2 572 | GAAAAAAAAA | 581 | Seq 2 | 302 | TCGAATGTGA | 311 |

Alignment Length: 10; Identity: 10

Alignment Length: 11; Identity: 11

| Seq 1 976 | GAAAAAAAAA | 985 | Seq 1 | 1882 | AATGATGATCA | 1892 |
| --- | --- | --- | --- | --- | --- | --- |
|  | \|\|\|\|\|\|\|\|\|\| |  |  |  | \|\|\|\|\|\|\|\|\|\|\| |  |
| Seq 2 866 | GAAAAAAAAA | 875 | Seq 2 | 467 | AATGATGATCA | 477 |

Alignment Length: 11; Identity: 11

Alignment Length: 11; Identity: 11

| Seq 1 977 | AAAAAAAAAAA | 987 | Seq 1 1950 | CGCTAACCAAT | 1960 |
| --- | --- | --- | --- | --- | --- |
|  | \|\|\|\|\|\|\|\|\|\|\| |  |  | \|\|\|\|\|\|\|\|\|\|\| |  |
| Seq 2 1054 | AAAAAAAAAAA | 1064 | Seq 2 723 | CGCTAACCAAT | 733 |

Alignment Length: 11; Identity: 11

Alignment Length: 10; Identity: 10

| Seq 1 977 | AAAAAAAAAAA | 987 | Seq 1 | 2098 | GAATGTGAAA | 2107 |
| --- | --- | --- | --- | --- | --- | --- |
|  | \|\|\|\|\|\|\|\|\|\|\| |  |  |  | \|\|\|\|\|\|\|\|\|\| |  |
| Seq 2 1055 | AAAAAAAAAAA | 1065 | Seq 2 | 304 | GAATGTGAAA | 313 |

**S9 Figure, continued.** Motifs with identity between *C. elegans* and orthologous *elt-2* upstream sequences.

Alignment Length: 10; Identity: 10

Alignment Length: 10; Identity: 10

| Seq 1 2298 | TGATTTTGAT | 2307 | Seq 1 | 3005 | TTCTTGAAAG | 2996 |
| --- | --- | --- | --- | --- | --- | --- |
|  | \|\|\|\|\|\|\|\|\|\| |  |  |  | \|\|\|\|\|\|\|\|\|\| |  |
| Seq 2 1281 | TGATTTTGAT | 1290 | Seq 2 | 2729 | TTCTTGAAAG | 2738 |

Alignment Length: 10; Identity: 10

Alignment Length: 10; Identity: 10

| Seq 1 2393 | AATGAGAGCA | 2402 | Seq 1 | 2873 | TCAAAAGAAG | 2864 |
| --- | --- | --- | --- | --- | --- | --- |
|  | \|\|\|\|\|\|\|\|\|\| |  |  |  | \|\|\|\|\|\|\|\|\|\| |  |
| Seq 2 1892 | AATGAGAGCA | 1901 | Seq 2 | 1855 | TCAAAAGAAG | 1864 |

Alignment Length: 10; Identity: 10

Alignment Length: 10; Identity: 10

| Seq 1 2482 | GTTTTCACTG | 2491 | Seq 1 | 2847 | TTATCAATTT | 2838 |
| --- | --- | --- | --- | --- | --- | --- |
|  | \|\|\|\|\|\|\|\|\|\| |  |  |  | \|\|\|\|\|\|\|\|\|\| |  |
| Seq 2 3359 | GTTTTCACTG | 3368 | Seq 2 | 2137 | TTATCAATTT | 2146 |

Alignment Length: 11; Identity: 11

Alignment Length: 10; Identity: 10

| Seq 1 2607 | ATAATTTTTTT | 2617 | Seq 1 | 2755 | ATGTTTAATA | 2746 |
| --- | --- | --- | --- | --- | --- | --- |
|  | \|\|\|\|\|\|\|\|\|\|\| |  |  |  | \|\|\|\|\|\|\|\|\|\| |  |
| Seq 2 1695 | ATAATTTTTTT | 1705 | Seq 2 | 910 | ATGTTTAATA | 919 |

Alignment Length: 10; Identity: 10

Alignment Length: 11; Identity: 11

| Seq 1 2614 | TTTTGATTTT | 2623 | Seq 1 | 2649 | AATAATATAAA | 2639 |
| --- | --- | --- | --- | --- | --- | --- |
|  | \|\|\|\|\|\|\|\|\|\| |  |  |  | \|\|\|\|\|\|\|\|\|\|\| |  |
| Seq 2 1278 | TTTTGATTTT | 1287 | Seq 2 | 434 | AATAATATAAA | 444 |

Alignment Length: 11; Identity: 11

Alignment Length: 10; Identity: 10

| Seq 1 2614 | TTTTGATTTTC | 2624 | Seq 1 | 2617 | AAAAAAATTA | 2608 |
| --- | --- | --- | --- | --- | --- | --- |
|  | \|\|\|\|\|\|\|\|\|\|\| |  |  |  | \|\|\|\|\|\|\|\|\|\| |  |
| Seq 2 1284 | TTTTGATTTTC | 1294 | Seq 2 | 2712 | AAAAAAATTA | 2721 |

Alignment Length: 10; Identity: 10

Alignment Length: 11; Identity: 11

| Seq 1 2614 | TTTTGATTTT | 2623 | Seq 1 | 2491 | CAGTGAAAACG | 2481 |
| --- | --- | --- | --- | --- | --- | --- |
|  | \|\|\|\|\|\|\|\|\|\| |  |  |  | \|\|\|\|\|\|\|\|\|\|\| |  |
| Seq 2 2740 | TTTTGATTTT | 2749 | Seq 2 | 2878 | CAGTGAAAACG | 2888 |

Alignment Length: 10; Identity: 10

Alignment Length: 10; Identity: 10

| Seq 1 2774 | AAATAGTTTT | 2783 | Seq 1 | 2225 | ACCAATTTAT | 2216 |
| --- | --- | --- | --- | --- | --- | --- |
|  | \|\|\|\|\|\|\|\|\|\| |  |  |  | \|\|\|\|\|\|\|\|\|\| |  |
| Seq 2 3504 | AAATAGTTTT | 3513 | Seq 2 | 2002 | ACCAATTTAT | 2011 |

Alignment Length: 10; Identity: 10

Alignment Length: 10; Identity: 10

| Seq 1 2791 | AACGGAGTCA | 2800 | Seq 1 | 2198 | TTGATAATTA | 2189 |
| --- | --- | --- | --- | --- | --- | --- |
|  | \|\|\|\|\|\|\|\|\|\| |  |  |  | \|\|\|\|\|\|\|\|\|\| |  |
| Seq 2 1402 | AACGGAGTCA | 1411 | Seq 2 | 2448 | TTGATAATTA | 2457 |

Alignment Length: 10; Identity: 10

Alignment Length: 11; Identity: 11

| Seq 1 3019 | CAAAAAAGAA | 3028 | Seq 1 | 2115 | TTCAGAATTTT | 2105 |
| --- | --- | --- | --- | --- | --- | --- |
|  | \|\|\|\|\|\|\|\|\|\| |  |  |  | \|\|\|\|\|\|\|\|\|\|\| |  |
| Seq 2 922 | CAAAAAAGAA | 931 | Seq 2 | 981 | TTCAGAATTTT | 991 |

**OPPOSITE STRAND**

Alignment Length: 11; Identity: 11

Alignment Length: 10; Identity: 10

Seq 1 2092 AAAAGTTCCA 2083

| Seq 1 3029 | TTTCTTTTTTG | 3019 |  |  | \|\|\|\|\|\|\|\|\|\| |  |
| --- | --- | --- | --- | --- | --- | --- |
|  | \|\|\|\|\|\|\|\|\|\|\| |  | Seq 2 | 2220 | AAAAGTTCCA | 2229 |
| Seq 2 1178 | TTTCTTTTTTG | 1188 |  |  |  |  |

Alignment Length: 10; Identity: 10

Seq 1 3010 TGATTTTCTT 3001

||||||||||

Seq 2 1287 TGATTTTCTT 1296

Alignment Length: 11; Identity: 11

Alignment Length: 12; Identity: 12

Seq 1 2082 TATCAAATTAAA 2071

||||||||||||

Seq 2 2702 TATCAAATTAAA 2713

Alignment Length: 10; Identity: 10

Seq 1 1941 TGTATTTGTA 1932

| Seq 1 3010 | TGATTTTCTTG | 3000 |  |  | \|\|\|\|\|\|\|\|\|\| |  |
| --- | --- | --- | --- | --- | --- | --- |
|  | \|\|\|\|\|\|\|\|\|\|\| |  | Seq 2 | 2460 | TGTATTTGTA | 2469 |
| Seq 2 3637 | TGATTTTCTTG | 3647 |  |  |  |  |

**S9 Figure, continued.** Motifs with identity between *C. elegans* and orthologous *elt-2* upstream sequences.

Alignment Length: 10; Identity: 10

Alignment Length: 10; Identity: 10

| Seq 1 1937 | TTTGTAGATT | 1928 | Seq 1 | 181 | ACAGTTTAAA | 172 |
| --- | --- | --- | --- | --- | --- | --- |
|  | \|\|\|\|\|\|\|\|\|\| |  |  |  | \|\|\|\|\|\|\|\|\|\| |  |
| Seq 2 547 | TTTGTAGATT | 556 | Seq 2 | 2504 | ACAGTTTAAA | 2513 |

Alignment Length: 10; Identity: 10

Alignment Length: 11; Identity: 11

| Seq 1 1602 | GCTCACTTAC | 1593 | Seq 1 | 158 | ATTATCTGATA | 148 |
| --- | --- | --- | --- | --- | --- | --- |
|  | \|\|\|\|\|\|\|\|\|\| |  |  |  | \|\|\|\|\|\|\|\|\|\|\| |  |
| Seq 2 1864 | GCTCACTTAC | 1873 | Seq 2 | 2066 | ATTATCTGATA | 2076 |

Alignment Length: 10; Identity: 10

Alignment Length: 11; Identity: 11

| Seq 1 1541 | ATCTAGCTTT | 1532 | Seq 1 | 95 | TTTGCAAAATT | 85 |
| --- | --- | --- | --- | --- | --- | --- |
|  | \|\|\|\|\|\|\|\|\|\| |  |  |  | \|\|\|\|\|\|\|\|\|\|\| |  |
| Seq 2 2659 | ATCTAGCTTT | 2668 | Seq 2 | 2917 | TTTGCAAAATT | 2927 |

Alignment Length: 10; Identity: 10

| Seq 1 1337 | TATAATAATA | 1328 |
| --- | --- | --- |
|  | \|\|\|\|\|\|\|\|\|\| |  |
| Seq 2 2431 | TATAATAATA | 2440 |

Alignment Length: 12; Identity: 12

| Seq 1 981 | TTTTTCTTTTTT | 970 |
| --- | --- | --- |
|  | \|\|\|\|\|\|\|\|\|\|\|\| |  |
| Seq 2 1176 | TTTTTCTTTTTT | 1187 |

Alignment Length: 10; Identity: 10

| Seq 1 980 | TTTTCTTTTT | 971 |
| --- | --- | --- |
|  | \|\|\|\|\|\|\|\|\|\| |  |
| Seq 2 1588 | TTTTCTTTTT | 1597 |

Alignment Length: 10; Identity: 10

| Seq 1 980 | TTTTCTTTTT | 971 |
| --- | --- | --- |
|  | \|\|\|\|\|\|\|\|\|\| |  |
| Seq 2 3278 | TTTTCTTTTT | 3287 |

Alignment Length: 10; Identity: 10

| Seq 1 761 | TAAAGAATTT | 752 |
| --- | --- | --- |
|  | \|\|\|\|\|\|\|\|\|\| |  |
| Seq 2 1271 | TAAAGAATTT | 1280 |

Alignment Length: 10; Identity: 10

| Seq 1 708 | GTTTTTCTCT | 699 |
| --- | --- | --- |
|  | \|\|\|\|\|\|\|\|\|\| |  |
| Seq 2 1730 | GTTTTTCTCT | 1739 |

Alignment Length: 11; Identity: 11

| Seq 1 630 | TATTACTATGT | 620 |
| --- | --- | --- |
|  | \|\|\|\|\|\|\|\|\|\|\| |  |
| Seq 2 2828 | TATTACTATGT | 2838 |

Alignment Length: 11; Identity: 11

| Seq 1 405 | TGTATGTTTTC | 395 |
| --- | --- | --- |
|  | \|\|\|\|\|\|\|\|\|\|\| |  |
| Seq 2 2466 | TGTATGTTTTC | 2476 |

Alignment Length: 10; Identity: 10

| Seq 1 386 | TTTTTCTACA | 377 |
| --- | --- | --- |
|  | \|\|\|\|\|\|\|\|\|\| |  |
| Seq 2 2144 | TTTTTCTACA | 2153 |

Alignment Length: 11; Identity: 11

| Seq 1 185 | ATTTACAGTTT | 175 |
| --- | --- | --- |
|  | \|\|\|\|\|\|\|\|\|\|\| |  |
| Seq 2 1015 | ATTTACAGTTT | 1025 |

**S9 Figure, continued.** Motifs with identity between *C. elegans* and orthologous *elt-2* upstream sequences.

**T. spiralis/C. elegans elt-2, 10bp window seq 1: tspelt2**

**seq 2: celelt2**

Alignment Length: 10; Identity: 10

| Seq 1 7 | TTGATAACCA | 16 |
| --- | --- | --- |
|  | \|\|\|\|\|\|\|\|\|\| |  |
| Seq 2 75 | TTGATAACCA | 84 |

Alignment Length: 12; Identity: 12

| Seq 1 254 | TTATTTTTCATT | 265 |
| --- | --- | --- |
|  | \|\|\|\|\|\|\|\|\|\|\|\| |  |
| Seq 2 2171 | TTATTTTTCATT | 2182 |

**OPPOSITE STRAND**

Alignment Length: 10; Identity: 10

| Seq 1 539 | TTATTATTAT | 530 |
| --- | --- | --- |
|  | \|\|\|\|\|\|\|\|\|\| |  |
| Seq 2 104 | TTATTATTAT | 113 |

Alignment Length: 12; Identity: 12

| Seq 1 538 | TATTATTATTAT | 527 |
| --- | --- | --- |
|  | \|\|\|\|\|\|\|\|\|\|\|\| |  |
| Seq 2 102 | TATTATTATTAT | 113 |

Alignment Length: 12; Identity: 12

| Seq 1 535 | TATTATTATTAT | 524 |
| --- | --- | --- |
|  | \|\|\|\|\|\|\|\|\|\|\|\| |  |
| Seq 2 102 | TATTATTATTAT | 113 |

Alignment Length: 10; Identity: 10

| Seq 1 532 | TATTATTATT | 523 |
| --- | --- | --- |
|  | \|\|\|\|\|\|\|\|\|\| |  |
| Seq 2 102 | TATTATTATT | 111 |

Alignment Length: 10; Identity: 10

| Seq 1 320 | AAATCAGCCT | 311 |
| --- | --- | --- |
|  | \|\|\|\|\|\|\|\|\|\| |  |
| Seq 2 3292 | AAATCAGCCT | 3301 |

Alignment Length: 10; Identity: 10

| Seq 1 263 | TGAAAAATAA | 254 |
| --- | --- | --- |
|  | \|\|\|\|\|\|\|\|\|\| |  |
| Seq 2 610 | TGAAAAATAA | 619 |

Alignment Length: 10; Identity: 10

| Seq 1 196 | GAGAGCAAAA | 187 |
| --- | --- | --- |
|  | \|\|\|\|\|\|\|\|\|\| |  |
| Seq 2 1895 | GAGAGCAAAA | 1904 |
