# Supplementary material for: Phylum-Level Conservation of Regulatory Information in Nematodes despite Extensive Non-coding Sequence Divergence
Source: PLoS Genet. 2015 May 28;11(5):e1005268. doi: 10.1371/journal.pgen.1005268 (PMC4447282; doi:10.1371/journal.pgen.1005268)
Supplement: S10 Fig — Graphs showing the proportion of sequence similarity over different window sizes for unc-25, mec-3, and elt-2. The number of identical 10 nt blocks between each of the nematode relatives and the C. elegans upstream sequence of the same genes shown in red, on histograms showing the number of predicted 10 nt matches between the relatives’ sequences and 1000 reshuffled C. elegans sequences that preserve tri-nucleotide frequencies. (PDF) [file pgen.1005268.s010.pdf]

Figure S10. Sequence identity in orthologous upstream sequences is not greater than expected by chance. See legend next page.

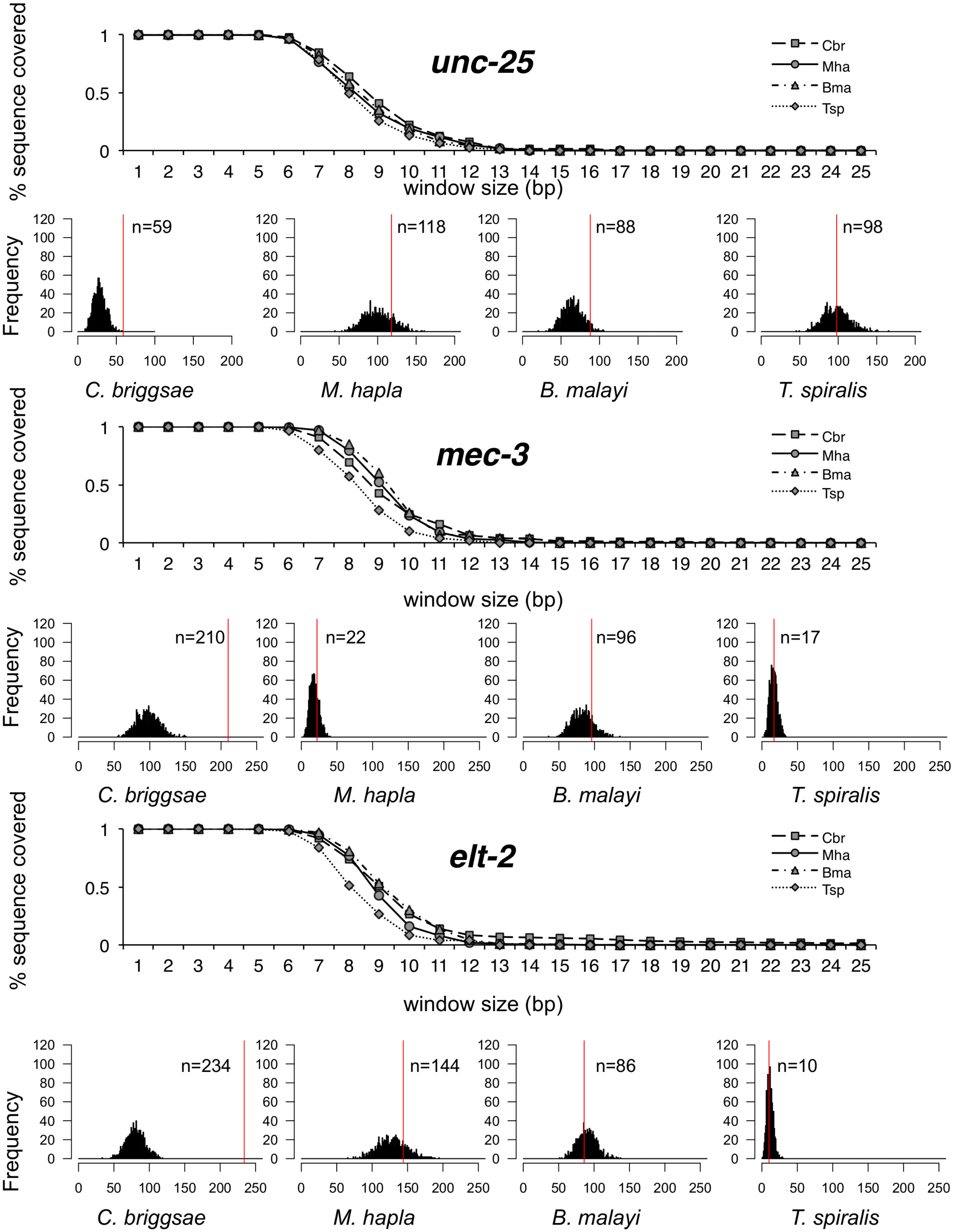

S10 Figure. Sequence identity in orthologous upstream sequences is not greater than expected by chance. Graphs showing the proportion of sequence similarity over different window sizes for *unc-25, mec-3,* and *elt-2*. The number of identical 10 nt blocks between each of the nematode relatives and the *C. elegans* upstream sequence of the same genes shown in red, on histograms showing the number of predicted 10 nt matches between the relatives' sequences and 1000 reshuffled *C. elegans* sequences that preserve tri-nucleotide frequencies.
