# Supplementary material for: Phylum-Level Conservation of Regulatory Information in Nematodes despite Extensive Non-coding Sequence Divergence
Source: PLoS Genet. 2015 May 28;11(5):e1005268. doi: 10.1371/journal.pgen.1005268 (PMC4447282; doi:10.1371/journal.pgen.1005268)
Supplement: S11 Fig — Cartoons depicting all orthologous upstream unc-25 sequences fused to mCherry (C. elegans) or GFP (all others) near the translation start site (bent arrow), or further downstream. Exons are thick black boxes, introns are gray lines. UNC-30 (box arrow) consensus motifs are shown above, in uppercase letters; conserved flanking nucleotides shown in lowercase. Locations of motifs relative to the endogenous translation start site are indicated. Daggers denote binding sites found to be bound by UNC-30. See S1 Text. (PDF) [file pgen.1005268.s011.pdf]

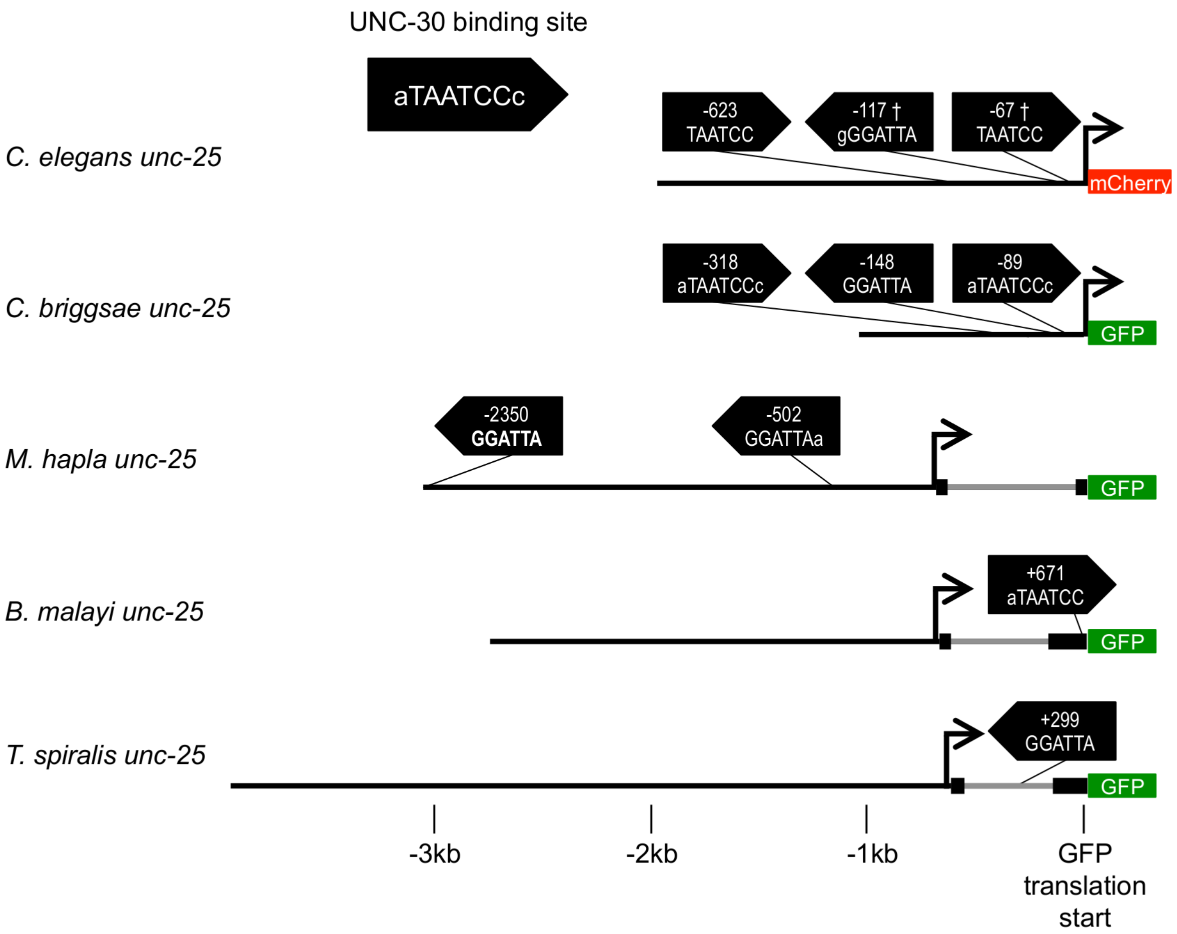

S11 Figure. Matches to motifs responsible for the *C. elegans unc-25* gene expression pattern can be found in orthlogous sequences. Cartoons depicting all orthologous upstream *unc-25* sequences fused to *mCherry* (*C. elegans*) or *GFP* (all others) near the translation start site (bent arrow), or further downstream. Exons are thick black boxes, introns are gray lines. UNC-30 (box arrow) consensus motifs are shown above, in uppercase letters; conserved flanking nucleotides shown in lowercase. Locations of motifs relative to the endogenous translation start site are indicated. Daggers denote binding sites found to be bound by UNC-30. See S1 Text.
