# Supplementary material for: Phylum-Level Conservation of Regulatory Information in Nematodes despite Extensive Non-coding Sequence Divergence
Source: PLoS Genet. 2015 May 28;11(5):e1005268. doi: 10.1371/journal.pgen.1005268 (PMC4447282; doi:10.1371/journal.pgen.1005268)
Supplement: S12 Fig — Cartoons depicting the all orthologous upstream mec-3 sequences fused to GFP near the translation start site (bent arrow) or further downstream. Exons are thick black boxes, introns are gray lines. UNC-86 (triangle) and MEC-3 (fletched arrow) consensus motifs are shown above. Locations of motifs relative to the endogenous translation start site are indicated. Several distal motifs are omitted from C. elegans, C. briggsae, and B. malayi. See S1 Text. (PDF) [file pgen.1005268.s012.pdf]

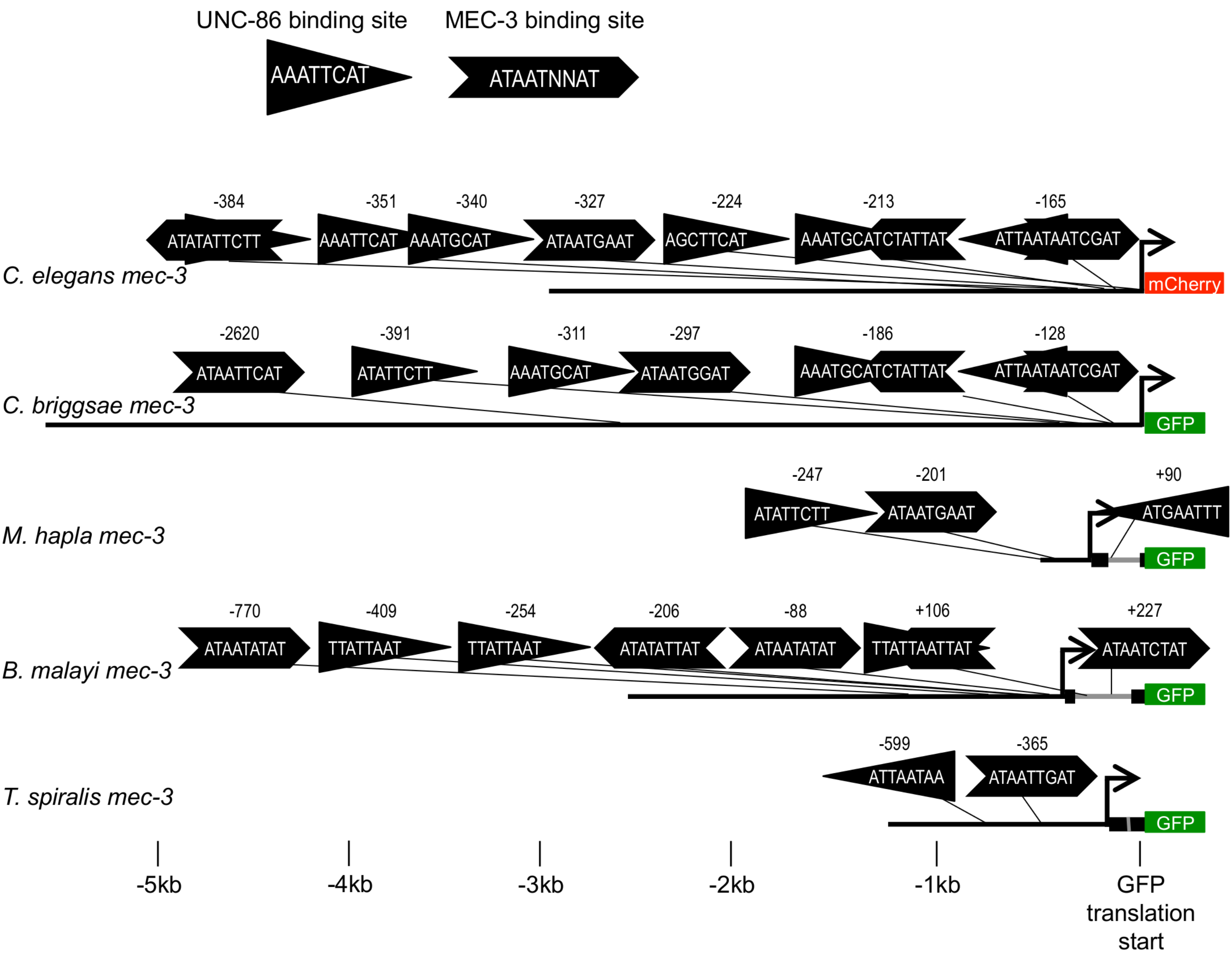

S12 Figure. Matches to motifs responsible for the *C. elegans mec-3* gene expression pattern can be found in orthlogous sequences. Cartoons depicting the all orthologous upstream *mec-3* sequences fused to *GFP* near the translation start site (bent arrow) or further downstream. Exons are thick black boxes, introns are gray lines. UNC-86 (triangle) and MEC-3 (fletched arrow) consensus motifs are shown above. Locations of motifs relative to the endogenous translation start site are indicated. Several distal motifs are omitted from *C. elegans, C. briggsae,* and *B. malayi.* See S1 Text.
