# Supplementary material for: Phylum-Level Conservation of Regulatory Information in Nematodes despite Extensive Non-coding Sequence Divergence
Source: PLoS Genet. 2015 May 28;11(5):e1005268. doi: 10.1371/journal.pgen.1005268 (PMC4447282; doi:10.1371/journal.pgen.1005268)
Supplement: S13 Fig — Cartoons depicting the all orthologous upstream elt-2 sequences fused to GFP near the translation start site (bent arrow). Gut-enriched extended GATA motif (sideways heart) and generic GATA motif (vertical lines) are shown above. Locations of motifs relative to the endogenous translation start site are indicated. See S1 Text. (PDF) [file pgen.1005268.s013.pdf]

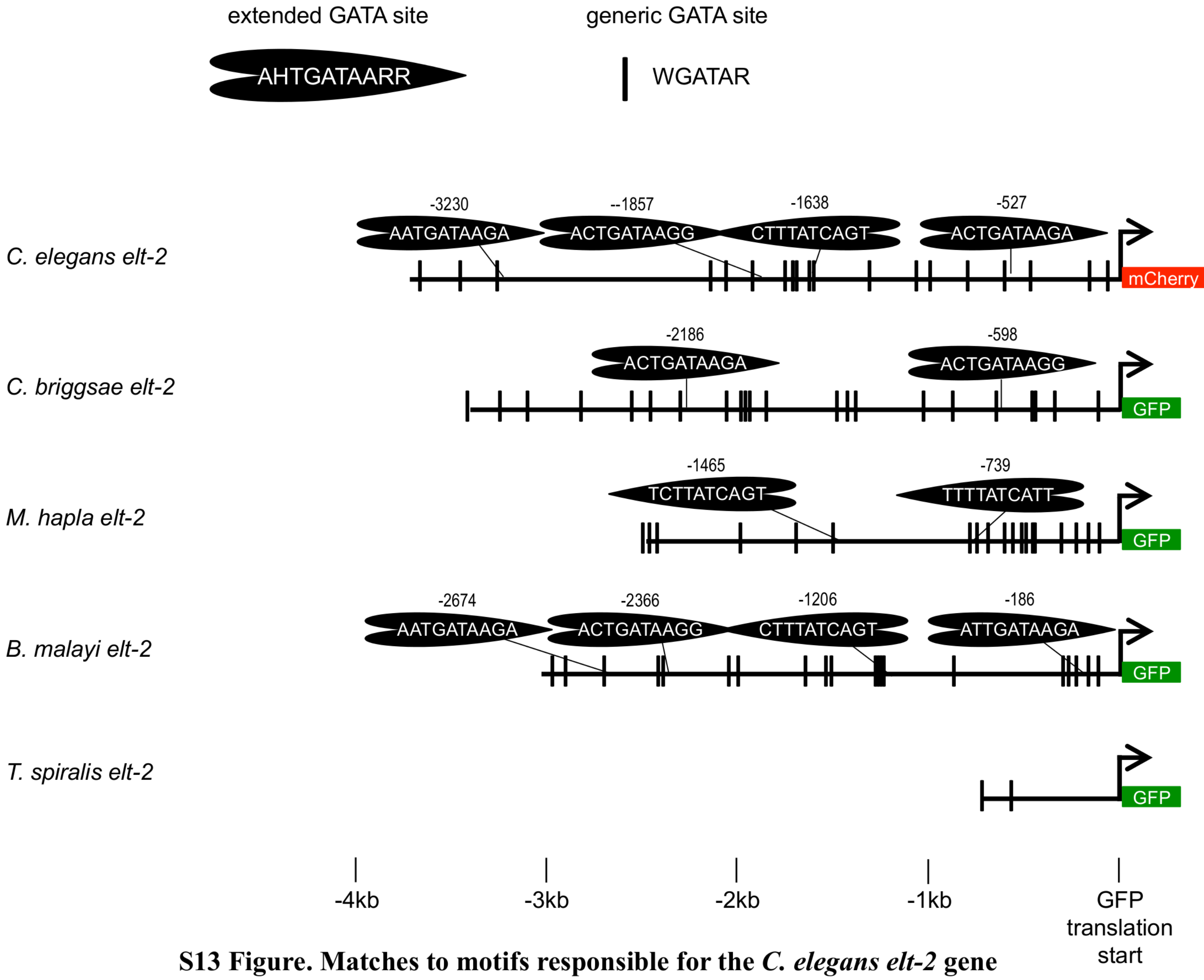

S13 Figure. Matches to motifs responsible for the *C. elegans elt-2* gene expression pattern can be found in orthlogous sequences. Cartoons depicting the all orthologous upstream *elt-2* sequences fused to *GFP* near the translation start site (bent arrow). Gut-enriched extended GATA motif (sideways heart) and generic GATA motif (vertical lines) are shown above. Locations of motifs relative to the endogenous translation start site are indicated. See S1 Text.
