# Supplementary material for: Phylum-Level Conservation of Regulatory Information in Nematodes despite Extensive Non-coding Sequence Divergence
Source: PLoS Genet. 2015 May 28;11(5):e1005268. doi: 10.1371/journal.pgen.1005268 (PMC4447282; doi:10.1371/journal.pgen.1005268)
Supplement: S14 Fig — (A) Mutant M. hapla unc-47 element drives expression of GFP. C. elegans unc-47 regulatory sequence drives expression of mCherry. Images are mosaics of single animals. Mutant T. spiralis unc-47 regulatory element drives expression of GFP, but not consistently in RIS (B) or DVB (C). μ denotes mutated unc-47 cis elements. C. elegans unc-47 regulatory sequence drives expression of mCherry in these cells. All animals photographed at 400x magnification. (PDF) [file pgen.1005268.s014.pdf]

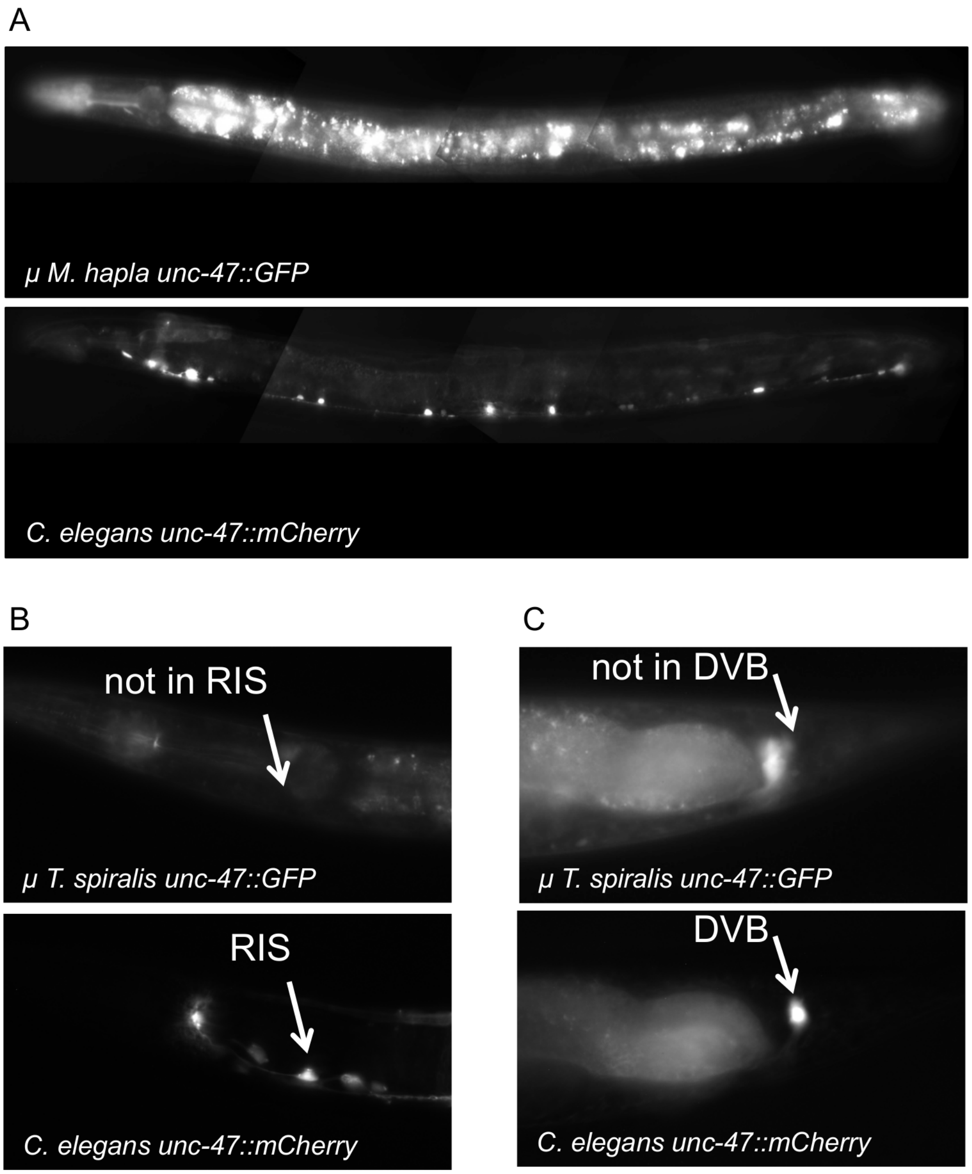

S14 Figure. Mutations to putative transcription factor binding sites in orthologous *cis*-regulatory sequences disrupt their functions. (A) Mutant *M. hapla unc-47* element drives expression of *GFP. C. elegans unc-47* regulatory sequence drives expression of *mCherry*. Images are mosaics of single animals. Mutant *T. spiralis unc-47* regulatory element drives expression of *GFP*, but not consistently in RIS (B) or DVB (C). μ denotes mutated *unc-47 cis* elements. *C. elegans unc-47* regulatory sequence drives expression of *mCherry* in these cells. All animals photographed at 400x magnification.
