# Supplementary material for: Phylum-Level Conservation of Regulatory Information in Nematodes despite Extensive Non-coding Sequence Divergence
Source: PLoS Genet. 2015 May 28;11(5):e1005268. doi: 10.1371/journal.pgen.1005268 (PMC4447282; doi:10.1371/journal.pgen.1005268)
Supplement: S15 Fig — (A) M. hapla unc-47::GFP is expressed in several D-type neurons of the ventral cord. (B) T. spiralis unc-47::GFP is expressed in RIS and DVB. (C) M. hapla mec-3::GFP is expressed in the head neuron FLP. (PDF) [file pgen.1005268.s015.pdf]

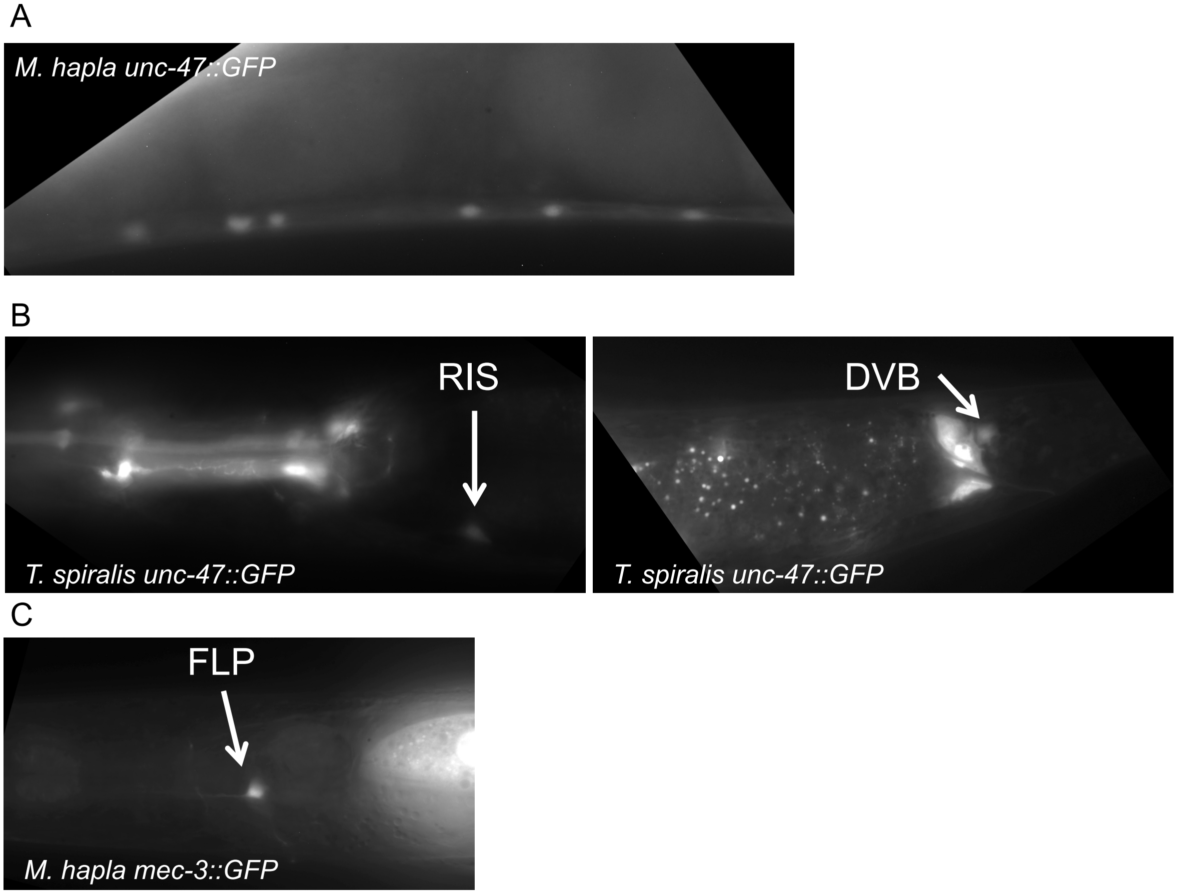

Figure S15. GFP expression driven by *cis* elements from distant relatives in the appropriate cells is detected when not coexpressed with mCherry. (A) *M. hapla unc-47::GFP* is expressed in several D-type neurons of the ventral cord. (B) *T. spiralis unc-47::GFP* is expressed in RIS and DVB. (C) *M. hapla mec-3::GFP* is expressed in the head neuron FLP. All images taken at 1000x magnification.
