# Supplementary material for: Phylum-Level Conservation of Regulatory Information in Nematodes despite Extensive Non-coding Sequence Divergence
Source: PLoS Genet. 2015 May 28;11(5):e1005268. doi: 10.1371/journal.pgen.1005268 (PMC4447282; doi:10.1371/journal.pgen.1005268)
Supplement: S1 Text — (DOCX) [file pgen.1005268.s019.docx]

**S1 Text. Short motifs with identity to *C. elegans* binding sites are present in promoters of all genes examined.**

The *C. elegans unc-25* promoter is regulated in D-type neurons by UNC-30, which binds one TAATCC motif on each strand [1]. All tested *unc-25* non-coding elements had UNC-30 binding site matches (Figure S11), though both *B. malayi* and *T. spiralis* had these motif matches in what appear to be introns, and the *M. hapla* match is on the reverse strand. *C. elegans* has an UNC-30 motif in the first intron, although that sequence was not part of the fusion construct tested here (or in [1–3]), and therefore is

not necessary to drive proper gene expression. The two upstream UNC-30 binding sites have been experimentally verified to bind UNC-30 [1], so some degree of redundancy is possible within the given motif types.

The *C. elegans* and *C. remanei mec-3* promoters both contain binding sites for MEC-3 and UNC-86 transcription factors [4], which bind to known motifs [5,6]. Matches to these motifs can be found in *C. briggsae* and all three of the distantly related nematodes (Figure S12).

Expression from the *C. elegans elt-2* promoter is initiated by binding by the GATA transcription factors END-1 and END-3 which bind the degenerate GATA-binding motif WGATAR [7]. Expression is then sustained by ELT-2 autoregulation [8] via a conserved extended GATA binding site AHTGATAARR [9], which is a more specific instance of the WGATAR motif. The upstream regulatory regions of the *elt-2* orthologs from *C. elegans, C. briggsae, M. hapla,* and *B. malayi* have many such GATA binding sites each (Figure S13). The *T. spiralis elt-2 cis*-element has only two WGATAR motifs, and no AHTGATAARR site. This sequence does not drive any discernable gene expression. The fact that *elt-2* is regulated by a cascade of GATA transcription factors makes it difficult to distinguish which factors bind which motifs during what phase of expression, but our experimental evidence indicates that the initiation phase of the *M. hapla* and *B. malayi elt-2* elements is intact in the *C. elegans* transgenic host, while the autoregulatory maintenance phase fails, despite presence of the gut specific GATA sites in these regulatory DNAs (Figure 5).

Each of the ortholog groups have motifs that are important for expression of the *C. elegans* promoters and are present in the distant relatives’ upstream sequences. Notably, in all cases these motifs are 10 bp or shorter, a window size in which the distant relatives are not dramatically different in their levels of conservation from *C. briggsae* (Figure 6E and Figures S7-S9).

References

1. Eastman C, Horvitz HR, Jin YS (1999) Coordinated transcriptional regulation of the unc-25 glutamic acid decarboxylase and the unc-47 GABA vesicular transporter by the Caenorhabditis elegans UNC-90 homeodomain protein. J Neurosci 19: 6225–6234.

2. Ruvinsky I, Ruvkun G (2003) Functional tests of enhancer conservation between distantly related species. Development 130: 5133–5142.

3. Barrière A, Ruvinsky I (2014) Pervasive Divergence of Transcriptional Gene Regulation in Caenorhabditis Nematodes. PLoS Genet 10: e1004435.

4. Way JC, Wang L, Run JQ, Wang a (1991) The mec-3 gene contains cis-acting elements mediating positive and negative regulation in cells produced by asymmetric cell division in Caenorhabditis elegans. Genes Dev 5: 2199–2211.

5. Wang L, Way JC (1996) Promoter sequences for the establishment of mec-3 expression in the nematode Caenorhabditis elegans. Mech Dev 56: 183–196.

6. Xue D, Finney M, Ruvkun G, Chalfie M (1992) Regulation of the mec-3 gene by the C.elegans homeoproteins UNC-86 and MEC-3. EMBO J 11: 4969–4979.

7. McGhee JD (2012) The Caenorhabditis elegans intestine. Wiley Interdiscip Rev Dev Biol 2: 347–367.

8. Fukushige T, Hendzel MJ, Bazett-Jones DP, McGhee JD (1999) Direct visualization of the elt-2 gut-specific GATA factor binding to a target promoter inside the living Caenorhabditis elegans embryo. Proc Natl Acad Sci U S A 96: 11883–11888.

9. McGhee JD, Sleumer MC, Bilenky M, Wong K, McKay SJ, et al. (2007) The ELT-2 GATA-factor and the global regulation of transcription in the C. elegans intestine. Dev Biol 302: 627–645.
